# Identification of the key genes and pathways involved in the tumorigenesis and prognosis of kidney renal clear cell carcinoma

**DOI:** 10.1038/s41598-020-61162-4

**Published:** 2020-03-06

**Authors:** Hao Cui, Hongjian Shan, Michael Zhe Miao, Zhiguo Jiang, Yuanyuan Meng, Ran Chen, Longzhen Zhang, Yong Liu

**Affiliations:** 1 Cancer Institute, Xuzhou Medical University, Xuzhou, Jiangsu 221000 China; 2 Department of Radiotherapy, Xuzhou Medical University Affiliated Hospital, Xuzhou, Jiangsu 221000 China; 3 Department of Orthopaedics, Xuzhou Medical University Affiliated Hospital, Xuzhou, Jiangsu 221000 China; 4 Department of Oral and Craniofacial Health Sciences, Adams School of Dentistry, University of North Carolina at Chapel Hill, Chapel Hill, NC 27516 USA; 5 Department of Cardiology, Affiliated Hospital of Nanjing University of Chinese Medicine, Nanjing, Jiangsu 210029 China; 6 Center of Clinical Oncology, Xuzhou Medical University Affiliated Hospital, Xuzhou, Jiangsu 221000 China

**Keywords:** Cancer genetics, Tumour biomarkers

## Abstract

Kidney renal clear cell carcinoma (KIRC) is the most common renal cell carcinoma (RCC). However, patients with KIRC usually have poor prognosis due to limited biomarkers for early detection and prognosis prediction. In this study, we analysed key genes and pathways involved in KIRC from an array dataset including 26 tumour and 26 adjacent normal tissue samples. Weighted gene co-expression network analysis (WGCNA) was performed with the WGCNA package, and 20 modules were characterized as having the highest correlation with KIRC. The upregulated genes in the tumour samples are involved in the innate immune response, whereas the downregulated genes contribute to the cellular catabolism of glucose, amino acids and fatty acids. Furthermore, the key genes were evaluated through a protein-protein interaction (PPI) network combined with a co-expression network. The comparatively lower expression of AGXT, PTGER3 and SLC12A3 in tumours correlates with worse prognosis in KIRC patients, while higher expression of ALOX5 predicts reduced survival. Our integrated analysis illustrated the hub genes involved in KIRC tumorigenesis, shedding light on the development of prognostic markers. Further understanding of the function of the identified KIRC hub genes could provide deep insights into the molecular mechanisms of KIRC.

## Introduction

The latest edition of the World Cancer Report shows that kidney cancer is the ninth most common cancer in men and the fourteenth most common cancer in women^1^. In 2018, there were more than 400,000 (2.2%) new cases and approximately 175,000 deaths from kidney cancer. There is also a clear trend that the rate of new kidney cancer diagnoses is increasing. KIRC is the most common renal carcinoma^2^. Moreover, more than 30% of patients diagnosed with KIRC experience metastasis. Among the deadliest cancer types, the five-year survival rate for metastatic KIRC is no more than 10%, with a median survival of only 13 months^3^. It has been widely acknowledged that the prognosis of patients with metastatic KIRC is extremely poor mainly because of the failure of early diagnosis and resistance to chemoradiotherapy^4^.

The pathogenesis of kidney cancer involves VHL, c-Met, BAP1, PBRM1 and other genes. Multiple targeted therapies have been implemented based on different molecular signatures, including agents against PDGF, VEGF, MET and immune checkpoint^5^. However, as KIRC exhibits heterogeneity, the efficacy of targeted therapies greatly varies among patients. Accordingly, the selection of individual therapeutic agents is a great challenge in clinical practice, so identifying novel biomarkers and predictive models for KIRC treatment is high on the agenda.

Systematic analysis of the KIRC gene signature to identify novel biomarkers of KIRC is necessary. It not only benefits KIRC diagnosis but also provides novel drug targets for KIRC treatment in the future. Gene co-expression network analysis has recently been employed to identify candidate genes associated with tumorigenesis^6^. The connectivity among different candidate genes can also be evaluated by using weighted gene co-expression network analysis (WGCNA). Based on a microarray dataset (GSE66272) extracted from the Gene Expression Omnibus database, we constructed a gene co-expression network as well as a protein-protein interaction (PPI) network to screen candidate genes involved in KIRC tumorigenesis. Four genes, including AGXT, PTGER3, SLC12A3 and ALOX5, revealed a strong correlation with KIRC development, as supported by survival analysis of the TCGA kidney cancer dataset and validated by RT-qPCR in an independent cohort of patients with primary KIRC.

## Results

### Identification of differentially expressed genes between KIRC patients and normal controls

**Figure 1:**
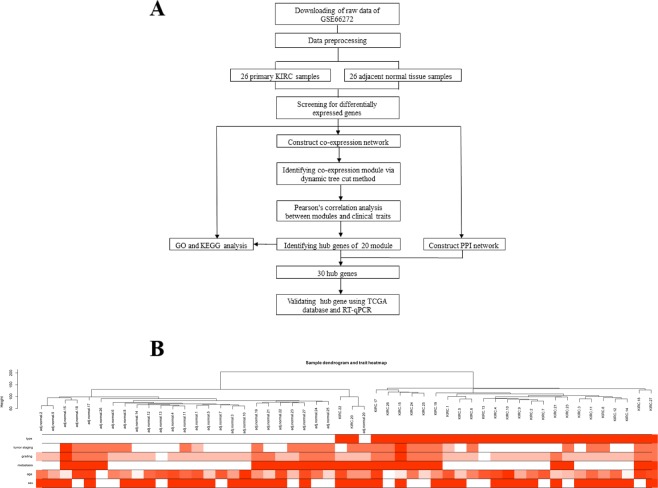
Study design and clustering dendrogram of the patient samples and clinical traits. (**A**) Flow diagram of the analysis procedure: data collection, preprocessing, analysis and validation. (**B**) Clustering was based on the expression data of the differentially expressed genes between KIRC (n = 26) and adjacent normal (n = 26) tissues. The red colour represents the tumour, metastasis and male. Colour intensity is proportional to tumour staging, grading, and age.

The workflow of our study is shown in Fig. 1A. After data preprocessing and quality assessment by the WGCNA R package, no sample was removed from subsequent analysis in the training set (GSE66272), in which the expression matrices were obtained from 26 tumour and 26 adjacent normal tissue samples (Fig. 1B). Under the threshold of FDR < 0.05 and adjusted p value < 0.0001, a total of 8763 differentially expressed genes (DEGs) were screened out for subsequent analysis, with the the minimum log |FC| is 0.349. Among these DEGs, 4287 genes were upregulated and 4476 genes were downregulated in tumours (Supplementary Table S4).

### GO and KEGG pathway analysis of DEGs

We classified all DEGs into clusters with the same GO terms and KEGG pathway functions through the DAVID website. As indicated by the GO term analysis results in Supplementary Table S2, the upregulated genes in tumours were mainly enriched in signal transduction, immune response, apoptotic process, innate immune response, and inflammatory response assembly, while the downregulated genes were enriched in the oxidation-reduction process, transport, transmembrane transport, intracellular signal transduction and metabolic process.

As shown in Supplementary Table S3, the upregulated DEGs were enriched in pathways in cancer, PI3K-Akt signalling pathway, HTLV-I infection, viral carcinogenesis, cytokine-cytokine receptor interaction, phagosome, tuberculosis, herpes simplex infection, systemic lupus erythematosus and influenza A, whereas the downregulated DEGs were enriched in metabolic pathways, biosynthesis of antibiotics, carbon metabolism, valine, leucine and isoleucine degradation, glycine, serine and threonine metabolism, peroxisome, fatty acid degradation, protein digestion and absorption, PPAR signalling pathway and tight junction. Collectively, both KEGG and GO analyses strongly indicate that the expression of genes involved in immune responses was substantially increased, whereas the expression of genes associated with cellular catabolism was significantly reduced.

### Construction of weighted co-expression network to identify key modules

**Figure 2:**
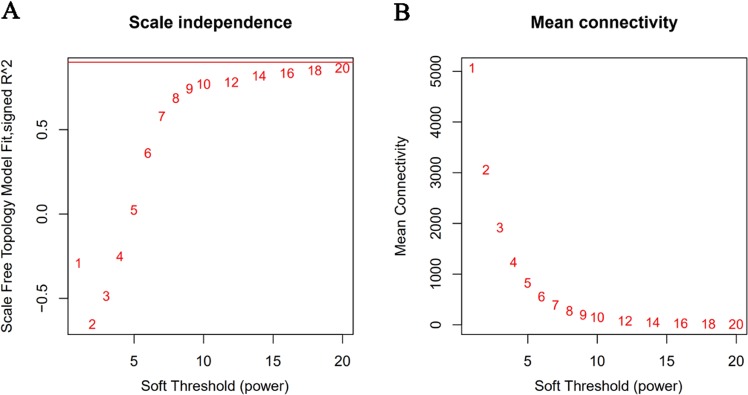
Determination of the soft-thresholding power (β) in weighted gene co-expression network analysis (WGCNA). (**A**) Analysis of the scale-free topology model fitting index (R2, y-axis). (**B**) Mean connectivity for various soft-thresholding powers. The red Arabic numbers in the panels denote different soft thresholds. There is a trade-off between maximizing R2 and maintaining a high mean number of connections. Thus, we set β = 20.

**Figure 3:**
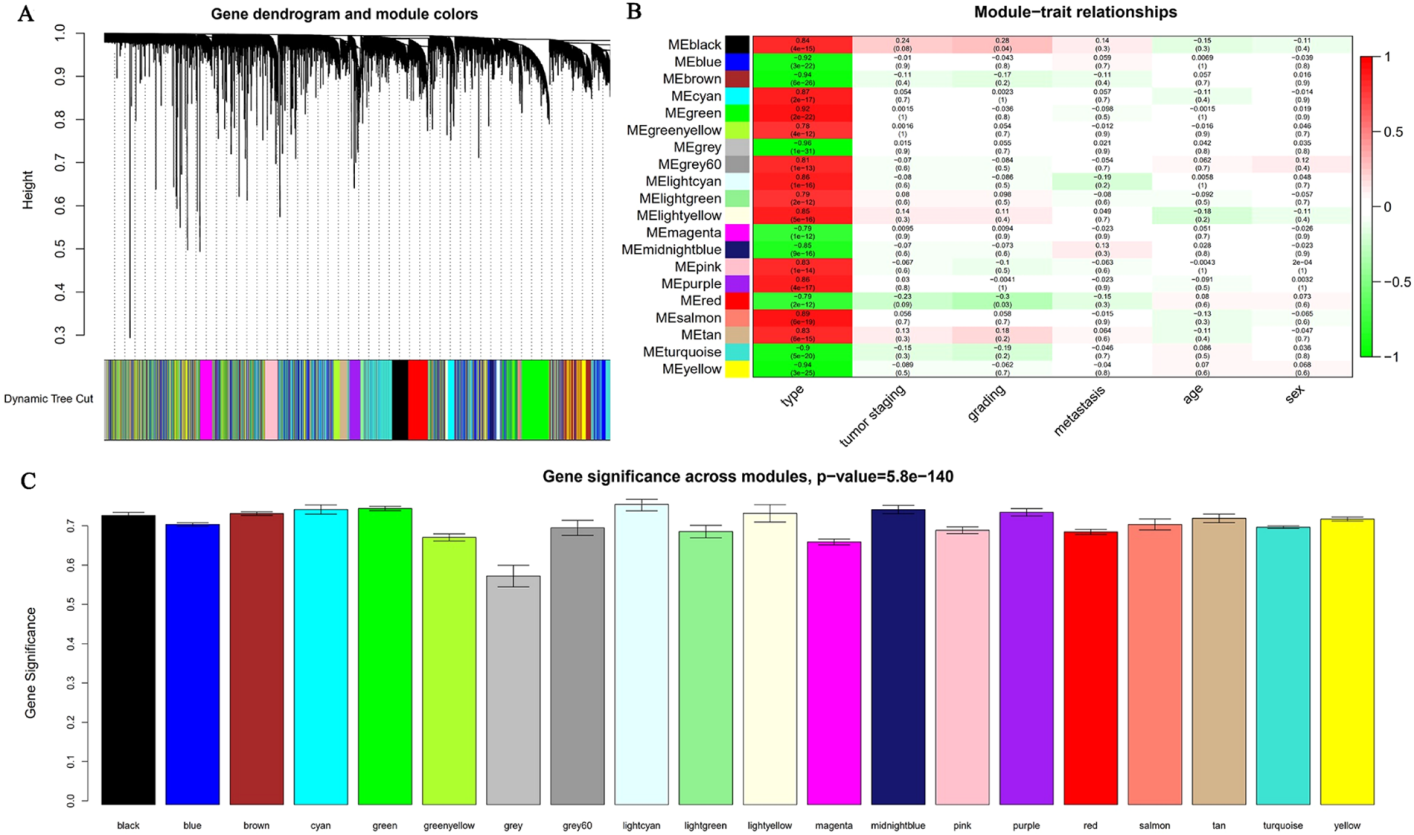
Identification of modules associated with the tumorigenesis of KIRC. (**A**) Dendrogram of all differentially expressed genes clustered based on a dissimilarity measure (1-TOM). (**B**) Heatmap of the correlations between the module eigengenes and clinical traits of KIRC. (**C**) Distribution of average gene significance and errors in the modules associated with the occurrence of KIRC.

Fifty-two samples with clinical data were included in co-expression analysis without significant abnormal values (Fig. 1B). Before the construction of the weighted co-expression network, a weighted parameter of the adjacency function (soft-threshold β = 20) was selected to ensure a scale-free network (Fig. 2). A total of 8763 DEGs were used to construct weighted gene co-expression networks. Twenty co-expressed gene modules were detected by the method of dynamic tree cutting and merging similar modules (Fig. 3A), as can be visualized by a clustering dendrogram. We found that all 20 modules showed a significant correlation with sample type (Fig. 3B) and exhibited high gene significance (Fig. 3C).

### Functional enrichment analysis of the hub genes in WGCNA

**Figure 4:**
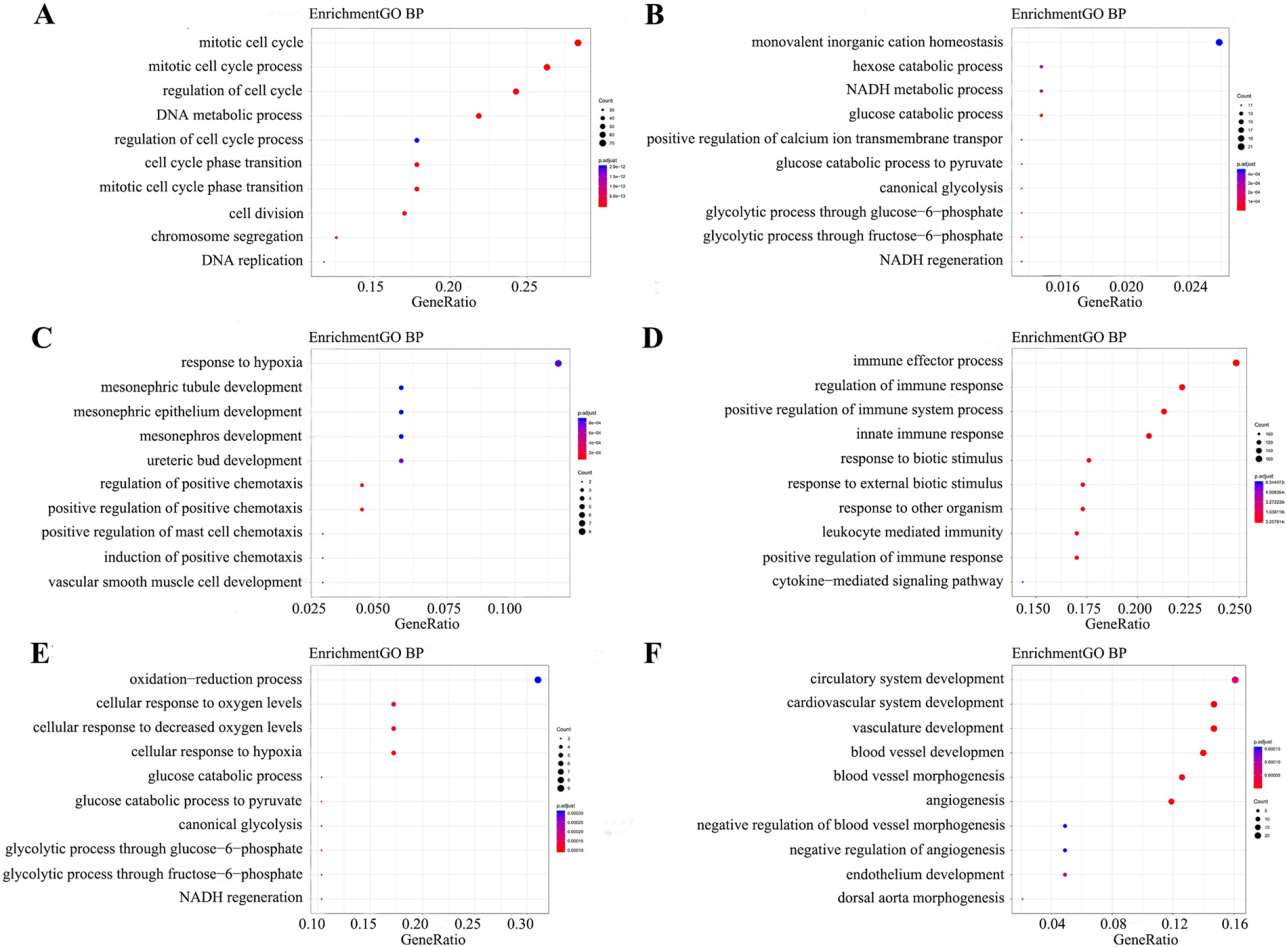
GO enrichment analysis of the genes in modules significantly related to tumorigenesis. GO analysis was carried out on 20 identified modules, among which 6 modules with the highest correlation with tumorigenesis included (**A**) black module, (**B**) brown module, (**C**) cyan module, (**D**) green module, (**E**) lightyellow module, and (**F**) pink module. The size of the bubble indicates the enrichment score, while the colours represent enrichment significance.

**Figure 5:**
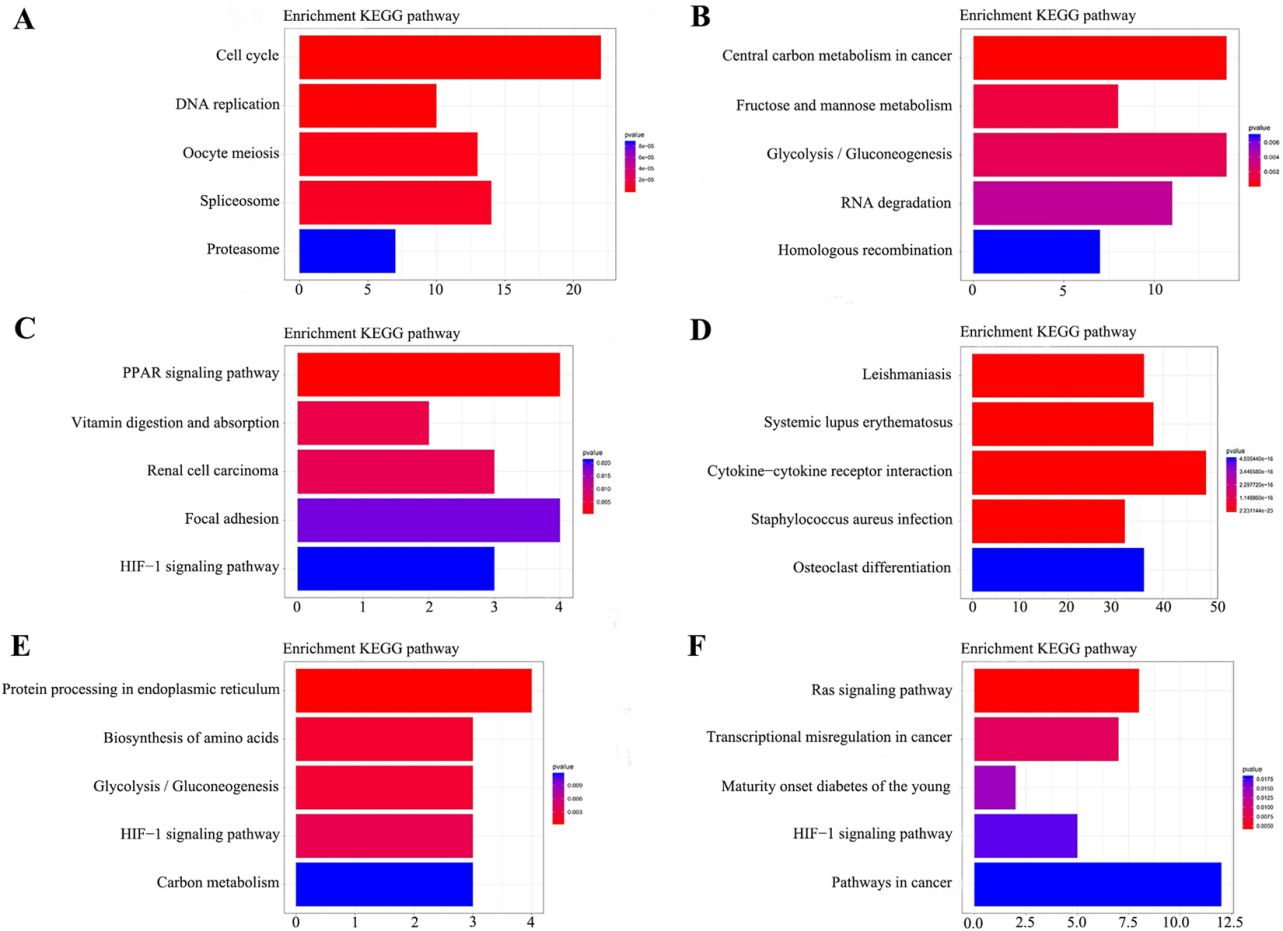
KEGG pathway analysis of the genes in modules significantly related to tumorigenesis. KEGG analysis was carried out on 20 identified modules, among which 6 modules with the highest correlation with tumorigenesis included (**A**) black module, (**B**) brown module, (**C**) cyan module, (**D**) green module, (**E**) lightyellow module, and (**F**) pink module. The length of the column indicates the enrichment score, while the colours represent enrichment significance.

Twenty modules were identified to be significantly associated with the clinical type of the samples. To obtain further insight into the function of the DEGs in KIRC, we performed GO and KEGG pathway enrichment analysis for the identified DEGs in the 20 modules. The results of functional enrichment analysis in each module are depicted in Figs. 4 and 5 by using Gene Ontology and KEGG pathway enrichment analysis. Within the 20 modules, several BPs and pathways that are closely related to the occurrence of KIRC were enriched, including the mitotic cell cycle in the black module (Fig. 4A); monovalent inorganic cation homeostasis (Fig. 4B) and response to hypoxia in the cyan module (Fig. 4C); and immune effector process (Fig. 4D), oxidation-reduction process (Fig. 4E) and circulatory system development (Fig. 4F). The genes were enriched in several signalling pathways, including the cell cycle in the black module (Fig. 5A), central carbon metabolism in cancer in the brown module (Fig. 5B), cytokine-cytokine receptor interaction in the green module (Fig. 5D), Ras signalling pathway in the pink module (Fig. 5E), etc. The HIF-1 signalling pathway was enriched in several modules, including the cyan, pink and lightyellow modules (Fig. 5C,E,F). These significantly enriched GO and KEGG pathway terms enable us to better understand the role of the DEGs in the tumorigenesis and prognosis of KIRC.

### Hub gene identification through WGCNA and PPI

**Figure 6:**
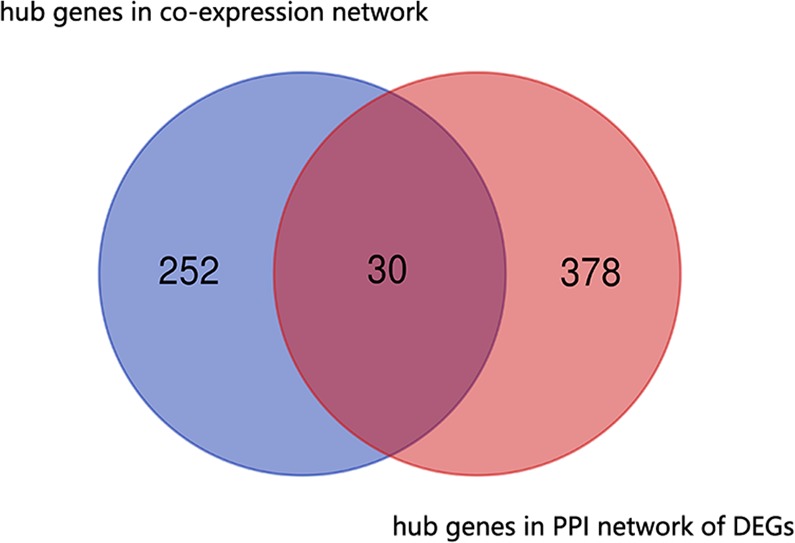
Common hub genes in the co-expression network and PPI network. A Venn diagram was utilized to screen the hub genes between the DEGs and WGCNA. Thirty common network genes were screened as candidates for further analysis and validation.

To identify hub genes, we first constructed a PPI network of the DEGs based on the STRING profile obtained from the STRING analysis tool. A total of 408 genes that reached the cutoff criterion (degree ≥ 10) were regarded as hub genes out of the DEGs. In this study, 20 modules were significantly related to the types of samples. Defined by module connectivity, 282 hub genes in the co-expression network were identified in the 20 modules. As shown in Fig. 6, a total of 30 genes, including PTGER3, IDO1, GDA, ALOX5, SLC12A3, RUNX3, GPC5, SAMHD1, UPB1, CALML3, SELPLG, PKLR, PAG1, GNB4, INPP5D, TLR4, VCAN, SLC22A11, LOX, CCND2, AGXT, KCNJ10, GBP5, BCAT1, TGFB1, NEK2, PRODH2, HCLS1, ANGPT2 and BIRC3, were identified by both WGCNA and PPI network analysis and were then screened out for further validation.

### Hub gene validation

**Figure 7:**
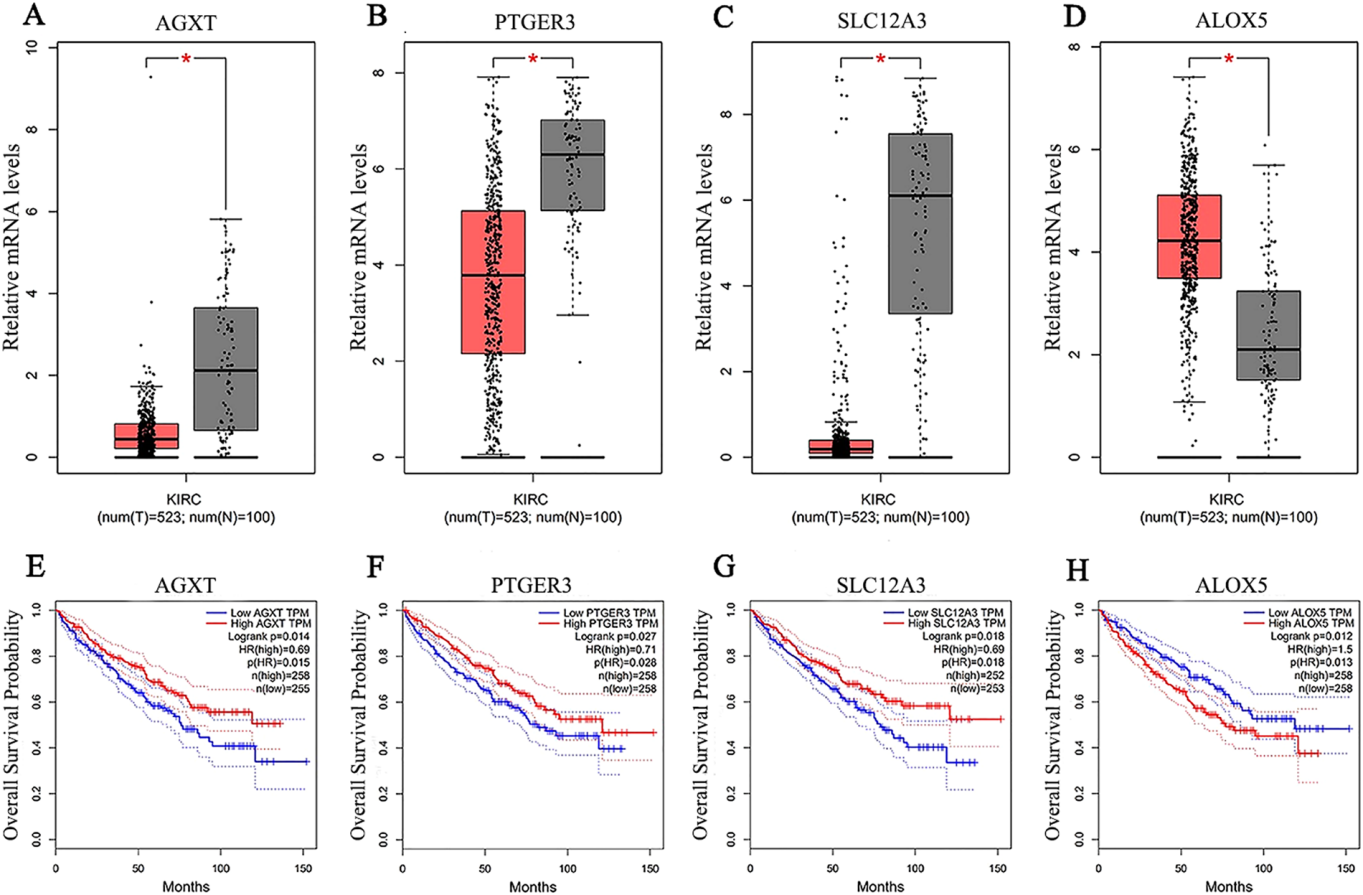
Hub gene validation based on TCGA data in GEPIA. (**A–D**) Gene expression levels between tumours and normal tissues. (**A**) AGXT, (**B**) PTGER3, (**C**) SLC12A3, (**D**) ALOX5. (**E–H**) Survival analysis of the relevance between the overall survival time and the relative expression levels of the hub genes in KIRC. (**E**) AGXT, (**F**) PTGER3, (**G**) SLC12A3, (**H**) ALOX5. The red line represents the samples with high gene expression, and the blue line indicates the samples with low gene expression.

**Figure 8:**
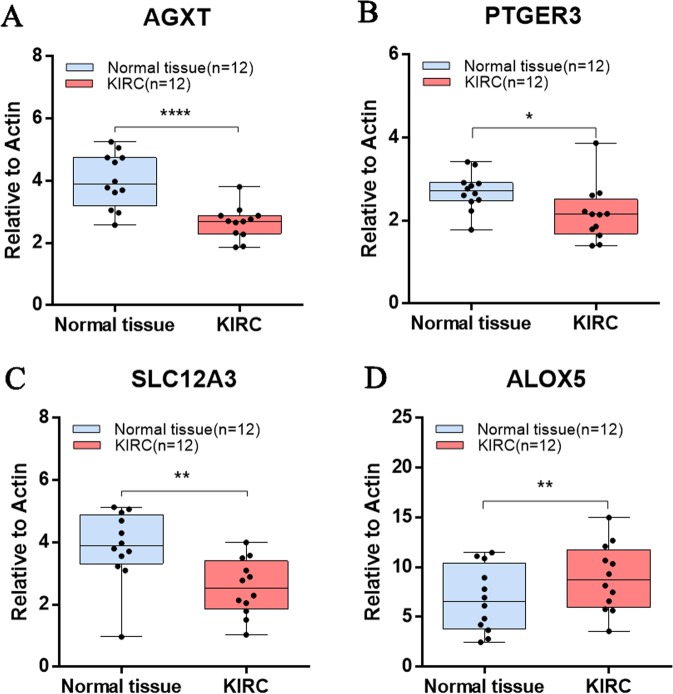
Validation of the hub genes by using RT-qPCR analysis. (**A**) AGXT, (**B**) PTGER3, (**C**) SLC12A3, (**D**) ALOX5. Tumour tissue and paired normal tissue were collected from 12 KIRC patients, and a paired t test was used to evaluate the statistical significance of differences.

Among the 30 candidate hub genes, we then screened out the expression of hub genes that were significantly increased or decreased compared to that in normal tissue in the training dataset by using GEPIA based on the TCGA database. Only four hub gene expression levels were proportional to the overall survival percentage, with *p* values less than 0.05. As shown in Fig. 7, we identified three genes (AGXT, PTGER3 and SLC12A3) with lower mRNA levels in tumour samples (Fig. 7A–C), and patients with a high expression of these three genes exhibited prolonged survival (Fig. 7E–G). In contrast, ALOX5 demonstrated higher mRNA expression levels in tumours (Fig. 7D), and patients with high levels of ALOX5 expression had a low survival rate (Fig. 7H). We then collected 12 KIRC samples along with adjacent normal tissue samples to validate the expression of the key hub genes in KIRC patients. Consistently, we found that ALOX5 was upregulated in the tumour samples, while AGXT, PTGER3, and SLC12A3 were downregulated in the tumour samples (Fig. 8A–D).

## Discussion

Kidney cancers are a common group of chemotherapy-resistant diseases featured by various genetic changes. There are no obvious symptoms in the early stage of kidney cancer, and approximately 30% of patients have metastatic kidney cancer at the time of diagnosis. To discover new biomarkers and predictive models applicable for the early identification of patients who may respond to specific treatments, we integrated data from genome‐wide gene expression datasets to explore the molecular mechanism of KIRC at the systemic level. Through the analysis, we surprisingly found that the expression of innate immune response-associated genes is increased in KIRC, while the expression of genes involved in cellular catabolism is reduced. Further analysis pinpoints 4 key hub genes, AGXT, PTGER3, SLC12A3 and ALOX5, which are closely associated with the progression and prognosis of KIRC. Of importance, some identified DEGs are novel KIRC gene signatures, and their molecular functions in KIRC pathogenesis remain largely unknown.

By functional analysis, we found that a large number of upregulated genes were significantly enriched in the immune response and innate immune response (Supplementary Table S2), while the downregulated genes were enriched in the cellular catabolism of carbon sources (Supplementary Table S3). Of note, the glucose catabolic process and glucose catabolic process to pyruvate were concurrently enriched in two modules (Fig. 4B,C), indicating that glucose metabolism is one of the major targets for KIRC-associated metabolic reprogramming. In addition, the PI3K-Akt signalling pathway, which promotes anabolism and inhibits catabolism^7^, was also markedly enriched in KIRC (Supplementary Table S3). This finding is consistent with the overwhelming demands of cancer cells for biosynthesis and therefore facilitates cell proliferation. Thus, targeting basic metabolic abnormalities in kidney cancer provides a unique opportunity to develop more effective treatments.

In the hub modules, we found that the genes were also enriched in the immune effector process, regulation of immune response, positive regulation of immune system process and innate immune response (Fig. 4D). RCC has been characterized as an immunogenic tumour^8^. Although it is strongly infiltrated by macrophages, T cells, dendritic cells (DCs) and natural killer (NK) cells, RCC generally fails to be eliminated due to the functional impairment of innate immune cells and adaptive immune cells^9^. Although many therapeutic strategies focus on stimulating adaptive immunity, an increasing number of studies have discovered the potential anti-tumour role of innate immunity^10^. Several combination treatments have now been found to have important innate immune stimulatory features that contribute in important ways alongside adaptive immune effectors to controlling tumour progression. New immune checkpoints are emerging with key roles in regulating both the T cell response and innate immune response^11,12^. Combined treatment with the immune checkpoint inhibitors nivolumab plus ipilimumab has a significant effect on the overall survival rate of patients with intermediate- and high-risk advanced renal cancer, with an overall survival rate of 75% at 18 months^13^. However, the use of strategies activating different components of the innate immune system is still in its infancy but might find a place in clinical oncology.

Among these real hub genes, AGXT was mainly enriched in the biosynthesis of antibiotics, carbon metabolism, glycine, serine and threonine metabolism and peroxisomes in the KEGG pathway analysis (Supplementary Table S3). In particular, AGXT-encoded proteins are mainly located in peroxisomes and participate in β-oxidation^14^. Downregulated peroxisome pathways may result in oxidation-reduction processes and accumulated fatty acids (Fig. 4E and Supplementary Table S2). Prostaglandin E2 (PGE2) can trigger mast cell activation, which can inhibit tumours by releasing IL-6 and TNF-α^15^ in a mechanism involving PTGER3, which plays an important role in suppression of cell growth, and its downregulation enhances colon carcinogenesis at a later stage and may oppose the pro-tumorigenic effects of PGE2 elevation and COX-2 overexpression in breast cancer^16,17^. SLC12A3 is significantly enriched in the transport term in GO biological processes (Supplementary Table S2). Activating SLC to increase the transport of chemotherapeutic drugs may become a new strategy for cancer treatment^18^. ALOX5 plays a crucial role in regulating the interaction between innate and adaptive immunity^19,20^. M. Faronato’s study showed that ALOX5 protein levels were significantly increased in the majority of KIRCs (*p* < 0.001)^21^. In addition, their results suggest that ALOX5 pathway contributes to constitutive VEGF gene expression in KIRCs that have lost VHL function.

In conclusion, with the application of high-throughput sequencing, we now have a better understanding of the molecular hallmarks of cancer. Our study classifies the importance of some pathways related to KIRC, such as oncogenic metabolism and immune response. In addition, we used different strategies to identify new candidate genes, which may become a new target for cancer therapy in the future. However, this study still has some limitations. The mechanisms underlying how these genes promote or inhibit cancer development remain unclear. In addition, the relationship between these four genes and tumour metastasis is also worth studying. Further molecular biological experiments are needed to determine the mechanism of how these genes work.

## Methods

### Data collection

The mRNA expression profile and related clinical data of human KIRC were downloaded from the Gene Expression Omnibus database. The dataset GSE66272, performed on an Affymetrix Human Genome U133 Plus 2.0 Array was used to construct co-expression networks and identify hub genes in our study. This dataset included 26 primary KIRC and 26 adjacent normal tissue samples, and each pair was from the same patient. According to the description, sample ID GSM1618417 represents a patient with sarcoma instead of KIRC. That sample does not fit the scope of our investigation on KIRC. Consequently, we removed this sample and its adjacent normal tissue sample (GSM1618418) while we performed the subsequent analysis with data from GSE66272. The gene expression data were based on RNA-sequencing technology.

### Identification of differentially expressed genes (DEGs)

We downloaded raw mRNA expression data from KIRC patients from the Gene Expression Omnibus database. Genes differentially expressed between primary KIRC and adjacent normal tissues were identified through the “limma” R package^22^. Here, the Benjamin and Hochberg method was used to perform multiple testing corrections of the raw *p* values to achieve the false discovery rate (FDR)^23^. We set DEG thresholds with FDR less than 0.05 and |log FC| greater than 0.349. The DEGs between KIRC samples and adjacent normal tissue samples were all screened.

### Constructing the gene co-expression network

Co-expression measurements with WGCNA were converted into connection weights or topology overlap measurements to explore the interactions between the identified DEGs^24^. Co-expression methodology is typically conducted to explore correlations between gene expression levels. Genes involved in the same functional compound tend to exhibit similar expression patterns^25^. In this study, we inputted all identified DEGs to construct weighted co-expression modules by using the WGCNA package in R^26^. The co-expression module threshold was set as *p* < 0.05.

### Identification of clinically significant modules

Two approaches were used to identify modules related to the clinical traits of KIRC. First, we defined gene significance (GS) as the log10 transformation of the *p* value (GS = lgP) in a linear regression between the gene expression and clinical traits. Then, we defined module significance (MS) as the average GS for all genes in a module^27^. In general, the module with the absolute MS ranked first or second among all the selected modules was considered the one related to a clinical trait. MEs were considered the major component in principal component analysis for each gene module, and the expression patterns of all genes were summarized into a single characteristic expression profile. In addition, we calculated the correlation between the MEs and clinical traits to identify relevant modules. The module with the maximal absolute MS among all selected modules was usually considered related to a clinical trait. Finally, modules highly correlated with certain clinical traits were selected for further analysis.

### Identification of hub genes

The hub genes of the modules have more biological significance in disease association than the hub genes of global networks^28^. In this study, we considered a gene as a hub gene if it has a unique characteristic, e.g., high module membership (MM), high gene significance (GS), or high intramodular connectivity (IC) in the network^6^. GS shows different ICs, which represent the connectivity within the genes of the network. MM indicates the significance of genes in various networks. We therefore identified the hub genes in the modules through MM, GS and IC.

### Protein-protein interaction (PPI)

We uploaded all identified DEGs to the Search Tool for the Retrieval of Interacting Genes/Proteins (STRING) database to construct a PPI network^29^. The degree of each gene was calculated by Network Analyzer (a tool in Cytoscape software 3.6.0 (https://cytoscape.org/)). Then, genes with a degree greater than or equal to 10 were defined as hub genes^30^.

### Function enrichment analyses

DAVID^31^ (https://david.ncifcrf.gov/), a common functional annotation tool for bioinformatics resources, was utilized to distinguish the biological attributes. A *p* value of less than 0.05 was used as the cutoff criterion. For the identified DEGs in the 20 modules, we performed GO and KEGG pathway enrichment analysis by using the R package “cluster Profiler”^32^. Gene sets with a *p* value of less than 0.05 were considered significantly enriched.

### Patients and samples

The institutional review board approved the study. All methods were performed in accordance with the relevant guidelines and regulations. Written informed consent was obtained before clinical sample collection. KIRC and adjacent normal tissues were collected from 12 patients. The histology of all tumour samples was centrally reviewed by a pathologist. At the time point of sample collection, none of the patients had received therapeutic medications or previous surgical interventions.

### Real-time quantitative PCR

Total RNA from the frozen tumour samples of the KIRC patients was extracted by using the RNeasy Mini Kit (QIAGEN, Hilden, Germany) according to the manual instructions. We performed RT-qPCR by using SYBR Green reaction mixture in a 7900HT Fast Real-Time PCR System (Applied Biosystems, Foster City, CA, USA). The primer sequences used for amplification are detailed in Supplementary Table S1.

### Statistical analysis

In this study, we used a paired *t* test to examine the differences in gene expression between tumour and normal tissues. A *p* value of less than 0.05 was considered statistically significant (*, ** and **** are used to indicate statistical significance corresponding to a *P*-value < 0.05, *P*-value < 0.01 and *P*-value < 0.0001, respectively). Error bars denote the S.E.M. We used R and GraphPad Prism version 7.0 (GraphPad Software, San Diego, CA, USA) to perform statistical analysis.

### Ethics approval

This study was approved by Ethics Committee of Xuzhou Medical College Affiliated Hospital.

## Supplementary information

Supplementary information.
