# Supplementary material for: Identification of the key genes and pathways involved in the tumorigenesis and prognosis of kidney renal clear cell carcinoma
Source: Sci Rep. 2020 Mar 6;10:4271. doi: 10.1038/s41598-020-61162-4 (PMC7060270; doi:10.1038/s41598-020-61162-4)
Supplement: Supplementary file 1 — Supplementary information. [file 41598_2020_61162_MOESM1_ESM.pdf]

Identification of the key genes and pathways involved in the tumorigenesis and prognosis of kidney renal clear cell carcinoma

Hao Cui[1,2], Hongjian Shan[3], Michael Zhe Miao[4], Zhiguo Jiang[1], Yuanyuan Meng[1], Ran Chen[5], Longzhen Zhang[1,2], Yong Liu[1,6]

[1] Cancer Institute, Xuzhou Medical University, Xuzhou, Jiangsu, 221000, China
[2] Department of Radiotherapy, Xuzhou Medical University Affiliated Hospital, Xuzhou, Jiangsu, 221000, China
[3] Department of Orthopaedics, Xuzhou Medical University Affiliated Hospital, Xuzhou, Jiangsu, 221000, China
[4] Department of Oral and Craniofacial Health Sciences, Adams School of Dentistry, University of North Carolina at Chapel Hill, Chapel Hill, NC, 27516, USA
[5] Department of Cardiology, Affiliated Hospital of Nanjing University of Chinese Medicine, Nanjing, Jiangsu, 210029, China
[6] Center of Clinical Oncology, Xuzhou Medical University Affiliated Hospital, Xuzhou, Jiangsu, 221000, China

Corresponding Authors' E-mail addresses:
Yong Liu: liuymito@xzhmu.edu.cn
Longzhen Zhang: zlzxzhmu@gmail.com

Hao Cui and Hongjian Shan should be regard as co-first authors.

Supplemental Table S1. Primers used in RT-qPCR

Gene		Primer
Actin	forward	CCTAAGGCCAACCGTGAAAA
	reverse	AGGCATACAGGGACAGCACA
AGXT	forward	CCAGGATGTACCATCACACAATC
	reverse	GTAGCTGACGATGTCTCTCC
PTGER3	forward	TCAATCAGACATCAGTTGAGCAC
	reverse	CTGGATGCATAGTTGTTTGTGTG
SLC12A3	forward	GAAGACCACATCAAGAACTACCGC
	reverse	GTAGAAGGCCTTGATCTTCCTCTTG
ALOX5	forward	TGGAATGACTTCGCCGACTTTGAG
	reverse	TAGCCAAACATCAGGTCTTCCTGC

Supplemental Table S2. GO enrichment analysis of DEGs in KIRC.

Expression	Category	Term	Count	%	P Value
Up-regulation	GOTERM_BP_DIRECT	GO:0007165~signal transduction	181	7.4	9.47E-14
	GOTERM_BP_DIRECT	GO:0006955~immune response	110	4.5	4.70E-25
	GOTERM_BP_DIRECT	GO:0006915~apoptotic process	104	4.2	2.81E-12
	GOTERM_BP_DIRECT	GO:0045087~innate immune response	96	3.9	5.51E-17
	GOTERM_BP_DIRECT	GO:0006954~inflammatory response	93	3.8	2.73E-19
	GOTERM_CC_DIRECT	GO:0005737~cytoplasm	539	22.0	5.58E-06
	GOTERM_CC_DIRECT	GO:0005886~plasma membrane	513	21.0	9.48E-20
	GOTERM_CC_DIRECT	GO:0016021~integral component of membrane	512	20.9	7.58E-04
	GOTERM_CC_DIRECT	GO:0005634~nucleus	509	20.8	0.043736
	GOTERM_CC_DIRECT	GO:0005829~cytosol	407	16.6	8.66E-14
	GOTERM_MF_DIRECT	GO:0005515~protein binding	965	39.5	1.69E-22
	GOTERM_MF_DIRECT	GO:0005524~ATP binding	200	8.1	3.20E-09
	GOTERM_MF_DIRECT	GO:0042802~identical protein binding	117	4.7	2.55E-09
	GOTERM_MF_DIRECT	GO:0042803~protein homodimerization activity	108	4.4	1.96E-07
	GOTERM_MF_DIRECT	GO:0005509~calcium ion binding	97	3.9	3.90E-05
Down-regulation	GOTERM_BP_DIRECT	GO:0055114~oxidation-reduction process	105	4.3	7.41E-13
	GOTERM_BP_DIRECT	GO:0006810~transport	58	2.4	1.35E-06
	GOTERM_BP_DIRECT	GO:0055085~transmembrane transport	46	1.9	6.52E-07
	GOTERM_BP_DIRECT	GO:0035556~intracellular signal transduction	46	1.9	0.039590
	GOTERM_BP_DIRECT	GO:0008152~metabolic process	41	1.7	1.81E-09
	GOTERM_CC_DIRECT	GO:0016021~integral component of membrane	491	20	9.98E-04
	GOTERM_CC_DIRECT	GO:0070062~extracellular exosome	422	17	1.51E-36
	GOTERM_CC_DIRECT	GO:0005886~plasma membrane	412	17	5.19E-05
	GOTERM_CC_DIRECT	GO:0005739~mitochondrion	187	7.7	1.96E-12
	GOTERM_CC_DIRECT	GO:0005887~integral component of plasma membrane	171	7.0	1.18E-06
	GOTERM_MF_DIRECT	GO:0042803~protein homodimerization activity	93	3.8	7.93E-05
	GOTERM_MF_DIRECT	GO:0016491~oxidoreductase activity	46	1.9	1.06E-09
	GOTERM_MF_DIRECT	GO:0005102~receptor binding	46	1.9	0.004045
	GOTERM_MF_DIRECT	GO:0003824~catalytic activity	41	1.7	4.64E-08
	GOTERM_MF_DIRECT	GO:0005215~transporter activity	37	1.5	1.63E-05

Supplemental Table S3. KEGG pathway analysis of DEGs in KIRC.

Expression	Pathway ID	Name	Count	%	P Value
Up-regulation	hsa05200	Pathways in cancer	74	3.0	4.35E-06
	hsa04151	PI3K-Akt signaling pathway	71	2.9	2.35E-07
	hsa05166	HTLV-I infection	67	2.7	1.14E-11
	hsa05203	Viral carcinogenesis	58	2.3	1.69E-11
	hsa04060	Cytokine-cytokine receptor interaction	58	2.3	1.88E-08
	hsa04145	Phagosome	56	2.2	6.53E-17
	hsa05152	Tuberculosis	54	2.2	3.91E-12
	hsa05168	Herpes simplex infection	48	1.9	1.77E-08
	hsa05322	Systemic lupus erythematosus	47	1.9	4.88E-13
	hsa05164	Influenza A	44	1.9	2.37E-07
Down-regulation	hsa01100	Metabolic pathways	220	9.1	3.79E-26
	hsa00280	Biosynthesis of antibiotics	58	2.4	5.56E-14
	hsa01130	Carbon metabolism	36	1.4	6.83E-11
	hsa00260	Valine, leucine and isoleucine degradation	27	1.1	2.06E-15
	hsa01200	Glycine, serine and threonine metabolism	21	0.9	2.52E-11
	hsa00071	Peroxisome	21	0.9	5.66E-05
	hsa00640	Fatty acid degradation	20	0.8	1.25E-09
	hsa00620	Protein digestion and absorption	20	0.8	4.00E-04
	hsa00650	PPAR signaling pathway	19	0.8	2.79E-05
	hsa00630	Tight junction	19	0.8	9.63E-04

Supplemental Table S4. DEGs in GSE66272.

	ID	logFC	adj.P.Va	Gene.Symbol
1	218484_at	-7.184551852	7.25E-13	NDUFA4L2
2	204416_x_at	-5.939425926	5.17E-19	APOC1
3	206025_s_at	-5.803781481	7.25E-16	TNFAIP6
4	213479_at	-5.726459259	3.11E-11	NPTX2
5	223333_s_at	-5.656514815	2.34E-13	ANGPTL4
6	202934_at	-5.417133333	3.26E-19	HK2
7	206026_s_at	-5.371125926	6.54E-16	TNFAIP6
8	217078_s_at	-5.3649	2.72E-21	CD300A
9	205029_s_at	-5.333937037	3.00E-06	FABP7
10	1554195_a_at	-5.143481481	4.05E-10	C5orf46
11	216834_at	-5.088840741	7.15E-16	RGS1
12	242517_at	-4.915259259	1.49E-12	KISS1R
13	202238_s_at	-4.841788889	2.83E-19	LOC101928916 /// NNMT
14	221870_at	-4.788614815	1.35E-18	EHD2
15	201291_s_at	-4.739166667	2.72E-12	TOP2A
16	205030_at	-4.709337037	1.02E-06	FABP7
17	210445_at	-4.679833333	4.57E-10	FABP6
18	205199_at	-4.654733333	1.09E-11	CA9
19	225681_at	-4.629518519	3.86E-15	CTHRC1
20	231007_at	-4.519422222	1.25E-10	
21	219725_at	-4.513851852	5.75E-18	TREM2
22	201313_at	-4.498803704	8.83E-18	ENO2
23	202237_at	-4.407896296	3.26E-19	LOC101928916 /// NNMT
24	202988_s_at	-4.381607407	1.36E-13	RGS1
25	221755_at	-4.328737037	7.20E-20	EHBP1L1
26	236180_at	-4.320944444	9.37E-12	
27	1554452_a_at	-4.272151852	8.19E-15	HILPDA
28	211571_s_at	-4.241659259	2.20E-13	VCAN
29	226661_at	-4.229755556	5.55E-11	CDCA2
30	213915_at	-4.212751852	3.21E-14	NKG7
31	218507_at	-4.197859259	8.90E-16	HILPDA
32	218308_at	-4.190003704	1.22E-19	TACC3
33	204698_at	-4.178740741	1.29E-15	ISG20
34	201506_at	-4.169248148	1.22E-16	TGFBI
35	32128_at	-4.168003704	2.35E-10	CCL18
36	229390_at	-4.161659259	1.03E-17	FAM26F
37	219918_s_at	-4.158703704	1.90E-13	ASPM
38	222838_at	-4.147718519	2.76E-13	SLAMF7
39	202855_s_at	-4.097496296	3.59E-15	MIR6787 /// SLC16A3
40	211192_s_at	-4.081881481	1.48E-15	CD84

41	215646_s_at	-4.07377037	1.94E-13	VCAN
42	1558034_s_at	-4.031674074	1.69E-07	CP
43	221530_s_at	-4.024648148	2.63E-18	BHLHE41
44	221009_s_at	-4.023262963	6.02E-11	ANGPTL4
45	220832_at	-4.020225926	1.16E-16	TLR8
46	222847_s_at	-4.003988889	8.98E-11	EGLN3
47	202628_s_at	-3.996662963	1.48E-10	SERPINE1
48	242943_at	-3.971003704	5.77E-16	ST8SIA4
49	211122_s_at	-3.962696296	2.19E-11	CXCL11
50	207419_s_at	-3.924766667	8.27E-14	RAC2
51	206914_at	-3.893107407	5.51E-13	CRTAM
52	212143_s_at	-3.888637037	4.15E-15	IGFBP3
53	201292_at	-3.88347037	5.85E-13	TOP2A
54	209683_at	-3.8729	7.28E-15	FAM49A
55	215446_s_at	-3.855940741	2.97E-13	LOX
56	211756_at	-3.853674074	3.70E-09	PTHLH
57	218585_s_at	-3.85252963	3.94E-15	DTL
58	219990_at	-3.824892593	3.13E-11	E2F8
59	223185_s_at	-3.821403704	3.10E-14	BHLHE41
60	210992_x_at	-3.805348148	4.09E-16	FCGR2C
61	1405_i_at	-3.786251852	2.23E-14	CCL5
62	228054_at	-3.780825926	1.96E-16	TMEM44
63	204846_at	-3.765818519	1.47E-06	CP
64	1555214_a_at	-3.760425926	2.30E-10	CLEC7A
65	202345_s_at	-3.748007407	3.70E-12	FABP5
66	213553_x_at	-3.736922222	7.62E-14	APOC1
67	204014_at	-3.729733333	1.25E-14	DUSP4
68	222608_s_at	-3.718322222	6.90E-10	ANLN
69	219410_at	-3.710344444	6.55E-13	TMEM45A
70	230836_at	-3.698540741	2.00E-17	ST8SIA4
71	207104_x_at	-3.698037037	1.05E-16	LILRB1
72	209652_s_at	-3.691337037	2.07E-08	PGF
73	207091_at	-3.688540741	2.93E-17	P2RX7
74	204006_s_at	-3.679666667	4.45E-19	FCGR3A /// FCGR3B
75	209924_at	-3.678851852	1.05E-08	CCL18
76	219249_s_at	-3.669048148	2.86E-11	FKBP10
77	244044_at	-3.653433333	1.71E-08	
78	209859_at	-3.653348148	4.23E-12	TRIM9
79	217028_at	-3.647351852	2.44E-19	CXCR4
80	205686_s_at	-3.640344444	9.61E-15	CD86
81	240058_at	-3.631292593	1.14E-06	
82	218888_s_at	-3.622433333	8.88E-11	NETO2
83	202998_s_at	-3.621922222	2.36E-16	LOXL2

84	225353_s_at	-3.607759259	9.13E-18	C1QC
85	211708_s_at	-3.606814815	7.34E-12	SCD
86	204298_s_at	-3.598748148	3.90E-11	LOX
87	212992_at	-3.597596296	8.52E-17	AHNAK2
88	227253_at	-3.581966667	4.24E-07	CP
89	204192_at	-3.569218519	3.01E-14	CD37
90	205572_at	-3.56907037	6.55E-10	ANGPT2
91	218542_at	-3.561481481	1.42E-10	CEP55
92	218755_at	-3.554148148	1.46E-11	KIF20A
93	223280_x_at	-3.54557037	3.05E-17	MS4A6A
94	202095_s_at	-3.535111111	2.66E-11	BIRC5
95	201720_s_at	-3.534814815	3.26E-19	LAPTM5
96	1555756_a_at	-3.531740741	2.54E-11	CLEC7A
97	1553043_a_at	-3.530644444	2.24E-14	CD300LF
98	204825_at	-3.521522222	5.82E-13	MELK
99	227645_at	-3.521388889	2.17E-16	PIK3R5
100	222774_s_at	-3.514274074	7.33E-12	NETO2
101	1555533_at	-3.506707407	8.22E-10	QRFPR
102	206508_at	-3.500892593	1.54E-11	CD70
103	225655_at	-3.499311111	3.41E-11	UHRF1
104	220146_at	-3.498751852	9.32E-14	TLR7
105	229168_at	-3.485751852	4.87E-09	COL23A1
106	218145_at	-3.481359259	1.39E-15	TRIB3
107	1555788_a_at	-3.475722222	5.89E-12	TRIB3
108	203065_s_at	-3.464948148	3.94E-15	CAV1
109	219574_at	-3.462007407	3.63E-14	1-Mar
110	1555745_a_at	-3.460881481	3.55E-08	LYZ
111	204162_at	-3.459381481	1.55E-11	NDC80
112	1556209_at	-3.459111111	1.71E-09	CLEC2B
113	213160_at	-3.454251852	4.04E-13	DOCK2
114	206836_at	-3.447418519	3.99E-08	SLC6A3
115	206219_s_at	-3.429474074	5.11E-14	VAV1
116	211887_x_at	-3.424403704	1.21E-12	MSR1
117	202404_s_at	-3.422681481	3.66E-11	COL1A2
118	230422_at	-3.420518519	9.30E-16	FPR3
119	219191_s_at	-3.418303704	9.28E-18	BIN2
120	209642_at	-3.411188889	1.93E-09	BUB1
121	202856_s_at	-3.403481481	1.10E-16	MIR6787 /// SLC16A3
122	221731_x_at	-3.401744444	2.75E-13	VCAN
123	219888_at	-3.397781481	4.11E-14	SPAG4
124	210895_s_at	-3.396018519	8.52E-17	CD86
125	206420_at	-3.392874074	2.09E-14	IGSF6
126	203331_s_at	-3.389007407	8.63E-14	INPP5D

127	218741_at	-3.385955556	2.11E-12	CENPM
128	210029_at	-3.382211111	6.55E-13	IDO1
129	205242_at	-3.375651852	4.70E-07	CXCL13
130	202627_s_at	-3.372659259	1.83E-10	SERPINE1
131	233510_s_at	-3.3722	1.94E-13	PARVG
132	211148_s_at	-3.370696296	1.20E-10	ANGPT2
133	224356_x_at	-3.36987037	5.28E-17	MS4A6A
134	204620_s_at	-3.369718519	8.88E-14	VCAN
135	231559_at	-3.365551852	1.81E-13	NNMT
136	1555229_a_at	-3.363803704	2.55E-09	C1S
137	208071_s_at	-3.361507407	1.77E-16	LAIR1
138	236957_at	-3.359074074	2.11E-12	CDCA2
139	212097_at	-3.357803704	6.54E-18	CAV1
140	202953_at	-3.355	3.05E-16	C1QB
141	206134_at	-3.350792593	3.06E-08	ADAMDEC1
142	207677_s_at	-3.349711111	2.60E-12	NCF4
143	212998_x_at	-3.34807037	2.07E-16	HLA-DQB1 /// LOC101060835
144	217984_at	-3.345633333	1.52E-17	RNASET2
145	200832_s_at	-3.332318519	2.09E-14	SCD
146	210095_s_at	-3.32222963	1.29E-15	IGFBP3
147	221724_s_at	-3.321325926	2.20E-12	CLEC4A
148	228273_at	-3.320940741	2.73E-15	PRR11
149	224358_s_at	-3.319207407	1.00E-11	MS4A7
150	1554899_s_at	-3.315192593	1.06E-17	FCER1G
151	203915_at	-3.306096296	4.99E-13	CXCL9
152	214228_x_at	-3.30497037	1.08E-16	TNFRSF4
153	209933_s_at	-3.304725926	1.40E-14	CD300A
154	205523_at	-3.30397037	4.16E-08	HAPLN1
155	214366_s_at	-3.287196296	4.45E-10	ALOX5
156	204982_at	-3.269714815	3.10E-14	GIT2
157	201890_at	-3.268018519	5.30E-14	RRM2
158	204619_s_at	-3.267318519	1.86E-12	VCAN
159	1552619_a_at	-3.2647	1.11E-10	ANLN
160	229391_s_at	-3.260674074	4.18E-16	FAM26F
161	1553392_at	-3.259381481	1.28E-07	EFCAB3 /// LOC102724660
162	217767_at	-3.256237037	7.19E-11	C3
163	204962_s_at	-3.247611111	4.91E-13	CENPA /// SLC35F6
164	206488_s_at	-3.244548148	2.81E-08	CD36
165	1552691_at	-3.243907407	1.11E-11	ARL11
166	207085_x_at	-3.238807407	1.14E-14	CSF2RA
167	210984_x_at	-3.236333333	2.87E-09	EGFR
168	202112_at	-3.233081481	1.67E-10	VWF
169	232694_at	-3.224062963	3.88E-09	ZNF395

170	206686_at	-3.222944444	2.34E-10	PDK1
171	218232_at	-3.222592593	4.50E-16	C1QA
172	204900_x_at	-3.219988889	3.75E-18	SAP30
173	204533_at	-3.215948148	6.40E-15	CXCL10
174	224252_s_at	-3.20297037	1.72E-14	FXYD5
175	231513_at	-3.199388889	1.49E-10	
176	214567_s_at	-3.195933333	1.31E-10	XCL1 /// XCL2
177	202503_s_at	-3.184292593	7.00E-15	KIAA0101
178	206666_at	-3.182488889	7.62E-10	GZMK
179	91703_at	-3.173640741	2.41E-14	EHBP1L1
180	223344_s_at	-3.168466667	1.09E-14	MS4A7
181	209474_s_at	-3.166222222	7.76E-12	ENTPD1
182	212464_s_at	-3.165411111	2.10E-16	FN1
183	217590_s_at	-3.162374074	1.71E-09	TRPA1
184	1555759_a_at	-3.161885185	9.13E-12	CCL5
185	230261_at	-3.160140741	1.36E-14	ST8SIA4
186	211607_x_at	-3.146455556	1.36E-11	EGFR
187	241118_at	-3.141096296	1.34E-07	LINC00462
188	213566_at	-3.140818519	4.76E-15	RNASE6
189	204446_s_at	-3.138385185	4.03E-14	ALOX5
190	1558290_a_at	-3.138103704	1.34E-18	MIR1204 /// PVT1
191	206991_s_at	-3.135496296	1.48E-14	CCR5
192	217983_s_at	-3.125437037	9.93E-16	RNASET2
193	204641_at	-3.117644444	5.78E-11	NEK2
194	224102_at	-3.116074074	5.33E-08	P2RY12
195	237737_at	-3.111451852	9.37E-07	ANKRD20A12P /// LOC101059949 /// LOC101060632 /// LOC101927345
196	243366_s_at	-3.098107407	3.16E-12	
197	204198_s_at	-3.097377778	2.07E-11	RUNX3
198	202207_at	-3.097340741	2.96E-13	ARL4C
199	210052_s_at	-3.090011111	8.55E-13	TPX2
200	214511_x_at	-3.088388889	4.32E-15	FCGR1B
201	204655_at	-3.085633333	1.34E-12	CCL5
202	206298_at	-3.084422222	1.89E-11	ARHGAP22
203	204122_at	-3.068066667	5.63E-18	TYROBP
204	202902_s_at	-3.066151852	1.92E-16	CTSS
205	218839_at	-3.052759259	6.29E-12	HEY1
206	1555349_a_at	-3.052381481	8.73E-17	ITGB2
207	219666_at	-3.045174074	1.47E-16	MS4A6A
208	209083_at	-3.041555556	5.02E-15	CORO1A
209	203409_at	-3.032725926	2.68E-18	DDB2
210	207697_x_at	-3.032425926	1.06E-13	LILRB2
211	216442_x_at	-3.030433333	5.04E-15	FN1

212	232278_s_at	-3.029792593	8.87E-08	DEPDC1
213	232231_at	-3.029722222	1.02E-13	RUNX2
214	203324_s_at	-3.0296	6.54E-18	CAV2
215	203820_s_at	-3.02682963	4.06E-06	IGF2BP3
216	1555907_at	-3.02272963	4.68E-09	AGAP2-AS1
217	229490_s_at	-3.01162963	2.89E-10	
218	201818_at	-3.01112963	9.32E-16	LPCAT1
219	213640_s_at	-3.008577778	5.84E-08	LOX
220	216950_s_at	-3.001577778	4.89E-16	FCGR1A /// FCGR1B /// FCGR1C
221	211719_x_at	-3.001440741	4.17E-15	FN1
222	228776_at	-2.990803704	1.20E-14	GJC1
223	220066_at	-2.989892593	1.86E-12	NOD2
224	1555728_a_at	-2.982888889	4.25E-14	MS4A4A
225	236034_at	-2.981955556	4.93E-10	ANGPT2
226	201037_at	-2.98102963	6.62E-17	PFKP
227	209823_x_at	-2.979444444	3.43E-11	HLA-DQB1 /// LOC101060835
228	206503_x_at	-2.974062963	6.12E-15	PML
229	218726_at	-2.97082963	3.30E-08	HJURP
230	204882_at	-2.970611111	3.60E-11	ARHGAP25
231	211796_s_at	-2.966218519	9.50E-12	TRBC1
232	207414_s_at	-2.964337037	1.91E-09	LOC100507472 /// PCSK6
233	206925_at	-2.956340741	2.30E-11	ST8SIA4
234	209671_x_at	-2.955307407	2.94E-08	TRAC
235	219607_s_at	-2.948344444	5.83E-13	MS4A4A
236	1553423_a_at	-2.948214815	8.68E-10	SLFN13
237	209773_s_at	-2.94477037	6.15E-12	RRM2
238	214282_at	-2.942940741	3.12E-07	
239	229937_x_at	-2.942655556	3.97E-17	
240	214770_at	-2.941088889	6.54E-18	MSR1
241	230741_at	-2.936348148	6.10E-18	P2RX7
242	215813_s_at	-2.930881481	7.59E-11	PTGS1
243	1552256_a_at	-2.929822222	1.99E-12	SCARB1
244	204961_s_at	-2.928977778	7.64E-15	NCF1 /// NCF1B /// NCF1C
245	204951_at	-2.924840741	1.13E-12	RHOH
246	233986_s_at	-2.924425926	3.54E-13	PLEKHG2
247	221520_s_at	-2.922451852	2.19E-09	CDCA8
248	212671_s_at	-2.921722222	1.62E-14	HLA-DQA1 /// HLA-DQA2 /// LOC100509457
249	221566_s_at	-2.920492593	7.11E-14	NOL3
250	224428_s_at	-2.919585185	2.10E-11	CDCA7
251	218663_at	-2.918688889	6.34E-08	NCAPG
252	238750_at	-2.914866667	2.33E-10	CCL28
253	207238_s_at	-2.914392593	6.07E-17	PTPRC
254	211919_s_at	-2.903514815	7.59E-17	CXCR4

255	235202_x_at	-2.901207407	1.61E-11	IKBIP
256	210538_s_at	-2.900477778	2.87E-14	BIRC3
257	228394_at	-2.900266667	1.01E-10	STK10
258	210495_x_at	-2.899833333	4.51E-15	FN1
259	210340_s_at	-2.898562963	5.52E-12	CSF2RA
260	211844_s_at	-2.897377778	1.07E-09	NRP2
261	229437_at	-2.896722222	2.36E-11	MIR155 /// MIR155HG
262	235964_x_at	-2.894596296	1.50E-14	SAMHD1
263	210972_x_at	-2.890314815	3.99E-10	TRAC /// TRAJ17 /// TRAV20 /// TRDV2
264	202499_s_at	-2.889740741	5.15E-09	SLC2A3
265	204913_s_at	-2.888714815	8.33E-07	SOX11
266	200831_s_at	-2.886981481	1.71E-11	SCD
267	223381_at	-2.886740741	3.19E-08	NUF2
268	204015_s_at	-2.886666667	8.58E-12	DUSP4
269	202803_s_at	-2.885818519	1.43E-17	ITGB2
270	226743_at	-2.876974074	8.33E-17	SLFN11
271	228563_at	-2.872437037	9.19E-09	GJC1
272	202619_s_at	-2.8719	9.23E-13	PLOD2
273	204103_at	-2.871762963	2.00E-10	CCL4
274	219719_at	-2.86767037	6.88E-10	HIGD1B
275	206060_s_at	-2.864085185	2.42E-10	PTPN22
276	210163_at	-2.853692593	5.27E-10	CXCL11
277	223488_s_at	-2.851959259	1.53E-14	GNB4
278	205569_at	-2.849366667	5.47E-09	LAMP3
279	230966_at	-2.849014815	7.58E-13	IL4I1
280	206366_x_at	-2.847066667	6.03E-11	XCL1
281	230550_at	-2.846507407	2.29E-14	MS4A6A
282	205128_x_at	-2.845337037	1.00E-10	PTGS1
283	204914_s_at	-2.84382963	1.27E-06	SOX11
284	205269_at	-2.837522222	1.96E-16	LCP2
285	209955_s_at	-2.834618519	1.39E-09	FAP
286	203923_s_at	-2.832833333	2.97E-17	CYBB
287	202580_x_at	-2.826988889	9.64E-09	FOXM1
288	214978_s_at	-2.824188889	4.54E-07	PPFIA4
289	1555758_a_at	-2.815251852	1.47E-08	CDKN3
290	226311_at	-2.813448148	1.07E-06	ADAMTS2
291	214467_at	-2.812677778	2.80E-12	GPR65
292	206214_at	-2.809737037	7.64E-09	PLA2G7
293	202206_at	-2.809362963	7.23E-12	ARL4C
294	1570329_at	-2.806737037	4.05E-08	
295	206866_at	-2.805385185	4.96E-07	CDH4
296	204222_s_at	-2.804555556	6.70E-17	GLIPR1
297	219519_s_at	-2.804188889	1.24E-09	SIGLEC1

298	202800_at	-2.802962963	1.29E-13	SLC1A3
299	1554406_a_at	-2.7999	1.40E-12	CLEC7A
300	225081_s_at	-2.791085185	9.94E-17	CDCA7L
301	240152_at	-2.787088889	3.36E-09	
302	210287_s_at	-2.786685185	5.90E-08	FLT1
303	203066_at	-2.785844444	1.60E-14	CHST15
304	222848_at	-2.784485185	2.10E-10	CENPK
305	202498_s_at	-2.784474074	3.17E-10	SLC2A3
306	208965_s_at	-2.783622222	7.20E-20	IFI16
307	201939_at	-2.777062963	9.08E-15	PLK2
308	205258_at	-2.776925926	5.67E-09	INHBB
309	213975_s_at	-2.77437037	1.40E-12	LYZ
310	215509_s_at	-2.774307407	4.82E-08	BUB1
311	226452_at	-2.772796296	3.46E-17	PDK1
312	1552507_at	-2.771214815	7.85E-10	KCNE4
313	240137_at	-2.768292593	4.21E-07	
314	235385_at	-2.767766667	2.68E-15	1-Mar
315	201721_s_at	-2.76712963	1.12E-18	LAPTM5
316	227346_at	-2.766766667	3.25E-14	IKZF1
317	215465_at	-2.764033333	6.96E-07	ABCA12
318	45297_at	-2.762814815	2.07E-17	EHD2
319	237261_at	-2.7623	1.08E-08	ANGPT2
320	207691_x_at	-2.758018519	9.07E-15	ENTPD1
321	204971_at	-2.755177778	1.65E-15	CSTA
322	203470_s_at	-2.754648148	4.04E-13	PLEK
323	1552846_s_at	-2.750437037	1.77E-11	RAB42
324	208423_s_at	-2.750348148	8.05E-11	MSR1
325	236313_at	-2.749937037	1.55E-08	CDKN2B
326	1552798_a_at	-2.747403704	2.78E-12	TLR4
327	219232_s_at	-2.747048148	5.59E-09	EGLN3
328	223343_at	-2.745207407	2.24E-14	MS4A7
329	221581_s_at	-2.743777778	9.24E-17	LAT2
330	217466_x_at	-2.737377778	8.64E-17	RPS2 /// SNORA64
331	202202_s_at	-2.735055556	4.58E-12	LAMA4
332	220651_s_at	-2.729166667	4.38E-07	MCM10
333	204774_at	-2.728459259	1.51E-15	EVI2A
334	243764_at	-2.722333333	1.60E-09	VSIG1
335	230276_at	-2.719755556	7.35E-13	FAM49A
336	219619_at	-2.719292593	8.23E-06	DIRAS2
337	205127_at	-2.718755556	1.51E-06	PTGS1
338	203418_at	-2.716988889	3.72E-10	CCNA2
339	211902_x_at	-2.711574074	3.06E-09	YME1L1
340	213603_s_at	-2.710877778	4.28E-17	RAC2

341	209201_x_at	-2.709911111	8.66E-16	CXCR4
342	235751_s_at	-2.706796296	3.76E-09	VMO1
343	202997_s_at	-2.703918519	8.62E-08	LOXL2
344	1558972_s_at	-2.70317037	1.59E-07	THEMIS
345	228499_at	-2.703144444	6.93E-14	PFKFB4
346	215049_x_at	-2.701848148	8.15E-12	CD163
347	225626_at	-2.701351852	9.97E-17	PAG1
348	1552584_at	-2.700648148	6.69E-12	IL12RB1
349	230252_at	-2.700611111	2.33E-14	LPAR5
350	1555725_a_at	-2.698259259	4.70E-07	RGS5
351	216705_s_at	-2.698055556	4.84E-11	ADA
352	205495_s_at	-2.694348148	1.53E-08	GNLY
353	221666_s_at	-2.690207407	6.04E-15	PYCARD
354	202497_x_at	-2.690144444	1.17E-08	SLC2A3
355	221583_s_at	-2.689722222	6.69E-09	KCNMA1
356	227607_at	-2.688803704	3.22E-17	STAMBPL1
357	1559502_s_at	-2.68877037	6.22E-13	LRRC25
358	212587_s_at	-2.6867	2.54E-17	PTPRC
359	228532_at	-2.682059259	5.02E-15	C1orf162
360	211395_x_at	-2.679788889	3.10E-13	FCGR2C
361	223502_s_at	-2.677662963	1.96E-15	TNFSF13B
362	1558971_at	-2.676303704	5.88E-07	THEMIS
363	205885_s_at	-2.674403704	2.33E-14	ITGA4
364	202431_s_at	-2.669207407	1.88E-14	MYC
365	201858_s_at	-2.668485185	1.58E-16	SRGN
366	223610_at	-2.66707037	4.94E-12	SEMA5B
367	206584_at	-2.662551852	3.03E-14	LY96
368	226906_s_at	-2.661540741	1.69E-11	ARHGAP9
369	213537_at	-2.658733333	1.55E-09	HLA-DPA1
370	206785_s_at	-2.656544444	4.18E-08	KLRC1 /// KLRC2
371	213906_at	-2.656477778	1.08E-12	MYBL1
372	202901_x_at	-2.652340741	1.56E-13	CTSS
373	212873_at	-2.649322222	4.85E-11	HMHA1
374	211654_x_at	-2.648485185	3.19E-12	HLA-DQB1
375	225701_at	-2.640985185	5.13E-10	AKNA
376	37145_at	-2.640559259	1.34E-09	GNLY
377	209182_s_at	-2.636040741	1.36E-12	C10orf10
378	205046_at	-2.635185185	2.52E-07	CENPE
379	230391_at	-2.634218519	1.09E-13	CD84
380	1553299_at	-2.633818519	2.15E-09	DUSP5P1
381	1554834_a_at	-2.632622222	2.56E-10	RASSF5
382	203760_s_at	-2.6306	1.61E-14	SLA
383	228033_at	-2.630222222	1.60E-07	E2F7

384	222088_s_at	-2.629674074	3.19E-10	SLC2A14 /// SLC2A3
385	202310_s_at	-2.625562963	1.62E-07	COL1A1
386	219892_at	-2.62377037	3.86E-16	TM6SF1
387	228438_at	-2.622955556	7.18E-14	LOC100132891
388	201105_at	-2.621596296	4.37E-19	LGALS1
389	213343_s_at	-2.616348148	6.33E-10	GDPD5
390	218009_s_at	-2.615625926	7.26E-11	PRC1
391	209555_s_at	-2.613511111	5.62E-07	CD36
392	212488_at	-2.613477778	2.08E-07	COL5A1
393	210362_x_at	-2.607240741	7.24E-11	PML
394	229638_at	-2.605251852	2.54E-12	IRX3
395	242037_at	-2.60487037	1.31E-09	ASPH
396	211367_s_at	-2.601748148	9.96E-15	CASP1
397	225622_at	-2.593555556	4.38E-18	PAG1
398	221658_s_at	-2.593003704	3.95E-11	IL21R
399	229560_at	-2.590574074	8.17E-14	TLR8
400	236641_at	-2.589185185	9.10E-09	KIF14
401	223809_at	-2.58652963	7.49E-11	RGS18
402	204726_at	-2.586285185	5.19E-08	CDH13
403	229625_at	-2.584507407	1.88E-10	GBP5
404	203185_at	-2.582574074	1.24E-15	RASSF2
405	206120_at	-2.581337037	1.55E-12	CD33
406	226034_at	-2.580974074	2.46E-11	DUSP4
407	205831_at	-2.577559259	1.49E-10	CD2
408	205884_at	-2.572996296	3.03E-12	ITGA4
409	226142_at	-2.572874074	4.35E-14	GLIPR1
410	229041_s_at	-2.569488889	9.04E-09	ITGB2-AS1
411	204279_at	-2.56682963	2.05E-18	PSMB9
412	222911_s_at	-2.564314815	1.69E-08	CXorf36
413	33304_at	-2.564266667	5.22E-12	ISG20
414	218084_x_at	-2.560581481	1.14E-18	FXYD5
415	205488_at	-2.560092593	9.92E-12	GZMA
416	222680_s_at	-2.559133333	4.05E-10	DTL
417	204286_s_at	-2.556977778	2.80E-07	PMAIP1
418	205524_s_at	-2.556462963	3.34E-08	HAPLN1
419	205034_at	-2.555925926	2.50E-10	CCNE2
420	213416_at	-2.554388889	7.33E-16	ITGA4
421	204959_at	-2.554311111	4.37E-16	MNDA
422	209584_x_at	-2.549981481	6.69E-17	APOBEC3C
423	209879_at	-2.549007407	3.21E-13	SELPLG
424	219452_at	-2.546833333	1.34E-08	DPEP2
425	208966_x_at	-2.546222222	1.22E-19	IFI16
426	203645_s_at	-2.545448148	3.03E-12	CD163

427	226659_at	-2.539344444	1.05E-09	DEF6
428	223922_x_at	-2.539292593	8.23E-15	MS4A6A
429	202705_at	-2.537011111	2.73E-08	CCNB2
430	209732_at	-2.536040741	1.14E-13	CLEC2B
431	213599_at	-2.535940741	1.54E-09	OIP5
432	202589_at	-2.535140741	1.32E-14	TYMS
433	219424_at	-2.533803704	1.06E-10	EBI3
434	219594_at	-2.531192593	3.32E-11	NINJ2
435	209156_s_at	-2.529696296	4.01E-10	COL6A2
436	210889_s_at	-2.52907037	2.32E-09	FCGR2B
437	230710_at	-2.525162963	6.51E-10	MIR210HG
438	219386_s_at	-2.52047037	3.98E-13	SLAMF8
439	203967_at	-2.519551852	1.92E-09	CDC6
440	206765_at	-2.519544444	4.71E-11	KCNJ2
441	213349_at	-2.518907407	4.60E-10	TMCC1
442	202912_at	-2.5183	2.64E-12	ADM
443	212588_at	-2.514992593	3.35E-15	PTPRC
444	224027_at	-2.513474074	5.28E-08	CCL28
445	227347_x_at	-2.511388889	1.45E-10	HES4
446	204924_at	-2.511166667	3.78E-15	TLR2
447	205695_at	-2.505307407	7.43E-12	SDS
448	223839_s_at	-2.505192593	2.03E-12	
449	225799_at	-2.504477778	1.56E-17	LINC00152 /// LOC101930489 /// MIR4435-1HG
450	204126_s_at	-2.503759259	6.71E-08	CDC45
451	231925_at	-2.500718519	1.79E-08	RP11-38P22.2
452	211991_s_at	-2.499807407	1.73E-17	HLA-DPA1
453	219282_s_at	-2.499444444	1.16E-12	TRPV2
454	214297_at	-2.497111111	4.76E-07	CSPG4
455	203764_at	-2.494548148	2.52E-09	DLGAP5
456	236988_x_at	-2.493418519	1.35E-07	ITGB2
457	210116_at	-2.492348148	3.47E-08	SH2D1A
458	235885_at	-2.488207407	1.60E-08	P2RY12
459	203213_at	-2.488203704	3.85E-11	CDK1
460	202208_s_at	-2.486285185	7.58E-08	ARL4C
461	223501_at	-2.485859259	3.57E-15	TNFSF13B
462	1557905_s_at	-2.485014815	1.47E-11	CD44
463	214560_at	-2.48467037	1.84E-12	FPR3
464	211012_s_at	-2.484511111	3.83E-10	PML
465	221602_s_at	-2.481981481	5.24E-09	FAIM3
466	210298_x_at	-2.477403704	4.56E-07	FHL1
467	232693_s_at	-2.474851852	4.06E-12	FBXO16 /// ZNF395
468	205106_at	-2.470466667	8.81E-09	CMC4 /// MTCP1
469	1565627_a_at	-2.469125926	5.45E-13	

470	229411_at	-2.467362963	5.52E-06	PNCK
471	228766_at	-2.466207407	7.10E-07	CD36
472	235709_at	-2.465933333	1.54E-08	GAS2L3
473	238429_at	-2.465411111	9.53E-09	TMEM71
474	220485_s_at	-2.4616	6.82E-07	SIRPG
475	205681_at	-2.459307407	1.57E-11	BCL2A1
476	211530_x_at	-2.458959259	6.54E-16	HLA-G
477	205419_at	-2.456903704	9.63E-11	GPR183
478	219947_at	-2.456018519	7.58E-13	CLEC4A
479	204232_at	-2.453685185	5.53E-16	FCER1G
480	204891_s_at	-2.451111111	1.90E-09	LCK
481	219593_at	-2.450796296	1.02E-13	SLC15A3
482	205744_at	-2.449737037	2.92E-09	DOC2A
483	212977_at	-2.449225926	8.13E-12	ACKR3
484	207610_s_at	-2.448222222	4.66E-10	EMR2
485	225834_at	-2.447437037	9.75E-12	FAM72A /// FAM72B /// FAM72C /// FAM72D
486	1552398_a_at	-2.446922222	1.48E-08	CLEC12A
487	201819_at	-2.443096296	6.50E-11	SCARB1
488	239410_at	-2.442948148	8.69E-10	HK2 /// RP11-259N19.1
489	213539_at	-2.440425926	1.43E-10	CD3D
490	228863_at	-2.439581481	1.48E-09	PCDH17
491	34210_at	-2.4356	2.82E-10	CD52
492	242388_x_at	-2.432037037	1.09E-08	TAGAP
493	206369_s_at	-2.431855556	2.70E-10	PIK3CG
494	201438_at	-2.430603704	1.82E-07	COL6A3
495	204736_s_at	-2.428718519	2.18E-08	CSPG4
496	235733_at	-2.427933333	5.05E-10	GXYLT2
497	223773_s_at	-2.427118519	1.71E-12	SNHG12 /// SNORA16A /// SNORA44 /// SNORA61
498	200951_s_at	-2.4265	5.25E-09	CCND2
499	210644_s_at	-2.425803704	7.51E-16	LAIR1
500	223660_at	-2.423966667	6.38E-09	ADORA3
501	204822_at	-2.422811111	1.88E-07	TTK
502	222877_at	-2.421559259	3.66E-10	NRP2
503	223774_at	-2.420211111	4.08E-10	SNHG12 /// SNORA16A /// SNORA44 /// SNORA61
504	211286_x_at	-2.419466667	6.26E-14	CSF2RA
505	223216_x_at	-2.418633333	6.00E-13	FBXO16 /// ZNF395
506	204174_at	-2.415737037	3.23E-16	ALOX5AP
507	229635_at	-2.414551852	1.16E-12	LINC01094
508	210553_x_at	-2.412766667	1.43E-06	LOC100507472 /// PCSK6
509	211656_x_at	-2.412514815	1.31E-10	HLA-DQB1 /// LOC101060835
510	211527_x_at	-2.412303704	4.43E-09	VEGFA

511	1553781_at	-2.411225926	1.13E-06	ZC3HAV1L
512	209070_s_at	-2.410496296	7.45E-07	RGS5
513	217297_s_at	-2.410355556	3.81E-10	MYO9B
514	229800_at	-2.409562963	2.28E-11	DCLK1
515	240070_at	-2.408192593	1.14E-07	TIGIT
516	222536_s_at	-2.407996296	1.70E-11	ZNF395
517	203561_at	-2.404625926	4.28E-17	FCGR2A
518	236471_at	-2.40242963	9.93E-10	NFE2L3
519	221698_s_at	-2.401796296	5.04E-14	CLEC7A
520	1568813_at	-2.401633333	2.05E-06	SLC16A1-AS1
521	239294_at	-2.401540741	1.97E-14	PIK3CG
522	222305_at	-2.399348148	8.63E-10	HK2 /// RP11-259N19.1
523	224225_s_at	-2.397688889	8.74E-08	ETV7
524	201666_at	-2.395344444	3.26E-15	TIMP1
525	205312_at	-2.393037037	2.09E-08	SPI1
526	211742_s_at	-2.39267037	4.94E-17	EVI2B
527	211013_x_at	-2.390377778	2.13E-11	PML
528	232617_at	-2.389459259	3.02E-15	CTSS
529	234987_at	-2.389081481	3.28E-16	SAMHD1
530	203940_s_at	-2.385814815	8.40E-12	VASH1
531	223562_at	-2.383892593	1.19E-11	PARVG
532	202957_at	-2.383033333	4.91E-17	HCLS1
533	1560396_at	-2.379925926	3.78E-09	KLHL6
534	222958_s_at	-2.378707407	1.29E-08	DEPDC1
535	205859_at	-2.378007407	4.20E-17	LY86
536	202887_s_at	-2.377633333	1.70E-10	DDIT4
537	238581_at	-2.375707407	3.05E-07	GBP5
538	209360_s_at	-2.374962963	1.76E-13	LOC100506403 /// LOC101928269 /// RUNX1
539	222288_at	-2.374951852	1.23E-06	
540	240983_s_at	-2.372674074	2.67E-09	CARS
541	203323_at	-2.371196296	6.12E-15	CAV2
542	210869_s_at	-2.366844444	3.42E-14	MCAM /// MIR6756
543	1557078_at	-2.365362963	2.02E-08	SLFN5
544	213113_s_at	-2.365285185	5.83E-13	SLC43A3
545	205729_at	-2.36412963	2.00E-13	OSMR
546	223620_at	-2.359040741	5.86E-15	GPR34
547	219890_at	-2.356748148	1.29E-08	CLEC5A
548	211210_x_at	-2.354911111	4.96E-09	SH2D1A
549	204236_at	-2.354222222	1.02E-14	FLI1
550	208747_s_at	-2.352844444	6.90E-12	C1S
551	218223_s_at	-2.35157037	1.14E-14	PLEKHO1
552	211829_s_at	-2.350733333	2.34E-08	GPER1
553	201761_at	-2.349007407	1.02E-13	MTHFD2

554	228056_s_at	-2.348755556	3.58E-07	NAPSB
555	223655_at	-2.348425926	1.26E-08	CD163L1
556	236513_at	-2.348088889	3.27E-11	PRELID2
557	218898_at	-2.345666667	8.82E-15	FAM57A
558	213418_at	-2.3452	9.13E-09	HSPA6
559	214845_s_at	-2.34497037	2.42E-12	CALU
560	218149_s_at	-2.344877778	1.14E-13	ZNF395
561	211368_s_at	-2.344037037	1.16E-16	CASP1
562	205291_at	-2.341207407	4.71E-11	IL2RB
563	230464_at	-2.34082963	3.30E-08	S1PR5
564	201137_s_at	-2.340581481	1.21E-13	HLA-DPB1
565	206332_s_at	-2.340496296	7.09E-18	IFI16
566	202403_s_at	-2.3397	2.44E-09	COL1A2
567	204787_at	-2.339359259	9.80E-14	VSIG4
568	210206_s_at	-2.339277778	2.00E-09	DDX11
569	230962_at	-2.338233333	1.29E-06	DCLK1
570	227211_at	-2.336803704	2.16E-08	PHF19
571	216191_s_at	-2.336340741	1.18E-06	TRDV3
572	223700_at	-2.332148148	1.59E-06	MND1
573	212658_at	-2.33177037	4.89E-15	LHFPL2
574	207651_at	-2.331111111	4.59E-08	GPR171
575	219434_at	-2.331018519	7.92E-06	TREM1
576	206513_at	-2.328159259	9.95E-08	AIM2
577	230664_at	-2.326755556	6.26E-08	H2BFXP
578	219634_at	-2.325314815	7.49E-10	CHST11
579	205285_s_at	-2.3248	5.02E-10	FYB
580	211794_at	-2.322251852	1.04E-11	FYB
581	210321_at	-2.321755556	6.93E-11	GZMH
582	222891_s_at	-2.320959259	2.65E-07	BCL11A
583	203416_at	-2.320759259	1.68E-17	CD53
584	209835_x_at	-2.320474074	6.39E-11	CD44
585	203554_x_at	-2.319222222	1.74E-12	PTTG1
586	229543_at	-2.319037037	5.24E-12	RP1-93H18.6
587	218355_at	-2.315114815	1.68E-10	KIF4A
588	226498_at	-2.315044444	5.48E-09	FLT1
589	238551_at	-2.313414815	7.37E-14	FUT11
590	1556499_s_at	-2.310848148	8.32E-09	COL1A1
591	1552703_s_at	-2.310066667	1.38E-14	CARD16 /// CASP1
592	204152_s_at	-2.306933333	9.51E-12	MFNG
593	204915_s_at	-2.306633333	7.20E-07	SOX11
594	238756_at	-2.305692593	5.11E-12	GAS2L3
595	205898_at	-2.305207407	8.95E-10	CX3CR1
596	209906_at	-2.305207407	2.88E-13	C3AR1

597	212063_at	-2.304848148	6.92E-13	CD44
598	205624_at	-2.303851852	4.43E-06	CPA3
599	211864_s_at	-2.3038	6.63E-13	MYOF
600	216120_s_at	-2.300822222	4.25E-07	ATP2B2
601	238021_s_at	-2.300762963	1.12E-10	CRNDE
602	205789_at	-2.297014815	6.58E-13	CD1D
603	1552717_s_at	-2.296011111	1.01E-10	CEP170 /// CEP170P1
604	205345_at	-2.294940741	4.58E-08	BARD1
605	205952_at	-2.294311111	5.11E-07	KCNK3
606	219148_at	-2.29352963	1.04E-09	PBK
607	210146_x_at	-2.2931	1.34E-09	LILRB2
608	206486_at	-2.29262963	9.54E-07	LAG3
609	203358_s_at	-2.291574074	1.32E-08	EZH2
610	209727_at	-2.290077778	1.63E-09	GM2A
611	227295_at	-2.287274074	8.55E-16	IKBIP
612	221031_s_at	-2.285562963	1.32E-07	APOLD1
613	218782_s_at	-2.283544444	2.11E-09	ATAD2
614	204639_at	-2.282403704	8.18E-12	ADA
615	223533_at	-2.280977778	9.98E-12	LOC101927933 /// LRRC8C
616	202990_at	-2.278614815	2.40E-16	PYGL
617	213589_s_at	-2.276362963	7.43E-08	B3GNTL1
618	208010_s_at	-2.275185185	5.20E-06	PTPN22
619	202391_at	-2.272844444	3.74E-09	BASP1
620	216236_s_at	-2.270759259	8.58E-10	SLC2A14 /// SLC2A3
621	221123_x_at	-2.270207407	4.58E-12	FBXO16 /// ZNF395
622	203755_at	-2.269555556	7.90E-09	BUB1B
623	204489_s_at	-2.268840741	1.24E-10	CD44
624	211924_s_at	-2.268277778	7.56E-08	PLAUR
625	244352_at	-2.268059259	3.40E-13	CD84
626	206171_at	-2.267807407	7.12E-13	ADORA3
627	1552807_a_at	-2.267566667	1.71E-12	SIGLEC10
628	220132_s_at	-2.265251852	8.94E-08	CLEC2D
629	222740_at	-2.264766667	1.12E-08	ATAD2
630	212858_at	-2.264637037	2.56E-09	PAQR4
631	214452_at	-2.264018519	1.34E-06	BCAT1
632	213010_at	-2.262177778	6.43E-15	PRKCDBP
633	201564_s_at	-2.2609	1.21E-09	FSCN1
634	205504_at	-2.260322222	6.34E-13	BTK
635	204490_s_at	-2.259788889	6.45E-09	CD44
636	214032_at	-2.259555556	4.26E-08	ZAP70
637	240413_at	-2.259362963	2.71E-06	PYHIN1
638	229723_at	-2.256837037	1.44E-14	TAGAP
639	221567_at	-2.254751852	6.40E-15	NOL3

640	204912_at	-2.254462963	2.59E-15	IL10RA
641	241068_at	-2.251766667	4.82E-09	IGSF6
642	220005_at	-2.246959259	4.60E-12	P2RY13
643	204146_at	-2.246811111	3.14E-10	RAD51AP1
644	227606_s_at	-2.246037037	5.94E-16	STAMBPL1
645	1556423_at	-2.245066667	5.55E-08	VASH1
646	208711_s_at	-2.24417037	5.30E-11	CCND1
647	201668_x_at	-2.243740741	1.07E-14	MARCKS
648	208594_x_at	-2.243540741	3.72E-11	LILRA6
649	214617_at	-2.242592593	1.12E-11	PRF1
650	232024_at	-2.242577778	2.13E-13	GIMAP2
651	203741_s_at	-2.239218519	2.95E-16	ADCY7
652	205447_s_at	-2.23802963	2.06E-11	MAP3K12
653	219457_s_at	-2.237688889	2.35E-10	RIN3
654	219279_at	-2.23742963	8.89E-18	DOCK10
655	209734_at	-2.237037037	5.24E-11	NCKAP1L
656	201389_at	-2.237022222	3.41E-11	ITGA5
657	203085_s_at	-2.236744444	1.76E-15	TGFB1
658	202910_s_at	-2.23532963	2.62E-15	CD97
659	202270_at	-2.234422222	4.57E-12	GBP1
660	226219_at	-2.232592593	1.29E-15	ARHGAP30
661	203523_at	-2.232525926	2.17E-11	LSP1
662	205988_at	-2.231762963	1.62E-14	CD84
663	1559883_s_at	-2.23122963	1.76E-10	SAMHD1
664	228401_at	-2.228148148	1.86E-08	ATAD2
665	218883_s_at	-2.2273	3.24E-13	CENPU
666	239237_at	-2.226825926	3.43E-08	TRG-AS1
667	226818_at	-2.226333333	5.16E-14	MPEG1
668	202022_at	-2.223181481	8.71E-12	ALDOC
669	205692_s_at	-2.222940741	9.26E-07	CD38
670	215925_s_at	-2.221311111	6.82E-10	CD72
671	209546_s_at	-2.220574074	1.71E-08	APOL1
672	210140_at	-2.220196296	2.23E-10	CST7
673	235291_s_at	-2.219933333	1.14E-09	FLJ32255
674	1552806_a_at	-2.218407407	2.74E-10	SIGLEC10
675	208096_s_at	-2.217062963	3.15E-07	COL21A1
676	242587_at	-2.215262963	3.17E-07	SLC9A9
677	228964_at	-2.214803704	6.24E-14	PRDM1
678	227354_at	-2.213722222	1.80E-17	PAG1
679	233500_x_at	-2.211955556	8.09E-09	CLEC2D
680	217967_s_at	-2.209488889	4.76E-15	FAM129A
681	1554768_a_at	-2.208511111	1.00E-09	MAD2L1

682	212949_at	-2.208207407	2.42E-06	NCAPH
683	209191_at	-2.207207407	1.02E-14	TUBB6
684	1557545_s_at	-2.206811111	1.48E-08	RNF165
685	215834_x_at	-2.206618519	1.39E-08	SCARB1
686	201743_at	-2.206488889	3.15E-14	CD14
687	203968_s_at	-2.206	4.08E-10	CDC6
688	220658_s_at	-2.204477778	3.86E-06	ARNTL2
689	204802_at	-2.202014815	1.62E-07	RRAD
690	215223_s_at	-2.201940741	9.58E-10	LOC100129518 /// SOD2
691	238668_at	-2.199666667	2.52E-14	NCKAP1L
692	207828_s_at	-2.199555556	2.81E-08	CENPF
693	209891_at	-2.198092593	3.68E-08	SPC25
694	242714_at	-2.193348148	3.21E-12	LOC101928429
695	226991_at	-2.19322963	5.96E-10	NFATC2
696	207723_s_at	-2.193140741	6.04E-08	KLRC3
697	228997_at	-2.193140741	2.67E-08	TRNAU1AP
698	235735_at	-2.190559259	3.37E-11	TNFSF8
699	226841_at	-2.190166667	4.54E-12	MPEG1
700	209183_s_at	-2.188725926	7.99E-13	C10orf10
701	228362_s_at	-2.188351852	8.58E-07	FAM26F
702	231772_x_at	-2.188351852	1.16E-14	CENPH
703	204221_x_at	-2.188318519	2.28E-11	GLIPR1
704	224451_x_at	-2.188292593	2.96E-14	ARHGAP9
705	1558217_at	-2.187003704	1.25E-10	SLFN13
706	222936_s_at	-2.186255556	9.38E-08	DESI2
707	225285_at	-2.186159259	1.42E-08	BCAT1
708	204205_at	-2.1849	1.35E-15	APOBEC3G
709	208018_s_at	-2.184148148	1.68E-14	HCK
710	204963_at	-2.183255556	3.37E-14	SSPN
711	204502_at	-2.182948148	2.84E-15	SAMHD1
712	1553159_at	-2.181907407	2.49E-06	DNAH11
713	202686_s_at	-2.180425926	3.04E-17	AXL
714	1557918_s_at	-2.178403704	1.40E-08	SLC16A1
715	217966_s_at	-2.176537037	1.83E-14	FAM129A
716	232722_at	-2.176281481	7.48E-07	RNASET2
717	227212_s_at	-2.173318519	7.65E-09	PHF19
718	202311_s_at	-2.173192593	5.16E-06	COL1A1
719	227628_at	-2.171618519	6.66E-12	GPX8
720	217246_s_at	-2.170185185	6.90E-08	DIAPH2
721	209598_at	-2.168785185	2.01E-07	PNMA2
722	230925_at	-2.168192593	6.80E-12	APBB1IP
723	221584_s_at	-2.164274074	1.06E-10	KCNMA1
724	235740_at	-2.162977778	4.58E-08	MCTP1

725	238669_at	-2.162196296	1.14E-06	PTGS1
726	219497_s_at	-2.159333333	1.14E-06	BCL11A
727	209928_s_at	-2.156848148	2.33E-10	MSC
728	1553055_a_at	-2.156048148	2.92E-08	SLFN5
729	234992_x_at	-2.15537037	2.14E-06	ECT2
730	204890_s_at	-2.1552	8.68E-08	LCK
731	227566_at	-2.154625926	2.09E-07	LOC102725271 /// NTM
732	202307_s_at	-2.153937037	1.14E-14	TAP1
733	236420_s_at	-2.152733333	3.99E-07	ANO4
734	214438_at	-2.151703704	2.96E-09	HLX
735	209949_at	-2.151459259	3.20E-10	NCF2
736	229045_at	-2.151266667	5.05E-07	SNX20
737	222379_at	-2.149862963	2.49E-09	KCNE4
738	224357_s_at	-2.149155556	2.85E-11	MS4A4A
739	222662_at	-2.147792593	4.42E-19	PPP1R3B
740	211003_x_at	-2.14672963	3.21E-08	TGM2
741	244598_at	-2.146637037	1.70E-11	LCP2
742	215891_s_at	-2.142733333	8.30E-11	GM2A
743	218870_at	-2.142218519	2.66E-09	ARHGAP15 /// LOC101928361
744	204899_s_at	-2.141248148	1.37E-11	SAP30
745	219700_at	-2.141159259	4.61E-13	PLXDC1
746	1554240_a_at	-2.141022222	1.74E-14	ITGAL
747	226244_at	-2.138977778	2.06E-07	CLEC14A
748	201116_s_at	-2.136977778	5.18E-06	CPE
749	204661_at	-2.134107407	3.39E-09	CD52
750	212613_at	-2.133159259	1.09E-08	BTN3A2
751	225057_at	-2.13062963	3.76E-16	SLC15A4
752	210513_s_at	-2.129833333	5.10E-08	VEGFA
753	210915_x_at	-2.129525926	1.26E-10	TRBC1
754	219014_at	-2.129496296	6.96E-08	PLAC8
755	209087_x_at	-2.12527037	2.88E-13	MCAM
756	232234_at	-2.123088889	1.17E-09	SLA2
757	209040_s_at	-2.122577778	1.20E-18	PSMB8
758	213008_at	-2.118851852	1.72E-09	FANCI
759	59625_at	-2.117488889	5.93E-13	NOL3
760	212014_x_at	-2.117140741	8.68E-10	CD44
761	205240_at	-2.116748148	3.73E-08	GPSM2
762	213125_at	-2.116537037	2.69E-11	OLFML2B
763	210132_at	-2.114781481	3.73E-12	EFNA3
764	208107_s_at	-2.114059259	7.11E-08	LOC81691
765	202659_at	-2.11237037	3.78E-15	PSMB10
766	210559_s_at	-2.111407407	1.25E-08	CDK1
767	219385_at	-2.109118519	5.22E-11	SLAMF8

768	202620_s_at	-2.1073	8.12E-13	PLOD2
769	206364_at	-2.106688889	6.25E-07	KIF14
770	218802_at	-2.106274074	5.51E-11	CCDC109B
771	225566_at	-2.106044444	1.61E-10	NRP2
772	229383_at	-2.105788889	7.18E-16	1-Mar
773	219978_s_at	-2.105240741	1.62E-15	NUSAP1
774	218115_at	-2.104677778	4.15E-09	ASF1B
775	234976_x_at	-2.104133333	6.36E-06	MTHFD2
776	209071_s_at	-2.10402963	1.33E-07	RGS5
777	1554704_at	-2.104007407	3.87E-08	ATP8B3
778	205270_s_at	-2.103866667	1.34E-15	LCP2
779	215633_x_at	-2.10212963	5.72E-15	LST1
780	201798_s_at	-2.10122963	2.70E-13	MYOF
781	205758_at	-2.100103704	4.46E-06	CD8A
782	1569154_a_at	-2.100085185	3.62E-07	GRAMD4
783	205399_at	-2.099011111	3.44E-09	DCLK1
784	206011_at	-2.098177778	7.82E-16	CASP1
785	210184_at	-2.097788889	1.76E-08	ITGAX
786	201539_s_at	-2.097322222	1.53E-07	FHL1
787	210031_at	-2.097277778	6.66E-12	CD247
788	1555680_a_at	-2.095637037	1.11E-06	SMOX
789	220330_s_at	-2.095403704	7.23E-10	SAMSN1
790	212171_x_at	-2.095137037	4.34E-09	VEGFA
791	216841_s_at	-2.093318519	2.33E-10	LOC100129518 /// SOD2
792	227002_at	-2.091681481	5.21E-13	FAM78A /// LOC101927137
793	209597_s_at	-2.091485185	1.28E-06	PNMA2
794	226237_at	-2.087411111	4.61E-08	COL8A1
795	240572_s_at	-2.086781481	5.83E-08	LOC374443
796	208580_x_at	-2.08677037	5.31E-09	HIST1H4A /// HIST1H4B /// HIST1H4C /// HIST1H4D /// HIST1H4E /// HIST1H4F /// HIST1H4H /// HIST1H4I /// HIST1H4J /// HIST1H4K /// HIST1H4L /// HIST2H4A /// HIST2H4B /// HIST4H4
797	204220_at	-2.08587037	2.34E-16	GMFG
798	211162_x_at	-2.085688889	1.72E-06	SCD
799	228376_at	-2.085622222	2.68E-13	GGTA1P
800	208368_s_at	-2.083814815	1.16E-06	BRCA2
801	212489_at	-2.083011111	6.22E-07	COL5A1
802	207172_s_at	-2.081781481	2.94E-09	CDH11
803	210415_s_at	-2.080885185	1.71E-08	ODF2
804	205024_s_at	-2.080848148	7.17E-09	RAD51
805	207957_s_at	-2.080737037	3.67E-10	PRKCB
806	204118_at	-2.080648148	9.08E-12	CD48

807	207039_at	-2.079322222	2.02E-06	CDKN2A
808	219971_at	-2.079044444	1.23E-06	IL21R
809	210916_s_at	-2.076377778	3.27E-10	CD44
810	235371_at	-2.075588889	6.76E-08	GXYLT2
811	228585_at	-2.073392593	1.04E-09	ENTPD1
812	209294_x_at	-2.071733333	7.07E-10	TNFRSF10B
813	226497_s_at	-2.071422222	1.30E-08	FLT1
814	210139_s_at	-2.070666667	4.65E-14	PMP22
815	209832_s_at	-2.07042963	6.73E-08	CDT1
816	214505_s_at	-2.069811111	7.26E-07	FHL1
817	211786_at	-2.068359259	1.74E-06	TNFRSF9
818	205114_s_at	-2.067781481	1.76E-08	CCL3 /// CCL3L1 /// CCL3L3
819	228762_at	-2.067614815	4.31E-07	LFNG
820	204858_s_at	-2.067566667	2.73E-11	TYMP
821	225897_at	-2.066266667	2.44E-15	MARCKS
822	203047_at	-2.0656	8.51E-11	STK10
823	232451_at	-2.065477778	1.88E-09	RP11-274H2.5
824	229957_at	-2.064966667	1.46E-06	TMEM91
825	227799_at	-2.063551852	9.89E-09	MYO1G
826	225464_at	-2.060674074	2.32E-10	FRMD6
827	209828_s_at	-2.06067037	7.80E-06	IL16
828	214437_s_at	-2.058951852	1.30E-15	SHMT2
829	219159_s_at	-2.057596296	1.11E-07	SLAMF7
830	208982_at	-2.057025926	3.14E-11	PECAM1
831	218237_s_at	-2.055688889	1.19E-12	SLC38A1
832	204960_at	-2.054607407	1.29E-07	PTPRCAP
833	221807_s_at	-2.054440741	8.64E-13	TRABD
834	1554696_s_at	-2.053959259	2.45E-11	TYMS
835	226835_s_at	-2.053944444	8.55E-16	ZFAS1
836	215076_s_at	-2.051196296	1.22E-07	COL3A1
837	203761_at	-2.050433333	1.27E-14	SLA
838	204964_s_at	-2.048059259	3.87E-11	SSPN
839	211339_s_at	-2.046362963	6.77E-10	ITK
840	217513_at	-2.044955556	5.54E-08	MILR1
841	209611_s_at	-2.044122222	8.39E-09	SLC1A4
842	206206_at	-2.043277778	4.14E-08	CD180
843	226347_at	-2.042962963	9.32E-17	
844	228094_at	-2.041762963	2.04E-08	AMICA1
845	226621_at	-2.041274074	2.71E-14	OSMR
846	209464_at	-2.041066667	4.89E-08	AURKB
847	225673_at	-2.039774074	2.26E-14	MYADM
848	1569003_at	-2.038288889	8.19E-10	VMP1
849	217143_s_at	-2.038222222	5.61E-07	TCRDV2 /// TRDC /// TRDC /// YME1L1

850	211161_s_at	-2.03687037	3.75E-08	COL3A1
851	201482_at	-2.036440741	2.02E-08	QSOX1
852	208394_x_at	-2.036237037	6.86E-07	ESM1
853	203805_s_at	-2.035240741	1.82E-06	FANCA
854	210982_s_at	-2.034833333	6.84E-16	HLA-DRA
855	214390_s_at	-2.033559259	4.73E-06	BCAT1
856	227353_at	-2.03137037	4.27E-10	TMC8
857	1559051_s_at	-2.029081481	8.41E-08	MB21D1
858	204766_s_at	-2.025725926	1.21E-11	NUDT1
859	203922_s_at	-2.024711111	4.60E-12	CYBB
860	1552312_a_at	-2.02372963	3.94E-08	MFAP3
861	222908_at	-2.022448148	6.60E-10	PIEZO2
862	227677_at	-2.022355556	1.57E-15	JAK3
863	211599_x_at	-2.020711111	1.60E-12	MET
864	211799_x_at	-2.018925926	3.78E-16	HLA-C
865	225541_at	-2.017692593	4.25E-12	RPL22L1
866	217549_at	-2.015762963	4.00E-09	NCKAP1L
867	206907_at	-2.015225926	6.29E-09	TNFSF9
868	229373_at	-2.0149	4.07E-06	
869	219225_at	-2.014837037	2.18E-08	PGBD5
870	243154_at	-2.014396296	1.50E-06	
871	238063_at	-2.013207407	7.18E-11	TMEM154
872	202765_s_at	-2.011451852	1.74E-06	FBN1
873	210405_x_at	-2.011385185	4.07E-09	TNFRSF10B
874	225662_at	-2.00887037	7.40E-15	ZAK
875	44790_s_at	-2.008722222	3.75E-09	KIAA0226L
876	205382_s_at	-2.007214815	4.13E-07	CFD
877	211211_x_at	-2.006833333	5.80E-06	SH2D1A
878	1564027_a_at	-2.006459259	3.03E-06	FAM115C /// FAM115D ///
879	218039_at	-2.006077778	1.17E-15	NUSAP1
880	238865_at	-2.003648148	4.07E-08	PABPC4L
881	208092_s_at	-2.002696296	4.40E-12	FAM49A
882	212810_s_at	-2.002359259	3.18E-12	SLC1A4
883	209473_at	-2.001833333	6.58E-15	ENTPD1
884	242100_at	-2.0017	9.86E-07	CHSY3
885	227140_at	-2.000359259	6.33E-07	INHBA
886	201136_at	-1.99917037	2.33E-10	PLP2
887	203471_s_at	-1.997688889	9.79E-12	PLEK
888	209876_at	-1.997403704	1.46E-09	GIT2
889	214085_x_at	-1.996537037	6.62E-11	GLIPR1
890	214615_at	-1.996503704	5.36E-06	P2RY10
891	224753_at	-1.996396296	4.69E-08	CDCA5
892	231776_at	-1.994159259	1.37E-08	EOMES

893	223583_at	-1.993922222	3.17E-12	TNFAIP8L2
894	203362_s_at	-1.990874074	5.00E-12	MAD2L1
895	205798_at	-1.990203704	7.43E-07	IL7R
896	213226_at	-1.988148148	2.24E-10	CCNA2
897	1555638_a_at	-1.987959259	4.50E-09	SAMSN1
898	205081_at	-1.986455556	2.48E-07	CRIP1
899	211190_x_at	-1.986244444	1.27E-06	CD84
900	1552386_at	-1.983881481	7.13E-10	GAPT
901	211824_x_at	-1.981759259	8.52E-07	NLRP1
902	201669_s_at	-1.981522222	3.76E-16	MARCKS
903	228868_x_at	-1.978874074	5.26E-10	CDT1
904	236915_at	-1.978322222	2.14E-07	C4orf47
905	210260_s_at	-1.975374074	6.42E-14	TNFAIP8
906	204158_s_at	-1.975033333	5.65E-12	TCIRG1
907	242786_at	-1.973718519	8.09E-08	SBF2-AS1
908	210299_s_at	-1.972974074	2.81E-07	FHL1
909	235529_x_at	-1.972792593	2.87E-14	SAMHD1
910	202638_s_at	-1.972225926	3.90E-14	ICAM1
911	235572_at	-1.971755556	2.55E-08	SPC24
912	231243_s_at	-1.971366667	1.39E-07	BHLHE41
913	201670_s_at	-1.970207407	2.77E-14	MARCKS
914	35820_at	-1.969944444	3.03E-14	GM2A
915	226348_at	-1.969803704	6.62E-17	FUT11
916	235534_at	-1.968992593	4.04E-11	RP11-134G8.8
917	232918_at	-1.968425926	1.77E-09	MIR4435-1HG
918	222646_s_at	-1.968340741	4.11E-11	ERO1L
919	1552335_at	-1.96772963	7.56E-08	CATSPER1
920	203547_at	-1.96697037	9.95E-16	CD4
921	202954_at	-1.964314815	3.18E-07	UBE2C
922	225043_at	-1.96402963	3.55E-16	SLC15A4
923	1555812_a_at	-1.961451852	1.61E-15	ARHGDIB
924	239975_at	-1.960862963	3.99E-07	HLA-DPB2
925	206470_at	-1.957666667	6.84E-10	PLXNC1
926	204492_at	-1.957488889	9.90E-06	ARHGAP11A
927	226517_at	-1.957411111	4.92E-07	BCAT1
928	222644_s_at	-1.957066667	8.38E-13	COLGALT1
929	203932_at	-1.956855556	1.86E-12	HLA-DMB
930	210785_s_at	-1.956392593	2.61E-14	THEMIS2
931	203886_s_at	-1.955533333	2.03E-06	FBLN2
932	242907_at	-1.955322222	8.51E-11	GBP2
933	202898_at	-1.955251852	3.68E-08	SDC3
934	209795_at	-1.95392963	2.03E-09	CD69
935	201667_at	-1.952107407	2.20E-10	GJA1

936	234985_at	-1.952062963	3.56E-14	LDLRAD3
937	207224_s_at	-1.951574074	2.51E-10	SIGLEC7
938	207536_s_at	-1.951548148	3.42E-07	TNFRSF9
939	1552553_a_at	-1.950125926	1.88E-08	NLRC4
940	201954_at	-1.95	2.15E-13	ARPC1B
941	1566433_at	-1.949803704	6.14E-07	PLD4
942	213888_s_at	-1.948707407	8.36E-09	TRAF3IP3
943	204886_at	-1.947703704	6.29E-06	PLK4
944	211582_x_at	-1.945707407	6.22E-16	LST1
945	1554937_x_at	-1.944111111	2.13E-07	EXOC3L1
946	214321_at	-1.943481481	9.20E-07	NOV
947	230593_at	-1.942888889	1.16E-06	GRIK3
948	226372_at	-1.940040741	1.56E-13	CHST11
949	201044_x_at	-1.937292593	1.60E-07	DUSP1
950	1567628_at	-1.936059259	5.35E-12	CD74
951	227344_at	-1.935918519	2.44E-09	IKZF1
952	224790_at	-1.93507037	4.86E-15	ASAP1
953	201852_x_at	-1.934896296	1.99E-06	COL3A1
954	204567_s_at	-1.933788889	8.98E-12	ABCG1
955	210279_at	-1.933188889	7.86E-08	GPR18
956	239078_at	-1.931781481	3.74E-06	BROX
957	222020_s_at	-1.931118519	1.23E-07	LOC102725271 /// NTM
958	228708_at	-1.927244444	1.83E-06	RAB27B
959	207714_s_at	-1.926851852	7.71E-12	SERPINH1
960	205968_at	-1.923707407	2.04E-11	KCNS3
961	1555355_a_at	-1.922581481	1.03E-08	ETS1
962	214898_x_at	-1.920855556	4.09E-06	MUC3B
963	218353_at	-1.918851852	3.00E-06	RGS5
964	208885_at	-1.918511111	6.54E-14	LCP1
965	204553_x_at	-1.916459259	1.22E-08	INPP4A
966	210692_s_at	-1.914874074	1.07E-10	SLC43A3
967	212235_at	-1.913914815	2.99E-09	PLXND1
968	1555705_a_at	-1.913781481	4.95E-12	CMTM3
969	203729_at	-1.913018519	6.02E-13	EMP3
970	228167_at	-1.912462963	1.22E-10	KLHL6
971	202870_s_at	-1.911618519	2.72E-06	CDC20
972	211266_s_at	-1.910077778	7.58E-07	GPR4
973	1555691_a_at	-1.908944444	2.00E-07	KLRC4-KLRK1 /// KLRK1
974	226777_at	-1.908855556	1.42E-06	ADAM12
975	205804_s_at	-1.907481481	3.94E-07	TRAF3IP3
976	209846_s_at	-1.906148148	4.04E-10	BTN3A2
977	221729_at	-1.905777778	9.30E-08	COL5A2
978	201859_at	-1.904466667	1.62E-15	SRGN

979	227198_at	-1.903762963	8.69E-08	AFF3
980	202637_s_at	-1.903333333	1.20E-13	ICAM1
981	206148_at	-1.902888889	3.81E-07	IL3RA
982	225045_at	-1.902518519	8.20E-15	CCDC88A
983	217497_at	-1.901888889	4.73E-09	TYMP
984	1554018_at	-1.899603704	6.47E-08	GPNMB
985	219872_at	-1.897381481	1.21E-07	FAM198B
986	227868_at	-1.895196296	1.20E-12	LOC154761
987	207968_s_at	-1.894	5.27E-08	MEF2C
988	235359_at	-1.891959259	3.38E-06	NRROS
989	218611_at	-1.891862963	3.04E-17	IER5
990	207571_x_at	-1.891618519	3.09E-14	THEMIS2
991	221539_at	-1.890874074	7.95E-11	EIF4EBP1
992	206116_s_at	-1.890351852	2.82E-12	TPM1
993	201288_at	-1.890207407	7.58E-16	ARHGDIB
994	221211_s_at	-1.889944444	3.40E-07	MAP3K7CL
995	203214_x_at	-1.888774074	2.20E-07	CDK1
996	212918_at	-1.888755556	3.17E-17	RECQL
997	209970_x_at	-1.88852963	2.91E-15	CASP1
998	206761_at	-1.887303704	5.29E-06	CD96
999	230161_at	-1.886925926	1.85E-07	
1000	224579_at	-1.884362963	5.92E-12	SLC38A1
1001	237585_at	-1.882037037	4.71E-08	C4orf47
1002	231577_s_at	-1.881177778	7.47E-10	GBP1
1003	221840_at	-1.881044444	1.24E-15	PTPRE
1004	230466_s_at	-1.879481481	3.32E-14	RASSF3
1005	218600_at	-1.879314815	3.28E-07	LIMD2
1006	223303_at	-1.87917037	2.24E-07	FERMT3
1007	212385_at	-1.876807407	2.76E-09	TCF4
1008	214463_x_at	-1.8758	2.04E-08	HIST1H4J
1009	213807_x_at	-1.875496296	1.04E-09	MET
1010	220615_s_at	-1.874966667	1.70E-11	FAR2
1011	226955_at	-1.874618519	1.03E-11	AFAP1L1
1012	212746_s_at	-1.874455556	3.15E-10	CEP170 /// CEP170P1
1013	205922_at	-1.874288889	2.09E-07	VNN2
1014	1559584_a_at	-1.870592593	5.20E-09	C16orf54
1015	204444_at	-1.87012963	5.71E-08	KIF11
1016	228245_s_at	-1.869518519	1.59E-06	LOC100509445 /// LOC728715 /// OVOS /// OVOS2
1017	235574_at	-1.869396296	1.08E-08	GBP4
1018	1558750_a_at	-1.867788889	9.86E-07	ARHGAP11B /// LOC100288637
1019	206271_at	-1.865918519	1.89E-07	TLR3
1020	1552701_a_at	-1.865403704	6.82E-13	CARD16

1021	236179_at	-1.864477778	1.27E-06	CDH11
1022	201984_s_at	-1.863944444	2.64E-08	EGFR
1023	223307_at	-1.863144444	8.58E-10	CDCA3
1024	214084_x_at	-1.861814815	2.85E-09	NCF1
1025	219812_at	-1.861740741	1.28E-06	PVRIG
1026	1555167_s_at	-1.860737037	1.48E-08	NAMPT
1027	203167_at	-1.859425926	5.89E-11	TIMP2
1028	222449_at	-1.859403704	6.25E-12	PMEPA1
1029	225699_at	-1.858407407	2.83E-11	LOC101928927 /// SNHG15 /// SNORA9
1030	221601_s_at	-1.85752963	6.68E-08	FAIM3
1031	239108_at	-1.856359259	7.39E-09	FAR2
1032	221521_s_at	-1.855762963	1.34E-07	GINS2
1033	205123_s_at	-1.855077778	1.73E-08	MSANTD3-TMEFF1 /// TMEFF1
1034	228298_at	-1.85357037	1.12E-08	PCED1B
1035	206220_s_at	-1.853562963	3.17E-07	RASA3
1036	201469_s_at	-1.853440741	2.20E-10	SHC1
1037	223322_at	-1.853296296	1.57E-13	RASSF5
1038	211121_s_at	-1.852796296	7.83E-09	DOK1
1039	213891_s_at	-1.851511111	2.61E-12	TCF4
1040	238600_at	-1.849325926	1.08E-06	JAKMIP1
1041	240055_at	-1.848503704	2.76E-06	
1042	224927_at	-1.848440741	5.96E-15	PPP1R18
1043	219588_s_at	-1.847596296	9.01E-11	NCAPG2
1044	234306_s_at	-1.845307407	3.73E-06	SLAMF7
1045	213193_x_at	-1.845007407	1.76E-09	TRBC1
1046	211795_s_at	-1.843218519	6.97E-12	FYB
1047	205038_at	-1.842803704	1.06E-10	IKZF1
1048	207795_s_at	-1.841940741	2.06E-07	KLRD1
1049	238790_at	-1.841903704	9.94E-08	LOC374443
1050	202411_at	-1.841859259	3.79E-06	IFI27
1051	203472_s_at	-1.838788889	3.39E-08	SLCO2B1
1052	201983_s_at	-1.838307407	9.54E-10	EGFR
1053	228280_at	-1.836662963	5.85E-14	ZC3HAV1L
1054	212021_s_at	-1.83522963	1.21E-08	MKI67
1055	202338_at	-1.834403704	2.82E-06	TK1
1056	210786_s_at	-1.831203704	9.28E-11	FLI1
1057	215719_x_at	-1.831044444	1.11E-11	FAS
1058	204439_at	-1.830844444	9.39E-08	IFI44L
1059	240407_at	-1.830633333	9.97E-06	LOC100126784
1060	214574_x_at	-1.827814815	1.11E-15	LST1
1061	208983_s_at	-1.826222222	4.86E-09	PECAM1
1062	201063_at	-1.820844444	2.74E-14	RCN1
1063	235229_at	-1.820348148	7.24E-06	

1064	201847_at	-1.819677778	2.11E-16	LIPA
1065	212386_at	-1.819244444	4.92E-12	TCF4
1066	214866_at	-1.819151852	2.52E-06	PLAUR
1067	214481_at	-1.8191	6.08E-06	HIST1H2AM
1068	214995_s_at	-1.818725926	2.79E-13	APOBEC3F /// APOBEC3G
1069	213007_at	-1.817744444	5.54E-13	FANCI
1070	221730_at	-1.817455556	2.44E-06	COL5A2
1071	211366_x_at	-1.817340741	2.33E-14	CASP1
1072	210164_at	-1.81667037	6.58E-10	GZMB
1073	218723_s_at	-1.814518519	2.59E-07	RGCC
1074	208130_s_at	-1.814137037	2.49E-11	TBXAS1
1075	208894_at	-1.8135	4.09E-16	HLA-DRA
1076	226932_at	-1.813381481	1.72E-12	SSPN
1077	213256_at	-1.812985185	3.21E-08	3-Mar
1078	205122_at	-1.811788889	1.10E-07	MSANTD3-TMEFF1 /// TMEFF1
1079	211101_x_at	-1.80687037	3.13E-08	LILRA2
1080	236249_at	-1.80622963	2.00E-12	IKBIP
1081	204428_s_at	-1.805618519	9.57E-06	LCAT
1082	209803_s_at	-1.805022222	7.69E-08	PHLDA2
1083	207173_x_at	-1.804525926	6.55E-08	CDH11
1084	204281_at	-1.804451852	5.62E-08	TEAD4
1085	201251_at	-1.804262963	5.18E-13	PKM
1086	203022_at	-1.804259259	3.21E-13	RNASEH2A
1087	203591_s_at	-1.803618519	7.56E-09	CSF3R
1088	227112_at	-1.802777778	1.67E-10	TMCC1
1089	219134_at	-1.801555556	6.68E-09	ELTD1
1090	204284_at	-1.799925926	9.98E-10	PPP1R3C
1091	204702_s_at	-1.79977037	6.45E-13	NFE2L3
1092	228127_at	-1.798481481	1.57E-06	KCNK3
1093	221591_s_at	-1.797625926	6.81E-09	FAM64A
1094	202107_s_at	-1.7971	6.10E-11	MCM2
1095	212724_at	-1.796855556	1.32E-11	RND3
1096	236480_at	-1.792392593	2.61E-09	MIR210HG
1097	209621_s_at	-1.790192593	4.86E-08	PDLIM3
1098	219736_at	-1.790118519	3.03E-07	TRIM36
1099	211272_s_at	-1.788414815	1.21E-07	DGKA
1100	213733_at	-1.786440741	2.13E-12	MYO1F
1101	218832_x_at	-1.786155556	8.96E-06	ARRB1
1102	207543_s_at	-1.786055556	7.74E-10	P4HA1
1103	209250_at	-1.785648148	8.12E-16	DEGS1
1104	203510_at	-1.784714815	2.11E-14	MET
1105	213293_s_at	-1.784611111	3.07E-12	TRIM22
1106	207566_at	-1.784125926	3.22E-08	MR1

1107	204319_s_at	-1.781907407	9.07E-15	RGS10
1108	211434_s_at	-1.781637037	5.36E-09	CCRL2
1109	1555938_x_at	-1.781537037	1.26E-08	VIM
1110	222592_s_at	-1.781237037	1.61E-09	ACSL5
1111	236539_at	-1.779644444	1.44E-06	PTPN22
1112	226218_at	-1.779214815	5.10E-06	IL7R
1113	223158_s_at	-1.778885185	1.82E-13	NEK6
1114	201923_at	-1.778659259	1.91E-18	PRDX4
1115	225707_at	-1.777807407	4.56E-14	ARL6IP6
1116	210784_x_at	-1.776444444	2.14E-10	LILRB3
1117	1567105_at	-1.775633333	3.99E-07	
1118	211661_x_at	-1.775359259	6.05E-09	PTAFR
1119	238327_at	-1.774903704	1.48E-11	ODF3B
1120	211981_at	-1.773811111	1.62E-10	COL4A1
1121	1554676_at	-1.7713	1.80E-09	SRGN
1122	223204_at	-1.769133333	3.29E-10	FAM198B
1123	229686_at	-1.768185185	1.04E-09	P2RY8
1124	211804_s_at	-1.767622222	7.65E-07	CDK2
1125	205965_at	-1.767488889	2.56E-10	BATF
1126	225904_at	-1.766144444	1.66E-10	CCSAP
1127	200859_x_at	-1.765762963	5.18E-11	FLNA
1128	221799_at	-1.765503704	2.72E-10	CHPF2
1129	222952_s_at	-1.765025926	2.50E-08	TLR7
1130	207595_s_at	-1.765014815	1.88E-09	BMP1
1131	209806_at	-1.764707407	8.70E-09	HIST1H2BK
1132	227697_at	-1.764507407	1.41E-06	SOCS3
1133	204781_s_at	-1.764292593	6.68E-13	FAS
1134	1558046_x_at	-1.763762963	8.15E-07	LOC389906
1135	228080_at	-1.763311111	1.18E-07	LAYN
1136	238462_at	-1.762859259	2.11E-06	UBASH3B
1137	238622_at	-1.761574074	2.35E-08	RAP2B
1138	226789_at	-1.761359259	2.87E-11	EMB
1139	202274_at	-1.759548148	4.82E-07	ACTG2
1140	201188_s_at	-1.759481481	8.61E-10	ITPR3
1141	216901_s_at	-1.759337037	1.71E-06	IKZF1
1142	206553_at	-1.7586	9.21E-08	OAS2
1143	213415_at	-1.756577778	8.85E-13	CLIC2
1144	237623_at	-1.755855556	4.32E-08	CST3
1145	206574_s_at	-1.754755556	1.48E-09	PTP4A3
1146	1554835_a_at	-1.754692593	4.32E-06	B3GNT5
1147	211573_x_at	-1.7543	1.27E-07	TGM2
1148	206221_at	-1.753374074	2.02E-06	RASA3
1149	224916_at	-1.751762963	5.07E-14	TMEM173

1150	220122_at	-1.75142963	7.68E-07	MCTP1
1151	212501_at	-1.750666667	4.22E-12	CEBPB
1152	209086_x_at	-1.750618519	1.65E-11	MCAM /// MIR6756
1153	219367_s_at	-1.750277778	9.90E-07	NRP2
1154	1554408_a_at	-1.749466667	1.78E-09	TK1
1155	1552670_a_at	-1.748703704	5.30E-14	PPP1R3B
1156	AFFX-	-1.746348148	8.13E-10	STAT1
1157	211336_x_at	-1.746322222	3.58E-09	LILRB1
1158	201141_at	-1.745396296	5.54E-08	GPNMB
1159	221565_s_at	-1.745366667	5.28E-12	CALHM2
1160	200756_x_at	-1.745318519	1.78E-10	CALU
1161	235266_at	-1.74442963	1.91E-06	ATAD2
1162	201663_s_at	-1.743825926	1.43E-12	SMC4
1163	224915_x_at	-1.741914815	2.53E-14	ZFAS1
1164	201161_s_at	-1.741862963	5.67E-15	YBX3
1165	204806_x_at	-1.738859259	5.30E-14	HLA-F
1166	210225_x_at	-1.738825926	3.09E-11	LILRB3
1167	31845_at	-1.738659259	2.11E-16	ELF4
1168	208079_s_at	-1.737422222	5.98E-08	AURKA
1169	231094_s_at	-1.735533333	4.90E-09	LOC100996643 /// LOC101928195 /// MTHFD1L
1170	222062_at	-1.735488889	2.82E-08	IL27RA
1171	217763_s_at	-1.735218519	6.11E-15	RAB31
1172	212737_at	-1.733014815	1.22E-14	GM2A
1173	1563719_a_at	-1.732388889	4.14E-06	
1174	225481_at	-1.730574074	1.55E-08	FRMD6
1175	225763_at	-1.730311111	1.92E-15	RCSD1
1176	231788_at	-1.729785185	3.36E-06	LPAR5
1177	205159_at	-1.7294	2.03E-13	CSF2RB
1178	223276_at	-1.728266667	3.64E-08	SMIM3
1179	221258_s_at	-1.725288889	1.29E-06	KIF18A
1180	204285_s_at	-1.724074074	9.40E-06	PMAIP1
1181	202748_at	-1.721537037	1.39E-10	GBP2
1182	207075_at	-1.720944444	2.61E-06	NLRP3
1183	201012_at	-1.719881481	1.82E-09	ANXA1
1184	204033_at	-1.719881481	5.48E-07	TRIP13
1185	216237_s_at	-1.719340741	7.52E-17	MCM5
1186	200953_s_at	-1.716892593	4.53E-12	CCND2
1187	201422_at	-1.715925926	7.05E-11	IFI30 /// PIK3R2
1188	205579_at	-1.715837037	4.77E-08	HRH1
1189	224374_s_at	-1.712477778	2.21E-12	EMILIN2
1190	218498_s_at	-1.710362963	4.58E-11	ERO1L
1191	209496_at	-1.709874074	1.20E-06	RARRES2
1192	228258_at	-1.708781481	2.27E-07	TBC1D10C

1193	210294_at	-1.708696296	2.35E-07	TAPBP
1194	211990_at	-1.707855556	3.20E-15	HLA-DPA1
1195	220840_s_at	-1.705622222	3.66E-07	C1orf112
1196	209439_s_at	-1.704922222	4.28E-10	PHKA2
1197	219387_at	-1.704681481	2.08E-11	CCDC88A
1198	207292_s_at	-1.703418519	2.25E-08	MAPK7
1199	204116_at	-1.702	8.72E-11	IL2RG
1200	227337_at	-1.698714815	9.71E-12	ANKRD37
1201	201249_at	-1.698437037	4.28E-06	SLC2A1
1202	223525_at	-1.698396296	3.08E-06	DLL4
1203	224240_s_at	-1.696718519	4.69E-07	CCL28
1204	228837_at	-1.695518519	6.70E-07	TCF4
1205	238469_at	-1.694796296	1.49E-12	OGFRL1
1206	211528_x_at	-1.694340741	5.48E-16	HLA-G
1207	202158_s_at	-1.694259259	8.29E-10	CELF2
1208	224999_at	-1.69332963	3.14E-09	EGFR
1209	238480_at	-1.692685185	1.02E-07	TTC39C
1210	213262_at	-1.692288889	6.06E-15	SACS
1211	205574_x_at	-1.69	2.16E-08	BMP1
1212	224950_at	-1.68892963	3.28E-06	PTGFRN
1213	236884_at	-1.688696296	9.10E-06	RIMKLA
1214	204463_s_at	-1.686322222	5.47E-06	EDNRA
1215	203477_at	-1.686255556	2.64E-09	COL15A1
1216	206832_s_at	-1.685114815	1.65E-06	SEMA3F
1217	216915_s_at	-1.684425926	1.11E-09	PTPN12
1218	238025_at	-1.683774074	1.76E-15	MLKL
1219	202828_s_at	-1.683622222	6.38E-07	MMP14
1220	225717_at	-1.683355556	4.37E-14	KIAA1715
1221	228752_at	-1.6831	1.92E-08	EFCAB4B
1222	202685_s_at	-1.682874074	2.51E-08	AXL
1223	214710_s_at	-1.682162963	6.64E-07	CCNB1
1224	228729_at	-1.68197037	5.35E-07	CCNB1
1225	210978_s_at	-1.681944444	3.01E-11	TAGLN2
1226	1555852_at	-1.681662963	4.13E-17	PSMB8-AS1
1227	239843_at	-1.681137037	2.10E-13	RIT1
1228	218400_at	-1.680766667	1.14E-10	OAS3
1229	217478_s_at	-1.680551852	1.23E-11	HLA-DMA
1230	212141_at	-1.6803	3.44E-06	MCM4
1231	227877_at	-1.679914815	3.20E-09	ANXA2R
1232	202269_x_at	-1.679844444	2.16E-11	GBP1
1233	1555137_a_at	-1.679637037	6.17E-09	FGD6
1234	203439_s_at	-1.6793	3.40E-07	STC2
1235	231726_at	-1.678992593	6.43E-10	PCDHB14

1236	232543_x_at	-1.677662963	9.45E-14	ARHGAP9
1237	214181_x_at	-1.676677778	2.00E-12	LST1
1238	203104_at	-1.676281481	3.16E-15	CSF1R
1239	221039_s_at	-1.675948148	2.17E-15	ASAP1
1240	205824_at	-1.675111111	1.82E-06	HSPB2
1241	219202_at	-1.675044444	5.09E-08	RHBDF2
1242	206978_at	-1.67502963	3.13E-09	CCR2
1243	205016_at	-1.674807407	1.64E-09	TGFA
1244	223487_x_at	-1.674625926	6.23E-07	GNB4
1245	204803_s_at	-1.674488889	1.09E-06	RRAD
1246	214097_at	-1.674433333	1.51E-09	RPS21
1247	209670_at	-1.673603704	6.20E-09	TRAC
1248	219479_at	-1.672044444	2.89E-11	KDELC1
1249	1556102_x_at	-1.672040741	7.66E-07	LOC389906
1250	225710_at	-1.671881481	3.15E-14	GNB4
1251	238996_x_at	-1.670877778	6.34E-15	ALDOA
1252	202644_s_at	-1.670455556	1.98E-14	TNFAIP3
1253	204197_s_at	-1.669414815	1.06E-13	RUNX3
1254	216360_x_at	-1.667714815	1.10E-06	RRP12
1255	217762_s_at	-1.667659259	6.84E-16	RAB31
1256	214727_at	-1.667085185	2.21E-06	BRCA2
1257	202949_s_at	-1.66677037	5.74E-08	FHL2
1258	227844_at	-1.666225926	1.76E-10	FMNL3
1259	210606_x_at	-1.665618519	3.97E-06	KLRD1
1260	201655_s_at	-1.665503704	1.47E-09	HSPG2
1261	200665_s_at	-1.664181481	1.71E-13	SPARC
1262	205379_at	-1.664174074	2.97E-07	CBR3
1263	205786_s_at	-1.66407037	2.49E-11	ITGAM
1264	209122_at	-1.664040741	7.60E-08	PLIN2
1265	214701_s_at	-1.663433333	2.81E-06	FN1
1266	226055_at	-1.663274074	2.24E-11	ARRDC2
1267	239730_at	-1.661418519	3.76E-07	DGCR14 /// TSSK2
1268	228869_at	-1.661092593	5.80E-13	SNX20
1269	223454_at	-1.660981481	2.86E-13	CXCL16
1270	214054_at	-1.660307407	2.86E-08	DOK2
1271	206034_at	-1.6591	2.54E-09	SERPINB8
1272	205774_at	-1.6584	4.09E-07	F12
1273	217310_s_at	-1.657759259	5.37E-06	FOXJ3
1274	212292_at	-1.657666667	2.37E-09	SLC7A1
1275	201189_s_at	-1.657144444	1.90E-09	ITPR3
1276	219402_s_at	-1.656333333	5.18E-14	DERL1
1277	224580_at	-1.65622963	8.00E-12	SLC38A1
1278	211822_s_at	-1.655788889	5.43E-06	NLRP1

1279	201426_s_at	-1.655777778	1.10E-16	VIM
1280	224833_at	-1.654992593	7.72E-13	ETS1
1281	205909_at	-1.654525926	2.27E-09	POLE2
1282	210113_s_at	-1.653251852	9.67E-06	NLRP1
1283	235199_at	-1.65242963	1.84E-09	RNF125
1284	210042_s_at	-1.65207037	1.05E-11	CTSZ
1285	219569_s_at	-1.651118519	2.68E-10	SLC35G2
1286	205483_s_at	-1.650385185	3.72E-08	ISG15
1287	206039_at	-1.65027037	2.64E-09	RAB33A
1288	209199_s_at	-1.646648148	1.74E-12	MEF2C
1289	239431_at	-1.646544444	4.02E-10	TICAM2 /// TMED7-TICAM2
1290	235626_at	-1.646022222	3.95E-10	CAMK1D
1291	1552411_at	-1.643214815	1.37E-07	DEFB106A /// DEFB106B
1292	202157_s_at	-1.642722222	7.90E-12	CELF2
1293	204166_at	-1.642659259	7.04E-07	SBNO2
1294	44783_s_at	-1.642188889	9.90E-10	HEY1
1295	215708_s_at	-1.640525926	9.27E-07	PRIM2 /// PRIM2B
1296	213192_at	-1.640385185	3.08E-08	THAP3
1297	235948_at	-1.6397	2.64E-07	RIMKLA
1298	212382_at	-1.639485185	1.26E-09	TCF4
1299	210174_at	-1.638740741	1.02E-06	NR5A2
1300	207877_s_at	-1.638492593	8.97E-09	NVL
1301	203332_s_at	-1.638062963	7.46E-11	INPP5D
1302	1555830_s_at	-1.63557037	5.43E-08	ESYT2
1303	219243_at	-1.635137037	5.02E-11	GIMAP4
1304	207111_at	-1.635122222	2.91E-07	EMR1
1305	211529_x_at	-1.634325926	1.17E-14	HLA-G
1306	219494_at	-1.634148148	7.60E-07	RAD54B
1307	206788_s_at	-1.633374074	1.69E-11	CBFB
1308	221875_x_at	-1.633359259	8.55E-16	HLA-F
1309	207270_x_at	-1.633140741	3.79E-07	CD300C
1310	222557_at	-1.632992593	1.55E-08	STMN3
1311	215193_x_at	-1.631981481	1.27E-13	HLA-DQB1 /// HLA-DRB1 /// HLA-DRB3 /// HLA-DRB4 /// LOC100996809 /// LOC101060835
1312	211540_s_at	-1.631592593	5.88E-06	RB1
1313	229711_s_at	-1.630766667	4.73E-13	MDM2
1314	207824_s_at	-1.630492593	2.77E-07	MAZ
1315	208296_x_at	-1.629444444	4.34E-12	TNFAIP8
1316	211258_s_at	-1.629422222	6.32E-08	TGFA
1317	203726_s_at	-1.629155556	4.92E-06	LAMA3
1318	217739_s_at	-1.628948148	1.07E-09	NAMPT
1319	226136_at	-1.628885185	1.25E-11	GLIPR1

1320	203490_at	-1.6283	3.83E-06	ELF4
1321	209566_at	-1.628285185	7.18E-16	INSIG2
1322	209610_s_at	-1.626325926	9.63E-15	SLC1A4
1323	204668_at	-1.626274074	3.63E-06	RNF24
1324	227791_at	-1.626122222	7.43E-08	SLC9A9
1325	227966_s_at	-1.624181481	7.69E-06	CCDC74A /// CCDC74B
1326	206078_at	-1.622525926	2.92E-06	KALRN
1327	204821_at	-1.620848148	3.07E-13	BTN3A3
1328	206632_s_at	-1.61997037	2.54E-09	APOBEC3B
1329	212067_s_at	-1.619337037	4.31E-08	C1R
1330	209921_at	-1.618948148	3.96E-06	SLC7A11
1331	209118_s_at	-1.617396296	4.46E-12	TUBA1A
1332	236275_at	-1.616866667	5.83E-07	KRBA1
1333	224341_x_at	-1.615425926	1.14E-09	TLR4
1334	226474_at	-1.614277778	1.87E-11	NLRC5
1335	213351_s_at	-1.614018519	2.27E-08	TMCC1
1336	223229_at	-1.613785185	2.55E-08	UBE2T
1337	203320_at	-1.613137037	1.38E-14	SH2B3
1338	219994_at	-1.612477778	1.32E-06	APBB1IP
1339	231887_s_at	-1.612337037	1.04E-09	PALD1
1340	219762_s_at	-1.611662963	8.24E-16	RPL36
1341	228207_at	-1.610403704	3.50E-06	LOC100499489
1342	202240_at	-1.608922222	9.42E-06	PLK1
1343	219684_at	-1.608751852	1.32E-08	RTP4
1344	208636_at	-1.607207407	2.03E-12	ACTN1
1345	206111_at	-1.607022222	1.10E-10	RNASE2
1346	242625_at	-1.606037037	7.14E-07	RSAD2
1347	1560156_at	-1.605066667	1.05E-07	LOC101928054
1348	1553997_a_at	-1.604225926	6.69E-08	ASPHD1
1349	223489_x_at	-1.603855556	3.81E-08	EXOSC3
1350	1553530_a_at	-1.602781481	2.93E-10	ITGB1
1351	227289_at	-1.602588889	5.71E-08	PCDH17
1352	210845_s_at	-1.600048148	3.58E-07	PLAUR
1353	222409_at	-1.599937037	7.51E-14	CORO1C
1354	201641_at	-1.599622222	1.76E-07	BST2
1355	227801_at	-1.599222222	1.83E-09	TRIM59
1356	1552422_at	-1.598807407	6.71E-08	C10orf25
1357	224841_x_at	-1.598333333	2.86E-11	GAS5 /// SNORD44 /// SNORD47 /// SNORD74 /// SNORD76 /// SNORD77 /// SNORD79 /// SNORD80 /// SNORD81
1358	230499_at	-1.598292593	6.06E-10	BIRC3
1359	208097_s_at	-1.598155556	1.07E-08	TMX1
1360	225612_s_at	-1.597548148	6.79E-12	B3GNT5

1361	227271_at	-1.595725926	8.86E-06	FGF11
1362	203044_at	-1.593407407	3.13E-11	CHSY1
1363	1560579_s_at	-1.592255556	1.27E-06	SLC16A1-AS1
1364	205808_at	-1.592177778	3.40E-08	ASPH
1365	210514_x_at	-1.591337037	1.76E-11	HLA-G
1366	221529_s_at	-1.590703704	2.25E-06	PLVAP
1367	219118_at	-1.590681481	4.57E-10	FKBP11
1368	226702_at	-1.590388889	2.76E-09	CMPK2
1369	204887_s_at	-1.589014815	2.78E-07	PLK4
1370	226810_at	-1.58712963	9.03E-14	OGFRL1
1371	205091_x_at	-1.586462963	1.30E-14	RECQL
1372	222235_s_at	-1.585892593	3.33E-09	CSGALNACT2
1373	204057_at	-1.584937037	1.28E-11	IRF8
1374	218181_s_at	-1.582196296	3.59E-15	MAP4K4
1375	211825_s_at	-1.581903704	3.94E-07	FLI1
1376	209308_s_at	-1.581696296	9.37E-13	BNIP2
1377	208981_at	-1.580481481	6.48E-09	PECAM1
1378	224909_s_at	-1.580288889	6.54E-14	PREX1
1379	226227_x_at	-1.580218519	1.89E-13	ZFAS1
1380	203508_at	-1.580214815	9.44E-12	TNFRSF1B
1381	206150_at	-1.579937037	7.08E-06	CD27
1382	226936_at	-1.579596296	9.17E-09	CENPW
1383	205153_s_at	-1.577577778	4.40E-13	CD40
1384	1566633_at	-1.577011111	5.55E-06	
1385	209685_s_at	-1.576896296	1.00E-11	PRKCB
1386	239798_at	-1.57667037	6.79E-07	PDK1
1387	202156_s_at	-1.576074074	5.35E-07	CELF2
1388	1557557_at	-1.575925926	4.47E-06	MATN1-AS1
1389	205937_at	-1.575659259	4.04E-06	CGREF1
1390	230640_at	-1.575522222	1.47E-09	FMNL3
1391	208690_s_at	-1.575222222	7.27E-12	PDLIM1
1392	235292_at	-1.573681481	4.07E-08	FLJ32255
1393	211841_s_at	-1.573588889	6.22E-06	TNFRSF25
1394	242288_s_at	-1.57327037	3.74E-06	EMILIN2
1395	227963_at	-1.572848148	5.13E-06	
1396	208729_x_at	-1.572614815	3.99E-16	HLA-B
1397	208789_at	-1.572577778	1.81E-13	PTRF
1398	224741_x_at	-1.571337037	2.74E-11	GAS5 /// SNORD44 /// SNORD47 /// SNORD74 /// SNORD76 /// SNORD77 /// SNORD79 /// SNORD80 /// SNORD81
1399	210089_s_at	-1.571088889	3.52E-07	LAMA4
1400	223274_at	-1.569548148	2.39E-07	TCF19
1401	AFFX-	-1.569455556	4.23E-07	STAT1

1402	203696_s_at	-1.569218519	2.02E-13	RFC2
1403	225604_s_at	-1.56877037	2.71E-11	GLIPR2
1404	223519_at	-1.567240741	4.58E-12	ZAK
1405	219471_at	-1.5668	2.44E-09	KIAA0226L
1406	210004_at	-1.566740741	6.22E-08	OLR1
1407	212013_at	-1.566077778	6.21E-08	PXDN
1408	239388_at	-1.565466667	2.91E-08	RNASET2
1409	227779_at	-1.564274074	5.14E-07	ECSCR
1410	1553432_s_at	-1.563825926	8.36E-06	LOC101060424 /// LOC653786 /// OTOA
1411	228442_at	-1.56232963	3.07E-08	NFATC2
1412	210176_at	-1.559825926	4.60E-12	TLR1
1413	209641_s_at	-1.559488889	2.22E-06	ABCC3
1414	201272_at	-1.558940741	6.08E-08	AKR1B1
1415	205248_at	-1.558603704	2.38E-07	DOPEY2
1416	203302_at	-1.558140741	6.91E-16	DCK
1417	235476_at	-1.556940741	7.60E-08	IFT80 /// TRIM59
1418	206756_at	-1.556644444	1.42E-06	CHST7
1419	221561_at	-1.555507407	6.92E-10	SOAT1
1420	203300_x_at	-1.555444444	1.18E-13	AP1S2
1421	224964_s_at	-1.554637037	2.90E-11	GNG2
1422	209295_at	-1.5546	4.46E-14	TNFRSF10B
1423	236345_at	-1.552703704	1.44E-07	TBXAS1
1424	208161_s_at	-1.552407407	5.27E-06	ABCC3
1425	220301_at	-1.551792593	1.22E-07	CCDC102B
1426	207431_s_at	-1.551255556	5.47E-12	DEGS1
1427	223640_at	-1.551077778	3.75E-13	HCST
1428	207428_x_at	-1.550988889	3.86E-06	CDK11A /// CDK11B
1429	225056_at	-1.549459259	1.67E-09	SIPA1L2
1430	200821_at	-1.549140741	2.48E-14	LAMP2
1431	222217_s_at	-1.549111111	5.31E-13	SLC27A3
1432	211911_x_at	-1.5488	2.24E-14	HLA-B
1433	210732_s_at	-1.548374074	3.46E-07	LGALS8
1434	201664_at	-1.548077778	2.03E-12	SMC4
1435	238949_at	-1.546685185	5.72E-13	RNF145
1436	233852_at	-1.544759259	1.01E-08	POLH
1437	202679_at	-1.544425926	1.40E-10	NPC1
1438	1564494_s_at	-1.544114815	4.92E-11	P4HB
1439	33646_g_at	-1.543814815	6.48E-09	GM2A
1440	213004_at	-1.543414815	4.66E-10	ANGPTL2
1441	226077_at	-1.542940741	4.01E-18	RNF145
1442	209321_s_at	-1.542462963	7.89E-14	ADCY3
1443	238022_at	-1.542251852	6.27E-08	CRNDE
1444	202766_s_at	-1.541055556	4.04E-06	FBN1

1445	209448_at	-1.541040741	1.10E-11	HTATIP2
1446	215215_s_at	-1.540837037	9.07E-08	LOC81691
1447	205214_at	-1.540651852	1.23E-07	STK17B
1448	204780_s_at	-1.538477778	1.16E-12	FAS
1449	211340_s_at	-1.538388889	1.57E-11	MCAM /// MIR6756
1450	217985_s_at	-1.538351852	5.00E-13	BAZ1A
1451	224610_at	-1.537903704	1.09E-12	SNHG1 /// SNORD22 /// SNORD25 /// SNORD26 /// SNORD27 /// SNORD28 /// SNORD29 /// SNORD30 /// SNORD31
1452	203964_at	-1.537840741	1.03E-15	NMI
1453	1555326_a_at	-1.537092593	1.47E-07	ADAM9
1454	228333_at	-1.535203704	7.02E-14	ZEB2
1455	202375_at	-1.53302963	2.56E-10	SEC24D
1456	211725_s_at	-1.532966667	9.07E-15	BID
1457	202236_s_at	-1.532955556	1.73E-06	SLC16A1
1458	213830_at	-1.532114815	9.83E-06	YME1L1
1459	201540_at	-1.532011111	6.18E-07	FHL1
1460	206090_s_at	-1.532011111	3.92E-09	DISC1 /// TSNAX-DISC1
1461	204493_at	-1.53062963	4.99E-13	BID
1462	205821_at	-1.52997037	8.94E-08	KLRC4-KLRK1 /// KLRK1
1463	238846_at	-1.528785185	9.65E-10	TNFRSF11A
1464	218662_s_at	-1.528685185	1.39E-06	NCAPG
1465	1552921_a_at	-1.527711111	6.01E-10	FIGNL1
1466	228141_at	-1.525407407	1.77E-08	GPX8
1467	214532_x_at	-1.525066667	3.78E-06	POU5F1B
1468	201755_at	-1.524896296	2.48E-08	MCM5
1469	200916_at	-1.520077778	2.01E-12	TAGLN2
1470	213001_at	-1.519759259	1.02E-08	ANGPTL2
1471	207719_x_at	-1.518803704	7.51E-14	CEP170 /// CEP170P1
1472	222895_s_at	-1.518640741	5.11E-07	BCL11B
1473	205119_s_at	-1.517814815	1.33E-09	FPR1
1474	204820_s_at	-1.516344444	2.51E-12	BTN3A2 /// BTN3A3
1475	219359_at	-1.515618519	1.11E-06	ATHL1
1476	212856_at	-1.51522963	1.21E-10	GRAMD4
1477	1555797_a_at	-1.511203704	2.49E-12	ARPC5
1478	203535_at	-1.511062963	7.94E-07	S100A9
1479	201719_s_at	-1.510851852	7.51E-14	EPB41L2
1480	214453_s_at	-1.509737037	7.46E-10	IFI44
1481	233337_s_at	-1.508974074	1.45E-06	SEZ6L2
1482	228069_at	-1.508718519	1.38E-08	MTFR2
1483	201401_s_at	-1.508366667	5.85E-08	ADRBK1
1484	205656_at	-1.507611111	2.65E-07	PCDH17
1485	200648_s_at	-1.507333333	1.34E-09	GLUL

1486	1554271_a_at	-1.507188889	6.92E-10	CENPL
1487	217764_s_at	-1.506403704	4.29E-15	RAB31
1488	239587_at	-1.506018519	9.66E-08	TLR3
1489	200757_s_at	-1.50592963	1.35E-11	CALU
1490	215933_s_at	-1.505859259	1.55E-09	HHEX
1491	202651_at	-1.503751852	8.57E-12	LPGAT1
1492	226382_at	-1.503455556	9.26E-10	LOC283070
1493	212850_s_at	-1.503103704	1.08E-06	LRP4
1494	227125_at	-1.502225926	1.33E-13	IFNAR2
1495	208540_x_at	-1.500392593	1.23E-10	S100A11P1 /// S100A11P1
1496	204972_at	-1.500288889	7.61E-11	OAS2
1497	211113_s_at	-1.499911111	2.57E-10	ABCG1
1498	219787_s_at	-1.4998	6.95E-08	ECT2
1499	223767_at	-1.499744444	6.89E-06	GPR84
1500	219403_s_at	-1.499033333	6.71E-07	HPSE
1501	242521_at	-1.49892963	2.27E-12	LOC100505812
1502	200841_s_at	-1.498359259	5.04E-09	EPRS
1503	1554126_at	-1.498244444	1.89E-07	MSRB3
1504	222846_at	-1.497259259	3.09E-09	RAB8B
1505	204725_s_at	-1.495792593	2.82E-13	NCK1
1506	227897_at	-1.49572963	9.00E-11	
1507	219441_s_at	-1.495659259	2.05E-09	LRRK1
1508	241386_at	-1.494592593	1.95E-07	LOC100506691
1509	204164_at	-1.493748148	4.66E-13	SIPA1
1510	218172_s_at	-1.493718519	8.05E-14	DERL1
1511	235609_at	-1.493322222	1.66E-06	BRIP1
1512	210152_at	-1.492974074	1.64E-10	LILRB4
1513	202878_s_at	-1.49272963	4.33E-10	CD93
1514	210337_s_at	-1.492281481	9.48E-11	ACLY
1515	229553_at	-1.49187037	2.23E-11	PGM2L1
1516	206082_at	-1.490844444	4.33E-09	HCP5
1517	232792_at	-1.490333333	5.16E-06	TRIM69
1518	203140_at	-1.49007037	6.35E-12	BCL6
1519	202786_at	-1.489403704	2.24E-08	STK39
1520	242843_at	-1.489351852	9.71E-09	BCAN
1521	203484_at	-1.488622222	2.02E-13	SEC61G
1522	225800_at	-1.488588889	3.23E-06	JAZF1
1523	217744_s_at	-1.488222222	1.61E-08	PERP
1524	221293_s_at	-1.487177778	4.19E-06	DEF6
1525	205443_at	-1.486892593	2.65E-08	SNAPC1
1526	203186_s_at	-1.486766667	3.26E-10	S100A4
1527	204007_at	-1.486655556	6.91E-06	FCGR3B
1528	212689_s_at	-1.486281481	1.08E-15	KDM3A

1529	210463_x_at	-1.486144444	5.50E-09	TRMT1
1530	212680_x_at	-1.48567037	4.19E-08	PPP1R14B
1531	1553678_a_at	-1.485585185	7.18E-11	ITGB1
1532	1560916_a_at	-1.485281481	5.85E-09	DPY19L1
1533	213746_s_at	-1.484125926	1.44E-09	FLNA
1534	203702_s_at	-1.484011111	8.88E-06	TTLL4
1535	201301_s_at	-1.483803704	9.86E-10	ANXA4
1536	203276_at	-1.483774074	6.93E-11	LMNB1
1537	220088_at	-1.483722222	1.14E-06	C5AR1
1538	211966_at	-1.483492593	2.23E-09	COL4A2
1539	214791_at	-1.483366667	3.62E-12	SP140L
1540	217677_at	-1.483122222	2.82E-10	PLEKHA2
1541	217599_s_at	-1.482888889	3.43E-10	MDFIC
1542	203857_s_at	-1.4818	9.07E-11	MIR7110 /// PDIA5
1543	222077_s_at	-1.481796296	5.74E-10	RACGAP1
1544	1554390_s_at	-1.481659259	6.87E-09	ACTR2
1545	240890_at	-1.479803704	1.17E-08	LOC643733
1546	210629_x_at	-1.479677778	1.89E-12	LST1
1547	224576_at	-1.479151852	5.23E-11	ERGIC1
1548	221676_s_at	-1.4789	2.84E-11	CORO1C
1549	214992_s_at	-1.478833333	2.07E-11	DNASE2
1550	225415_at	-1.47842963	3.33E-15	DTX3L
1551	227526_at	-1.476107407	4.44E-08	CDON
1552	244434_at	-1.475425926	4.21E-06	GPR82
1553	213923_at	-1.475418519	7.16E-13	RAP2B
1554	204833_at	-1.475233333	1.37E-10	ATG12
1555	213466_at	-1.474562963	5.13E-06	RAB40C
1556	226430_at	-1.473659259	7.21E-15	RELL1
1557	221269_s_at	-1.473233333	6.98E-12	SH3BGRL3
1558	208712_at	-1.47252963	2.31E-07	CCND1
1559	205580_s_at	-1.472207407	2.11E-06	HRH1
1560	223854_at	-1.471985185	4.13E-07	PCDHB10 /// PCDHB9
1561	206289_at	-1.471866667	2.50E-07	HOXA4
1562	224791_at	-1.471474074	6.12E-15	ASAP1
1563	205456_at	-1.471362963	8.60E-07	CD3E
1564	222879_s_at	-1.469944444	7.00E-06	POLH
1565	227266_s_at	-1.469944444	1.45E-10	FYB
1566	222843_at	-1.469007407	5.05E-15	FIGNL1
1567	208579_x_at	-1.46882963	2.63E-08	H2BFS
1568	226368_at	-1.46827037	2.61E-13	CHST11
1569	208646_at	-1.466848148	2.44E-09	
1570	1558738_at	-1.46587037	7.72E-09	NOL3
1571	202096_s_at	-1.465740741	4.61E-13	TSPO

1572	211602_s_at	-1.464696296	2.86E-07	TRPC1
1573	223751_x_at	-1.464551852	2.75E-06	TLR10
1574	223451_s_at	-1.464518519	1.36E-14	CKLF
1575	207668_x_at	-1.464059259	2.83E-12	PDIA6
1576	216952_s_at	-1.46122963	3.58E-06	LMNB2
1577	205349_at	-1.46057037	1.99E-09	GNA15
1578	210087_s_at	-1.459766667	6.87E-10	MPZL1
1579	203293_s_at	-1.458918519	3.40E-06	LMAN1
1580	205393_s_at	-1.457840741	7.90E-07	CHEK1
1581	213358_at	-1.457088889	1.40E-06	MTCL1
1582	213797_at	-1.456796296	1.38E-06	RSAD2
1583	1554466_a_at	-1.456344444	3.04E-10	C16orf13
1584	218991_at	-1.4563	1.43E-07	HEATR6
1585	1557938_s_at	-1.455966667	1.56E-13	PTRF
1586	212792_at	-1.455533333	1.43E-11	DPY19L1
1587	210151_s_at	-1.455451852	1.89E-08	DYRK3
1588	235252_at	-1.454885185	4.34E-08	KSR1
1589	230413_s_at	-1.454448148	4.25E-10	
1590	206236_at	-1.454437037	3.29E-07	GPR4
1591	243957_at	-1.453911111	6.49E-07	LOC100128108
1592	223879_s_at	-1.453333333	3.85E-09	OXR1
1593	224995_at	-1.452744444	2.73E-12	SPIRE1
1594	209714_s_at	-1.451911111	1.43E-06	CDKN3
1595	209408_at	-1.451874074	3.01E-07	KIF2C
1596	216252_x_at	-1.451825926	5.27E-09	FAS
1597	200784_s_at	-1.451225926	3.60E-06	LRP1
1598	223790_at	-1.450807407	2.99E-06	KATNAL1
1599	200755_s_at	-1.450625926	7.14E-11	CALU
1600	231964_at	-1.449277778	2.28E-08	BICD1
1601	244000_at	-1.448903704	2.17E-06	
1602	38671_at	-1.448881481	1.17E-12	PLXND1
1603	225750_at	-1.447762963	5.64E-12	ERO1L
1604	222548_s_at	-1.447344444	2.35E-08	MAP4K4
1605	204923_at	-1.447333333	2.70E-12	SASH3
1606	221827_at	-1.447148148	1.06E-08	RBCK1
1607	231957_s_at	-1.447003704	5.27E-06	DPP9
1608	220128_s_at	-1.446751852	5.29E-07	NIPAL2
1609	222039_at	-1.446511111	1.98E-06	KIF18B
1610	228234_at	-1.446174074	2.04E-14	TICAM2 /// TMED7-TICAM2
1611	219815_at	-1.445325926	3.09E-09	GAL3ST4
1612	227249_at	-1.444107407	2.68E-10	NDE1
1613	203087_s_at	-1.443622222	8.27E-12	KIF2A
1614	201624_at	-1.442844444	7.07E-15	DARS

1615	238423_at	-1.440096296	3.83E-07	SYTL3
1616	212917_x_at	-1.439948148	6.81E-14	RECQL
1617	222723_at	-1.439318519	9.45E-06	VWA1
1618	221691_x_at	-1.437955556	8.73E-15	NPM1
1619	200660_at	-1.437848148	2.42E-08	S100A11
1620	239619_at	-1.437514815	1.70E-07	
1621	211160_x_at	-1.437351852	8.62E-08	ACTN1
1622	228990_at	-1.436696296	1.82E-06	SNHG12 /// SNORA16A /// SNORA44 /// SNORA61
1623	229517_at	-1.43582963	3.40E-07	PTPDC1
1624	200872_at	-1.435192593	9.50E-14	S100A10
1625	200737_at	-1.435159259	5.01E-13	PGK1
1626	204464_s_at	-1.43457037	3.20E-08	EDNRA
1627	202663_at	-1.43447037	3.00E-12	WIPF1
1628	228531_at	-1.433762963	5.69E-11	SAMD9
1629	228617_at	-1.433603704	3.35E-07	XAF1
1630	231747_at	-1.432277778	5.99E-10	CYSLTR1
1631	205206_at	-1.43122963	5.16E-09	KAL1
1632	225520_at	-1.42882963	7.26E-11	LOC100996643 /// LOC101928195 /// MTHFD1L
1633	200762_at	-1.428007407	1.31E-13	DPYSL2
1634	239675_at	-1.427540741	4.35E-06	LINC00900
1635	206102_at	-1.426888889	3.10E-07	GINS1
1636	203650_at	-1.426785185	4.90E-10	PROCR
1637	219161_s_at	-1.426307407	3.08E-14	CKLF /// CKLF-CMTM1
1638	206471_s_at	-1.426	1.62E-10	PLXNC1
1639	202643_s_at	-1.425081481	9.53E-11	TNFAIP3
1640	219412_at	-1.424718519	9.37E-11	RAB38
1641	210385_s_at	-1.423862963	2.42E-10	ERAP1
1642	221078_s_at	-1.423474074	7.94E-08	CCDC88A
1643	225973_at	-1.423259259	1.04E-12	TAP2
1644	236027_at	-1.422781481	1.30E-11	SFR1
1645	203236_s_at	-1.422688889	3.48E-08	LGALS9
1646	AFFX-	-1.422274074	2.12E-08	STAT1
1647	202726_at	-1.422114815	5.29E-10	LIG1
1648	202276_at	-1.42087037	1.10E-13	SHFM1
1649	206247_at	-1.420003704	7.20E-10	MICB
1650	212020_s_at	-1.419922222	8.96E-06	MKI67
1651	223798_at	-1.418744444	3.96E-10	SLC41A2
1652	1558511_s_at	-1.417877778	1.39E-10	ESYT2
1653	205321_at	-1.417537037	4.70E-07	EIF2S3
1654	219117_s_at	-1.417288889	2.23E-09	FKBP11
1655	1555826_at	-1.416907407	3.12E-06	BIRC5 /// EPR-1
1656	201399_s_at	-1.415955556	9.55E-09	TRAM1

1657	206133_at	-1.415540741	8.78E-08	XAF1
1658	211980_at	-1.414292593	3.18E-10	COL4A1
1659	213241_at	-1.413422222	5.75E-12	PLXNC1
1660	213006_at	-1.411537037	3.61E-06	CEBPD
1661	205474_at	-1.411088889	1.78E-17	CRLF3
1662	227364_at	-1.410892593	1.27E-10	
1663	205053_at	-1.410692593	5.86E-15	PRIM1
1664	208998_at	-1.410051852	8.56E-10	UCP2
1665	217733_s_at	-1.40972963	7.50E-18	TMSB10
1666	215990_s_at	-1.409359259	2.84E-08	BCL6
1667	219047_s_at	-1.409096296	3.83E-06	ZNF668
1668	205726_at	-1.4085	5.24E-14	DIAPH2
1669	1553681_a_at	-1.40847037	3.10E-10	PRF1
1670	223095_at	-1.407944444	2.30E-10	MARVELD1
1671	224314_s_at	-1.407592593	2.33E-10	EGLN1
1672	204395_s_at	-1.406496296	3.39E-06	GRK5
1673	217986_s_at	-1.404896296	1.80E-13	BAZ1A
1674	200905_x_at	-1.403162963	7.23E-15	HLA-E
1675	231629_x_at	-1.402255556	2.92E-10	KLK3
1676	244251_at	-1.40182963	1.11E-07	LCP2
1677	201649_at	-1.400740741	3.58E-15	UBE2L6
1678	204610_s_at	-1.400696296	5.25E-11	CCDC85B
1679	243999_at	-1.400459259	1.73E-09	SLFN5
1680	208392_x_at	-1.400374074	6.39E-07	SP110
1681	1555896_a_at	-1.400292593	5.44E-08	ADAM15
1682	225979_at	-1.400244444	1.79E-06	PLEKHG2
1683	228252_at	-1.400048148	7.82E-07	PIF1
1684	230264_s_at	-1.399385185	3.24E-13	AP1S2
1685	201330_at	-1.399003704	2.72E-10	RARS
1686	209575_at	-1.398185185	8.52E-17	IL10RB
1687	45633_at	-1.396511111	3.93E-12	GINS3
1688	201529_s_at	-1.396396296	4.13E-14	RPA1
1689	204786_s_at	-1.396103704	1.11E-07	IFNAR2
1690	212012_at	-1.396022222	5.33E-08	PXDN
1691	209200_at	-1.395322222	4.02E-10	MEF2C
1692	1053_at	-1.395133333	1.96E-16	RFC2
1693	206313_at	-1.394996296	6.93E-08	HLA-DOA
1694	205462_s_at	-1.394344444	2.09E-07	HPCAL1
1695	35150_at	-1.394244444	1.06E-14	CD40
1696	202625_at	-1.394048148	5.22E-14	LYN
1697	224699_s_at	-1.394	1.08E-11	ESYT2
1698	204528_s_at	-1.391522222	7.02E-13	NAP1L1
1699	203603_s_at	-1.390081481	3.30E-12	ZEB2

1700	213002_at	-1.389092593	7.40E-14	MARCKS
1701	214895_s_at	-1.388514815	1.22E-09	ADAM10
1702	235571_at	-1.3874	3.70E-08	MIR34A
1703	217657_at	-1.387296296	7.69E-06	
1704	200869_at	-1.386477778	3.55E-17	RPL18A /// SNORA68
1705	238151_at	-1.38592963	2.10E-08	
1706	212314_at	-1.384992593	9.75E-09	SEL1L3
1707	214095_at	-1.382914815	4.10E-11	SHMT2
1708	1558397_at	-1.382655556	9.07E-06	PECAM1
1709	219924_s_at	-1.3826	1.13E-09	ZMYM6
1710	222817_at	-1.381755556	2.13E-08	HSD3B7
1711	212438_at	-1.380792593	3.63E-14	SNRNP27
1712	229597_s_at	-1.380692593	8.41E-07	WDFY4
1713	209539_at	-1.379944444	3.48E-13	ARHGEF6
1714	227167_s_at	-1.379911111	4.52E-14	RASSF3
1715	210568_s_at	-1.379811111	1.90E-11	RECQL
1716	219105_x_at	-1.379281481	1.64E-07	ORC6
1717	212387_at	-1.37847037	3.22E-09	TCF4
1718	228171_s_at	-1.378448148	1.52E-08	PLEKHG4
1719	227184_at	-1.377011111	1.67E-12	PTAFR
1720	1561937_x_at	-1.376544444	6.55E-06	IGHA1 /// IGHG1 /// IGHM /// IGHV4- 31
1721	228230_at	-1.376359259	1.06E-06	HELZ2
1722	204445_s_at	-1.375981481	1.30E-06	ALOX5
1723	203210_s_at	-1.375296296	1.39E-07	RFC5
1724	227514_at	-1.374696296	2.05E-12	ITPRIPL2
1725	238641_at	-1.374333333	1.12E-06	TMEM51-AS1
1726	38149_at	-1.374155556	1.65E-13	ARHGAP25
1727	229442_at	-1.374085185	3.49E-08	C18orf54
1728	209312_x_at	-1.373044444	1.06E-13	HLA-DQB1 /// HLA-DRB1 /// HLA-DRB4 /// HLA-DRB5 /// LOC100996809 /// LOC101060835
1729	214752_x_at	-1.37262963	1.54E-09	FLNA
1730	223544_at	-1.372533333	2.50E-06	TMEM79
1731	227954_at	-1.372503704	2.74E-11	ITPRIPL2
1732	213075_at	-1.372477778	4.42E-06	OLFML2A
1733	217294_s_at	-1.371374074	1.85E-09	ENO1
1734	212995_x_at	-1.371011111	1.24E-14	MZT2A /// MZT2B /// PHGDH
1735	211067_s_at	-1.370466667	2.42E-07	GAS7
1736	225129_at	-1.370181481	7.85E-14	CPNE2
1737	1555434_a_at	-1.3688	3.73E-06	SLC39A14
1738	225798_at	-1.368477778	1.40E-09	JAZF1
1739	225367_at	-1.368007407	2.60E-12	PGM2
1740	225414_at	-1.36792963	7.25E-16	RNF149

1741	202779_s_at	-1.366355556	6.65E-06	UBE2S
1742	225898_at	-1.366281481	1.14E-09	WDR54
1743	203233_at	-1.36627037	1.45E-15	IL4R
1744	210754_s_at	-1.366037037	1.34E-15	LYN
1745	235668_at	-1.365355556	7.78E-09	PRDM1
1746	214500_at	-1.364459259	1.35E-11	H2AFY
1747	225711_at	-1.363744444	6.55E-13	ARL6IP6
1748	204150_at	-1.363692593	2.19E-11	STAB1
1749	219423_x_at	-1.361374074	8.00E-06	TNFRSF25
1750	227378_x_at	-1.361322222	2.78E-14	C16orf13
1751	214512_s_at	-1.36047037	1.68E-12	SUB1
1752	202284_s_at	-1.360411111	2.81E-09	CDKN1A
1753	238794_at	-1.359903704	1.82E-09	SFR1
1754	238725_at	-1.358462963	1.13E-09	IRF1
1755	1558365_at	-1.358455556	4.14E-08	PGK1
1756	230165_at	-1.358337037	8.64E-09	SGOL2
1757	219502_at	-1.357922222	9.79E-06	NEIL3
1758	211671_s_at	-1.356914815	4.80E-13	NR3C1
1759	208782_at	-1.354959259	8.21E-08	FSTL1
1760	210951_x_at	-1.354588889	3.22E-09	RAB27A
1761	220494_s_at	-1.353666667	2.78E-07	
1762	222037_at	-1.352048148	5.27E-06	MCM4
1763	1554519_at	-1.351707407	2.66E-06	CD80
1764	220617_s_at	-1.351248148	1.00E-09	ZNF532
1765	237753_at	-1.35057037	7.62E-07	IL21R
1766	117_at	-1.350548148	2.74E-07	HSPA6
1767	226878_at	-1.350392593	9.02E-08	HLA-DOA
1768	204521_at	-1.349566667	1.99E-08	FAM216A
1769	212552_at	-1.3469	1.02E-07	HPCAL1
1770	202664_at	-1.34597037	5.83E-14	WIPF1
1771	38241_at	-1.345211111	2.64E-13	BTN3A3
1772	213503_x_at	-1.344677778	1.77E-11	ANXA2
1773	218848_at	-1.343785185	6.02E-09	THOC6
1774	218331_s_at	-1.343677778	1.03E-15	FAM208B
1775	206467_x_at	-1.343211111	5.59E-07	RTEL1-TNFRSF6B /// TNFRSF6B
1776	226751_at	-1.34312963	2.78E-09	CNRIP1
1777	240277_at	-1.343051852	3.57E-07	
1778	244544_at	-1.340659259	4.68E-06	
1779	224733_at	-1.340177778	1.21E-09	CMTM3
1780	206015_s_at	-1.339348148	2.63E-06	FOXJ3
1781	204531_s_at	-1.339096296	3.24E-09	BRCA1
1782	1557411_s_at	-1.339055556	4.10E-11	SLC25A43
1783	226314_at	-1.339044444	2.92E-15	CHST14

1784	231431_s_at	-1.337355556	2.00E-08	AL832909 /// LINC00984 /// LINC00984 /// LINC00984
1785	218634_at	-1.336996296	3.17E-12	PHLDA3
1786	208639_x_at	-1.336477778	8.39E-12	PDIA6
1787	221978_at	-1.336177778	5.78E-09	HLA-F
1788	217356_s_at	-1.335833333	5.14E-12	PGK1
1789	203507_at	-1.33532963	5.65E-07	CD68 /// LOC101928634 /// SNORA67
1790	202381_at	-1.335314815	7.95E-10	ADAM9
1791	204513_s_at	-1.335148148	1.50E-12	ELMO1
1792	201466_s_at	-1.33402963	4.06E-06	JUN
1793	200785_s_at	-1.333592593	3.79E-08	LRP1
1794	221423_s_at	-1.33012963	1.01E-10	YIPF5
1795	229225_at	-1.329866667	7.52E-09	NRP2
1796	202968_s_at	-1.329222222	2.10E-12	DYRK2
1797	212250_at	-1.328740741	5.09E-14	MTDH
1798	211075_s_at	-1.327340741	2.24E-12	CD47
1799	225273_at	-1.327325926	4.13E-11	WWC3
1800	200650_s_at	-1.326311111	4.20E-15	LDHA
1801	218849_s_at	-1.326296296	2.82E-14	PPP1R13L
1802	226350_at	-1.326259259	3.72E-11	CHML
1803	205552_s_at	-1.325340741	1.05E-07	OAS1
1804	211287_x_at	-1.324262963	2.61E-06	CSF2RA
1805	241733_at	-1.324177778	1.73E-06	C18orf54
1806	238013_at	-1.323866667	2.85E-08	PLEKHA2
1807	225567_at	-1.32377037	4.85E-12	AL832909 /// LINC00984
1808	219901_at	-1.322751852	2.86E-10	FGD6
1809	208754_s_at	-1.322662963	3.64E-10	NAP1L1
1810	224973_at	-1.321814815	1.02E-07	FAM46A
1811	217995_at	-1.321677778	3.81E-15	SQRDL
1812	214700_x_at	-1.32152963	4.19E-11	LOC101929336 /// RIF1
1813	221843_s_at	-1.321485185	1.41E-06	TLDC1
1814	225411_at	-1.3214	1.24E-12	TMEM87B
1815	201897_s_at	-1.321322222	1.99E-10	CKS1B
1816	1558517_s_at	-1.320377778	1.73E-09	LOC101927933 /// LRRC8C
1817	201250_s_at	-1.320066667	4.28E-07	SLC2A1
1818	225547_at	-1.319940741	2.25E-11	SNHG6 /// SNORD87
1819	202006_at	-1.319381481	2.90E-16	PTPN12
1820	1553856_s_at	-1.318337037	1.23E-06	P2RY10
1821	216250_s_at	-1.3182	2.74E-11	LPXN
1822	225136_at	-1.317988889	2.50E-14	PLEKHA2
1823	1555841_at	-1.317511111	2.17E-11	MSANTD3
1824	210594_x_at	-1.317437037	7.49E-08	MPZL1
1825	208808_s_at	-1.316318519	1.60E-11	HMGB2

1826	208790_s_at	-1.316114815	1.23E-13	PTRF
1827	204092_s_at	-1.315288889	4.37E-06	AURKA
1828	205685_at	-1.314774074	3.51E-09	CD86
1829	200697_at	-1.313711111	9.27E-12	HK1
1830	208438_s_at	-1.313414815	4.18E-08	FGR
1831	201020_at	-1.312951852	5.97E-14	YWHAH
1832	214317_x_at	-1.311759259	8.55E-13	RPS9
1833	205639_at	-1.311192593	7.47E-07	AOAH
1834	208637_x_at	-1.310507407	4.83E-10	ACTN1
1835	217027_x_at	-1.310081481	6.19E-06	AC004941.5
1836	209387_s_at	-1.309966667	2.65E-06	TM4SF1
1837	227188_at	-1.309181481	3.17E-06	EVA1C
1838	216902_s_at	-1.308903704	3.31E-07	RRN3 /// RRN3P1 /// RRN3P2
1839	202570_s_at	-1.308766667	2.39E-07	DLGAP4
1840	209825_s_at	-1.308448148	3.18E-07	MIR3658 /// UCK2
1841	208829_at	-1.308244444	6.11E-13	TAPBP
1842	216640_s_at	-1.307892593	1.21E-11	PDIA6
1843	201028_s_at	-1.307351852	8.57E-11	CD99
1844	204159_at	-1.306822222	1.46E-07	CDKN2C
1845	210512_s_at	-1.306533333	3.95E-06	VEGFA
1846	207704_s_at	-1.306348148	7.89E-06	GAS7
1847	212791_at	-1.306144444	2.67E-13	C1orf216
1848	201090_x_at	-1.305692593	8.82E-16	TUBA1B
1849	214806_at	-1.305274074	7.38E-06	BICD1
1850	211031_s_at	-1.30457037	5.92E-09	CLIP2
1851	225665_at	-1.304055556	7.14E-11	ZAK
1852	215501_s_at	-1.302892593	8.27E-08	DUSP10
1853	207686_s_at	-1.302714815	5.33E-07	CASP8
1854	207180_s_at	-1.301033333	3.76E-11	HTATIP2
1855	209264_s_at	-1.300840741	5.53E-14	TSPAN4
1856	211085_s_at	-1.300207407	5.99E-08	STK4
1857	219799_s_at	-1.299677778	3.95E-07	DHRS9
1858	229064_s_at	-1.2995	2.75E-06	RCAN3
1859	202626_s_at	-1.299288889	1.85E-12	LYN
1860	213816_s_at	-1.299266667	1.61E-08	MET
1861	210427_x_at	-1.299159259	2.65E-12	ANXA2
1862	217007_s_at	-1.298177778	6.52E-07	ADAM15
1863	204767_s_at	-1.297225926	5.92E-10	FEN1
1864	215051_x_at	-1.295944444	2.03E-07	AIF1
1865	204929_s_at	-1.295055556	7.19E-15	VAMP5
1866	201590_x_at	-1.294225926	1.99E-12	ANXA2
1867	224796_at	-1.294114815	2.14E-12	ASAP1
1868	202897_at	-1.294107407	1.18E-11	SIRPA

1869	204859_s_at	-1.293674074	4.25E-10	APAF1
1870	215836_s_at	-1.293411111	2.74E-13	PCDHGA1 /// PCDHGA10 /// PCDHGA11 /// PCDHGA12 /// PCDHGA2 /// PCDHGA3 /// PCDHGA4 /// PCDHGA5 /// PCDHGA6 /// PCDHGA7 /// PCDHGA8 /// PCDHGA9 /// PCDHGB1 /// PCDHGB2 /// PCDHGB3 /// PCDHGB4 /// PCDHGB5 /// PCDHGB6 /// PCDHGB7 /// PCDHGC3 /// PCDHGC4 /// PCDHGC5
1871	200771_at	-1.292703704	8.69E-12	LAMC1
1872	200920_s_at	-1.292007407	3.55E-11	BTG1
1873	201477_s_at	-1.291692593	4.35E-10	RRM1
1874	1554761_a_at	-1.29137037	4.37E-08	HEATR2
1875	210186_s_at	-1.291248148	4.36E-09	FKBP1A /// LOC101929368
1876	205796_at	-1.290985185	7.31E-08	TCP11L1
1877	221563_at	-1.290477778	5.46E-09	DUSP10
1878	226499_at	-1.288748148	1.75E-06	NRARP
1879	203222_s_at	-1.288577778	5.55E-06	TLE1
1880	203414_at	-1.288455556	3.22E-09	MMD
1881	207813_s_at	-1.288418519	2.58E-09	FDXR
1882	224577_at	-1.286151852	2.34E-12	ERGIC1
1883	223553_s_at	-1.285925926	7.73E-11	DOK3
1884	219033_at	-1.285255556	2.74E-10	PARP8
1885	221058_s_at	-1.285018519	7.35E-11	CKLF
1886	226632_at	-1.284633333	6.91E-06	CYGB
1887	1556051_a_at	-1.283655556	9.18E-07	BICD1
1888	218818_at	-1.283551852	3.22E-06	FHL3
1889	201091_s_at	-1.282925926	4.21E-13	CBX3
1890	222547_at	-1.282655556	3.88E-10	MAP4K4
1891	214441_at	-1.282496296	7.30E-06	STX6
1892	210257_x_at	-1.281437037	9.67E-14	CUL4B
1893	202804_at	-1.280996296	1.30E-08	ABCC1
1894	228597_at	-1.280966667	1.09E-12	MIS18A
1895	217503_at	-1.280959259	6.80E-09	STK17B
1896	225833_at	-1.280903704	5.41E-09	DAGLB
1897	218913_s_at	-1.280759259	2.89E-08	GMIP
1898	223169_s_at	-1.2804	2.74E-06	RHOU
1899	217791_s_at	-1.27937037	1.10E-10	ALDH18A1
1900	204153_s_at	-1.279122222	1.99E-10	MFNG
1901	225698_at	-1.278914815	2.67E-08	EPB41L4A-AS1
1902	222918_at	-1.278648148	7.16E-08	RAB9B
1903	205020_s_at	-1.27822963	1.11E-08	ARL4A
1904	215143_at	-1.277881481	7.63E-07	DPY19L2P2
1905	228461_at	-1.27772963	1.48E-08	SH3RF3

1906	212268_at	-1.277625926	1.34E-11	SERPINB1
1907	212481_s_at	-1.277051852	2.02E-07	TPM4
1908	202191_s_at	-1.276825926	1.08E-06	GAS7
1909	205802_at	-1.275922222	1.05E-10	TRPC1
1910	205047_s_at	-1.275814815	2.30E-07	ASNS
1911	205027_s_at	-1.275074074	7.91E-09	MAP3K8
1912	221497_x_at	-1.275059259	6.39E-12	EGLN1
1913	218559_s_at	-1.274977778	6.50E-09	MAFB
1914	202727_s_at	-1.274825926	3.28E-15	IFNGR1
1915	229629_at	-1.274440741	3.54E-06	
1916	209901_x_at	-1.274348148	6.55E-07	AIF1
1917	210705_s_at	-1.2735	3.45E-11	TRIM5
1918	218729_at	-1.273159259	1.78E-08	LXN
1919	201697_s_at	-1.271785185	1.07E-12	DNMT1
1920	232048_at	-1.271244444	7.75E-07	FAM76B
1921	232033_at	-1.270125926	2.14E-08	USP37
1922	204017_at	-1.269574074	4.89E-06	KDELR3
1923	235489_at	-1.268359259	3.54E-06	RHOJ
1924	213864_s_at	-1.268207407	7.87E-12	NAP1L1
1925	1552648_a_at	-1.268077778	3.27E-11	TNFRSF10A
1926	201866_s_at	-1.267825926	1.96E-11	NR3C1
1927	204172_at	-1.266877778	2.72E-08	CPOX
1928	200656_s_at	-1.266748148	2.80E-12	P4HB
1929	204681_s_at	-1.266562963	7.57E-06	RAPGEF5
1930	209535_s_at	-1.264040741	4.89E-08	
1931	229968_at	-1.263785185	1.33E-08	
1932	210044_s_at	-1.263688889	2.41E-07	LYL1
1933	204244_s_at	-1.262	8.70E-08	DBF4
1934	218618_s_at	-1.261322222	3.45E-12	FNDC3B /// LOC101928615
1935	242444_at	-1.260744444	1.75E-06	C1QTNF6
1936	224996_at	-1.260614815	1.58E-11	ASPH
1937	209172_s_at	-1.260592593	9.35E-06	CENPF
1938	210896_s_at	-1.260388889	2.98E-08	ASPH
1939	234996_at	-1.260185185	3.09E-06	CALCRL
1940	216483_s_at	-1.259807407	4.93E-14	C19orf10
1941	209026_x_at	-1.259777778	1.66E-10	TUBB
1942	214632_at	-1.259048148	1.10E-07	NRP2
1943	235183_at	-1.258759259	3.58E-06	FILIP1
1944	212811_x_at	-1.258333333	2.77E-12	SLC1A4
1945	201617_x_at	-1.257844444	6.23E-07	CALD1
1946	215617_at	-1.256718519	8.55E-10	SPATS2L
1947	211450_s_at	-1.256714815	5.32E-09	MSH6
1948	202720_at	-1.255477778	3.92E-11	TES

1949	218602_s_at	-1.255366667	4.11E-09	HAUS6
1950	221773_at	-1.254848148	3.43E-10	ELK3
1951	209198_s_at	-1.254166667	1.01E-08	SYT11
1952	217192_s_at	-1.25387037	6.39E-06	PRDM1
1953	202657_s_at	-1.253681481	8.88E-13	SERTAD2
1954	1554885_a_at	-1.253640741	1.38E-06	PRIM2 /// PRIM2B
1955	235901_at	-1.253096296	5.22E-06	RP11-589P10.5
1956	65588_at	-1.252985185	8.58E-08	SNHG17
1957	226117_at	-1.252318519	8.66E-10	TIFA
1958	202969_at	-1.251974074	1.08E-09	DYRK2
1959	220199_s_at	-1.251362963	3.04E-11	AIDA
1960	210865_at	-1.251222222	1.07E-06	FASLG
1961	212484_at	-1.249951852	1.78E-10	FAM89B
1962	211623_s_at	-1.249022222	2.48E-14	FBL
1963	228382_at	-1.248177778	9.43E-06	OTULIN
1964	206693_at	-1.246518519	1.70E-08	IL7
1965	202163_s_at	-1.245866667	3.04E-10	CNOT8
1966	222450_at	-1.24577037	4.35E-08	PMEPA1
1967	208442_s_at	-1.245544444	9.88E-09	ATM
1968	200641_s_at	-1.245237037	2.87E-08	YWHAZ
1969	218764_at	-1.244833333	4.12E-08	PRKCH
1970	1558015_s_at	-1.24482963	7.57E-07	ACTR2
1971	209780_at	-1.244459259	1.52E-15	PHTF2
1972	203867_s_at	-1.244292593	1.23E-06	NLE1
1973	202122_s_at	-1.244274074	3.05E-09	PLIN3
1974	210733_at	-1.243392593	5.89E-07	TRAM1
1975	205746_s_at	-1.241811111	1.16E-09	ADAM17
1976	211963_s_at	-1.241451852	1.96E-15	ARPC5
1977	211329_x_at	-1.240485185	6.41E-06	HFE
1978	202649_x_at	-1.239981481	3.95E-17	RPS19
1979	228311_at	-1.239803704	3.77E-07	BCL6B
1980	226950_at	-1.238455556	2.04E-06	ACVRL1
1981	204525_at	-1.238451852	1.36E-06	PHF14
1982	236046_at	-1.237503704	5.78E-09	CCDC127
1983	244661_at	-1.236851852	1.72E-08	SOAT1
1984	204795_at	-1.23672963	2.96E-07	PRR3
1985	200966_x_at	-1.236681481	9.77E-15	ALDOA
1986	209822_s_at	-1.236537037	9.13E-11	VLDLR
1987	220239_at	-1.235837037	4.65E-09	KLHL7
1988	217949_s_at	-1.235222222	3.17E-11	VKORC1
1989	213414_s_at	-1.234692593	1.91E-18	RPS19
1990	203132_at	-1.23392963	1.38E-12	RB1
1991	209053_s_at	-1.232855556	7.18E-07	WHSC1

1992	212639_x_at	-1.232392593	1.45E-14	TUBA1B
1993	215346_at	-1.232366667	9.84E-11	CD40
1994	214687_x_at	-1.231885185	5.58E-15	ALDOA
1995	221935_s_at	-1.231866667	4.85E-10	EOGT
1996	213166_x_at	-1.231777778	2.92E-11	MIR4784 /// MZT2A /// MZT2B
1997	201160_s_at	-1.231611111	1.39E-13	YBX3
1998	212295_s_at	-1.23147037	1.24E-08	SLC7A1
1999	202007_at	-1.231211111	1.03E-07	NID1
2000	211072_x_at	-1.230081481	2.40E-16	TUBA1B
2001	211581_x_at	-1.229977778	4.45E-11	LST1
2002	235122_at	-1.229059259	3.24E-07	HIVEP3
2003	222858_s_at	-1.228407407	1.59E-07	DAPP1
2004	202213_s_at	-1.228359259	3.81E-13	CUL4B
2005	201042_at	-1.227355556	3.28E-06	TGM2
2006	227107_at	-1.227259259	6.42E-12	PANX1
2007	209606_at	-1.226437037	1.50E-07	CYTIP
2008	217379_at	-1.226055556	1.38E-09	RP11-209A2.1
2009	204670_x_at	-1.225818519	5.32E-11	HLA-DRB1 /// HLA-DRB4 /// LOC100996809
2010	215411_s_at	-1.225666667	1.13E-11	TRAF3IP2
2011	202458_at	-1.225648148	1.23E-06	PRSS23
2012	210840_s_at	-1.225181481	4.33E-13	IQGAP1
2013	200853_at	-1.225033333	1.60E-11	H2AFZ
2014	212563_at	-1.224237037	7.54E-09	BOP1 /// MIR7112
2015	211135_x_at	-1.223722222	1.60E-07	LILRB3
2016	209310_s_at	-1.223703704	4.33E-11	CASP4
2017	202971_s_at	-1.223525926	2.26E-09	DYRK2
2018	229173_at	-1.223481481	7.87E-11	KIAA1715
2019	203269_at	-1.221711111	3.63E-13	NSMAF
2020	209644_x_at	-1.2216	3.82E-07	CDKN2A
2021	202848_s_at	-1.220507407	1.88E-06	GRK6
2022	218404_at	-1.220459259	1.78E-07	SNX10
2023	207857_at	-1.22042963	4.79E-08	LILRA2
2024	204396_s_at	-1.220059259	5.68E-07	GRK5
2025	212110_at	-1.218762963	5.25E-06	SLC39A14
2026	38964_r_at	-1.218418519	4.24E-11	WAS
2027	226799_at	-1.217785185	6.87E-09	FGD6
2028	235286_at	-1.216825926	3.85E-09	
2029	201718_s_at	-1.215688889	3.09E-09	EPB41L2
2030	200921_s_at	-1.215466667	2.49E-13	BTG1
2031	222693_at	-1.214803704	9.03E-09	FNDC3B /// LOC101928615
2032	220603_s_at	-1.214607407	8.38E-09	MCTP2
2033	211863_x_at	-1.2139	6.35E-06	HFE
2034	44120_at	-1.213774074	9.84E-16	ADCK2

2035	209723_at	-1.213440741	1.10E-08	SERPINB9
2036	212672_at	-1.213359259	1.84E-10	ATM
2037	204115_at	-1.213207407	5.76E-06	GNG11
2038	216620_s_at	-1.212814815	9.26E-14	ARHGEF10
2039	1552555_at	-1.21262963	1.04E-06	PRSS36
2040	208047_s_at	-1.212625926	1.52E-09	NAB1
2041	222876_s_at	-1.212240741	1.22E-07	ADAP2
2042	235295_at	-1.212074074	2.56E-10	PANX1
2043	201865_x_at	-1.21102963	4.80E-14	NR3C1
2044	208363_s_at	-1.210144444	6.97E-06	INPP4A
2045	203888_at	-1.208814815	4.39E-08	THBD
2046	235965_at	-1.208433333	2.21E-06	
2047	212667_at	-1.208059259	4.16E-08	SPARC
2048	224928_at	-1.207511111	1.96E-14	SETD7
2049	229510_at	-1.207088889	9.28E-06	MS4A14
2050	227139_s_at	-1.206881481	2.30E-11	HPS3
2051	219358_s_at	-1.206777778	1.62E-10	ADAP2
2052	239657_x_at	-1.206451852	6.70E-07	FOXO6
2053	40420_at	-1.206318519	3.36E-14	STK10
2054	206667_s_at	-1.205692593	5.69E-06	SCAMP1
2055	208934_s_at	-1.205659259	2.10E-10	LGALS8
2056	200707_at	-1.20427037	2.46E-13	PRKCSH
2057	219183_s_at	-1.203848148	1.75E-09	CYTH4
2058	207565_s_at	-1.203651852	5.85E-10	MR1
2059	32137_at	-1.203414815	1.59E-10	JAG2
2060	209166_s_at	-1.203048148	1.11E-11	MAN2B1
2061	214797_s_at	-1.203025926	2.82E-08	CDK18
2062	213646_x_at	-1.20257037	4.44E-15	TUBA1B
2063	209827_s_at	-1.202285185	3.01E-11	IL16
2064	210660_at	-1.201911111	2.03E-06	LILRA1
2065	221580_s_at	-1.200918519	3.63E-10	MIR1304 /// SNORA1 /// SNORA18 /// SNORA32 /// SNORA40 /// SNORA8 /// SNORD5 /// TAF1D
2066	236782_at	-1.2003	1.25E-06	SAMD3
2067	210154_at	-1.199103704	1.76E-09	ME2
2068	202693_s_at	-1.198955556	1.00E-08	STK17A
2069	59644_at	-1.198151852	6.94E-09	BMP2K
2070	1553021_s_at	-1.197988889	9.65E-06	BICD2
2071	225030_at	-1.197674074	1.89E-13	BOD1
2072	242281_at	-1.197340741	5.95E-08	GLUL
2073	229614_at	-1.197137037	5.28E-06	ZNF320
2074	1557236_at	-1.196811111	1.49E-06	APOL6
2075	225018_at	-1.196792593	5.66E-09	SPIRE1
2076	212815_at	-1.195503704	2.21E-09	ASCC3

2077	202820_at	-1.194133333	2.02E-09	AHR
2078	214247_s_at	-1.1939	1.73E-06	DKK3
2079	225793_at	-1.193722222	1.68E-15	LIX1L
2080	203086_at	-1.193677778	1.04E-08	KIF2A
2081	222543_at	-1.192544444	3.58E-13	DERL1
2082	208659_at	-1.190488889	1.11E-13	CLIC1
2083	204860_s_at	-1.190240741	4.85E-07	NAIP
2084	219691_at	-1.190085185	3.68E-06	SAMD9
2085	206342_x_at	-1.190055556	3.99E-12	IDS
2086	1555247_a_at	-1.188722222	2.53E-09	RAPGEF6
2087	218481_at	-1.188185185	1.26E-12	EXOSC5
2088	217738_at	-1.188011111	2.40E-08	NAMPT
2089	235593_at	-1.187811111	2.81E-08	ZEB2
2090	236831_at	-1.1876	9.96E-09	CCDC50
2091	225536_at	-1.187537037	2.89E-06	TMEM54
2092	214096_s_at	-1.186051852	5.67E-11	SHMT2
2093	205191_at	-1.185792593	4.20E-13	RP2
2094	219303_at	-1.185533333	1.71E-12	RNF219
2095	218008_at	-1.185344444	3.23E-12	TMEM248
2096	203664_s_at	-1.184844444	4.03E-11	POLR2D
2097	209230_s_at	-1.184192593	1.50E-07	NUPR1
2098	208876_s_at	-1.1836	1.99E-12	PAK2
2099	216862_s_at	-1.183477778	6.04E-10	CMC4
2100	208319_s_at	-1.183159259	6.92E-10	RBM3
2101	244487_at	-1.1828	1.21E-10	NCK1
2102	209762_x_at	-1.18227037	3.04E-14	SP110
2103	226837_at	-1.182062963	1.03E-12	SPRED1
2104	210416_s_at	-1.181881481	2.43E-07	CHEK2
2105	206472_s_at	-1.181740741	5.55E-12	TLE3
2106	215812_s_at	-1.181551852	2.80E-06	SLC6A10P /// SLC6A10PB /// SLC6A8
2107	201476_s_at	-1.181474074	1.20E-08	RRM1
2108	208306_x_at	-1.180581481	1.74E-11	HLA-DRB1 /// HLA-DRB1
2109	202014_at	-1.180459259	2.45E-07	PPP1R15A
2110	201202_at	-1.180337037	2.45E-10	PCNA
2111	226320_at	-1.179514815	5.82E-12	ALYREF
2112	226525_at	-1.178825926	2.96E-09	STK17B
2113	212561_at	-1.178618519	3.26E-14	DENND5A
2114	234725_s_at	-1.178577778	2.58E-08	SEMA4B
2115	238034_at	-1.178388889	5.44E-13	CANX
2116	1555594_a_at	-1.177044444	6.21E-07	MBNL1
2117	219885_at	-1.176962963	1.81E-10	SLFN12
2118	219278_at	-1.176837037	6.09E-09	MAP3K6
2119	207485_x_at	-1.176303704	2.09E-09	BTN3A1

2120	201876_at	-1.175651852	1.54E-06	PON2
2121	209969_s_at	-1.175533333	4.42E-06	STAT1
2122	1555565_s_at	-1.17517037	1.75E-08	TAPBP
2123	202877_s_at	-1.175033333	1.72E-07	CD93
2124	1556389_at	-1.174522222	3.01E-08	CNPY3
2125	221218_s_at	-1.17397037	3.21E-10	TPK1
2126	217456_x_at	-1.173281481	9.69E-12	HLA-E
2127	203753_at	-1.173025926	9.33E-09	TCF4
2128	212646_at	-1.172788889	1.79E-08	RFTN1
2129	209574_s_at	-1.172418519	9.97E-07	LDLRAD4
2130	206637_at	-1.171666667	2.38E-06	P2RY14
2131	228607_at	-1.171222222	9.75E-07	OAS2
2132	218193_s_at	-1.169592593	1.80E-08	GOLT1B
2133	205003_at	-1.169474074	4.76E-13	DOCK4
2134	227726_at	-1.169422222	8.54E-14	RNF166
2135	211300_s_at	-1.169325926	2.85E-07	TP53
2136	240757_at	-1.16792963	1.06E-06	CLASP1
2137	213535_s_at	-1.167648148	1.34E-13	UBE2I
2138	204265_s_at	-1.166103704	1.95E-08	GPSM3
2139	1552613_s_at	-1.1661	7.18E-06	CDC42SE2
2140	201577_at	-1.16597037	4.15E-09	NME1
2141	223172_s_at	-1.165844444	7.49E-08	MTFP1
2142	210983_s_at	-1.165748148	5.01E-10	MCM7
2143	230206_at	-1.165507407	1.84E-08	DOCK5
2144	206571_s_at	-1.164903704	4.53E-09	MAP4K4
2145	224468_s_at	-1.164011111	1.60E-07	C19orf48 /// SNORD88C
2146	201151_s_at	-1.163240741	3.29E-08	MBNL1
2147	227598_at	-1.162096296	9.01E-06	ZBED6CL
2148	213860_x_at	-1.161540741	2.71E-12	CSNK1A1
2149	229367_s_at	-1.160344444	1.45E-06	GIMAP6
2150	200638_s_at	-1.160296296	3.87E-11	YWHAZ
2151	219361_s_at	-1.160233333	1.48E-07	AEN
2152	208675_s_at	-1.159640741	9.79E-14	DDOST
2153	219481_at	-1.159625926	2.78E-09	TTC13
2154	227787_s_at	-1.159585185	6.02E-12	MED30
2155	223079_s_at	-1.159296296	4.72E-06	GLS
2156	223452_s_at	-1.158755556	3.33E-08	ATL3
2157	235766_x_at	-1.158392593	5.68E-09	RAB27A
2158	205147_x_at	-1.157125926	1.63E-07	NCF4
2159	1555762_s_at	-1.157092593	1.69E-10	RBM15
2160	219777_at	-1.1569	7.19E-07	GIMAP6
2161	216449_x_at	-1.156577778	1.79E-09	HSP90B1
2162	223584_s_at	-1.156362963	8.74E-09	KBTBD2

2163	203504_s_at	-1.156240741	1.30E-07	ABCA1
2164	239952_at	-1.156211111	3.18E-07	LOC100996668 /// ZEB1
2165	222503_s_at	-1.156181481	2.40E-14	WDR41
2166	200904_at	-1.153892593	7.01E-10	HLA-E
2167	205997_at	-1.15292963	6.80E-06	ADAM28
2168	243539_at	-1.150940741	4.58E-07	KIAA1841
2169	224937_at	-1.150696296	3.01E-06	PTGFRN
2170	209770_at	-1.150477778	1.17E-10	BTN3A1
2171	211714_x_at	-1.149707407	2.11E-11	TUBB
2172	208960_s_at	-1.149396296	1.87E-06	KLF6
2173	211063_s_at	-1.148040741	1.01E-12	NCK1
2174	1552264_a_at	-1.147637037	8.41E-09	MAPK1
2175	212311_at	-1.145722222	4.76E-07	SEL1L3
2176	209754_s_at	-1.145603704	1.09E-06	TMPO
2177	226568_at	-1.144792593	5.48E-11	FAM102B
2178	210567_s_at	-1.14477037	6.39E-06	SKP2
2179	213101_s_at	-1.143774074	2.94E-14	ACTR3
2180	210022_at	-1.142822222	9.27E-13	PCGF1
2181	204569_at	-1.141766667	2.22E-09	ICK
2182	216899_s_at	-1.141474074	2.41E-08	SKAP2
2183	218854_at	-1.141322222	9.27E-08	DSE
2184	211676_s_at	-1.140944444	5.78E-12	IFNGR1
2185	218984_at	-1.140414815	2.22E-10	PUS7
2186	218823_s_at	-1.140381481	1.21E-12	KCTD9
2187	225558_at	-1.140348148	1.81E-10	GIT2
2188	230748_at	-1.139892593	3.35E-06	SLC16A6
2189	209662_at	-1.139748148	1.98E-13	CETN3
2190	201848_s_at	-1.139303704	2.42E-06	BNIP3
2191	210987_x_at	-1.139033333	1.12E-08	TPM1
2192	217436_x_at	-1.138448148	1.85E-09	HLA-J
2193	205235_s_at	-1.13832963	2.21E-08	KIF20B
2194	226879_at	-1.137603704	1.84E-09	HVCN1
2195	214487_s_at	-1.137140741	2.21E-06	RAP2A /// RAP2B
2196	204415_at	-1.137062963	1.50E-06	IFI6
2197	205519_at	-1.136896296	2.94E-07	WDR76
2198	227609_at	-1.136851852	4.35E-07	EPSTI1
2199	204427_s_at	-1.136496296	1.63E-07	TMED2
2200	217299_s_at	-1.135848148	7.83E-12	NBN
2201	209135_at	-1.135307407	1.23E-06	ASPH
2202	1552610_a_at	-1.134607407	7.66E-09	JAK1
2203	205071_x_at	-1.133766667	3.36E-08	XRCC4
2204	209421_at	-1.133314815	4.10E-12	MSH2
2205	232065_x_at	-1.132844444	2.13E-07	CENPL

2206	221638_s_at	-1.132677778	6.29E-06	STX16
2207	1555106_a_at	-1.132177778	8.81E-08	CTDSPL2
2208	206613_s_at	-1.132174074	1.84E-08	TAF1A
2209	218728_s_at	-1.132048148	1.04E-09	CNIH4
2210	200889_s_at	-1.131603704	1.66E-07	SSR1
2211	230621_at	-1.130359259	1.68E-10	IAH1
2212	208925_at	-1.130077778	9.02E-09	CLDND1
2213	222146_s_at	-1.130059259	8.74E-07	TCF4
2214	217371_s_at	-1.129759259	1.94E-08	IL15
2215	209514_s_at	-1.129407407	5.78E-09	RAB27A
2216	201528_at	-1.129385185	1.81E-12	RPA1
2217	224925_at	-1.129103704	7.29E-09	PREX1
2218	218880_at	-1.1283	6.25E-07	FOSL2
2219	206117_at	-1.12787037	5.05E-06	TPM1
2220	202153_s_at	-1.127055556	3.04E-14	NUP62
2221	209215_at	-1.127018519	1.34E-07	MFSD10
2222	241704_x_at	-1.126685185	8.01E-07	ZNF320
2223	222392_x_at	-1.126144444	5.69E-09	PERP
2224	226876_at	-1.125314815	3.44E-08	FAM101B
2225	230314_at	-1.12497037	4.27E-11	
2226	202719_s_at	-1.123840741	7.81E-10	TES
2227	200760_s_at	-1.123203704	1.25E-10	ARL6IP5
2228	203175_at	-1.123003704	7.13E-15	RHOG
2229	201642_at	-1.122851852	1.92E-13	IFNGR2
2230	243252_at	-1.122614815	1.50E-06	
2231	204436_at	-1.121488889	5.83E-13	PLEKHO2
2232	213872_at	-1.121403704	1.73E-06	
2233	242228_at	-1.1214	1.94E-06	BICD1
2234	212647_at	-1.121285185	6.65E-11	RRAS
2235	200798_x_at	-1.120440741	3.92E-08	MCL1
2236	221060_s_at	-1.119637037	3.29E-09	TLR4
2237	219332_at	-1.119314815	1.22E-09	MICALL2
2238	222659_at	-1.118892593	2.35E-12	IPO11 /// IPO11-LRRC70
2239	210153_s_at	-1.117196296	6.72E-10	ME2
2240	209788_s_at	-1.117022222	1.75E-06	ERAP1
2241	211833_s_at	-1.116822222	3.50E-11	BAX
2242	216971_s_at	-1.116555556	1.91E-06	PLEC
2243	202763_at	-1.115118519	8.40E-12	CASP3
2244	211048_s_at	-1.114844444	1.53E-08	PDIA4
2245	200654_at	-1.114674074	1.25E-10	P4HB
2246	200727_s_at	-1.1129	1.05E-08	ACTR2
2247	208657_s_at	-1.112807407	9.82E-07	9-Sep
2248	226464_at	-1.112788889	2.79E-12	C3orf58

2249	214853_s_at	-1.111392593	4.30E-07	SHC1
2250	209062_x_at	-1.110125926	8.41E-06	NCOA3
2251	201930_at	-1.109792593	2.72E-10	MCM6
2252	227964_at	-1.109666667	4.34E-12	FRMD8
2253	210357_s_at	-1.109303704	1.14E-06	SMOX
2254	204214_s_at	-1.109011111	1.53E-12	RAB32
2255	221894_at	-1.108566667	9.19E-11	ADCK2
2256	216323_x_at	-1.10817037	2.16E-08	TUBA3C /// TUBA3D
2257	221685_s_at	-1.107018519	2.20E-07	SPDL1
2258	211986_at	-1.106474074	4.17E-09	AHNAK
2259	200003_s_at	-1.106340741	3.03E-15	MIR6805 /// RPL28
2260	212115_at	-1.106062963	2.37E-09	HN1L
2261	225600_at	-1.106037037	4.64E-09	TRIQK
2262	215997_s_at	-1.105244444	1.59E-09	CUL4B
2263	221649_s_at	-1.104877778	7.41E-06	PPAN /// PPAN-P2RY11
2264	221677_s_at	-1.104125926	7.43E-10	DONSON
2265	230186_at	-1.102462963	6.21E-08	TMEM136
2266	213175_s_at	-1.102133333	1.30E-12	SNRPB
2267	229670_at	-1.101807407	5.36E-10	
2268	208816_x_at	-1.101662963	5.17E-07	ANXA2P2
2269	208351_s_at	-1.101437037	8.67E-08	MAPK1
2270	1553906_s_at	-1.100992593	4.80E-07	FGD2
2271	232645_at	-1.100903704	8.80E-06	LOC153684
2272	235036_at	-1.099637037	8.11E-08	LIX1L
2273	225971_at	-1.098548148	1.75E-08	DDHD1
2274	201657_at	-1.098337037	1.71E-09	ARL1
2275	205603_s_at	-1.098014815	4.93E-10	DIAPH2
2276	208961_s_at	-1.097925926	9.74E-07	KLF6
2277	225674_at	-1.097325926	2.71E-10	BCAP29
2278	217202_s_at	-1.097103704	3.76E-07	GLUL
2279	204194_at	-1.096974074	4.49E-11	BACH1
2280	221989_at	-1.096651852	1.15E-06	RPL10 /// SNORA70
2281	211964_at	-1.095833333	2.24E-09	COL4A2
2282	201851_at	-1.095392593	1.25E-08	SH3GL1
2283	208752_x_at	-1.095262963	6.95E-11	NAP1L1
2284	202805_s_at	-1.094925926	3.01E-07	ABCC1
2285	202370_s_at	-1.094807407	4.94E-16	CBFB
2286	221471_at	-1.094637037	9.98E-15	SERINC3
2287	204413_at	-1.093966667	8.43E-06	TRAF2
2288	204785_x_at	-1.092718519	9.84E-11	IFNAR2
2289	209451_at	-1.092718519	4.91E-09	TANK
2290	227545_at	-1.092622222	1.43E-06	BARD1
2291	212800_at	-1.091751852	5.55E-10	STX6

2292	227792_at	-1.091607407	3.66E-09	ITPRIPL2
2293	222685_at	-1.091592593	1.92E-11	HAUS6
2294	225160_x_at	-1.091285185	2.27E-08	MDM2
2295	228641_at	-1.091188889	9.59E-09	CARD8
2296	202639_s_at	-1.091048148	9.98E-06	RANBP3
2297	203622_s_at	-1.090833333	1.84E-11	PNO1
2298	218977_s_at	-1.090607407	1.68E-11	TRNAU1AP
2299	228725_x_at	-1.090522222	6.41E-10	PRMT2
2300	208309_s_at	-1.088314815	1.22E-07	MALT1
2301	216321_s_at	-1.087325926	2.35E-11	NR3C1
2302	225366_at	-1.086303704	1.05E-10	PGM2
2303	231775_at	-1.085851852	4.73E-11	TNFRSF10A
2304	227846_at	-1.0855	4.43E-09	GPR176
2305	202594_at	-1.085185185	2.03E-08	LEPROTL1
2306	210904_s_at	-1.084881481	1.29E-07	IL13RA1
2307	238455_at	-1.084659259	2.25E-08	PLXDC2
2308	208921_s_at	-1.084025926	1.33E-12	SRI
2309	223413_s_at	-1.083659259	4.02E-12	LYAR
2310	38487_at	-1.08347037	1.63E-08	STAB1
2311	202656_s_at	-1.08332963	3.21E-11	SERTAD2
2312	212685_s_at	-1.082544444	5.80E-13	TBL2
2313	210212_x_at	-1.081977778	3.77E-09	CMC4
2314	211675_s_at	-1.080844444	8.23E-13	MDFIC
2315	229304_s_at	-1.080259259	4.33E-06	CENPU
2316	209079_x_at	-1.079362963	8.47E-10	PCDHGA1 /// PCDHGA10 /// PCDHGA11 /// PCDHGA12 /// PCDHGA2 /// PCDHGA3 /// PCDHGA4 /// PCDHGA5 /// PCDHGA6 /// PCDHGA7 /// PCDHGA8 /// PCDHGA9 /// PCDHGB1 /// PCDHGB2 /// PCDHGB3 /// PCDHGB4 /// PCDHGB5 /// PCDHGB6 /// PCDHGB7 /// PCDHGC3 /// PCDHGC4 /// PCDHGC5
2317	200782_at	-1.079337037	9.83E-13	ANXA5
2318	210829_s_at	-1.078977778	2.57E-07	SSBP2
2319	211557_x_at	-1.078362963	2.10E-06	SLCO2B1
2320	210792_x_at	-1.078237037	5.47E-07	SIVA1
2321	234942_s_at	-1.077766667	5.52E-07	DNTTIP1
2322	214167_s_at	-1.07772963	2.11E-14	RPLP0
2323	225836_s_at	-1.077414815	5.97E-08	RHNO1
2324	200729_s_at	-1.076966667	6.23E-10	ACTR2
2325	233587_s_at	-1.076607407	1.63E-07	SIPA1L2
2326	205992_s_at	-1.07472963	1.64E-09	IL15
2327	238475_at	-1.074359259	7.39E-07	ALG10 /// ALG10B
2328	211358_s_at	-1.073922222	3.68E-10	CIZ1

2329	212853_at	-1.073559259	5.37E-06	DCUN1D4
2330	232149_s_at	-1.07337037	6.19E-10	NSMAF
2331	238430_x_at	-1.072992593	9.28E-09	SLFN5
2332	213857_s_at	-1.07267037	1.07E-10	CD47
2333	229689_s_at	-1.07237037	2.36E-06	RP13-39P12.3
2334	212577_at	-1.072318519	6.74E-10	SMCHD1
2335	229830_at	-1.072207407	4.80E-07	
2336	1552316_a_at	-1.07212963	3.87E-09	GIMAP1
2337	1565951_s_at	-1.072062963	4.69E-06	CHML
2338	1555831_s_at	-1.070911111	3.72E-06	LRRC41
2339	229256_at	-1.070748148	2.07E-07	PGM2L1
2340	235828_at	-1.070744444	4.56E-11	PRELID2
2341	219979_s_at	-1.070725926	4.64E-09	C11orf73
2342	203675_at	-1.070640741	1.43E-10	NUCB2
2343	226630_at	-1.070337037	7.84E-11	MIS18BP1
2344	200887_s_at	-1.069366667	3.15E-08	STAT1
2345	213620_s_at	-1.069051852	3.79E-06	ICAM2
2346	243299_at	-1.068107407	7.75E-06	
2347	210458_s_at	-1.067637037	2.42E-08	TANK
2348	218997_at	-1.067603704	4.97E-12	POLR1E
2349	201616_s_at	-1.067207407	8.67E-08	CALD1
2350	206550_s_at	-1.066466667	7.70E-10	NUP155
2351	225599_s_at	-1.064855556	6.77E-08	TRIQK
2352	202947_s_at	-1.063666667	2.22E-10	GYPC
2353	202531_at	-1.062925926	7.15E-08	IRF1
2354	200950_at	-1.061911111	5.63E-12	ARPC1A
2355	201888_s_at	-1.061007407	8.74E-10	IL13RA1
2356	223686_at	-1.060681481	1.54E-07	TPK1
2357	218614_at	-1.060474074	2.59E-09	KIAA1551
2358	218055_s_at	-1.060177778	1.29E-07	WDR41
2359	212433_x_at	-1.059085185	4.02E-16	RPS2 /// SNORA64
2360	210223_s_at	-1.058851852	4.97E-11	MR1
2361	202730_s_at	-1.057914815	1.75E-08	MIR4680 /// PDCD4
2362	201231_s_at	-1.057244444	9.75E-11	ENO1
2363	201584_s_at	-1.056792593	2.91E-10	DDX39A
2364	1568611_at	-1.056777778	9.27E-06	
2365	208310_s_at	-1.056074074	8.40E-13	CCZ1 /// CCZ1B
2366	219512_at	-1.05587037	1.30E-07	DSN1
2367	243894_at	-1.055725926	9.75E-07	SLC41A2
2368	213532_at	-1.054877778	7.98E-13	ADAM17
2369	223018_at	-1.054751852	2.12E-11	NOB1
2370	202161_at	-1.05427037	3.81E-11	PKN1
2371	222204_s_at	-1.054077778	1.10E-12	RRN3

2372	208012_x_at	-1.053940741	2.42E-12	SP110
2373	208050_s_at	-1.053685185	2.79E-06	CASP2
2374	213026_at	-1.053511111	1.45E-11	ATG12
2375	205660_at	-1.053255556	2.85E-06	OASL
2376	211058_x_at	-1.052666667	1.57E-13	TUBA1B
2377	236115_at	-1.0526	5.09E-07	HTR7P1
2378	200644_at	-1.051807407	1.98E-06	MARCKSL1
2379	201623_s_at	-1.051081481	1.45E-10	DARS
2380	204023_at	-1.050959259	6.10E-11	RFC4
2381	91816_f_at	-1.050825926	1.71E-07	MEX3D
2382	219862_s_at	-1.050607407	3.58E-12	NARF
2383	1557910_at	-1.050325926	6.91E-07	HSP90AB1
2384	200909_s_at	-1.049781481	7.39E-14	RPLP2 /// SNORA52
2385	212630_at	-1.048877778	4.15E-07	EXOC3
2386	203771_s_at	-1.048492593	8.90E-08	BLVRA
2387	1552263_at	-1.048240741	1.76E-10	MAPK1
2388	222670_s_at	-1.04767037	1.30E-07	MAFB
2389	1559517_a_at	-1.047340741	1.71E-07	SPIRE1
2390	47550_at	-1.045885185	3.38E-06	LZTS1
2391	209402_s_at	-1.04527037	6.98E-06	SLC12A4
2392	203817_at	-1.043655556	3.94E-06	GUCY1B3
2393	211750_x_at	-1.043614815	9.64E-12	TUBA1C
2394	1563646_a_at	-1.043488889	1.13E-06	TMEM67
2395	242447_at	-1.042903704	6.18E-06	C3orf70
2396	207760_s_at	-1.042544444	1.44E-09	NCOR2
2397	213352_at	-1.042203704	4.91E-06	TMCC1
2398	1554464_a_at	-1.041481481	2.68E-08	CRTAP
2399	218986_s_at	-1.04027037	8.81E-08	DDX60
2400	203505_at	-1.040137037	3.09E-06	ABCA1
2401	212636_at	-1.039981481	4.68E-13	QKI
2402	232612_s_at	-1.039622222	5.29E-07	ATG16L1
2403	222565_s_at	-1.039088889	5.71E-06	PRKD3
2404	200999_s_at	-1.039062963	8.09E-06	CKAP4
2405	201041_s_at	-1.039003704	2.29E-06	DUSP1
2406	235425_at	-1.038048148	4.70E-06	SGOL2
2407	1729_at	-1.038011111	5.31E-13	TRADD
2408	202164_s_at	-1.037396296	1.23E-11	CNOT8
2409	205210_at	-1.037307407	4.17E-06	TGFBRAP1
2410	235088_at	-1.036218519	5.01E-07	C4orf46
2411	1552277_a_at	-1.03612963	8.32E-09	MSANTD3
2412	204228_at	-1.035951852	1.08E-10	PPIH
2413	209544_at	-1.035785185	1.57E-06	RIPK2
2414	226858_at	-1.035225926	1.82E-07	CSNK1E

2415	201474_s_at	-1.035214815	4.68E-06	ITGA3
2416	208962_s_at	-1.03467037	3.98E-06	FADS1 /// MIR1908
2417	225230_at	-1.034022222	2.33E-10	DRAM2
2418	201647_s_at	-1.033937037	2.83E-07	SCARB2
2419	223168_at	-1.033777778	4.39E-08	RHOU
2420	226422_at	-1.032851852	2.11E-09	ERGIC2
2421	235802_at	-1.032796296	4.22E-06	PLD4
2422	218871_x_at	-1.03257037	2.70E-10	CSGALNACT2
2423	221652_s_at	-1.032014815	9.27E-10	ASUN
2424	203686_at	-1.031748148	3.94E-11	MPG
2425	204510_at	-1.031055556	8.91E-06	CDC7
2426	225869_s_at	-1.031055556	5.38E-08	UNC93B1
2427	201309_x_at	-1.030996296	4.36E-06	NREP
2428	208853_s_at	-1.030859259	5.37E-09	CANX
2429	213054_at	-1.030733333	9.89E-06	HAUS5
2430	200956_s_at	-1.030637037	6.65E-11	SSRP1
2431	223220_s_at	-1.030366667	3.12E-09	PARP9
2432	227350_at	-1.028692593	2.51E-06	HELLS
2433	200805_at	-1.028362963	2.34E-12	LMAN2
2434	225190_x_at	-1.028362963	2.50E-12	RPL35A
2435	217691_x_at	-1.027944444	1.44E-08	MIR6787 /// SLC16A3
2436	208072_s_at	-1.02692963	5.17E-09	DGKD
2437	213598_at	-1.026277778	2.07E-08	
2438	205061_s_at	-1.02427037	1.04E-10	EXOSC9
2439	218589_at	-1.024140741	1.10E-06	LPAR6
2440	229450_at	-1.024040741	8.48E-08	IFIT3
2441	201329_s_at	-1.023862963	1.95E-06	ETS2
2442	204768_s_at	-1.0236	1.25E-08	FEN1
2443	209318_x_at	-1.023540741	8.78E-07	PLAGL1
2444	218319_at	-1.02322963	2.24E-09	PELI1
2445	218715_at	-1.02272963	2.97E-13	UTP6
2446	209013_x_at	-1.022666667	1.07E-06	TRIO
2447	1553956_at	-1.022196296	2.75E-07	TMEM237
2448	217492_s_at	-1.0219	1.33E-10	PTEN /// PTENP1
2449	203927_at	-1.021437037	1.82E-12	NFKBIE
2450	211066_x_at	-1.021337037	3.37E-09	PCDHGA1 /// PCDHGA10 /// PCDHGA11 /// PCDHGA12 /// PCDHGA2 /// PCDHGA3 /// PCDHGA4 /// PCDHGA5 /// PCDHGA6 /// PCDHGA7 /// PCDHGA8 /// PCDHGA9 /// PCDHGB1 /// PCDHGB2 /// PCDHGB3 /// PCDHGB4 /// PCDHGB5 /// PCDHGB6 /// PCDHGB7 /// PCDHGC3 /// PCDHGC4 /// PCDHGC5
2451	232902_s_at	-1.021114815	6.14E-08	RARS2

2452	208091_s_at	-1.020940741	5.06E-13	VOPP1
2453	201302_at	-1.020318519	9.56E-06	ANXA4
2454	225712_at	-1.020262963	1.02E-10	GEMIN5
2455	210691_s_at	-1.020048148	6.75E-09	CACYBP
2456	203556_at	-1.019485185	7.24E-08	ZHX2
2457	1554577_a_at	-1.018474074	2.65E-09	PSMD10
2458	225828_at	-1.017296296	1.40E-06	DAGLB
2459	216100_s_at	-1.017133333	3.08E-07	TOR1AIP1
2460	218014_at	-1.016925926	2.59E-12	NUP85
2461	206995_x_at	-1.01617037	2.94E-07	SCARF1
2462	210218_s_at	-1.015644444	1.31E-10	SP100
2463	227400_at	-1.01562963	6.18E-06	NFIX
2464	212136_at	-1.01552963	1.00E-11	ATP2B4
2465	209397_at	-1.015433333	4.23E-10	ME2
2466	202598_at	-1.015233333	1.09E-08	S100A13
2467	221766_s_at	-1.01517037	6.50E-06	FAM46A
2468	200779_at	-1.014925926	4.58E-12	ATF4
2469	217992_s_at	-1.014866667	4.27E-12	EFHD2
2470	218472_s_at	-1.014418519	5.86E-15	PELO
2471	1559006_at	-1.01382963	1.71E-08	CTD-2124B8.2
2472	214081_at	-1.013759259	3.05E-07	PLXDC1
2473	202595_s_at	-1.013562963	4.08E-06	LEPROTL1
2474	231823_s_at	-1.013125926	3.11E-06	SH3PXD2B
2475	211747_s_at	-1.0131	4.61E-11	LSM5
2476	201874_at	-1.012833333	1.84E-09	MPZL1
2477	204384_at	-1.012596296	9.48E-09	GOLGA2
2478	212471_at	-1.012566667	6.03E-06	AVL9
2479	201700_at	-1.012544444	1.03E-08	CCND3
2480	204699_s_at	-1.012296296	1.60E-11	DIEXF
2481	219390_at	-1.01167037	1.43E-06	FKBP14
2482	1568619_s_at	-1.010737037	2.42E-10	ITPRIPL2
2483	226725_at	-1.010655556	5.19E-08	SLFN5
2484	204336_s_at	-1.010644444	6.63E-12	RGS19
2485	200858_s_at	-1.010307407	6.40E-15	RPS8 /// SNORD38B /// SNORD55
2486	210041_s_at	-1.010085185	3.53E-08	PGM3
2487	219766_at	-1.009888889	4.58E-08	B9D2
2488	208800_at	-1.009651852	1.44E-11	SRP72
2489	218798_at	-1.009107407	4.69E-06	KRI1
2490	230532_at	-1.008562963	1.23E-10	CXorf38
2491	201064_s_at	-1.008559259	1.18E-10	LOC100996696 /// PABPC4
2492	201722_s_at	-1.007925926	9.91E-11	GALNT1
2493	225578_at	-1.007755556	1.21E-11	MZT1
2494	210180_s_at	-1.007414815	2.29E-08	TRA2B

2495	227850_x_at	-1.007003704	8.32E-07	CDC42EP5
2496	226287_at	-1.006296296	1.50E-10	CCDC34
2497	218086_at	-1.006262963	2.80E-06	NPDC1
2498	202690_s_at	-1.005896296	1.50E-10	SNRPD1
2499	230747_s_at	-1.005385185	3.15E-06	TTC39C
2500	219938_s_at	-1.005155556	1.81E-06	PSTPIP2
2501	209545_s_at	-1.004607407	2.55E-09	RIPK2
2502	211302_s_at	-1.004574074	6.73E-06	PDE4B
2503	201604_s_at	-1.004537037	4.66E-10	PPP1R12A
2504	200733_s_at	-1.003666667	4.77E-07	PTP4A1
2505	203767_s_at	-1.002451852	9.65E-06	STS
2506	213375_s_at	-1.002211111	1.63E-11	N4BP2L1
2507	203538_at	-1.002133333	2.16E-12	CAMLG
2508	209203_s_at	-1.001818519	4.34E-10	BICD2
2509	201127_s_at	-1.001811111	3.01E-08	ACLY
2510	221903_s_at	-1.001714815	1.68E-09	CYLD
2511	208436_s_at	-1.001640741	2.10E-07	IRF7
2512	227458_at	-1.001633333	8.23E-07	CD274
2513	200700_s_at	-1.001462963	3.05E-07	KDELR2
2514	203209_at	-1.001285185	2.86E-10	RFC5
2515	227568_at	-1.001211111	4.27E-09	HECTD2
2516	221203_s_at	-1.000881481	2.76E-09	YEATS2
2517	221514_at	-1.0007	1.88E-10	UTP14A
2518	202864_s_at	-1.000592593	3.47E-10	SP100
2519	204452_s_at	-1.0005	7.61E-06	FZD1
2520	223159_s_at	-1.000162963	5.50E-07	NEK6
2521	209640_at	-0.999425926	1.02E-08	PML
2522	217118_s_at	-0.998811111	2.90E-09	KIAA0930
2523	211787_s_at	-0.9977	1.59E-12	EIF4A1 /// LOC101928634 /// SENP3-EIF4A1 /// SNORA48 /// SNORA67 /// SNORD10
2524	221478_at	-0.997366667	1.61E-10	BNIP3L
2525	205690_s_at	-0.996659259	4.04E-10	BUD31
2526	212621_at	-0.996381481	2.19E-12	TMEM194A
2527	221739_at	-0.995440741	3.04E-10	C19orf10
2528	210054_at	-0.994596296	2.41E-14	HAUS3
2529	204841_s_at	-0.994388889	2.07E-07	EEA1
2530	210706_s_at	-0.994155556	5.13E-07	RNF24
2531	210023_s_at	-0.994077778	3.25E-13	PCGF1
2532	209515_s_at	-0.993881481	1.50E-07	RAB27A
2533	212899_at	-0.993114815	2.65E-10	CDK19
2534	1555844_s_at	-0.99297037	2.02E-13	HNRNPM
2535	220358_at	-0.992751852	9.25E-06	BATF3

2536	217894_at	-0.992311111	1.67E-09	KCTD3
2537	212591_at	-0.992162963	5.40E-11	ARID4B /// RBM34
2538	226673_at	-0.992096296	9.54E-06	SH2D3C
2539	225032_at	-0.990514815	1.03E-12	FNDC3B /// LOC101928615
2540	235299_at	-0.989311111	1.40E-06	SLC41A2
2541	213100_at	-0.988977778	1.00E-06	UNC5B
2542	212967_x_at	-0.988966667	3.61E-10	NAP1L1
2543	216593_s_at	-0.987981481	4.30E-09	PIGC
2544	219032_x_at	-0.987585185	8.21E-06	OPN3
2545	203270_at	-0.987107407	2.85E-06	DTYMK
2546	203739_at	-0.987003704	4.20E-10	ZNF217
2547	207740_s_at	-0.98602963	6.94E-10	NUP62
2548	223253_at	-0.985677778	1.48E-08	EPDR1
2549	1552977_a_at	-0.985148148	6.84E-13	CNPY3
2550	1554600_s_at	-0.985111111	2.55E-08	LMNA
2551	234304_s_at	-0.985007407	5.45E-09	IPO11 /// IPO11-LRRC70
2552	224857_s_at	-0.984414815	8.00E-10	POLR1D
2553	203411_s_at	-0.983625926	1.06E-08	LMNA
2554	202129_s_at	-0.983437037	2.81E-07	RIOK3
2555	230786_at	-0.983074074	3.84E-06	ZCCHC8
2556	218543_s_at	-0.982677778	8.84E-10	PARP12
2557	224571_at	-0.9826	3.70E-12	IRF2BP2
2558	201980_s_at	-0.981955556	2.96E-10	RSU1
2559	1555543_a_at	-0.981811111	7.18E-07	CLCC1
2560	227210_at	-0.981077778	2.14E-07	SFMBT2
2561	224455_s_at	-0.981022222	1.38E-09	ADPGK
2562	203284_s_at	-0.980907407	7.27E-11	HS2ST1
2563	200022_at	-0.980651852	5.56E-14	RPL18
2564	227476_at	-0.980525926	1.20E-07	LPGAT1
2565	223490_s_at	-0.980437037	1.13E-09	EXOSC3
2566	208867_s_at	-0.980244444	2.27E-11	CSNK1A1
2567	214459_x_at	-0.980181481	1.69E-11	HLA-C
2568	202223_at	-0.980174074	1.56E-07	STT3A
2569	207643_s_at	-0.979492593	7.89E-10	TNFRSF1A
2570	207375_s_at	-0.979251852	2.39E-09	IL15RA
2571	222868_s_at	-0.978744444	2.66E-08	IL18BP
2572	50314_i_at	-0.978618519	6.90E-11	C20orf27
2573	222826_at	-0.978411111	1.39E-11	BLOC1S6
2574	244563_at	-0.977848148	1.88E-06	QSER1
2575	214943_s_at	-0.977355556	9.18E-10	ARID4B /// RBM34
2576	235234_at	-0.977288889	8.20E-06	PATL1
2577	221893_s_at	-0.977240741	2.40E-09	ADCK2
2578	205745_x_at	-0.976218519	1.34E-08	ADAM17

2579	225524_at	-0.975933333	3.10E-06	ANTXR2
2580	200739_s_at	-0.975540741	1.68E-09	SUMO3
2581	218399_s_at	-0.975514815	4.06E-07	CDCA4
2582	205021_s_at	-0.975192593	2.06E-07	FOXN3
2583	210092_at	-0.974888889	1.23E-09	MAGOH /// MAGOHB
2584	200852_x_at	-0.974362963	5.19E-13	GNB2
2585	211703_s_at	-0.973585185	2.30E-08	TM2D1
2586	202089_s_at	-0.973033333	9.27E-07	SLC39A6
2587	225355_at	-0.972988889	6.18E-06	NEURL1B
2588	226697_at	-0.972462963	1.04E-07	FAM114A1
2589	203799_at	-0.971640741	2.31E-07	CD302 /// LY75 /// LY75-CD302
2590	226254_s_at	-0.971314815	9.32E-15	KIAA1430
2591	212160_at	-0.97122963	5.81E-07	XPOT
2592	225646_at	-0.971055556	8.99E-07	CTSC
2593	204451_at	-0.970677778	1.32E-06	FZD1
2594	210449_x_at	-0.969951852	4.08E-07	MAPK14
2595	202185_at	-0.969944444	6.23E-10	PLOD3
2596	41220_at	-0.969788889	9.77E-13	9-Sep
2597	216384_x_at	-0.969192593	1.32E-06	LOC100506248 /// LOC728026 /// MIR1244-1 /// MIR1244-2 /// MIR1244-3 /// PTMA
2598	76897_s_at	-0.968714815	2.47E-07	FKBP15
2599	212473_s_at	-0.968481481	7.98E-06	MICAL2
2600	220770_s_at	-0.96827037	5.81E-09	ZBED8
2601	216268_s_at	-0.968103704	4.07E-08	JAG1
2602	1555832_s_at	-0.967622222	9.71E-07	KLF6
2603	227485_at	-0.967537037	9.36E-09	DDX26B
2604	206173_x_at	-0.967459259	3.17E-06	GABPB1
2605	224719_s_at	-0.967425926	2.01E-12	C12orf57
2606	211989_at	-0.967348148	5.13E-12	SMARCE1
2607	208753_s_at	-0.965962963	3.94E-08	NAP1L1
2608	202647_s_at	-0.965907407	1.75E-07	NRAS
2609	222757_s_at	-0.965407407	2.52E-08	ZAK
2610	203217_s_at	-0.965051852	1.10E-07	ST3GAL5
2611	225297_at	-0.965048148	8.01E-10	HAUS1
2612	208908_s_at	-0.96452963	1.21E-10	CAST
2613	209091_s_at	-0.964433333	1.37E-10	SH3GLB1
2614	211501_s_at	-0.964422222	1.93E-09	EIF3B
2615	AFFX-	-0.96402963	3.36E-07	STAT1
2616	226641_at	-0.963974074	2.50E-08	ANKRD44
2617	209307_at	-0.963874074	1.55E-09	SWAP70
2618	203299_s_at	-0.963866667	8.17E-08	AP1S2
2619	212290_at	-0.963792593	3.43E-07	SLC7A1
2620	228304_at	-0.963648148	3.44E-10	RBM43

2621	223272_s_at	-0.963544444	3.42E-09	NTPCR
2622	201201_at	-0.96327037	3.20E-08	CSTB
2623	1568609_s_at	-0.963207407	1.88E-08	LINC00623 /// LINC00869 /// LINC01138 /// LOC103091866
2624	212922_s_at	-0.962925926	2.15E-10	SMYD2
2625	212981_s_at	-0.9629	1.24E-07	FAM115A /// LOC100294033
2626	34449_at	-0.962207407	6.75E-06	CASP2
2627	228700_at	-0.962166667	1.10E-12	CXorf38
2628	200923_at	-0.961448148	8.54E-06	LGALS3BP
2629	211762_s_at	-0.961307407	1.37E-06	KPNA2
2630	202131_s_at	-0.961048148	1.49E-07	RIOK3
2631	1554606_at	-0.960962963	2.03E-08	CEP120
2632	229838_at	-0.960818519	8.02E-07	NUCB2
2633	203415_at	-0.960411111	3.67E-10	PDCD6
2634	210813_s_at	-0.96032963	5.81E-10	XRCC4
2635	213959_s_at	-0.960077778	4.69E-08	RPGRIP1L
2636	37028_at	-0.960014815	3.88E-06	PPP1R15A
2637	1559776_at	-0.959992593	1.14E-06	
2638	221489_s_at	-0.959825926	1.36E-06	SPRY4
2639	204453_at	-0.958974074	2.15E-08	ZNF84
2640	221923_s_at	-0.958677778	2.48E-10	NPM1
2641	232794_at	-0.958503704	3.27E-06	LOC153682
2642	227926_s_at	-0.957733333	1.76E-07	NBPF20
2643	218134_s_at	-0.957548148	1.25E-11	RBM22
2644	204735_at	-0.957188889	1.48E-08	PDE4A
2645	209784_s_at	-0.956440741	5.20E-06	JAG2
2646	232843_s_at	-0.955759259	8.18E-06	DOCK8
2647	233020_at	-0.955433333	5.77E-07	
2648	212132_at	-0.955255556	2.17E-10	LSM14A
2649	200875_s_at	-0.955188889	2.51E-10	MIR1292 /// NOP56 /// SNORD110 /// SNORD57 /// SNORD86
2650	209927_s_at	-0.954448148	6.86E-11	CHTOP
2651	205361_s_at	-0.953659259	1.25E-08	PFDN4
2652	219626_at	-0.953611111	8.33E-07	MAP7D3
2653	225677_at	-0.953403704	1.23E-11	BCAP29
2654	203553_s_at	-0.953337037	3.59E-07	MAP4K5
2655	206016_at	-0.953025926	1.07E-11	CCDC22
2656	210986_s_at	-0.952133333	2.73E-07	TPM1
2657	222036_s_at	-0.952096296	3.13E-07	MCM4
2658	208949_s_at	-0.951703704	3.06E-07	LGALS3
2659	219522_at	-0.951451852	2.53E-07	FJX1
2660	208692_at	-0.950981481	5.59E-13	RPS3
2661	201456_s_at	-0.9509	3.11E-09	BUB3

2662	221479_s_at	-0.949866667	2.54E-09	BNIP3L
2663	207467_x_at	-0.949203704	4.58E-12	CAST
2664	208821_at	-0.948277778	1.49E-11	SNRPB
2665	235508_at	-0.948218519	5.53E-06	PML
2666	200788_s_at	-0.947977778	4.16E-10	PEA15
2667	208374_s_at	-0.947955556	4.52E-14	CAPZA1
2668	219953_s_at	-0.947733333	6.76E-10	AKIP1
2669	212418_at	-0.947611111	7.82E-10	ELF1
2670	204252_at	-0.947485185	1.75E-09	CDK2
2671	200820_at	-0.947392593	6.83E-10	PSMD8
2672	212297_at	-0.946985185	1.22E-07	ATP13A3
2673	214075_at	-0.946762963	1.05E-08	NENF
2674	218817_at	-0.946659259	2.11E-07	SPCS3
2675	1554868_s_at	-0.946455556	1.58E-06	PCNP
2676	1554479_a_at	-0.946177778	3.75E-09	CARD8
2677	212204_at	-0.946040741	2.49E-13	TMEM87A
2678	203743_s_at	-0.945640741	2.69E-07	TDG
2679	212366_at	-0.944974074	2.47E-08	ZNF292
2680	226885_at	-0.944896296	1.26E-09	RNF217
2681	204208_at	-0.944848148	5.60E-07	RNGTT
2682	224761_at	-0.943533333	1.13E-14	GNA13
2683	202604_x_at	-0.943059259	1.32E-06	ADAM10
2684	202731_at	-0.942922222	7.03E-07	MIR4680 /// PDCD4
2685	1555618_s_at	-0.942792593	4.58E-08	SAE1
2686	1557558_s_at	-0.942503704	5.67E-07	MATN1-AS1
2687	222728_s_at	-0.942455556	1.54E-07	MIR1304 /// SNORA1 /// SNORA18 /// SNORA32 /// SNORA40 /// SNORA8 /// SNORD5 /// TAF1D
2688	218875_s_at	-0.942222222	9.67E-06	FBXO5
2689	225602_at	-0.941718519	9.13E-09	GLIPR2
2690	209012_at	-0.939825926	2.37E-07	TRIO
2691	223492_s_at	-0.939318519	2.12E-06	LRRFIP1
2692	208698_s_at	-0.939292593	9.16E-08	NONO
2693	221428_s_at	-0.938333333	1.97E-07	TBL1XR1
2694	217823_s_at	-0.937925926	9.62E-09	UBE2J1
2695	221570_s_at	-0.937892593	2.69E-12	METTL5
2696	208178_x_at	-0.937833333	1.14E-06	TRIO
2697	222443_s_at	-0.937711111	5.23E-10	RBM8A
2698	210101_x_at	-0.9377	8.41E-11	SH3GLB1
2699	226051_at	-0.937633333	1.25E-07	SELM
2700	201376_s_at	-0.937414815	1.54E-11	HNRNPF
2701	220731_s_at	-0.937081481	3.13E-11	NECAP2
2702	209096_at	-0.936888889	1.67E-09	UBE2V2
2703	219112_at	-0.936362963	1.82E-06	RAPGEF6

2704	1555154_a_at	-0.935881481	3.74E-07	QKI
2705	230078_at	-0.935696296	6.42E-14	RAPGEF6
2706	225908_at	-0.935585185	3.50E-12	IAH1
2707	226319_s_at	-0.935525926	5.39E-09	ALYREF
2708	226503_at	-0.935492593	1.25E-10	LOC101929336 /// RIF1
2709	210371_s_at	-0.935051852	1.69E-11	RBBP4
2710	223984_s_at	-0.934822222	3.11E-08	NUPL1
2711	226603_at	-0.934662963	1.54E-06	SAMD9L
2712	217256_x_at	-0.934592593	1.70E-11	RP3-507I15.1
2713	226215_s_at	-0.933955556	9.05E-11	KDM2B
2714	226545_at	-0.933840741	9.19E-06	CD109
2715	201603_at	-0.933311111	4.08E-11	PPP1R12A
2716	227143_s_at	-0.931755556	8.89E-13	BID
2717	243521_at	-0.930792593	5.73E-11	ZXDA
2718	225099_at	-0.930785185	1.56E-09	FBXO45
2719	225502_at	-0.930011111	8.72E-07	DOCK8
2720	204426_at	-0.929759259	5.37E-06	TMED2
2721	218473_s_at	-0.929655556	4.28E-09	COLGALT1
2722	224953_at	-0.929196296	1.69E-11	YIPF5
2723	201345_s_at	-0.928985185	3.48E-11	UBE2D2
2724	218397_at	-0.928907407	2.50E-12	FANCL
2725	211967_at	-0.928774074	9.19E-09	TMEM123
2726	229269_x_at	-0.928640741	3.16E-09	SSBP4
2727	212138_at	-0.928533333	3.70E-11	PDS5A
2728	203137_at	-0.928444444	3.71E-09	WTAP
2729	203973_s_at	-0.928274074	3.05E-06	CEBPD
2730	219119_at	-0.927766667	1.04E-07	LSM8
2731	200834_s_at	-0.927240741	3.92E-14	RPS21
2732	212722_s_at	-0.927040741	4.80E-07	JMJD6
2733	217840_at	-0.926544444	3.71E-09	DDX41
2734	205763_s_at	-0.92647037	6.18E-12	DDX18
2735	201237_at	-0.92607037	1.45E-10	CAPZA2
2736	202501_at	-0.925718519	1.37E-08	MAPRE2
2737	204928_s_at	-0.925633333	3.80E-08	SLC10A3
2738	228188_at	-0.925537037	7.46E-07	FOSL2
2739	1553679_s_at	-0.92532963	3.09E-08	VKORC1L1
2740	222427_s_at	-0.925155556	5.72E-09	LARS
2741	1554807_a_at	-0.924877778	1.91E-06	SPIRE1
2742	1557227_s_at	-0.924737037	3.69E-07	TPR
2743	214119_s_at	-0.924548148	4.49E-08	FKBP1A /// LOC101929368
2744	200942_s_at	-0.924351852	1.75E-11	HSBP1
2745	228314_at	-0.9241	7.97E-09	LOC101927933 /// LRRC8C
2746	204566_at	-0.924037037	1.22E-07	PPM1D

2747	231973_s_at	-0.923096296	5.62E-09	ANAPC1 /// LOC101930107 /// LOC285074 /// LOC730268
2748	202483_s_at	-0.922837037	5.51E-10	RANBP1
2749	209721_s_at	-0.922762963	5.92E-07	IFFO1
2750	200934_at	-0.922618519	3.27E-10	DEK
2751	201629_s_at	-0.92182963	3.80E-06	ACP1
2752	222263_at	-0.921611111	6.31E-08	SLC35E1
2753	204306_s_at	-0.9214	3.83E-07	CD151
2754	219546_at	-0.921288889	1.45E-06	BMP2K
2755	224560_at	-0.921192593	6.87E-06	TIMP2
2756	212687_at	-0.920888889	6.29E-12	LIMS1 /// LIMS3 /// LIMS3L
2757	203612_at	-0.920774074	2.86E-06	BYSL
2758	213446_s_at	-0.920622222	3.27E-08	IQGAP1
2759	225723_at	-0.920592593	1.43E-08	CCDC167
2760	227539_at	-0.919633333	1.64E-10	GNA13
2761	220974_x_at	-0.91927037	6.98E-08	SFXN3
2762	223247_at	-0.919096296	7.24E-08	MED10
2763	215485_s_at	-0.918892593	5.44E-06	ICAM1
2764	202446_s_at	-0.917814815	3.77E-06	PLSCR1
2765	1553297_a_at	-0.91777037	3.15E-08	CSF3R
2766	224649_x_at	-0.91717037	1.31E-08	CCNY
2767	242515_x_at	-0.917111111	1.03E-12	AKIP1
2768	213798_s_at	-0.915888889	3.05E-13	CAP1
2769	217965_s_at	-0.915648148	4.55E-15	SAP30BP
2770	221781_s_at	-0.915644444	1.37E-07	DNAJC10
2771	223542_at	-0.915533333	2.02E-08	ANKRD32
2772	231990_at	-0.915377778	1.33E-06	MIR6125 /// USP15
2773	224920_x_at	-0.915085185	7.98E-07	MYADM
2774	209382_at	-0.914833333	2.75E-11	POLR3C
2775	224714_at	-0.913737037	6.43E-11	NIFK
2776	201052_s_at	-0.913455556	3.12E-12	PSMF1
2777	201948_at	-0.91342963	3.90E-13	GNL2
2778	204354_at	-0.913133333	1.11E-11	POT1
2779	219031_s_at	-0.913048148	2.16E-08	NIP7
2780	222218_s_at	-0.912992593	7.31E-08	PILRA
2781	212201_at	-0.912614815	4.69E-10	ANKLE2
2782	1555501_s_at	-0.912559259	2.61E-07	RSRC1
2783	214351_x_at	-0.912407407	1.23E-12	RPL13 /// SNORD68
2784	41037_at	-0.912366667	4.53E-07	TEAD4
2785	216438_s_at	-0.912125926	4.39E-11	TMSB4X
2786	1555058_a_at	-0.912025926	9.98E-08	LPGAT1
2787	200996_at	-0.911348148	1.91E-10	ACTR3
2788	205687_at	-0.910811111	3.15E-08	UBFD1

2789	213061_s_at	-0.910462963	7.40E-12	NTAN1
2790	213687_s_at	-0.910355556	4.47E-12	RPL35A
2791	202906_s_at	-0.909914815	2.50E-08	NBN
2792	201177_s_at	-0.909814815	3.98E-12	UBA2
2793	200598_s_at	-0.909714815	9.34E-07	HSP90B1 /// MIR3652
2794	225302_at	-0.908462963	1.36E-13	TMX3
2795	203278_s_at	-0.908422222	4.50E-07	PHF21A
2796	214056_at	-0.908262963	2.27E-07	MCL1
2797	234488_s_at	-0.908140741	6.46E-07	GMCL1 /// GMCL1P1
2798	201646_at	-0.908111111	2.97E-07	SCARB2
2799	200833_s_at	-0.907622222	1.67E-10	RAP1B
2800	205298_s_at	-0.906292593	1.70E-09	BTN2A2
2801	209000_s_at	-0.905966667	4.73E-07	8-Sep
2802	225071_at	-0.904625926	1.12E-07	NUS1
2803	201608_s_at	-0.904255556	1.02E-07	PWP1
2804	203422_at	-0.903633333	1.17E-07	POLD1
2805	228372_at	-0.903518519	9.87E-06	C10orf128
2806	208308_s_at	-0.903437037	1.24E-09	GPI
2807	56197_at	-0.902974074	1.80E-10	PLSCR3 /// TMEM256-PLSCR3
2808	213455_at	-0.902611111	5.48E-09	FAM114A1
2809	212377_s_at	-0.902374074	2.09E-10	NOTCH2
2810	233268_s_at	-0.901740741	7.17E-10	CHURC1
2811	218056_at	-0.901562963	1.40E-10	BFAR
2812	205395_s_at	-0.901411111	5.65E-06	MRE11A
2813	201268_at	-0.90117037	1.11E-08	NME1-NME2 /// NME2
2814	235103_at	-0.900337037	2.36E-07	MAN2A1
2815	228250_at	-0.900218519	2.74E-09	FNIP1
2816	231727_s_at	-0.899962963	3.12E-08	MIF4GD
2817	46665_at	-0.899951852	4.31E-08	SEMA4C
2818	218130_at	-0.899081481	8.55E-11	C17orf62
2819	201315_x_at	-0.898411111	5.27E-07	IFITM2
2820	202332_at	-0.897807407	2.32E-09	CSNK1E /// CSNK1E
2821	202591_s_at	-0.897677778	5.07E-12	SSBP1
2822	222203_s_at	-0.897618519	2.62E-10	NT5C1B-RDH14 /// RDH14
2823	222837_s_at	-0.89757037	9.76E-08	NAA15
2824	219540_at	-0.897533333	1.66E-08	ZNF267
2825	229295_at	-0.897374074	1.17E-09	IL17RA
2826	221782_at	-0.89702963	2.92E-07	DNAJC10
2827	206809_s_at	-0.896537037	3.29E-11	HNRNPA3 /// HNRNPA3P1
2828	200744_s_at	-0.896366667	2.81E-08	GNB1
2829	222792_s_at	-0.895885185	1.73E-07	CCDC59
2830	218358_at	-0.895577778	6.92E-07	CRELD2
2831	222602_at	-0.894303704	7.04E-07	UBA6

2832	217196_s_at	-0.894225926	3.69E-08	CAMSAP2
2833	204689_at	-0.894166667	4.43E-07	HHEX
2834	201126_s_at	-0.893181481	1.19E-10	MGAT1
2835	60471_at	-0.893103704	1.67E-08	RIN3
2836	1552315_at	-0.893081481	1.37E-08	GIMAP1
2837	207855_s_at	-0.892933333	8.22E-09	CLCC1
2838	218092_s_at	-0.892918519	1.27E-06	AGFG1
2839	204617_s_at	-0.89287037	6.76E-10	ACD
2840	203714_s_at	-0.892859259	1.23E-08	TBCE
2841	225163_at	-0.892751852	1.15E-06	FRMD4A
2842	207196_s_at	-0.892607407	9.46E-09	TNIP1
2843	213102_at	-0.892207407	1.25E-12	ACTR3
2844	212593_s_at	-0.891366667	7.74E-10	MIR4680 /// PDCD4
2845	226032_at	-0.890844444	2.23E-12	CASP2
2846	200922_at	-0.890785185	4.91E-09	KDELR1
2847	202413_s_at	-0.890674074	6.77E-12	USP1
2848	210110_x_at	-0.890618519	5.81E-08	HNRNPH3
2849	201920_at	-0.890085185	5.23E-06	SLC20A1
2850	224906_at	-0.8896	2.80E-09	ANO6
2851	224025_s_at	-0.889488889	7.86E-08	ATG7
2852	210389_x_at	-0.889440741	2.30E-11	TUBD1
2853	226276_at	-0.889237037	5.45E-07	TMEM167A
2854	222692_s_at	-0.889211111	8.06E-09	FNDC3B /// LOC101928615
2855	212766_s_at	-0.888381481	3.87E-11	ISG20L2
2856	219258_at	-0.888351852	1.78E-08	TIPIN
2857	1554076_s_at	-0.888333333	9.38E-09	TMEM136
2858	230537_at	-0.888103704	7.38E-06	
2859	218644_at	-0.888059259	2.70E-07	PLEK2
2860	214394_x_at	-0.888014815	8.55E-10	EEF1D
2861	202162_s_at	-0.887374074	2.04E-09	CNOT8
2862	225146_at	-0.887244444	9.56E-06	FAM219A
2863	218460_at	-0.886851852	8.56E-09	HEATR2
2864	222416_at	-0.886744444	1.69E-06	ALDH18A1
2865	218156_s_at	-0.885566667	2.90E-06	TSR1
2866	203787_at	-0.885466667	2.97E-06	SSBP2
2867	225282_at	-0.885311111	2.48E-10	SMAP2
2868	226160_at	-0.885311111	4.04E-07	H6PD
2869	212018_s_at	-0.884918519	7.63E-11	RSL1D1
2870	32209_at	-0.88442963	8.04E-11	FAM89B
2871	1554627_a_at	-0.88357037	5.22E-10	ASCC1
2872	228242_at	-0.883344444	4.44E-07	N4BP2
2873	218770_s_at	-0.883314815	2.89E-07	TMEM39B

2874	201530_x_at	-0.883188889	9.16E-11	EIF4A1 /// LOC101928634 /// SENP3-EIF4A1 /// SNORA48 /// SNORA67 /// SNORD10
2875	212959_s_at	-0.882851852	4.67E-09	GNPTAB
2876	219209_at	-0.882825926	4.69E-08	IFIH1
2877	209099_x_at	-0.882785185	9.13E-07	JAG1
2878	203388_at	-0.882711111	4.01E-07	ARRB2
2879	209811_at	-0.882559259	1.27E-07	CASP2
2880	224713_at	-0.882322222	3.14E-09	NIFK
2881	201938_at	-0.882274074	1.76E-10	CDK2AP1
2882	224308_s_at	-0.882196296	3.38E-12	INTS2
2883	201014_s_at	-0.882151852	2.42E-06	PAICS
2884	201153_s_at	-0.881392593	2.62E-13	MBNL1
2885	224298_s_at	-0.880596296	1.72E-08	UBAC2
2886	205260_s_at	-0.879325926	3.72E-06	ACYP1
2887	205803_s_at	-0.879055556	2.63E-08	TRPC1
2888	201875_s_at	-0.878181481	1.88E-06	MPZL1
2889	208549_x_at	-0.878037037	4.51E-12	PTMA
2890	212586_at	-0.877777778	3.66E-10	CAST
2891	218708_at	-0.877711111	1.04E-09	NXT1
2892	201589_at	-0.877388889	3.27E-10	SMC1A
2893	208980_s_at	-0.877337037	8.47E-14	UBC
2894	224452_s_at	-0.876981481	4.07E-10	FAM220A
2895	202980_s_at	-0.876977778	2.15E-09	SIAH1
2896	206499_s_at	-0.876740741	2.43E-07	RCC1
2897	227144_at	-0.876277778	4.12E-06	KIAA0930
2898	212048_s_at	-0.87627037	4.28E-07	YARS
2899	218537_at	-0.87582963	5.38E-07	HCFC1R1
2900	1558699_a_at	-0.875614815	1.47E-07	HERPUD2
2901	217313_at	-0.875433333	7.88E-09	AC004692.5
2902	64432_at	-0.875174074	7.02E-08	MAPKAPK5-AS1
2903	212262_at	-0.875025926	1.96E-11	QKI
2904	228654_at	-0.874666667	8.98E-08	SPIN4
2905	234512_x_at	-0.874425926	4.43E-07	RP3-486D24.1
2906	57715_at	-0.874377778	3.15E-08	CALHM2
2907	218163_at	-0.874307407	2.11E-08	MCTS1
2908	209358_at	-0.8735	3.57E-11	TAF11
2909	239835_at	-0.872862963	4.35E-06	KBTBD8
2910	208627_s_at	-0.872774074	2.69E-08	YBX1
2911	202396_at	-0.872366667	6.96E-09	TCERG1
2912	211543_s_at	-0.872240741	2.34E-06	GRK6
2913	221918_at	-0.871214815	7.09E-10	CDK17
2914	200705_s_at	-0.870818519	2.30E-11	EEF1B2 /// SNORA41
2915	209251_x_at	-0.870722222	1.49E-09	TUBA1C

2916	201723_s_at	-0.869911111	1.66E-09	GALNT1
2917	200076_s_at	-0.869133333	8.68E-11	KXD1
2918	213470_s_at	-0.868722222	2.77E-06	HNRNPH1
2919	218379_at	-0.868655556	2.42E-08	RBM7
2920	213237_at	-0.868503704	1.30E-09	KNOP1
2921	203986_at	-0.867425926	5.93E-07	FAM47E /// FAM47E-STBD1 /// STBD1
2922	217197_x_at	-0.867385185	9.08E-06	N4BP2L1
2923	209495_at	-0.867318519	1.38E-08	CEP250
2924	208696_at	-0.866874074	1.94E-08	CCT5
2925	223186_at	-0.866314815	5.71E-09	TMEM189 /// TMEM189-UBE2V1 /// UBE2V1
2926	205717_x_at	-0.865566667	3.71E-08	PCDHGA1 /// PCDHGA10 /// PCDHGA11 /// PCDHGA12 /// PCDHGA2 /// PCDHGA3 /// PCDHGA4 /// PCDHGA5 /// PCDHGA6 /// PCDHGA7 /// PCDHGA8 /// PCDHGA9 /// PCDHGB1 /// PCDHGB2 /// PCDHGB3 /// PCDHGB4 /// PCDHGB5 /// PCDHGB6 /// PCDHGB7 /// PCDHGC3 /// PCDHGC4 /// PCDHGC5
2927	212248_at	-0.865518519	1.37E-11	MTDH
2928	213911_s_at	-0.865366667	1.33E-09	H2AFZ
2929	212860_at	-0.864514815	5.59E-07	ZDHHC18
2930	236487_at	-0.864311111	9.45E-09	LOC101927137 /// SCLT1
2931	235085_at	-0.863944444	1.25E-07	SGK223
2932	223110_at	-0.863874074	2.51E-10	KIAA1429
2933	211946_s_at	-0.863518519	2.26E-11	PRRC2C
2934	222182_s_at	-0.863396296	2.80E-12	CNOT2
2935	213572_s_at	-0.86322963	1.23E-08	SERPINB1
2936	226949_at	-0.862944444	1.90E-06	GOLGA3
2937	214716_at	-0.86257037	2.50E-06	BMP2K
2938	203550_s_at	-0.862351852	1.25E-06	FAM189B
2939	218905_at	-0.861762963	1.23E-11	INTS8
2940	203194_s_at	-0.861544444	1.58E-09	NUP98
2941	215001_s_at	-0.861496296	7.36E-09	GLUL
2942	205013_s_at	-0.860477778	3.26E-07	ADORA2A /// SPECC1L-ADORA2A
2943	205463_s_at	-0.860362963	4.37E-08	PDGFA
2944	232007_at	-0.860288889	8.41E-07	AGPAT5
2945	229067_at	-0.859925926	6.20E-07	
2946	209257_s_at	-0.858885185	6.48E-07	SMC3
2947	213373_s_at	-0.858603704	1.48E-09	CASP8
2948	218295_s_at	-0.858566667	2.08E-07	NUP50
2949	221517_s_at	-0.858014815	7.00E-10	MED17
2950	207713_s_at	-0.8576	7.61E-06	RBCK1
2951	218943_s_at	-0.857014815	5.69E-06	DDX58
2952	209422_at	-0.856922222	3.59E-10	PHF20

2953	217948_at	-0.8566	2.69E-09	FAM127B
2954	200959_at	-0.856462963	5.55E-09	FUS
2955	225310_at	-0.856340741	1.02E-08	LOC101928747 /// RBMX /// SNORD61
2956	217946_s_at	-0.85617037	2.72E-10	SAE1
2957	226910_at	-0.855974074	8.30E-08	COMMD2
2958	225837_at	-0.855011111	2.70E-10	RHNO1
2959	222026_at	-0.854977778	6.01E-06	RBM3
2960	209187_at	-0.854596296	4.78E-09	DR1
2961	212878_s_at	-0.854592593	5.33E-06	KLC1
2962	217502_at	-0.854577778	6.36E-06	IFIT2
2963	225088_at	-0.854351852	6.84E-06	FOPNL
2964	214093_s_at	-0.854318519	1.59E-08	FUBP1
2965	212135_s_at	-0.854274074	9.55E-09	ATP2B4
2966	221473_x_at	-0.853451852	2.87E-10	SERINC3
2967	205170_at	-0.853325926	1.34E-06	STAT2
2968	227786_at	-0.852992593	4.24E-07	MED30
2969	208757_at	-0.851559259	4.63E-08	TMED9
2970	204747_at	-0.850818519	6.10E-06	IFIT3
2971	235056_at	-0.850685185	3.08E-07	ETV6
2972	218696_at	-0.850514815	1.95E-09	EIF2AK3
2973	200050_at	-0.850481481	1.16E-10	ZNF146
2974	1554678_s_at	-0.850288889	5.79E-07	HNRNPDL
2975	200069_at	-0.849955556	1.81E-06	SART3
2976	227534_at	-0.849785185	1.90E-09	AAED1
2977	230036_at	-0.849048148	4.28E-06	SAMD9L
2978	222634_s_at	-0.848637037	5.68E-08	TBL1XR1
2979	219506_at	-0.848392593	1.66E-07	C1orf54
2980	205716_at	-0.84812963	2.30E-06	SLC25A40
2981	212781_at	-0.848118519	3.94E-09	RBBP6
2982	214801_at	-0.848107407	2.14E-08	TOR1AIP2
2983	222418_s_at	-0.847718519	2.60E-11	TMEM43
2984	203847_s_at	-0.847448148	1.71E-11	AKAP8
2985	223017_at	-0.847274074	1.25E-10	TXNDC12
2986	208620_at	-0.846614815	1.54E-09	PCBP1
2987	208788_at	-0.84642963	7.87E-08	ELOVL5
2988	212060_at	-0.846318519	3.13E-09	U2SURP
2989	223269_at	-0.845796296	1.06E-08	POLR3GL
2990	218252_at	-0.845644444	9.05E-09	CKAP2
2991	205527_s_at	-0.845551852	1.49E-06	GEMIN4
2992	224698_at	-0.845266667	4.60E-08	ESYT2
2993	204053_x_at	-0.845088889	9.92E-09	PTEN
2994	202557_at	-0.845048148	1.23E-08	HSPA13
2995	227518_at	-0.844885185	1.42E-09	SLC35E1

2996	204559_s_at	-0.843788889	2.24E-09	LSM7
2997	208764_s_at	-0.843451852	6.69E-11	ATP5G2
2998	211931_s_at	-0.843388889	4.68E-10	HNRNPA3 /// HNRNPA3P1
2999	204949_at	-0.842896296	3.44E-10	ICAM3
3000	213554_s_at	-0.842307407	5.85E-07	CDV3
3001	222158_s_at	-0.842162963	2.97E-08	DESI2
3002	AFFX-	-0.841940741	2.36E-08	ACTB
3003	227649_s_at	-0.841907407	2.16E-07	SRGAP2 /// SRGAP2B /// SRGAP2C
3004	223043_at	-0.841877778	5.07E-11	EMC4
3005	226801_s_at	-0.841425926	1.11E-12	AIDA
3006	213086_s_at	-0.841396296	7.71E-10	CSNK1A1
3007	210573_s_at	-0.841137037	5.83E-09	POLR3C
3008	201751_at	-0.840918519	8.07E-11	JOSD1
3009	200812_at	-0.840833333	5.97E-11	CCT7
3010	1555579_s_at	-0.84017037	5.34E-07	PTPRM
3011	225021_at	-0.840114815	5.26E-06	ZNF532
3012	211769_x_at	-0.839481481	1.68E-10	SERINC3
3013	202899_s_at	-0.83907037	1.21E-08	SRSF3
3014	218323_at	-0.838762963	5.60E-10	RHOT1
3015	235003_at	-0.838507407	1.20E-06	UHMK1
3016	209034_at	-0.838444444	1.04E-07	PNRC1
3017	218036_x_at	-0.838351852	5.54E-06	NMD3
3018	219296_at	-0.838288889	2.53E-09	ZDHHC13
3019	224569_s_at	-0.838014815	4.96E-11	IRF2BP2
3020	203028_s_at	-0.837855556	1.30E-07	CYBA
3021	209704_at	-0.837162963	1.07E-08	MTF2
3022	207724_s_at	-0.836514815	3.06E-06	SPAST
3023	207338_s_at	-0.836066667	2.99E-08	ZNF200
3024	209463_s_at	-0.836051852	3.31E-08	TAF12
3025	201079_at	-0.835855556	1.33E-07	SYNGR2
3026	241342_at	-0.835662963	4.71E-09	TMEM65
3027	220688_s_at	-0.835522222	2.23E-09	MRTO4
3028	218288_s_at	-0.835477778	7.90E-11	CCDC90B
3029	225152_at	-0.835422222	6.99E-09	ZNF622
3030	208775_at	-0.835081481	3.86E-13	XPO1
3031	201773_at	-0.834955556	2.12E-10	ADNP
3032	218041_x_at	-0.834281481	1.11E-06	SLC38A2
3033	218996_at	-0.834225926	2.24E-06	TFPT
3034	234926_s_at	-0.83417037	2.87E-10	RTFDC1
3035	225447_at	-0.833959259	5.80E-07	GPD2
3036	222395_s_at	-0.833788889	9.59E-09	UBE2Z
3037	212698_s_at	-0.833744444	1.82E-07	10-Sep
3038	208478_s_at	-0.833203704	7.86E-09	BAX

3039	200019_s_at	-0.832922222	3.07E-13	FAU
3040	202911_at	-0.8326	5.76E-10	MSH6
3041	201004_at	-0.832514815	2.30E-10	SSR4
3042	230326_s_at	-0.832285185	1.52E-06	C11orf73
3043	211561_x_at	-0.831922222	9.38E-07	MAPK14
3044	212773_s_at	-0.8319	7.60E-10	TOMM20
3045	222527_s_at	-0.831003704	3.74E-09	RBM22
3046	216080_s_at	-0.830966667	1.35E-06	FADS3
3047	210466_s_at	-0.830511111	2.06E-11	SERBP1
3048	201179_s_at	-0.830233333	3.85E-09	GNAI3
3049	208447_s_at	-0.830207407	6.31E-07	PRPS1
3050	235678_at	-0.830055556	5.01E-06	GM2A
3051	208837_at	-0.829892593	8.68E-07	TMED3
3052	1553034_at	-0.828196296	3.72E-07	SDCCAG8
3053	226757_at	-0.827944444	2.92E-06	IFIT2
3054	226329_s_at	-0.827340741	2.34E-08	MITD1
3055	208879_x_at	-0.825988889	2.14E-06	PRPF6
3056	219520_s_at	-0.825859259	4.18E-07	WWC3
3057	201784_s_at	-0.825592593	4.67E-11	C11orf58
3058	225530_at	-0.825374074	7.55E-11	MOB3A
3059	201232_s_at	-0.824640741	5.26E-09	PSMD13
3060	223065_s_at	-0.824477778	6.85E-12	STARD3NL
3061	205042_at	-0.824381481	6.45E-07	GNE
3062	223271_s_at	-0.8239	1.92E-08	CTDSPL2
3063	212058_at	-0.8237	1.15E-09	U2SURP
3064	209263_x_at	-0.822514815	4.18E-11	TSPAN4
3065	211697_x_at	-0.822303704	1.50E-06	PNO1
3066	210470_x_at	-0.821922222	2.14E-07	NONO
3067	219553_at	-0.821825926	3.44E-06	NME7
3068	203275_at	-0.821207407	9.79E-09	IRF2
3069	218354_at	-0.821196296	9.92E-09	TRAPPC2L
3070	1558279_a_at	-0.821088889	9.20E-06	KDSR
3071	218889_at	-0.820962963	3.01E-10	NOC3L
3072	207945_s_at	-0.820922222	1.08E-08	CSNK1D
3073	204353_s_at	-0.820877778	9.08E-10	POT1
3074	218013_x_at	-0.820811111	6.27E-08	DCTN4
3075	204449_at	-0.820677778	2.40E-06	PDCL
3076	203564_at	-0.820240741	3.75E-09	FANCG
3077	204862_s_at	-0.819737037	6.71E-08	NME3
3078	224855_at	-0.819688889	8.52E-08	MIR6741 /// PYCR2
3079	209339_at	-0.819637037	4.95E-07	SIAH2
3080	212846_at	-0.819111111	2.47E-09	RRP1B
3081	202442_at	-0.818762963	1.31E-11	AP3S1

3082	201277_s_at	-0.818548148	7.64E-09	HNRNPAB
3083	218236_s_at	-0.818396296	9.07E-08	PRKD3
3084	201029_s_at	-0.818185185	1.01E-07	CD99
3085	225878_at	-0.817966667	3.00E-06	KIF1B
3086	201801_s_at	-0.817959259	3.10E-06	SLC29A1
3087	1558254_s_at	-0.817644444	2.96E-06	SRPK2
3088	210858_x_at	-0.817518519	9.50E-07	ATM
3089	218595_s_at	-0.817185185	9.49E-08	HEATR1
3090	201770_at	-0.816996296	1.63E-09	SNRPA
3091	201970_s_at	-0.816811111	2.44E-10	NASP
3092	218094_s_at	-0.816777778	8.32E-07	DBNDD2 /// SYS1 /// SYS1-DBNDD2
3093	218576_s_at	-0.8167	6.21E-09	DUSP12
3094	200073_s_at	-0.816459259	7.27E-12	HNRNPD
3095	214198_s_at	-0.815981481	3.81E-07	DGCR2
3096	234915_s_at	-0.815955556	1.64E-06	DENR
3097	224654_at	-0.815707407	8.24E-10	DDX21
3098	227068_at	-0.815640741	8.76E-08	PGK1
3099	1555760_a_at	-0.815522222	2.94E-10	RBM15
3100	203518_at	-0.814996296	7.76E-10	LYST
3101	209922_at	-0.814685185	5.23E-06	BRAP
3102	223133_at	-0.814485185	1.05E-07	TMEM14B
3103	1553976_a_at	-0.814385185	2.29E-06	DPCD
3104	220018_at	-0.814162963	1.33E-06	CBLL1
3105	203481_at	-0.813737037	2.75E-08	FAM178A
3106	225076_s_at	-0.8129	2.22E-10	ZNFX1
3107	226887_at	-0.812603704	2.87E-12	HSPA14
3108	203316_s_at	-0.812355556	1.22E-09	SNRPE
3109	225579_at	-0.81227037	4.12E-08	PQLC3
3110	212271_at	-0.812040741	6.76E-08	MAPK1
3111	217457_s_at	-0.811911111	8.43E-06	RAP1GDS1
3112	213044_at	-0.811833333	2.35E-10	ROCK1
3113	208750_s_at	-0.811214815	3.68E-08	ARF1 /// MIR3620
3114	238604_at	-0.81047037	9.92E-07	
3115	208795_s_at	-0.810244444	7.04E-08	MCM7
3116	203253_s_at	-0.810037037	1.20E-08	PPIP5K2
3117	244396_at	-0.810025926	1.21E-06	G3BP1
3118	200840_at	-0.809848148	1.25E-10	KARS
3119	227200_at	-0.809751852	3.36E-10	ETV3
3120	228185_at	-0.809696296	3.04E-08	ZNF25
3121	209023_s_at	-0.8094	2.88E-10	STAG2
3122	224701_at	-0.809392593	5.16E-07	PARP14
3123	227001_at	-0.808966667	1.09E-07	NIPAL2
3124	219275_at	-0.808796296	9.83E-07	PDCD5

3125	225159_s_at	-0.808581481	6.47E-12	ELK4
3126	212371_at	-0.8084	4.85E-09	DESI2
3127	202536_at	-0.808385185	1.82E-07	CHMP2B
3128	200599_s_at	-0.80837037	1.36E-07	HSP90B1 /// MIR3652
3129	228007_at	-0.80812963	1.22E-08	CEP85L
3130	202946_s_at	-0.807962963	4.23E-07	BTBD3
3131	211256_x_at	-0.806751852	2.97E-07	BTN2A1
3132	234299_s_at	-0.806648148	1.58E-06	NIN
3133	202329_at	-0.806466667	7.52E-09	CSK
3134	202364_at	-0.805996296	4.23E-08	MXI1
3135	206445_s_at	-0.805855556	1.89E-08	PRMT1
3136	231769_at	-0.805759259	5.10E-06	FBXO6
3137	228963_at	-0.805733333	6.89E-08	RSBN1L
3138	209619_at	-0.804896296	1.68E-07	CD74
3139	203653_s_at	-0.804488889	2.19E-07	COIL
3140	210588_x_at	-0.804481481	1.97E-08	HNRNPH3
3141	209066_x_at	-0.804340741	8.55E-08	UQCRB
3142	202596_at	-0.80402963	1.26E-09	ENSA
3143	225073_at	-0.803466667	4.48E-08	PPHLN1
3144	203552_at	-0.803459259	5.31E-07	MAP4K5
3145	224826_at	-0.803366667	1.50E-08	GPCPD1
3146	1554351_a_at	-0.802992593	6.25E-07	TIPRL
3147	200975_at	-0.802948148	3.99E-12	PPT1
3148	218231_at	-0.801103704	1.71E-09	NAGK
3149	228385_at	-0.800837037	2.14E-08	DDX59
3150	32502_at	-0.800511111	7.27E-06	GDPD5
3151	227268_at	-0.800481481	3.21E-06	RNFT1
3152	225037_at	-0.800385185	4.40E-07	SLC35C2
3153	210564_x_at	-0.800274074	2.73E-06	CFLAR
3154	208781_x_at	-0.800188889	8.70E-08	SNX3
3155	213699_s_at	-0.800111111	1.71E-10	YWHAQ
3156	209572_s_at	-0.800107407	3.75E-09	EED
3157	223090_x_at	-0.799866667	3.91E-09	VEZT
3158	211098_x_at	-0.7996	6.43E-08	TMCO1
3159	200886_s_at	-0.799448148	1.22E-09	PGAM1
3160	205812_s_at	-0.799292593	9.47E-09	TMED9
3161	217826_s_at	-0.7992	1.94E-06	UBE2J1
3162	236297_at	-0.798981481	3.23E-06	PLXDC2
3163	218598_at	-0.798814815	4.84E-08	RINT1
3164	216565_x_at	-0.798425926	2.16E-08	RP4-781L3.1
3165	225785_at	-0.798074074	1.65E-07	REEP3
3166	223443_s_at	-0.797985185	8.96E-08	AMZ2P1
3167	219544_at	-0.797966667	8.71E-07	BORA

3168	241706_at	-0.797851852	3.60E-07	CPNE8
3169	226963_at	-0.797348148	2.42E-11	BTF3L4
3170	205084_at	-0.7969	3.03E-06	BCAP29
3171	212202_s_at	-0.796581481	7.08E-11	TMEM87A
3172	209882_at	-0.796477778	8.24E-07	RIT1
3173	201181_at	-0.796411111	1.12E-09	GNAI3
3174	208897_s_at	-0.7963	7.40E-09	DDX18
3175	218660_at	-0.795592593	5.59E-06	DYSF
3176	227152_at	-0.79547037	2.12E-07	KIAA1551
3177	223176_at	-0.795337037	1.56E-08	KCTD20
3178	201379_s_at	-0.795266667	5.26E-09	TPD52L2
3179	224944_at	-0.795092593	1.86E-07	TMPO
3180	226600_at	-0.7946	1.07E-07	TMTC3
3181	205296_at	-0.794092593	4.35E-06	RBL1
3182	231862_at	-0.793962963	5.94E-07	CBX5
3183	210802_s_at	-0.793840741	4.56E-07	DIMT1
3184	217097_s_at	-0.79377037	5.35E-06	PHTF2
3185	208612_at	-0.793759259	1.39E-07	PDIA3
3186	209306_s_at	-0.793251852	5.43E-06	SWAP70
3187	208875_s_at	-0.792677778	2.59E-07	PAK2
3188	222976_s_at	-0.792507407	6.98E-12	TPM3
3189	221193_s_at	-0.792437037	1.18E-07	ZCCHC10
3190	201111_at	-0.792259259	9.99E-06	CSE1L
3191	203956_at	-0.792174074	3.47E-07	MORC2
3192	201305_x_at	-0.791725926	8.68E-10	ANP32B
3193	200037_s_at	-0.79157037	7.62E-12	CBX3
3194	200662_s_at	-0.791555556	8.48E-10	TOMM20
3195	211977_at	-0.791374074	1.63E-07	GPR107
3196	213151_s_at	-0.791218519	2.75E-12	7-Sep
3197	201478_s_at	-0.790933333	1.34E-06	DKC1 /// MIR664B /// SNORA56
3198	212875_s_at	-0.790666667	1.51E-09	C2CD2
3199	201746_at	-0.790181481	7.86E-08	TP53
3200	207630_s_at	-0.790081481	7.90E-06	CREM
3201	209140_x_at	-0.789344444	1.67E-10	HLA-B
3202	206562_s_at	-0.789137037	3.63E-08	CSNK1A1
3203	223164_at	-0.788377778	6.27E-07	CCM2
3204	203715_at	-0.788333333	5.42E-06	TBCE
3205	203582_s_at	-0.78812963	1.31E-08	RAB4A /// SPHAR
3206	223019_at	-0.787822222	4.19E-06	FAM129B
3207	238417_at	-0.787640741	2.53E-07	PGM2L1
3208	225866_at	-0.787433333	5.73E-07	RPF2
3209	229181_s_at	-0.787244444	9.55E-06	HAUS2
3210	201040_at	-0.786792593	2.89E-09	GNAI2

3211	205281_s_at	-0.786762963	2.63E-08	PIGA
3212	202633_at	-0.786462963	4.26E-09	TOPBP1
3213	213341_at	-0.7864	5.22E-08	FEM1C
3214	228799_at	-0.785914815	3.31E-08	NCK1-AS1
3215	200593_s_at	-0.785437037	7.57E-09	HNRNPU
3216	202049_s_at	-0.784911111	3.58E-07	ZMYM4
3217	226711_at	-0.784655556	3.22E-11	FOXN2
3218	213750_at	-0.784618519	6.18E-07	RSL1D1
3219	226349_at	-0.784411111	1.11E-06	C12orf45
3220	211594_s_at	-0.784092593	1.98E-07	MRPL9
3221	215009_s_at	-0.784092593	5.69E-06	THAP9-AS1
3222	1552618_at	-0.783385185	2.16E-08	STX6
3223	213754_s_at	-0.782766667	2.72E-10	PAIP1
3224	225128_at	-0.7825	4.30E-06	KDELC2
3225	213168_at	-0.781996296	2.69E-11	SP3
3226	224922_at	-0.781359259	2.50E-07	CSNK2A2
3227	224859_at	-0.781281481	7.69E-06	CD276
3228	227698_s_at	-0.780444444	1.28E-06	RAB40C
3229	200736_s_at	-0.780359259	7.29E-09	GPX1
3230	219294_at	-0.780218519	2.01E-06	CENPQ
3231	209953_s_at	-0.779962963	1.91E-10	CDC37
3232	214882_s_at	-0.779944444	1.39E-09	MIR636 /// SRSF2
3233	212245_at	-0.779477778	3.69E-11	MCFD2
3234	229410_at	-0.779040741	1.35E-10	SLC35E1
3235	201023_at	-0.778825926	2.10E-09	TAF7
3236	212104_s_at	-0.77877037	1.04E-08	RBFOX2
3237	224807_at	-0.778585185	4.53E-08	GRAMD1A
3238	209067_s_at	-0.77842963	4.81E-09	HNRNPDL
3239	227558_at	-0.777988889	1.73E-09	CBX4
3240	219029_at	-0.7772	1.18E-06	C5orf28
3241	202211_at	-0.777062963	1.60E-08	ARFGAP3
3242	200787_s_at	-0.776681481	1.33E-06	PEA15
3243	207079_s_at	-0.776588889	9.31E-06	MED6
3244	37462_i_at	-0.776511111	2.94E-06	SF3A2
3245	202697_at	-0.776055556	4.23E-06	NUDT21
3246	222279_at	-0.776025926	1.93E-06	HLA-F-AS1
3247	217865_at	-0.77592963	5.96E-11	RNF130
3248	202439_s_at	-0.775818519	1.03E-07	IDS
3249	202918_s_at	-0.775777778	1.15E-06	HSPE1-MOB4 /// MOB4
3250	200652_at	-0.775592593	2.12E-11	SSR2
3251	218256_s_at	-0.775566667	8.24E-10	NUP54
3252	218627_at	-0.77502963	1.57E-06	DRAM1
3253	222624_s_at	-0.775007407	3.33E-08	ZNF639

3254	223847_s_at	-0.774581481	7.59E-06	ERGIC1
3255	224934_at	-0.774488889	1.83E-08	YIPF5
3256	201275_at	-0.773718519	1.52E-09	FDPS
3257	216383_at	-0.773622222	3.64E-07	RPL18AP16 /// RPL18AP16
3258	228468_at	-0.77342963	5.47E-06	MASTL
3259	203298_s_at	-0.773314815	1.82E-06	JARID2
3260	201699_at	-0.77317037	1.14E-08	PSMC6
3261	209471_s_at	-0.77297037	1.13E-08	FNTA
3262	218838_s_at	-0.772325926	1.07E-11	TTC31
3263	209051_s_at	-0.771814815	1.51E-08	RALGDS
3264	201343_at	-0.771811111	7.57E-11	UBE2D2
3265	1563111_a_at	-0.771455556	9.26E-06	PIGX
3266	202603_at	-0.770922222	7.91E-10	ADAM10
3267	225545_at	-0.770685185	8.43E-09	EEF2K /// LOC101930123
3268	210104_at	-0.770340741	3.48E-07	MED6
3269	217301_x_at	-0.769762963	6.81E-07	RBBP4
3270	1552472_a_at	-0.769314815	4.62E-07	ACAP2
3271	207000_s_at	-0.769277778	1.36E-09	PPP3CC
3272	224893_at	-0.769207407	5.35E-09	ATL3
3273	204797_s_at	-0.769192593	3.28E-07	EML1
3274	235051_at	-0.769033333	2.77E-08	CCDC50
3275	202009_at	-0.768866667	1.77E-06	TWF2
3276	221725_at	-0.768844444	7.19E-11	WASF2
3277	226642_s_at	-0.768544444	4.28E-09	NUDCD2
3278	214193_s_at	-0.768407407	6.75E-07	DIEXF
3279	223001_at	-0.768325926	7.61E-07	OSTC
3280	201872_s_at	-0.768174074	7.34E-08	ABCE1
3281	225050_at	-0.768162963	1.41E-09	ZNF512
3282	214271_x_at	-0.768137037	5.66E-11	RPL12
3283	1568618_a_at	-0.767614815	6.62E-08	GALNT1
3284	38269_at	-0.767522222	3.65E-13	PRKD2
3285	201705_at	-0.767318519	2.70E-08	PSMD7
3286	244783_at	-0.767274074	2.85E-06	YAF2
3287	223389_s_at	-0.76702963	7.49E-08	ZNF581
3288	209050_s_at	-0.766403704	8.98E-07	RALGDS
3289	223189_x_at	-0.765955556	3.70E-11	KMT2E
3290	223675_s_at	-0.76562963	7.38E-10	VEZT
3291	1556006_s_at	-0.765551852	1.51E-06	CSNK1A1
3292	202268_s_at	-0.76542963	1.22E-09	NAE1
3293	204700_x_at	-0.764955556	7.61E-08	DIEXF
3294	1558201_s_at	-0.764588889	4.80E-11	SLC4A1AP
3295	212263_at	-0.764207407	4.43E-09	QKI
3296	224744_at	-0.7641	6.49E-07	IMPAD1

3297	217880_at	-0.764081481	1.75E-08	CDC27
3298	206687_s_at	-0.763722222	2.30E-07	PTPN6
3299	212168_at	-0.763288889	1.50E-09	RBM12
3300	212796_s_at	-0.763048148	2.73E-09	TBC1D2B
3301	212203_x_at	-0.762985185	1.10E-08	IFITM3
3302	210460_s_at	-0.761974074	5.48E-08	PSMD4
3303	203138_at	-0.761937037	1.41E-10	HAT1
3304	218513_at	-0.761777778	2.25E-06	TMA16
3305	228328_at	-0.761362963	1.94E-07	KLHL28
3306	200090_at	-0.761288889	1.01E-09	FNTA
3307	200687_s_at	-0.761233333	1.05E-09	SF3B3
3308	209258_s_at	-0.761185185	4.65E-09	SMC3
3309	1555278_a_at	-0.760611111	9.18E-07	CKAP5
3310	220032_at	-0.760333333	2.46E-06	CPED1
3311	201263_at	-0.760288889	1.41E-07	TARS
3312	225412_at	-0.759677778	1.11E-06	TMEM87B
3313	209127_s_at	-0.759618519	2.18E-07	SART3
3314	65884_at	-0.759437037	8.91E-09	MAN1B1
3315	219788_at	-0.759403704	2.00E-06	PILRA
3316	218405_at	-0.75912963	6.61E-07	ABT1
3317	1553528_a_at	-0.75902963	7.80E-07	TAF5
3318	218593_at	-0.758881481	1.04E-07	RBM28
3319	218374_s_at	-0.758366667	2.62E-08	C12orf4
3320	212569_at	-0.757555556	1.34E-06	SMCHD1
3321	203530_s_at	-0.757548148	1.41E-09	STX4
3322	218936_s_at	-0.757440741	9.36E-07	CCDC59
3323	201318_s_at	-0.756081481	1.12E-09	MYL12A /// MYL12B
3324	211284_s_at	-0.756025926	8.67E-07	GRN
3325	212010_s_at	-0.75552963	4.75E-09	CDV3
3326	234997_x_at	-0.755248148	3.38E-06	RP11-488L18.10
3327	201128_s_at	-0.754674074	3.90E-07	ACLY
3328	220924_s_at	-0.754459259	5.26E-06	SLC38A2
3329	202290_at	-0.754288889	1.75E-06	PDAP1
3330	218777_at	-0.754107407	4.76E-06	REEP4
3331	201356_at	-0.753855556	4.36E-09	SF3A1
3332	226315_at	-0.753748148	1.43E-10	ZNF830
3333	203620_s_at	-0.753614815	1.08E-09	FCHSD2
3334	208896_at	-0.753551852	7.26E-10	DDX18
3335	233936_s_at	-0.753051852	9.29E-10	GGNBP2
3336	212923_s_at	-0.752977778	2.49E-08	PXDC1
3337	218603_at	-0.752755556	7.07E-10	HECA
3338	223650_s_at	-0.751440741	7.58E-08	NRBF2
3339	204127_at	-0.751059259	1.26E-07	RFC3

3340	209831_x_at	-0.750918519	8.71E-10	DNASE2
3341	208393_s_at	-0.750318519	9.24E-08	RAD50
3342	225832_s_at	-0.750292593	6.04E-11	DAGLB
3343	1554417_s_at	-0.750266667	2.32E-06	APH1A
3344	224929_at	-0.749918519	7.89E-08	TMEM173
3345	201457_x_at	-0.749774074	2.73E-07	BUB3
3346	226601_at	-0.749514815	2.43E-07	SLC30A7
3347	209748_at	-0.749359259	6.76E-08	SPAST
3348	231869_at	-0.749318519	7.94E-06	KIAA1586
3349	223993_s_at	-0.74922963	1.18E-06	CNIH4
3350	212221_x_at	-0.749140741	1.37E-07	IDS
3351	203822_s_at	-0.748903704	1.31E-10	ELF2
3352	202190_at	-0.748340741	1.20E-08	CSTF1
3353	212769_at	-0.7482	2.90E-07	TLE3
3354	211152_s_at	-0.747955556	2.96E-09	HTRA2
3355	224656_s_at	-0.747907407	3.12E-11	LUZP6 /// MTPN
3356	32541_at	-0.747607407	3.20E-10	PPP3CC
3357	218954_s_at	-0.747296296	9.90E-07	BRF2
3358	209069_s_at	-0.746822222	3.06E-09	H3F3A /// H3F3B /// MIR4738
3359	217795_s_at	-0.7463	1.15E-08	TMEM43
3360	208803_s_at	-0.746040741	6.40E-09	SRP72
3361	217747_s_at	-0.745962963	1.51E-11	RPS9
3362	226251_at	-0.745922222	9.43E-08	ASXL2
3363	203113_s_at	-0.745781481	1.06E-10	EEF1D
3364	205588_s_at	-0.745696296	8.76E-06	FGFR1OP
3365	201406_at	-0.745274074	1.81E-10	RPL36A /// RPL36A-HNRNPH2
3366	208688_x_at	-0.745244444	1.11E-07	EIF3B
3367	200797_s_at	-0.744811111	1.39E-06	MCL1
3368	213142_x_at	-0.74467037	1.28E-06	GSAP
3369	200738_s_at	-0.744644444	1.84E-09	PGK1
3370	212191_x_at	-0.744422222	8.88E-11	RPL13 /// SNORD68
3371	219158_s_at	-0.744355556	4.45E-10	NAA15
3372	206989_s_at	-0.744155556	1.41E-07	SCAF11
3373	204618_s_at	-0.744088889	3.06E-11	GABPB1
3374	209628_at	-0.744074074	1.88E-08	NXT2
3375	224578_at	-0.744	8.88E-09	RCC2
3376	210224_at	-0.743825926	4.43E-08	MR1
3377	233655_s_at	-0.743622222	9.79E-07	HAUS6
3378	201479_at	-0.742937037	1.02E-06	DKC1 /// MIR664B /// SNORA56
3379	214706_at	-0.742825926	8.13E-08	ZNF200
3380	210018_x_at	-0.742337037	6.68E-06	MALT1
3381	241371_at	-0.742318519	5.88E-06	TNFRSF10A
3382	230207_s_at	-0.742277778	1.78E-06	DOCK5

3383	223513_at	-0.742196296	1.12E-07	CENPJ
3384	221683_s_at	-0.741418519	2.97E-06	CEP290
3385	204313_s_at	-0.741388889	2.63E-06	CREB1
3386	212200_at	-0.741303704	9.10E-07	ANKLE2
3387	208644_at	-0.740966667	8.00E-09	PARP1
3388	205264_at	-0.740914815	6.19E-06	CD3EAP
3389	202903_at	-0.740388889	4.90E-06	LSM5
3390	210563_x_at	-0.740188889	6.92E-07	CFLAR
3391	217816_s_at	-0.73967037	2.15E-09	PCNP
3392	218747_s_at	-0.739414815	4.03E-06	TAPBPL
3393	210621_s_at	-0.739203704	1.31E-06	RASA1
3394	202324_s_at	-0.739011111	1.92E-10	ACBD3
3395	208930_s_at	-0.738718519	3.11E-07	ILF3
3396	217854_s_at	-0.738651852	8.29E-08	POLR2E
3397	207988_s_at	-0.738540741	2.68E-08	ARPC2
3398	208895_s_at	-0.738411111	2.34E-11	DDX18
3399	204257_at	-0.738196296	9.00E-06	FADS3
3400	209272_at	-0.738066667	3.49E-09	NAB1
3401	224570_s_at	-0.737848148	4.02E-11	IRF2BP2
3402	213062_at	-0.737818519	9.44E-11	NTAN1
3403	213361_at	-0.7378	6.29E-08	TDRD7
3404	213097_s_at	-0.737637037	1.07E-06	DNAJC2
3405	233842_x_at	-0.73672963	2.96E-11	RTFDC1
3406	217140_s_at	-0.7367	7.81E-08	VDAC1
3407	217915_s_at	-0.736411111	7.20E-07	RSL24D1
3408	202246_s_at	-0.736355556	7.90E-06	CDK4
3409	213088_s_at	-0.736296296	5.88E-06	DNAJC9
3410	212897_at	-0.735837037	8.38E-06	CDK19
3411	216505_x_at	-0.735811111	6.71E-11	RPS10L
3412	222465_at	-0.7357	1.03E-08	RSL24D1
3413	204054_at	-0.735537037	3.81E-07	PTEN
3414	206205_at	-0.734811111	6.10E-06	MPHOSPH9
3415	208923_at	-0.734755556	2.36E-12	CYFIP1
3416	227288_at	-0.734248148	1.16E-08	SREK1IP1
3417	201606_s_at	-0.733833333	6.34E-07	PWP1
3418	207721_x_at	-0.733625926	5.23E-09	HINT1
3419	205072_s_at	-0.733577778	2.84E-06	XRCC4
3420	201762_s_at	-0.733233333	1.54E-06	MIR7703 /// PSME2
3421	201622_at	-0.733188889	1.27E-07	SND1
3422	201514_s_at	-0.732922222	2.52E-07	G3BP1
3423	201724_s_at	-0.732844444	6.50E-08	GALNT1
3424	201267_s_at	-0.732833333	8.33E-08	PSMC3
3425	208713_at	-0.732822222	4.06E-08	HNRNPUL1

3426	224677_x_at	-0.73277037	2.62E-08	C11orf31
3427	218448_at	-0.732607407	1.54E-08	GID8
3428	201398_s_at	-0.732574074	3.14E-08	TRAM1
3429	212546_s_at	-0.732518519	3.95E-10	FRYL
3430	223076_s_at	-0.732455556	4.53E-07	NSUN2
3431	244177_at	-0.731911111	1.13E-06	ING1
3432	226839_at	-0.731503704	2.53E-07	NR2C2AP
3433	204173_at	-0.731481481	4.68E-07	MYL6B
3434	219485_s_at	-0.731022222	2.86E-08	PSMD10
3435	203315_at	-0.730514815	1.81E-07	NCK2
3436	215136_s_at	-0.730366667	4.90E-09	EXOSC8
3437	209449_at	-0.729444444	8.77E-10	LSM2
3438	236192_at	-0.72937037	1.77E-08	HOOK3
3439	218932_at	-0.728637037	1.15E-08	ZNHIT6
3440	218388_at	-0.728544444	1.66E-07	PGLS
3441	201306_s_at	-0.728311111	1.81E-09	ANP32B
3442	223084_s_at	-0.727385185	3.44E-09	CCNDBP1
3443	218096_at	-0.727285185	1.10E-06	AGPAT5
3444	211997_x_at	-0.727022222	8.29E-10	H3F3A /// H3F3B /// MIR4738
3445	226901_at	-0.726933333	4.87E-08	C17orf58
3446	205126_at	-0.726837037	4.37E-08	VRK2
3447	209219_at	-0.72627037	2.55E-08	MIR1236 /// NELFE
3448	218159_at	-0.725966667	1.77E-08	DDRGK1
3449	223416_at	-0.725703704	2.34E-10	SF3B6
3450	200828_s_at	-0.725566667	1.38E-07	ZNF207
3451	217836_s_at	-0.725044444	1.65E-10	YY1AP1
3452	225644_at	-0.724996296	7.30E-08	CCDC117
3453	205756_s_at	-0.724959259	4.21E-06	F8
3454	222613_at	-0.724937037	1.66E-07	C12orf4
3455	201841_s_at	-0.724825926	2.30E-08	HSPB1
3456	218109_s_at	-0.724744444	5.47E-07	MFSD1
3457	1567458_s_at	-0.724537037	5.71E-08	RAC1
3458	209330_s_at	-0.723914815	9.44E-09	HNRNPD
3459	200675_at	-0.723707407	7.84E-09	CD81
3460	223532_at	-0.723388889	2.59E-07	ANKRD39
3461	224629_at	-0.72312963	1.45E-10	LMAN1
3462	219122_s_at	-0.7229	7.98E-06	THG1L
3463	218459_at	-0.722514815	8.87E-09	TOR3A
3464	231713_s_at	-0.722451852	2.05E-07	ELP2
3465	218514_at	-0.722207407	2.82E-11	SMG8
3466	59999_at	-0.722133333	6.67E-07	HIF1AN
3467	225702_at	-0.721274074	1.71E-06	C8orf76
3468	218622_at	-0.721085185	2.00E-06	NUP37

3469	227998_at	-0.720840741	5.69E-06	S100A16
3470	224574_at	-0.719985185	5.56E-07	C17orf49 /// RNASEK-C17orf49
3471	218213_s_at	-0.719781481	5.59E-11	TMEM258
3472	208852_s_at	-0.719666667	3.39E-06	CANX
3473	212723_at	-0.719507407	5.24E-06	JMJD6
3474	210276_s_at	-0.719359259	9.31E-07	NOL12 /// TRIOBP
3475	228812_at	-0.719292593	7.86E-08	REL
3476	222488_s_at	-0.719244444	4.14E-10	DCTN4
3477	228299_at	-0.719037037	7.41E-10	KCTD20
3478	226175_at	-0.718703704	7.94E-08	TTC9C
3479	219618_at	-0.718477778	9.55E-06	IRAK4
3480	200071_at	-0.718059259	5.58E-08	SMNDC1
3481	212469_at	-0.717551852	8.21E-07	NIPBL
3482	223270_at	-0.717533333	2.48E-08	CTDSPL2
3483	201233_at	-0.717225926	2.52E-07	PSMD13
3484	201922_at	-0.717222222	4.96E-07	NSA2
3485	200024_at	-0.717103704	8.62E-11	RPS5
3486	226640_at	-0.716681481	1.57E-07	DAGLB
3487	228149_at	-0.716007407	7.27E-07	C7orf60
3488	225669_at	-0.715814815	4.02E-08	IFNAR1
3489	203089_s_at	-0.715740741	5.88E-09	HTRA2
3490	221096_s_at	-0.715007407	3.21E-06	TMCO6
3491	200936_at	-0.714996296	2.12E-11	RPL8
3492	214649_s_at	-0.714907407	2.82E-06	MTMR2
3493	213812_s_at	-0.714622222	1.79E-06	CAMKK2
3494	203719_at	-0.71457037	3.71E-08	ERCC1
3495	200968_s_at	-0.71442963	6.45E-07	PPIB
3496	224740_at	-0.713833333	4.21E-10	SMIM15
3497	213507_s_at	-0.713781481	6.12E-07	KPNB1
3498	214543_x_at	-0.713603704	3.78E-06	QKI
3499	220980_s_at	-0.712185185	8.28E-09	ADPGK
3500	239346_at	-0.71187037	1.95E-06	GTF2H3
3501	204346_s_at	-0.711703704	3.54E-06	RASSF1
3502	233589_x_at	-0.711574074	6.18E-06	TOR4A
3503	226128_at	-0.711140741	3.15E-09	BROX
3504	202673_at	-0.711122222	6.32E-09	DPM1
3505	227388_at	-0.711107407	3.59E-06	TUSC1
3506	212026_s_at	-0.711048148	4.11E-08	EXOC7
3507	201832_s_at	-0.710722222	1.43E-10	USO1
3508	208736_at	-0.710722222	1.23E-09	ARPC3
3509	205241_at	-0.710122222	2.90E-06	SCO2
3510	226496_at	-0.709848148	3.37E-08	ZCCHC7
3511	211711_s_at	-0.709640741	5.73E-07	PTEN

3512	208328_s_at	-0.708977778	1.59E-06	MEF2A
3513	223306_at	-0.70827037	4.07E-08	EBPL
3514	202466_at	-0.707448148	9.25E-07	PAPD7
3515	204334_at	-0.707292593	5.45E-06	KLF7
3516	211945_s_at	-0.706148148	5.59E-09	ITGB1
3517	218395_at	-0.70562963	1.33E-07	ACTR6
3518	235648_at	-0.705544444	3.82E-07	ZNF567
3519	203944_x_at	-0.705525926	9.16E-09	BTN2A1
3520	201112_s_at	-0.705507407	4.05E-06	CSE1L
3521	203630_s_at	-0.705225926	1.67E-07	COG5
3522	218732_at	-0.705133333	1.85E-06	PTRH2
3523	209417_s_at	-0.705085185	2.61E-06	IFI35
3524	208743_s_at	-0.704951852	3.87E-08	YWHAB
3525	224913_s_at	-0.704911111	9.49E-07	TIMM50
3526	214906_x_at	-0.704648148	4.89E-07	N4BP2L1
3527	219251_s_at	-0.704596296	4.25E-06	WDR60
3528	203366_at	-0.703740741	2.85E-06	POLG
3529	212653_s_at	-0.703507407	8.09E-07	EHBP1
3530	218478_s_at	-0.703396296	7.30E-09	ZCCHC8
3531	226748_at	-0.702922222	2.48E-06	LYSMD2
3532	1554260_a_at	-0.702914815	2.68E-06	FRYL
3533	223411_at	-0.702777778	5.02E-09	MIF4GD
3534	218535_s_at	-0.7026	1.14E-09	RIOK2
3535	218370_s_at	-0.702388889	1.15E-08	S100PBP
3536	239042_at	-0.702003704	7.00E-07	TSR1
3537	224966_s_at	-0.701459259	6.60E-06	DUS3L
3538	204243_at	-0.7011	1.46E-07	RLF
3539	229101_at	-0.700762963	1.20E-12	IL17RA
3540	203082_at	-0.700692593	1.16E-06	BMS1
3541	208697_s_at	-0.7004	2.01E-08	EIF3E
3542	223046_at	-0.700303704	2.81E-09	EGLN1
3543	219099_at	-0.700255556	8.56E-06	C12orf5
3544	1568954_s_at	-0.700188889	8.47E-06	C16orf72
3545	202412_s_at	-0.700114815	4.36E-08	USP1
3546	212247_at	-0.699792593	1.68E-08	NUP205
3547	212241_at	-0.699377778	1.43E-07	GCOM1 /// MYZAP /// POLR2M
3548	213859_x_at	-0.699225926	3.33E-07	SMARCA5
3549	205089_at	-0.699211111	8.34E-09	ZNF7
3550	207614_s_at	-0.699137037	4.16E-10	CUL1
3551	209476_at	-0.698914815	2.30E-07	TMX1
3552	227767_at	-0.698655556	4.41E-08	CSNK1G3
3553	200072_s_at	-0.698348148	2.10E-10	HNRNPM
3554	221472_at	-0.698177778	1.64E-09	SERINC3

3555	203023_at	-0.698103704	3.61E-06	NOP16
3556	201555_at	-0.697877778	5.52E-07	MCM3
3557	32069_at	-0.697814815	1.60E-07	N4BP1
3558	213811_x_at	-0.69767037	3.47E-07	TCF3
3559	200728_at	-0.697325926	2.65E-09	ACTR2
3560	215091_s_at	-0.697318519	9.05E-09	GTF3A
3561	208726_s_at	-0.696977778	5.21E-08	EIF2S2
3562	55093_at	-0.696511111	1.23E-06	CHPF2
3563	207622_s_at	-0.696377778	1.94E-06	ABCF2
3564	223320_s_at	-0.696333333	8.59E-07	ABCB10
3565	200750_s_at	-0.695496296	4.00E-06	RAN
3566	203219_s_at	-0.695262963	5.12E-07	APRT
3567	208801_at	-0.695140741	3.40E-11	SRP72
3568	209974_s_at	-0.695011111	2.66E-07	BUB3
3569	203654_s_at	-0.694811111	1.14E-09	COIL
3570	222163_s_at	-0.694303704	7.55E-08	SPATA5L1
3571	225080_at	-0.694218519	1.43E-06	MYO1C
3572	224068_x_at	-0.694203704	3.23E-11	RBM22
3573	200075_s_at	-0.693996296	9.85E-08	GUK1
3574	208638_at	-0.693814815	1.47E-09	PDIA6
3575	200971_s_at	-0.693696296	4.00E-08	LOC101928061 /// SERP1
3576	225592_at	-0.693648148	9.40E-08	NRM
3577	201887_at	-0.693644444	1.23E-07	IL13RA1
3578	1553348_a_at	-0.6935	6.36E-06	NFX1
3579	212264_s_at	-0.693296296	5.72E-09	WAPAL
3580	228114_x_at	-0.693018519	8.47E-06	C16orf13
3581	244462_at	-0.692818519	3.94E-06	ZNF224
3582	219079_at	-0.692366667	8.50E-07	CYB5R4
3583	202979_s_at	-0.692240741	3.10E-08	CREBZF
3584	200826_at	-0.692177778	4.17E-09	SNRPD2
3585	208616_s_at	-0.692140741	6.88E-09	PTP4A2
3586	216526_x_at	-0.691851852	5.19E-10	HLA-C
3587	228914_at	-0.691607407	7.92E-07	
3588	226015_at	-0.690825926	1.20E-11	ZNF12
3589	1554153_a_at	-0.690592593	5.71E-07	PHF21A
3590	222103_at	-0.690566667	4.04E-07	ATF1
3591	226951_at	-0.69047037	1.79E-06	C2orf49
3592	209027_s_at	-0.690144444	3.23E-06	ABI1
3593	200842_s_at	-0.69012963	8.21E-06	EPRS
3594	225694_at	-0.689644444	5.23E-09	CDK12
3595	226633_at	-0.689403704	1.78E-07	RAB8B
3596	210093_s_at	-0.688803704	6.50E-08	MAGOH /// MAGOHB
3597	203102_s_at	-0.688559259	5.00E-07	MGAT2

3598	202691_at	-0.688425926	1.58E-06	SNRPD1
3599	214280_x_at	-0.688185185	2.58E-08	HNRNPA1
3600	212420_at	-0.688092593	9.16E-08	ELF1
3601	212507_at	-0.687985185	2.52E-09	TMEM131
3602	214501_s_at	-0.68752963	1.98E-07	H2AFY
3603	220990_s_at	-0.6874	2.48E-07	MIR21 /// VMP1
3604	214359_s_at	-0.686766667	2.31E-06	HSP90AB1
3605	201795_at	-0.686751852	8.27E-06	LBR
3606	203701_s_at	-0.686655556	1.75E-06	TRMT1
3607	227157_at	-0.68542963	7.06E-09	PRIMPOL
3608	201342_at	-0.685333333	4.02E-06	SNRPC
3609	204835_at	-0.685211111	6.17E-08	POLA1
3610	223174_at	-0.685055556	3.61E-08	BTBD10
3611	208975_s_at	-0.684903704	1.02E-07	KPNB1
3612	235170_at	-0.684811111	2.98E-06	ZNF92
3613	209549_s_at	-0.684418519	8.62E-11	DGUOK
3614	223066_at	-0.684248148	6.68E-10	SNAPIN
3615	211250_s_at	-0.684207407	4.33E-06	SH3BP2
3616	204215_at	-0.684055556	6.01E-07	TMEM243
3617	218768_at	-0.683485185	4.95E-07	NUP107
3618	205904_at	-0.682914815	7.10E-06	MICA
3619	210596_at	-0.682474074	1.24E-07	MAGT1
3620	222889_at	-0.682177778	6.77E-07	DCLRE1B
3621	201446_s_at	-0.681903704	2.66E-07	TIA1
3622	200036_s_at	-0.681222222	1.75E-09	RPL10A
3623	219212_at	-0.681007407	6.82E-08	HSPA14
3624	202306_at	-0.680503704	4.17E-09	POLR2G
3625	201157_s_at	-0.680492593	5.35E-07	NMT1
3626	201180_s_at	-0.679977778	2.33E-10	GNAI3
3627	221732_at	-0.679907407	5.88E-07	CANT1
3628	225124_at	-0.679774074	6.18E-09	PPP1R9B
3629	202670_at	-0.6797	7.10E-06	MAP2K1
3630	203466_at	-0.679262963	6.31E-08	MPV17
3631	202258_s_at	-0.6791	1.46E-07	N4BP2L2
3632	223476_s_at	-0.679014815	6.87E-07	C12orf65
3633	213803_at	-0.678685185	5.21E-06	KPNB1
3634	221734_at	-0.678574074	1.88E-06	PRRC1
3635	235812_at	-0.677696296	4.37E-10	CNEP1R1
3636	235767_x_at	-0.67752963	3.62E-08	PHAX
3637	203651_at	-0.677396296	1.04E-06	ZFYVE16
3638	218927_s_at	-0.676814815	4.61E-09	CHST12
3639	235177_at	-0.676751852	7.59E-06	METTL21A
3640	212496_s_at	-0.676640741	2.37E-06	KDM4B

3641	216380_x_at	-0.67597037	2.88E-07	GS1-111G14.1
3642	203218_at	-0.675918519	1.05E-08	MAPK9
3643	208809_s_at	-0.675837037	2.64E-08	C6orf62
3644	223647_x_at	-0.675533333	1.25E-07	HSCB
3645	213526_s_at	-0.6753	5.68E-09	LIN37
3646	223089_at	-0.675122222	1.47E-06	VEZT
3647	216274_s_at	-0.67497037	6.34E-09	SEC11A
3648	222387_s_at	-0.674944444	3.26E-06	VPS35
3649	234954_at	-0.674281481	5.70E-06	GAPDHP73 /// GAPDHP73
3650	210759_s_at	-0.674218519	7.67E-09	PSMA1
3651	202978_s_at	-0.674211111	9.31E-07	CREBZF
3652	201973_s_at	-0.6734	8.77E-10	CCZ1 /// CCZ1B
3653	218781_at	-0.673281481	1.69E-06	SMC6
3654	212770_at	-0.673225926	2.61E-07	TLE3
3655	221007_s_at	-0.672937037	1.75E-08	FIP1L1
3656	202443_x_at	-0.671714815	7.72E-09	NOTCH2
3657	212108_at	-0.671107407	1.58E-08	FAF2
3658	218108_at	-0.670755556	4.41E-08	UBR7
3659	218703_at	-0.670151852	8.37E-08	SEC22A
3660	222633_at	-0.670092593	9.42E-08	TBL1XR1
3661	225538_at	-0.670033333	1.04E-06	ZCCHC9
3662	223738_s_at	-0.669696296	3.29E-06	PGM2
3663	202863_at	-0.669677778	1.53E-07	SP100
3664	224572_s_at	-0.669077778	2.39E-07	IRF2BP2
3665	202092_s_at	-0.668677778	1.07E-08	ARL2BP
3666	224615_x_at	-0.668566667	1.54E-06	HM13
3667	226449_at	-0.668559259	3.55E-09	CEP120
3668	217608_at	-0.668522222	7.60E-07	SREK1IP1
3669	207831_x_at	-0.668418519	1.41E-07	DHPS
3670	AFFX-	-0.668285185	2.69E-07	GAPDH
3671	229194_at	-0.667974074	3.75E-07	PCGF5
3672	209482_at	-0.66782963	1.06E-08	POP7
3673	228992_at	-0.667774074	1.28E-08	MED28
3674	200088_x_at	-0.667492593	2.70E-10	NBEAL1 /// RPL12
3675	219348_at	-0.665281481	8.81E-07	USE1
3676	223493_at	-0.66432963	1.08E-06	FBXO4
3677	202393_s_at	-0.664074074	1.63E-06	KLF10
3678	1552617_a_at	-0.663233333	2.89E-07	RFWD2
3679	222001_x_at	-0.663207407	6.42E-06	LINC00623 /// LINC00869
3680	200634_at	-0.663033333	7.12E-07	PFN1
3681	200958_s_at	-0.662988889	4.30E-07	SDCBP
3682	228991_at	-0.662937037	1.48E-07	CDK13
3683	1553709_a_at	-0.662107407	1.42E-08	PRPF38A

3684	209682_at	-0.661107407	1.42E-06	CBLB
3685	209565_at	-0.661059259	2.68E-07	RNF113A
3686	212251_at	-0.658662963	6.12E-08	MTDH
3687	202355_s_at	-0.658533333	9.43E-09	GTF2F1
3688	222512_at	-0.658307407	8.42E-10	NUB1
3689	201258_at	-0.658144444	8.54E-10	RPS16
3690	200791_s_at	-0.657911111	3.63E-10	IQGAP1
3691	207168_s_at	-0.657892593	2.52E-08	H2AFY
3692	201763_s_at	-0.657644444	1.43E-09	DAXX
3693	208812_x_at	-0.657537037	1.55E-09	HLA-C
3694	218040_at	-0.657492593	1.28E-06	PRPF38B
3695	214684_at	-0.657188889	3.95E-07	MEF2A
3696	205192_at	-0.657055556	6.56E-06	MAP3K14
3697	200613_at	-0.656614815	2.08E-06	AP2M1
3698	200087_s_at	-0.656211111	1.16E-09	TMED2
3699	209786_at	-0.655959259	1.52E-07	HMGN4
3700	1558943_x_at	-0.65522963	5.18E-07	ZNF765
3701	202621_at	-0.655114815	9.05E-10	IRF3
3702	226720_at	-0.654762963	9.16E-08	PWWP2A
3703	228386_s_at	-0.654044444	1.51E-07	DDX59
3704	209716_at	-0.653874074	8.25E-06	CSF1
3705	212576_at	-0.653707407	7.46E-06	MGRN1
3706	219492_at	-0.653677778	9.24E-07	CHIC2
3707	225470_at	-0.653488889	2.74E-09	NUP35
3708	203107_x_at	-0.65332963	2.97E-10	RPS2 /// SNORA64
3709	200640_at	-0.653251852	1.77E-07	YWHAZ
3710	217750_s_at	-0.652762963	1.64E-07	UBE2Z
3711	208739_x_at	-0.652511111	2.90E-08	LOC101929087 /// SUMO2 /// SUMO3
3712	218495_at	-0.65247037	1.36E-07	UXT
3713	222464_s_at	-0.652181481	5.46E-08	MCMBP
3714	211061_s_at	-0.651881481	4.15E-06	MGAT2
3715	212745_s_at	-0.651825926	6.49E-06	BBS4
3716	228365_at	-0.651796296	2.97E-07	CPNE8
3717	209939_x_at	-0.651618519	4.65E-06	CFLAR
3718	212958_x_at	-0.651525926	4.90E-06	PAM
3719	233746_x_at	-0.651518519	8.19E-07	HYPK /// MIR1282 /// SERF2 /// SERF2-C15ORF63
3720	211935_at	-0.65142963	1.12E-06	ARL6IP1
3721	213320_at	-0.651407407	6.44E-06	PRMT3
3722	201864_at	-0.651311111	6.88E-06	GDI1
3723	208865_at	-0.65107037	1.76E-10	CSNK1A1
3724	219297_at	-0.651018519	4.20E-06	WDR44
3725	200693_at	-0.650988889	2.64E-08	YWHAQ

3726	200677_at	-0.650844444	1.57E-06	PTTG1IP
3727	208811_s_at	-0.650751852	4.83E-06	DNAJB6 /// TMEM135
3728	205707_at	-0.649940741	6.22E-09	IL17RA
3729	201338_x_at	-0.649733333	9.41E-09	GTF3A
3730	200991_s_at	-0.64967037	2.30E-07	SNX17
3731	208805_at	-0.649462963	1.78E-07	KIAA0391 /// PSMA6
3732	233632_s_at	-0.649411111	2.15E-07	XRN1
3733	201453_x_at	-0.649085185	1.08E-06	RHEB
3734	212540_at	-0.64902963	1.78E-07	CDC34
3735	235260_s_at	-0.648877778	5.76E-08	PACRGL
3736	224890_s_at	-0.648811111	2.84E-08	LAMTOR4
3737	201152_s_at	-0.648622222	1.21E-09	MBNL1
3738	225087_at	-0.6484	4.38E-09	FOPNL
3739	200023_s_at	-0.647966667	1.14E-08	EIF3F
3740	219035_s_at	-0.64782963	8.27E-10	RNF34
3741	201516_at	-0.647688889	6.12E-06	SRM
3742	202397_at	-0.647685185	3.69E-07	NUTF2 /// NUTF2P4
3743	226395_at	-0.647162963	4.07E-07	HOOK3
3744	224788_at	-0.64637037	1.91E-06	ARF6
3745	218073_s_at	-0.645803704	2.61E-06	NDC1
3746	210570_x_at	-0.645792593	2.71E-08	MAPK9
3747	203345_s_at	-0.645614815	1.43E-08	MTF2
3748	202907_s_at	-0.644814815	3.18E-07	NBN
3749	1553581_s_at	-0.6448	6.51E-08	SREK1IP1
3750	203396_at	-0.644714815	2.39E-07	PSMA4
3751	208641_s_at	-0.644674074	1.13E-08	RAC1
3752	200822_x_at	-0.644496296	2.13E-06	TPI1
3753	218257_s_at	-0.644177778	6.56E-06	UGGT1
3754	221746_at	-0.643985185	2.15E-07	UBL4A
3755	211084_x_at	-0.643840741	1.09E-06	PRKD3
3756	218767_at	-0.643755556	1.21E-08	REXO4
3757	211612_s_at	-0.643711111	7.36E-06	IL13RA1
3758	219421_at	-0.643444444	5.85E-06	TTC33
3759	202665_s_at	-0.643066667	6.36E-07	WIPF1
3760	221873_at	-0.642874074	4.99E-09	ZNF143
3761	202467_s_at	-0.642766667	3.86E-09	COPS2
3762	203109_at	-0.642688889	5.10E-07	UBE2M
3763	217789_at	-0.641892593	1.63E-07	SNX6
3764	225761_at	-0.641707407	2.21E-08	PAPD4
3765	213000_at	-0.640833333	6.92E-09	MORC3
3766	212320_at	-0.640511111	4.23E-06	TUBB
3767	229129_at	-0.640359259	1.89E-06	HNRNPD
3768	202088_at	-0.640218519	7.56E-06	SLC39A6

3769	209282_at	-0.640140741	1.98E-09	PRKD2
3770	1555751_a_at	-0.640062963	9.70E-09	GEMIN7
3771	213982_s_at	-0.6395	3.43E-06	RABGAP1L
3772	204732_s_at	-0.639385185	4.32E-06	TRIM23
3773	230618_s_at	-0.63922963	1.70E-07	
3774	235536_at	-0.639185185	9.39E-08	SNORD89
3775	230734_x_at	-0.638251852	3.81E-06	STRN
3776	216559_x_at	-0.638155556	1.25E-07	HNRNPA1P3 /// HNRNPA1P3
3777	217931_at	-0.638048148	2.74E-08	CNPY3
3778	219394_at	-0.637514815	7.64E-09	PGS1
3779	208866_at	-0.637107407	2.53E-07	CSNK1A1
3780	209932_s_at	-0.63682963	1.76E-06	DUT
3781	208802_at	-0.636277778	5.08E-09	SRP72
3782	205068_s_at	-0.636233333	2.48E-06	ARHGAP26
3783	200002_at	-0.636133333	1.04E-10	RPL35
3784	235542_at	-0.635718519	1.48E-07	TET3
3785	200082_s_at	-0.63562963	3.94E-10	RPS7
3786	209103_s_at	-0.634966667	2.13E-09	UFD1L
3787	221050_s_at	-0.634903704	9.03E-06	GTPBP2
3788	215313_x_at	-0.634603704	1.21E-08	HLA-A
3789	242760_x_at	-0.634355556	9.24E-07	PIGB
3790	202484_s_at	-0.634181481	5.99E-08	MBD2
3791	219861_at	-0.633666667	1.95E-06	DNAJC17
3792	222389_s_at	-0.633625926	5.46E-08	WAC
3793	215157_x_at	-0.632114815	6.54E-07	PABPC1
3794	224587_at	-0.631925926	3.84E-08	SUB1
3795	225332_at	-0.631837037	5.62E-07	OIP5-AS1
3796	214499_s_at	-0.631722222	7.74E-08	BCLAF1
3797	217831_s_at	-0.631574074	5.49E-06	NSFL1C
3798	226881_at	-0.629281481	8.64E-07	GRPEL2
3799	216602_s_at	-0.629085185	1.57E-06	FARSA
3800	214006_s_at	-0.629040741	9.42E-06	GGCX
3801	217737_x_at	-0.628492593	3.39E-10	RTFDC1
3802	222411_s_at	-0.628374074	2.44E-06	SSR3
3803	217938_s_at	-0.628096296	2.41E-07	KCMF1
3804	217778_at	-0.627359259	4.69E-07	SLC39A1
3805	205748_s_at	-0.627185185	7.62E-06	RNF126
3806	200064_at	-0.62632963	3.31E-07	HSP90AB1
3807	218244_at	-0.625944444	1.23E-06	NOL8
3808	224334_s_at	-0.625825926	1.69E-07	MRPL51 /// SPTLC1
3809	222398_s_at	-0.624996296	5.27E-07	EFTUD2
3810	224972_at	-0.624707407	7.86E-08	ROMO1
3811	57516_at	-0.624677778	8.47E-07	ZNF764

3812	218006_s_at	-0.624511111	1.86E-07	ZNF22
3813	201817_at	-0.624348148	2.57E-06	UBE3C
3814	223397_s_at	-0.623881481	2.27E-06	NIP7
3815	226628_at	-0.622996296	3.81E-07	THOC2
3816	221699_s_at	-0.622777778	2.21E-06	DDX50
3817	225317_at	-0.622518519	6.35E-08	ACBD6
3818	224866_at	-0.622303704	8.13E-07	FAR1
3819	208759_at	-0.622274074	1.57E-06	NCSTN
3820	213616_at	-0.621844444	1.77E-06	TPGS2
3821	231843_at	-0.621803704	1.83E-06	DDX55
3822	213762_x_at	-0.621533333	8.27E-07	LOC101928747 /// RBMX /// SNORD61
3823	202097_at	-0.621211111	5.63E-07	NUP153
3824	213123_at	-0.621125926	7.50E-10	MFAP3
3825	224800_at	-0.620266667	1.05E-06	WDFY1
3826	1553987_at	-0.620077778	2.21E-07	MAPKAPK5-AS1
3827	212180_at	-0.619977778	5.92E-10	CRKL
3828	208887_at	-0.619251852	1.45E-08	EIF3G
3829	200627_at	-0.619155556	1.02E-09	PTGES3
3830	208822_s_at	-0.618114815	4.40E-07	DAP3
3831	228745_at	-0.617740741	6.17E-07	SGTB
3832	224949_at	-0.617555556	1.00E-06	YIPF5
3833	200624_s_at	-0.61752963	2.05E-06	MATR3 /// SNHG4
3834	218085_at	-0.617525926	4.63E-07	CHMP5
3835	223145_s_at	-0.617251852	3.32E-07	AKIRIN2
3836	216194_s_at	-0.617022222	2.51E-08	TBCB
3837	224874_at	-0.616577778	2.05E-06	POLR1D
3838	225229_at	-0.616518519	1.12E-07	AFF4
3839	226242_at	-0.616133333	1.37E-08	C1orf131
3840	213883_s_at	-0.615511111	2.37E-06	TM2D1
3841	200723_s_at	-0.615388889	7.64E-06	CAPRIN1
3842	226236_at	-0.615244444	5.97E-07	LINC00493
3843	227132_at	-0.615196296	3.64E-07	ZNF706
3844	216304_x_at	-0.615011111	2.06E-07	YME1L1
3845	217770_at	-0.614607407	2.57E-06	PIGT
3846	212712_at	-0.614440741	4.93E-06	CAMSAP1
3847	232432_s_at	-0.614418519	6.97E-06	SLC30A5
3848	215963_x_at	-0.614218519	3.71E-09	RPL3
3849	212426_s_at	-0.613996296	3.79E-08	YWHAQ
3850	215493_x_at	-0.6136	4.56E-08	BTN2A1
3851	203462_x_at	-0.613562963	8.58E-06	EIF3B
3852	206833_s_at	-0.612662963	4.87E-07	ACYP2 /// LOC101927144
3853	218738_s_at	-0.611825926	9.99E-07	RNF138
3854	208679_s_at	-0.611751852	1.59E-06	ARPC2

3855	218117_at	-0.611462963	1.06E-08	RBX1
3856	242463_x_at	-0.611281481	2.60E-06	ZNF600
3857	213513_x_at	-0.610881481	7.27E-08	ARPC2
3858	212891_s_at	-0.610877778	5.36E-06	GADD45GIP1
3859	223980_s_at	-0.610603704	5.30E-07	SP110
3860	200639_s_at	-0.61002963	2.03E-06	YWHAZ
3861	205573_s_at	-0.609751852	6.12E-06	SNX7
3862	202824_s_at	-0.609248148	3.50E-07	TCEB1
3863	205644_s_at	-0.609085185	2.05E-07	SNRPG
3864	211921_x_at	-0.608488889	1.15E-07	LOC100506248 /// LOC728026 /// MIR1244-1 /// MIR1244-2 /// MIR1244-3 /// PTMA
3865	208858_s_at	-0.608437037	3.02E-06	ESYT1
3866	212530_at	-0.607759259	1.85E-07	NEK7
3867	218053_at	-0.607551852	3.64E-10	PRPF40A
3868	200957_s_at	-0.607292593	8.02E-07	SSRP1
3869	226082_s_at	-0.6071	4.24E-08	SCAF4
3870	200837_at	-0.607	1.69E-06	BCAP31
3871	200866_s_at	-0.606440741	6.27E-06	PSAP
3872	224968_at	-0.606381481	4.09E-06	CCDC104
3873	229175_at	-0.606355556	6.64E-06	SMYD4
3874	207198_s_at	-0.606203704	6.43E-07	LIMS1 /// LIMS3L
3875	214527_s_at	-0.605944444	6.36E-09	PQBP1
3876	223526_at	-0.605740741	8.62E-07	C18orf21
3877	212246_at	-0.605548148	1.16E-07	MCFD2
3878	226515_at	-0.605059259	2.35E-06	CCDC127
3879	213483_at	-0.604544444	8.27E-06	PPWD1
3880	212077_at	-0.604385185	2.07E-07	CALD1
3881	222610_s_at	-0.604140741	1.60E-08	S100PBP
3882	222630_at	-0.60397037	7.57E-06	RFX7
3883	204645_at	-0.603948148	3.09E-06	CCNT2
3884	207153_s_at	-0.603188889	1.03E-07	GLMN
3885	200715_x_at	-0.603044444	1.32E-06	RPL13A /// SNORD32A /// SNORD33 /// SNORD34 /// SNORD35A
3886	212447_at	-0.602981481	2.14E-08	KBTBD2
3887	223297_at	-0.602581481	2.37E-08	AMMECR1L
3888	227413_at	-0.602514815	6.83E-07	UBLCP1
3889	211972_x_at	-0.60162963	2.26E-08	RPLP0
3890	212519_at	-0.601340741	1.19E-07	UBE2E1
3891	223328_at	-0.601188889	5.89E-06	ARMC10
3892	204299_at	-0.601148148	4.92E-06	SRSF10
3893	39891_at	-0.600937037	1.73E-09	ZNF710
3894	202105_at	-0.600722222	6.52E-06	IGBP1
3895	200740_s_at	-0.600325926	2.72E-08	SUMO3

3896	218095_s_at	-0.599911111	1.71E-06	TMEM165
3897	66053_at	-0.599633333	3.22E-06	HNRNPUL2 /// HNRNPUL2-BSCL2
3898	213103_at	-0.598733333	5.09E-06	STARD13
3899	211318_s_at	-0.598614815	2.55E-06	RAE1
3900	218606_at	-0.598322222	9.03E-06	ZDHHC7
3901	200754_x_at	-0.597840741	7.26E-08	MIR636 /// SRSF2
3902	202868_s_at	-0.596911111	8.72E-08	POP4
3903	213124_at	-0.596881481	1.18E-06	ZNF473
3904	207785_s_at	-0.59687037	2.27E-08	RBPJ
3905	202212_at	-0.596474074	3.92E-07	PES1
3906	AFFX-	-0.5955	2.41E-08	ACTB
3907	212330_at	-0.595337037	6.35E-06	TFDP1
3908	213011_s_at	-0.595325926	9.82E-06	TPI1
3909	206052_s_at	-0.594825926	7.97E-07	SLBP
3910	209165_at	-0.594351852	9.32E-07	AATF
3911	203595_s_at	-0.594066667	7.66E-06	IFIT5
3912	201218_at	-0.59397037	1.36E-06	CTBP2
3913	222497_x_at	-0.593962963	3.97E-08	NMD3
3914	220933_s_at	-0.593796296	5.42E-08	ZCCHC6
3915	214800_x_at	-0.593788889	5.09E-08	BTF3
3916	203629_s_at	-0.593522222	3.65E-07	COG5
3917	227930_at	-0.593122222	8.18E-06	AGO4
3918	212072_s_at	-0.592707407	7.22E-07	CSNK2A1
3919	207585_s_at	-0.592644444	8.30E-07	RPL36AL
3920	218572_at	-0.5922	5.51E-06	CHMP4A /// TM9SF1
3921	218968_s_at	-0.592074074	1.55E-07	ZFP64
3922	226265_at	-0.591318519	4.48E-06	QSER1
3923	216996_s_at	-0.590803704	9.49E-06	FASTKD2
3924	230588_s_at	-0.590774074	9.75E-06	LOC285074 /// LOC730268
3925	208931_s_at	-0.590677778	1.18E-06	ILF3
3926	1557915_s_at	-0.5903	2.85E-06	GSTO1
3927	204460_s_at	-0.590074074	2.44E-07	RAD1
3928	208936_x_at	-0.590022222	6.91E-07	LGALS8
3929	228334_x_at	-0.589692593	3.53E-08	CEP44
3930	202579_x_at	-0.589685185	1.34E-06	HMGN4
3931	210053_at	-0.589481481	2.88E-06	TAF5
3932	213654_at	-0.589381481	1.74E-06	TAF5L
3933	222494_at	-0.588722222	2.39E-06	FOXN3
3934	219940_s_at	-0.588	7.93E-08	PCID2
3935	227836_at	-0.587725926	5.07E-06	UTP23
3936	200625_s_at	-0.587611111	7.49E-09	CAP1
3937	206860_s_at	-0.587503704	3.17E-06	MIOS
3938	228095_at	-0.587403704	2.17E-06	PHF14

3939	200709_at	-0.587333333	1.11E-08	FKBP1A /// LOC101929368
3940	209181_s_at	-0.587177778	5.70E-07	RABGGTB /// SNORD45A /// SNORD45B /// SNORD45C
3941	224671_at	-0.587066667	2.04E-06	MRPL10
3942	225082_at	-0.586114815	2.89E-07	CPSF3
3943	226100_at	-0.585492593	1.01E-08	KMT2E
3944	218571_s_at	-0.585448148	1.29E-06	CHMP4A /// TM9SF1
3945	223414_s_at	-0.585381481	1.29E-07	LYAR
3946	200809_x_at	-0.584407407	6.29E-09	RPL12
3947	231896_s_at	-0.584185185	3.31E-07	DENR
3948	201458_s_at	-0.583688889	6.41E-06	BUB3
3949	222821_s_at	-0.58362963	7.95E-08	GEMIN7
3950	216241_s_at	-0.583518519	5.69E-06	TCEA1
3951	212106_at	-0.583359259	2.21E-06	FAF2
3952	202034_x_at	-0.582644444	3.49E-06	RB1CC1
3953	202012_s_at	-0.582544444	1.12E-07	EXT2
3954	224602_at	-0.582248148	7.30E-08	C4orf3
3955	225253_s_at	-0.581622222	3.98E-07	METTL2A /// METTL2B
3956	225231_at	-0.581274074	5.71E-07	CBL
3957	202140_s_at	-0.580977778	7.06E-07	CLK3
3958	202352_s_at	-0.580944444	2.36E-07	PSMD12
3959	53720_at	-0.58077037	1.82E-06	C19orf66
3960	211939_x_at	-0.58062963	5.53E-07	BTF3
3961	209104_s_at	-0.580611111	4.04E-06	NHP2
3962	221803_s_at	-0.580074074	3.41E-06	NRBF2
3963	200651_at	-0.580040741	7.40E-09	GNB2L1 /// SNORD95 /// SNORD96A
3964	208856_x_at	-0.580040741	5.12E-09	RPLP0
3965	209814_at	-0.57992963	6.50E-07	ZNF330
3966	222401_s_at	-0.579040741	5.85E-07	TMEM50A
3967	225384_at	-0.5788	2.74E-06	DOCK7
3968	202717_s_at	-0.578614815	1.52E-06	CDC16
3969	201351_s_at	-0.578337037	6.68E-07	YME1L1
3970	229618_at	-0.5774	3.40E-06	SNX16
3971	202846_s_at	-0.576403704	3.40E-07	PIGC
3972	1552628_a_at	-0.5763	1.99E-08	HERPUD2
3973	212457_at	-0.576292593	5.45E-06	TFE3
3974	218438_s_at	-0.575025926	1.83E-07	MED28
3975	222428_s_at	-0.574774074	7.14E-06	LARS
3976	202214_s_at	-0.574266667	5.15E-08	CUL4B
3977	225398_at	-0.574088889	4.73E-06	RPUSD4
3978	214173_x_at	-0.573714815	8.88E-08	URI1
3979	219283_at	-0.573081481	8.09E-06	C1GALT1C1
3980	218286_s_at	-0.573074074	1.19E-06	RNF7

3981	202734_at	-0.572485185	6.17E-07	TRIP10
3982	229231_at	-0.572362963	2.19E-07	LOC101929841 /// LRRC37B
3983	91952_at	-0.572122222	1.20E-06	DCAF15
3984	202480_s_at	-0.571237037	2.73E-06	DEDD
3985	217403_s_at	-0.571062963	3.85E-06	ZNF227
3986	224885_s_at	-0.571048148	3.07E-06	KRTCAP2
3987	208674_x_at	-0.57082963	6.89E-06	DDOST
3988	202189_x_at	-0.569781481	4.79E-07	MIR4745 /// PTBP1
3989	200029_at	-0.569592593	2.67E-08	RPL19
3990	208771_s_at	-0.568962963	5.80E-06	LOC101928830 /// LTA4H
3991	212597_s_at	-0.568833333	3.06E-08	HMGXB4
3992	201033_x_at	-0.568566667	1.74E-08	RPLP0
3993	209593_s_at	-0.568085185	3.17E-08	TOR1B
3994	200749_at	-0.56707037	4.22E-07	RAN
3995	224935_at	-0.56672963	3.06E-06	EIF2S3
3996	208517_x_at	-0.566503704	1.84E-06	BTF3
3997	225222_at	-0.566055556	9.18E-08	HIAT1
3998	203947_at	-0.565362963	7.40E-06	CSTF3
3999	212417_at	-0.565303704	5.71E-06	SCAMP1
4000	223013_at	-0.565081481	9.95E-07	TBL1XR1
4001	204461_x_at	-0.564222222	1.30E-06	RAD1
4002	222751_at	-0.564188889	5.19E-07	HERPUD2
4003	226797_at	-0.563996296	5.44E-06	MBTD1
4004	223163_s_at	-0.563659259	3.17E-06	ZC3HC1
4005	209725_at	-0.56312963	5.16E-06	UTP20
4006	201994_at	-0.563074074	3.15E-08	MORF4L2
4007	202174_s_at	-0.562355556	9.01E-06	PCM1
4008	202408_s_at	-0.56207037	6.10E-08	PRPF31
4009	202462_s_at	-0.560955556	5.22E-06	DDX46
4010	208734_x_at	-0.560385185	3.43E-06	RAB2A
4011	217527_s_at	-0.56022963	2.95E-06	NFATC2IP
4012	203436_at	-0.558985185	1.32E-07	RPP30
4013	218076_s_at	-0.558359259	2.10E-06	ARHGAP17
4014	201238_s_at	-0.55817037	3.58E-06	CAPZA2
4015	212829_at	-0.558025926	6.93E-06	PIP4K2A
4016	200781_s_at	-0.557914815	1.46E-09	RPS15A
4017	218282_at	-0.557437037	4.21E-06	EDEM2
4018	224985_at	-0.557411111	1.02E-06	NRAS
4019	213696_s_at	-0.556837037	2.22E-06	MED8
4020	227286_at	-0.556659259	5.38E-06	INO80E
4021	219649_at	-0.556444444	3.17E-07	ALG6
4022	210849_s_at	-0.556125926	2.34E-06	VPS41
4023	226730_s_at	-0.555059259	8.98E-07	USP37

4024	209020_at	-0.554985185	8.80E-07	OSER1
4025	219292_at	-0.554714815	2.44E-06	THAP1
4026	201771_at	-0.554607407	6.34E-07	SCAMP3
4027	201636_at	-0.553937037	2.10E-06	FXR1
4028	201459_at	-0.553725926	4.52E-06	RUVBL2
4029	201648_at	-0.553485185	2.97E-06	JAK1
4030	202407_s_at	-0.553448148	1.07E-06	PRPF31
4031	201270_x_at	-0.553022222	1.48E-06	NUDCD3
4032	225127_at	-0.552766667	3.01E-06	TMEM181
4033	203957_at	-0.552674074	1.89E-07	E2F6
4034	200949_x_at	-0.552648148	2.27E-08	RPS20 /// SNORD54
4035	1558136_s_at	-0.552455556	2.04E-06	TAF11
4036	218972_at	-0.552322222	5.39E-06	TTC17
4037	218646_at	-0.552103704	9.36E-08	C4orf27
4038	212244_at	-0.551696296	3.99E-07	GCOM1 /// POLR2M
4039	226165_at	-0.551622222	1.54E-06	C8orf59
4040	239377_at	-0.55092963	1.06E-06	EIF1AD
4041	224607_s_at	-0.55067037	5.12E-10	SRP68
4042	202406_s_at	-0.550088889	8.78E-06	TIAL1
4043	208756_at	-0.549937037	1.90E-06	EIF3I
4044	243816_at	-0.549503704	8.91E-06	LL22NC03-N27C7.1 /// ZNF70
4045	209669_s_at	-0.54932963	5.29E-07	SERBP1
4046	223857_x_at	-0.549311111	1.77E-07	EMC4
4047	209143_s_at	-0.54882963	4.33E-07	CLNS1A
4048	224930_x_at	-0.547892593	9.65E-07	RPL7A /// SNORD24 /// SNORD36A /// SNORD36B
4049	211575_s_at	-0.547262963	1.67E-06	UBE3A
4050	205664_at	-0.547248148	7.87E-07	KIN
4051	200772_x_at	-0.546807407	2.26E-06	LOC100506248 /// LOC728026 /// MIR1244-1 /// MIR1244-2 /// MIR1244-3 /// PTMA
4052	225756_at	-0.545351852	2.82E-06	CSNK1E /// CSNK1E
4053	200014_s_at	-0.544677778	3.21E-08	HNRNPC
4054	221253_s_at	-0.544151852	7.50E-06	BLOC1S5-TXNDC5 /// TXNDC5
4055	201317_s_at	-0.543859259	1.67E-06	PSMA2
4056	217774_s_at	-0.543751852	2.07E-08	TRMT112
4057	218809_at	-0.54362963	3.77E-08	PANK2
4058	211666_x_at	-0.5434	3.14E-09	RNU86 /// RPL3 /// SNORD83B
4059	202542_s_at	-0.54267037	1.46E-06	AIMP1
4060	226740_x_at	-0.541803704	3.64E-09	NBPF10 /// NBPF14 /// NBPF26
4061	217885_at	-0.541755556	7.67E-06	IPO9
4062	201405_s_at	-0.541607407	1.63E-06	COPS6
4063	212227_x_at	-0.541303704	4.64E-07	EIF1
4064	208617_s_at	-0.539918519	1.72E-06	PTP4A2

4065	202905_x_at	-0.539055556	7.08E-06	NBN
4066	218348_s_at	-0.537625926	2.13E-09	ZC3H7A
4067	218387_s_at	-0.537518519	2.06E-06	PGLS
4068	235253_at	-0.536874074	4.24E-06	RAD1
4069	227205_at	-0.53652963	5.02E-07	TAF1
4070	208503_s_at	-0.5364	1.28E-07	GATAD1
4071	207616_s_at	-0.536281481	2.53E-06	TANK
4072	211999_at	-0.536107407	6.23E-07	H3F3A /// H3F3B /// MIR4738
4073	219162_s_at	-0.5358	8.90E-07	MRPL11
4074	219571_s_at	-0.535359259	1.32E-08	ZNF12
4075	236692_at	-0.535188889	2.11E-06	RP11-686D22.8
4076	201359_at	-0.535159259	3.93E-06	COPB1
4077	232524_x_at	-0.535025926	6.97E-07	ANAPC4
4078	202839_s_at	-0.53422963	4.04E-06	NDUFB7
4079	221816_s_at	-0.534096296	4.10E-07	PHF11
4080	200829_x_at	-0.534051852	2.41E-09	ZNF207
4081	209112_at	-0.533762963	3.27E-06	CDKN1B
4082	200877_at	-0.533233333	3.76E-08	CCT4
4083	222839_s_at	-0.533144444	7.75E-06	PAPOLG
4084	224369_s_at	-0.532496296	5.69E-06	FBXO38
4085	231718_at	-0.531811111	8.32E-07	SLU7
4086	1569057_s_at	-0.531655556	6.46E-06	MIA3
4087	233080_s_at	-0.531433333	1.74E-07	PRPF40A
4088	201271_s_at	-0.531166667	2.52E-07	RALY
4089	209313_at	-0.530714815	1.43E-06	GPN1
4090	203049_s_at	-0.530096296	1.88E-06	TTC37
4091	211375_s_at	-0.529933333	5.94E-07	ILF3
4092	212885_at	-0.5297	2.29E-07	MPHOSPH10
4093	225053_at	-0.528903704	3.42E-06	CNOT7
4094	204791_at	-0.528266667	4.26E-06	NR2C1
4095	203848_at	-0.527888889	3.08E-06	AKAP8
4096	226508_at	-0.527807407	1.80E-06	PHC3
4097	209751_s_at	-0.527559259	9.26E-06	TRAPPC2 /// TRAPPC2P1
4098	225585_at	-0.527103704	4.57E-06	RAP2A
4099	202042_at	-0.526896296	1.15E-06	HARS /// LOC101928623
4100	209761_s_at	-0.526892593	6.78E-07	SP110
4101	200725_x_at	-0.526774074	1.31E-07	RPL10 /// SNORA70
4102	200873_s_at	-0.526637037	3.18E-07	CCT8
4103	227267_at	-0.525933333	9.65E-06	POC5
4104	211720_x_at	-0.52532963	8.41E-08	RPLP0
4105	217346_at	-0.523237037	8.72E-06	PPIAP21 /// PPIAP21
4106	204314_s_at	-0.521496296	2.08E-06	CREB1
4107	225614_at	-0.520933333	4.21E-06	SAAL1

4108	226434_at	-0.520251852	3.52E-08	PPP1R35
4109	202117_at	-0.519966667	2.08E-07	ARHGAP1
4110	218882_s_at	-0.519603704	3.57E-07	WDR3
4111	213243_at	-0.518874074	8.79E-06	VPS13B
4112	229447_x_at	-0.518551852	8.92E-09	NBPF10 /// NBPF11 /// NBPF8
4113	226536_at	-0.518322222	2.49E-06	NSMCE2
4114	218269_at	-0.51812963	1.67E-06	DROSHA
4115	203544_s_at	-0.517274074	3.22E-06	STAM
4116	217871_s_at	-0.517085185	2.89E-06	MIF
4117	222985_at	-0.51707037	1.33E-06	YWHAG
4118	208095_s_at	-0.5168	1.77E-08	SRP72
4119	205849_s_at	-0.516474074	4.69E-06	UQCRB
4120	203776_at	-0.516266667	9.87E-07	GPKOW
4121	224562_at	-0.516111111	3.72E-07	WASF2
4122	208935_s_at	-0.515525926	4.91E-06	LGALS8
4123	1555730_a_at	-0.514988889	7.27E-06	CFL1
4124	231870_s_at	-0.514662963	9.58E-07	NMD3
4125	213677_s_at	-0.513488889	3.47E-07	PMS1
4126	1555889_a_at	-0.512551852	9.89E-06	CRTAP
4127	219067_s_at	-0.511622222	7.20E-06	NSMCE4A
4128	224880_at	-0.511485185	4.08E-07	RALA
4129	201303_at	-0.510514815	5.79E-06	EIF4A3
4130	201993_x_at	-0.509774074	2.03E-07	HNRNPDL
4131	36030_at	-0.509248148	8.58E-06	IFFO1
4132	224781_s_at	-0.50887037	5.01E-06	RBM17
4133	202021_x_at	-0.50862963	3.74E-06	EIF1
4134	212402_at	-0.508025926	4.57E-06	ZC3H13
4135	224206_x_at	-0.507674074	7.87E-07	MYNN
4136	203734_at	-0.506837037	2.11E-06	FOXJ2
4137	214714_at	-0.506285185	7.93E-06	ZNF394
4138	224630_at	-0.504025926	2.06E-06	ERLEC1
4139	215450_at	-0.503966667	3.05E-06	SNRPE
4140	214749_s_at	-0.503822222	7.10E-06	ARMCX6
4141	214794_at	-0.503192593	4.37E-06	PA2G4
4142	211933_s_at	-0.502333333	6.29E-07	HNRNPA3 /// HNRNPA3P1
4143	200093_s_at	-0.502118519	7.93E-07	HINT1
4144	202981_x_at	-0.501785185	4.15E-07	SIAH1
4145	208985_s_at	-0.501681481	7.16E-07	EIF3J
4146	224621_at	-0.50107037	1.04E-06	MAPK1
4147	200066_at	-0.500992593	8.28E-06	IK
4148	201245_s_at	-0.500555556	3.52E-06	LOC101927673 /// OTUB1
4149	200040_at	-0.500451852	5.02E-09	KHDRBS1
4150	212437_at	-0.499785185	2.26E-07	CENPB

4151	202144_s_at	-0.499744444	6.95E-07	ADSL
4152	200092_s_at	-0.499607407	4.47E-08	RPL37
4153	228495_at	-0.499288889	4.09E-06	GPATCH11
4154	223431_at	-0.499144444	2.61E-06	BLOC1S4
4155	227369_at	-0.498940741	1.45E-06	SERBP1
4156	200663_at	-0.497011111	4.37E-07	CD63
4157	224586_x_at	-0.494444444	1.13E-07	SUB1
4158	225144_at	-0.494392593	2.19E-06	BMPR2
4159	212015_x_at	-0.494325926	1.77E-06	MIR4745 /// PTBP1
4160	211746_x_at	-0.494288889	7.14E-06	PSMA1
4161	209467_s_at	-0.493496296	8.30E-06	MKNK1
4162	200044_at	-0.493274074	1.65E-06	GATC /// SRSF9
4163	224450_s_at	-0.493159259	3.29E-06	RIOK1
4164	200726_at	-0.492840741	1.81E-06	PPP1CC
4165	201039_s_at	-0.49257037	8.21E-06	RAD23A
4166	201240_s_at	-0.492188889	9.05E-07	SPCS2
4167	200888_s_at	-0.492137037	3.68E-08	RPL23 /// SNORA21
4168	212910_at	-0.492092593	8.21E-06	THAP11
4169	200679_x_at	-0.49102963	5.91E-07	HMGB1
4170	215148_s_at	-0.490440741	2.04E-06	APBA3
4171	223331_s_at	-0.4901	6.02E-06	DDX20
4172	225452_at	-0.490092593	1.37E-06	MED1
4173	218005_at	-0.489866667	4.58E-06	ZNF22
4174	223157_at	-0.489481481	5.69E-06	NOA1
4175	212130_x_at	-0.488985185	5.51E-06	EIF1
4176	223073_at	-0.488962963	1.39E-06	HIATL1
4177	217837_s_at	-0.488048148	3.09E-06	CHMP3 /// RNF103-CHMP3
4178	207983_s_at	-0.486740741	2.93E-06	STAG2
4179	221647_s_at	-0.486574074	2.13E-06	MIR6743 /// RIC8A
4180	218672_at	-0.486418519	1.46E-06	SCNM1 /// TNFAIP8L2-SCNM1
4181	201129_at	-0.485785185	3.23E-06	SRSF7
4182	204658_at	-0.485125926	4.63E-06	TRA2A
4183	206621_s_at	-0.485111111	4.75E-07	EIF4H
4184	208929_x_at	-0.48372963	1.97E-07	RPL13 /// SNORD68
4185	224899_s_at	-0.483222222	7.38E-06	MAGT1
4186	225210_s_at	-0.482455556	6.38E-06	FAM103A1
4187	31807_at	-0.482455556	3.64E-06	DDX49
4188	205930_at	-0.482051852	4.90E-06	GTF2E1
4189	222669_s_at	-0.481366667	1.03E-06	SBDS /// SBDSP1
4190	205545_x_at	-0.478248148	3.15E-06	DNAJC8
4191	1555961_a_at	-0.47737037	5.05E-07	HINT1
4192	202176_at	-0.47737037	6.16E-07	ERCC3
4193	200058_s_at	-0.476974074	7.22E-09	LOC101929240 /// SNRNP200

4194	218799_at	-0.476962963	5.74E-06	GPN2
4195	217954_s_at	-0.475366667	1.70E-06	PHF3
4196	200038_s_at	-0.475162963	1.12E-07	C18orf32 /// RPL17 /// RPL17- C18orf32 /// SNORD58A /// SNORD58B
4197	200084_at	-0.474696296	3.40E-06	C11orf58
4198	224670_at	-0.473866667	2.33E-06	SYS1
4199	222984_at	-0.473644444	2.10E-07	PAIP2
4200	229120_s_at	-0.473411111	1.17E-06	CDC42SE1
4201	224628_at	-0.47177037	7.14E-07	ERLEC1
4202	1554480_a_at	-0.471322222	3.75E-06	ARMC10
4203	211271_x_at	-0.471240741	4.39E-06	MIR4745 /// PTBP1
4204	201054_at	-0.470914815	9.81E-06	HNRNPA0
4205	222631_at	-0.47082963	8.79E-06	PI4K2B /// SEPSECS-AS1
4206	212799_at	-0.469922222	6.18E-07	STX6
4207	201592_at	-0.469307407	9.67E-06	EIF3H
4208	223705_s_at	-0.466518519	1.96E-07	GPBP1
4209	202076_at	-0.465418519	1.24E-06	BIRC2
4210	202653_s_at	-0.465074074	4.82E-06	7-Mar
4211	213628_at	-0.4644	7.97E-07	CLCC1
4212	226290_at	-0.463737037	3.71E-06	BDP1
4213	203048_s_at	-0.461303704	6.82E-06	TTC37
4214	1557966_x_at	-0.459511111	6.41E-06	MTERF4
4215	200062_s_at	-0.45947037	1.15E-06	RPL30
4216	238768_at	-0.458622222	3.31E-06	C2orf68
4217	208828_at	-0.458418519	5.52E-07	POLE3
4218	217740_x_at	-0.458348148	2.30E-07	RPL7A /// SNORD24 /// SNORD36A /// SNORD36B
4219	207181_s_at	-0.457518519	6.65E-06	CASP7
4220	214113_s_at	-0.45652963	1.58E-06	RBM8A
4221	212408_at	-0.453874074	9.69E-06	TOR1AIP1
4222	221798_x_at	-0.453781481	1.36E-07	RP5-882O7.1
4223	224892_at	-0.453381481	2.62E-06	BLOC1S6
4224	207541_s_at	-0.452192593	4.18E-06	EXOSC10
4225	200091_s_at	-0.451981481	1.30E-06	RPS25
4226	209239_at	-0.450607407	7.29E-06	NFKB1
4227	218243_at	-0.445951852	2.87E-07	RUFY1
4228	212440_at	-0.44462963	2.79E-06	SNRNP27
4229	208766_s_at	-0.444285185	7.65E-09	HNRNPR
4230	208826_x_at	-0.441748148	7.42E-06	HINT1
4231	212006_at	-0.441718519	1.03E-06	UBXN4
4232	212933_x_at	-0.440385185	6.58E-06	RPL13 /// SNORD68
4233	210908_s_at	-0.438162963	6.06E-07	PFDN5
4234	202184_s_at	-0.437518519	9.28E-06	NUP133

4235	212195_at	-0.437466667	5.24E-06	IL6ST
4236	217822_at	-0.436562963	2.34E-06	WBP11
4237	213251_at	-0.435744444	1.73E-06	SMARCA5
4238	209484_s_at	-0.433755556	6.68E-07	NSL1
4239	233642_s_at	-0.433740741	3.03E-06	HEATR5B
4240	200013_at	-0.433337037	7.17E-07	RPL24
4241	201600_at	-0.433048148	1.85E-07	PHB2
4242	201290_at	-0.432940741	4.34E-08	SEC11A
4243	201483_s_at	-0.431596296	6.50E-06	SUPT4H1
4244	213881_x_at	-0.431422222	3.07E-07	LOC101929087 /// SUMO2 /// SUMO3
4245	227567_at	-0.430211111	4.77E-06	LINC00674
4246	202029_x_at	-0.429481481	9.51E-07	RPL38
4247	200763_s_at	-0.429051852	5.53E-08	RPLP1
4248	203406_at	-0.426207407	7.85E-07	MFAP1
4249	221267_s_at	-0.424844444	6.41E-07	ABHD17A
4250	215884_s_at	-0.424566667	8.88E-06	UBQLN2
4251	218682_s_at	-0.421614815	8.13E-07	SLC4A1AP
4252	204145_at	-0.420503704	2.09E-06	FRG1 /// LOC100289097 /// LOC101930278 /// LOC101930531
4253	211270_x_at	-0.414855556	8.21E-06	PTBP1
4254	201488_x_at	-0.414040741	3.75E-06	KHDRBS1
4255	221700_s_at	-0.413062963	1.91E-06	UBA52
4256	209171_at	-0.41292963	6.96E-06	ITPA
4257	216306_x_at	-0.411866667	3.12E-06	MIR4745 /// PTBP1
4258	202333_s_at	-0.410711111	8.58E-06	UBE2B
4259	201344_at	-0.410537037	4.96E-07	UBE2D2
4260	225405_at	-0.409422222	7.67E-06	HNRNPUL2
4261	200600_at	-0.408681481	3.62E-06	MSN
4262	212780_at	-0.408192593	3.18E-06	SOS1
4263	210817_s_at	-0.407285185	5.73E-06	CALCOCO2
4264	209033_s_at	-0.40622963	8.32E-06	DYRK1A
4265	211378_x_at	-0.403377778	1.83E-06	LOC101060363 /// PPIA
4266	200099_s_at	-0.40292963	6.90E-06	RPS3A /// SNORD73A
4267	212734_x_at	-0.402777778	8.06E-06	RPL13 /// SNORD68
4268	200081_s_at	-0.39762963	7.86E-06	RPS6
4269	218604_at	-0.39717037	2.60E-06	LEMD3
4270	201103_x_at	-0.396755556	7.13E-07	NBPF10 /// NBPF11 /// NBPF12 /// NBPF14 /// NBPF15 /// NBPF20 /// NBPF8 /// NBPF9
4271	216326_s_at	-0.396248148	9.62E-06	HDAC3
4272	200926_at	-0.395911111	1.66E-06	RPS23
4273	200792_at	-0.394492593	2.69E-06	XRCC6
4274	201293_x_at	-0.394233333	4.14E-06	LOC101060363 /// PPIA
4275	200005_at	-0.3931	2.75E-07	EIF3D

4276	225925_s_at	-0.391733333	5.80E-06	USP48
4277	211765_x_at	-0.391392593	3.77E-06	PPIA
4278	200089_s_at	-0.388696296	1.47E-06	RPL4 /// SNORD16 /// SNORD18A /// SNORD18B /// SNORD18C
4279	212042_x_at	-0.379948148	2.75E-06	RPL7
4280	211942_x_at	-0.379551852	5.24E-06	RPL13A /// RPL13AP5 /// RPL13AP6 /// SNORD32A /// SNORD33 /// SNORD34 /// SNORD35A
4281	208635_x_at	-0.378177778	7.63E-06	NACA
4282	201086_x_at	-0.376355556	3.08E-06	SON
4283	211978_x_at	-0.371488889	5.62E-06	LOC101060363 /// PPIA
4284	216457_s_at	-0.370833333	5.13E-06	SF3A1
4285	200680_x_at	-0.363755556	3.00E-06	HMGB1
4286	208640_at	-0.358344444	3.73E-06	RAC1
4287	213941_x_at	-0.349292593	3.74E-06	RPS7
4288	203648_at	0.388533333	8.72E-06	TATDN2
4289	223309_x_at	0.402411111	7.55E-06	PNPLA8
4290	226987_at	0.407488889	5.93E-07	RBM15B
4291	210249_s_at	0.411748148	5.19E-06	NCOA1
4292	224737_x_at	0.415566667	7.10E-06	CCAR1
4293	229742_at	0.417148148	6.03E-06	C15orf61
4294	213538_at	0.418318519	8.14E-06	SON
4295	217927_at	0.418333333	9.11E-06	SPCS1
4296	201022_s_at	0.418903704	4.60E-06	DSTN
4297	224493_x_at	0.4199	1.49E-06	TMEM241
4298	217852_s_at	0.420474074	9.15E-07	ARL8B
4299	202753_at	0.422796296	4.34E-06	PSMD6
4300	212034_s_at	0.423292593	3.78E-06	EXOC7
4301	226874_at	0.424277778	2.18E-06	KLHL8
4302	222125_s_at	0.4257	3.20E-06	P4HTM
4303	208598_s_at	0.426885185	3.01E-06	HUWE1
4304	212643_at	0.428588889	5.72E-06	MAPK1IP1L
4305	200721_s_at	0.436237037	2.46E-06	ACTR1A
4306	202681_at	0.4369	1.72E-06	C3orf62 /// MIR4271 /// USP4
4307	200915_x_at	0.439522222	5.99E-06	KTN1
4308	218020_s_at	0.44662963	1.10E-06	ZFAND3
4309	226370_at	0.446659259	5.34E-06	KLHL15
4310	219816_s_at	0.448196296	1.72E-06	RBM23
4311	206323_x_at	0.451451852	1.49E-06	OPHN1
4312	217783_s_at	0.452937037	3.49E-07	YPEL5
4313	32099_at	0.456740741	6.33E-06	SAFB2
4314	40829_at	0.457159259	4.38E-08	WDTC1
4315	202797_at	0.457314815	7.57E-06	SACM1L

4316	225338_at	0.457722222	1.81E-06	ZYG11B
4317	205851_at	0.457774074	1.05E-06	NME6
4318	221791_s_at	0.458788889	2.83E-07	TMA7
4319	218043_s_at	0.45962963	9.16E-06	AZI2
4320	1555883_s_at	0.461803704	7.00E-06	SPIN3
4321	228130_at	0.461814815	7.08E-06	ZSCAN30
4322	202713_s_at	0.462951852	4.25E-06	KIAA0391 /// PSMA6
4323	202682_s_at	0.463603704	3.13E-07	USP4
4324	226607_at	0.467062963	4.45E-06	C20orf194
4325	225014_at	0.46812963	9.95E-07	SMIM20
4326	202488_s_at	0.468207407	5.79E-06	FXYD3
4327	226126_at	0.46917037	3.07E-07	TBCK
4328	222618_at	0.470181481	3.76E-06	SMU1
4329	208881_x_at	0.473014815	1.54E-06	IDI1
4330	228041_at	0.473522222	8.32E-07	AASDH
4331	211034_s_at	0.479077778	6.16E-07	HECTD4
4332	202845_s_at	0.481155556	5.85E-06	RALBP1
4333	211987_at	0.482744444	1.99E-06	TOP2B
4334	204308_s_at	0.48327037	7.93E-06	TECPR2
4335	212517_at	0.483307407	8.18E-06	ATRN
4336	225492_at	0.484885185	6.96E-06	TMEM33
4337	235792_x_at	0.485585185	6.19E-07	PIK3C2A
4338	212888_at	0.486348148	1.66E-06	DICER1
4339	226309_at	0.486403704	3.81E-06	DNAL1
4340	212605_s_at	0.487185185	3.45E-06	NUDT3
4341	219706_at	0.490633333	3.51E-07	AP5S1
4342	212585_at	0.492118519	3.30E-06	OSBPL8
4343	203487_s_at	0.492414815	6.02E-06	ARMC8
4344	225243_s_at	0.492551852	7.00E-07	SLMAP
4345	218544_s_at	0.492877778	1.10E-07	RCL1
4346	228070_at	0.493974074	4.40E-07	PPP2R5E
4347	201230_s_at	0.494074074	6.60E-07	ARIH2
4348	212222_at	0.494403704	1.85E-06	PSME4
4349	212436_at	0.495774074	7.58E-06	TRIM33
4350	225351_at	0.496559259	1.14E-06	FAM45A /// FAM45B
4351	203959_s_at	0.497766667	2.75E-07	ZBTB40
4352	202325_s_at	0.497785185	9.87E-08	ATP5J
4353	223141_at	0.498496296	3.85E-06	UCK1
4354	38710_at	0.498674074	1.60E-06	LOC101927673 /// OTUB1
4355	200694_s_at	0.501074074	6.79E-07	DDX24
4356	201800_s_at	0.501340741	1.91E-06	OSBP
4357	208632_at	0.502877778	1.87E-06	RNF10
4358	222445_at	0.503166667	6.13E-07	SLC39A9

4359	222674_at	0.503322222	2.56E-06	C9orf114
4360	223310_x_at	0.504377778	3.26E-07	PNPLA8
4361	218200_s_at	0.506351852	2.13E-06	NDUFB2
4362	217714_x_at	0.508048148	7.83E-07	MIR3917 /// STMN1
4363	224704_at	0.508340741	8.65E-06	TNRC6A
4364	200848_at	0.509255556	4.78E-06	AHCYL1
4365	226481_at	0.509533333	1.42E-07	VPRBP
4366	225011_at	0.511048148	4.65E-09	PRKAR2A
4367	50376_at	0.511325926	2.56E-06	ZNF444
4368	224993_at	0.512851852	4.20E-07	MLLT1
4369	40562_at	0.513485185	8.86E-09	GNA11
4370	202428_x_at	0.514	9.81E-06	DBI
4371	203831_at	0.514307407	5.38E-07	R3HDM2
4372	1552863_a_at	0.514459259	7.86E-06	CACNG6
4373	224319_s_at	0.515692593	4.92E-06	MIEF1
4374	201229_s_at	0.517222222	5.21E-07	ARIH2
4375	218306_s_at	0.517548148	2.94E-07	HERC1
4376	232899_at	0.517733333	1.35E-06	LOC101929177
4377	238893_at	0.520525926	5.29E-06	LINC00936
4378	201227_s_at	0.521222222	2.89E-06	NDUFB8
4379	217940_s_at	0.522540741	2.02E-06	CARKD
4380	202209_at	0.52272963	2.73E-06	LSM3
4381	227869_at	0.522996296	5.42E-07	FAM104B
4382	218601_at	0.523218519	5.26E-06	URGCP
4383	202136_at	0.523251852	5.89E-08	ZMYND11
4384	225350_s_at	0.523411111	1.88E-06	ZYG11B
4385	225862_at	0.524692593	8.33E-07	SLC25A26
4386	200642_at	0.5251	2.16E-06	SOD1
4387	213161_at	0.527040741	1.40E-06	TMOD1 /// TSTD2
4388	212359_s_at	0.527292593	4.58E-06	ZSWIM8
4389	220143_x_at	0.5274	1.71E-06	LUC7L
4390	216267_s_at	0.528592593	2.33E-07	TMEM115
4391	218483_s_at	0.529207407	7.17E-07	IFT46
4392	36566_at	0.529548148	1.46E-07	CTNS
4393	225276_at	0.531240741	2.96E-06	GSPT1
4394	217844_at	0.53282963	3.26E-06	CTDSP1
4395	223424_s_at	0.534514815	1.34E-06	ZSCAN21
4396	217045_x_at	0.534533333	1.60E-06	NCR2
4397	208405_s_at	0.53497037	3.36E-07	CD164
4398	226119_at	0.536911111	2.64E-07	PCMTD1
4399	218071_s_at	0.539837037	4.04E-08	MKRN2
4400	218671_s_at	0.540503704	8.70E-06	ATPIF1
4401	202460_s_at	0.540737037	4.12E-06	LPIN2

4402	201439_at	0.541103704	2.72E-06	GBF1
4403	229863_s_at	0.541118519	8.71E-06	ELP6
4404	208899_x_at	0.541162963	1.36E-06	ATP6V1D
4405	212714_at	0.541255556	6.48E-06	LARP4
4406	218174_s_at	0.542385185	4.37E-06	TMEM254
4407	201394_s_at	0.542607407	2.19E-07	RBM5
4408	219581_at	0.545103704	9.09E-06	TSEN2
4409	214679_x_at	0.545507407	5.16E-08	GNA11
4410	213039_at	0.545525926	1.34E-06	ARHGEF18
4411	33814_at	0.545540741	5.54E-07	PAK4
4412	212693_at	0.545877778	2.26E-06	MDN1
4413	201717_at	0.546111111	1.46E-07	MRPL49
4414	210775_x_at	0.546285185	1.57E-07	CASP9
4415	35265_at	0.546377778	6.89E-06	FXR2
4416	202110_at	0.546803704	2.73E-06	COX7B
4417	218780_at	0.549133333	9.35E-06	HOOK2
4418	220734_s_at	0.549551852	9.92E-06	CPTP
4419	206357_at	0.550081481	7.48E-07	OPA3
4420	206096_at	0.550114815	2.67E-07	ZNF35
4421	227712_at	0.550137037	1.50E-07	LYRM2
4422	213853_at	0.550407407	2.41E-06	DNAJC24
4423	235507_at	0.550755556	1.33E-07	PCMTD1
4424	227251_at	0.550922222	1.92E-06	DCAF5
4425	228446_at	0.553037037	7.63E-07	KIAA2026
4426	222143_s_at	0.556311111	4.92E-07	MTMR14
4427	228612_at	0.559025926	7.78E-07	RAB30-AS1
4428	242048_at	0.559451852	2.54E-06	MIR6834 /// PFDN6
4429	223027_at	0.559577778	1.66E-06	SNX9
4430	235917_at	0.559644444	9.98E-06	INIP
4431	209665_at	0.561022222	1.46E-06	CYB561D2
4432	222404_x_at	0.561196296	9.08E-07	PTPLAD1
4433	224907_s_at	0.561896296	5.23E-07	SH3GLB2
4434	208647_at	0.562248148	4.54E-07	FDFT1
4435	201728_s_at	0.562533333	5.66E-06	KIAA0100
4436	208248_x_at	0.562966667	2.59E-07	APLP2
4437	208678_at	0.563325926	3.30E-06	ATP6V1E1
4438	202624_s_at	0.563355556	2.07E-06	CABIN1
4439	222742_s_at	0.563625926	3.89E-07	IFT22
4440	203566_s_at	0.5656	7.16E-06	AGL
4441	202738_s_at	0.565911111	3.04E-07	PHKB
4442	230122_at	0.566548148	6.10E-06	MLLT10
4443	230727_at	0.566840741	5.23E-06	CISD3
4444	1554178_a_at	0.567066667	5.00E-06	FAM126B

4445	221963_x_at	0.567074074	6.20E-06	ZNF587B
4446	226078_at	0.568744444	1.70E-06	RPUSD1
4447	229025_s_at	0.568918519	8.17E-09	IMMP1L
4448	229664_at	0.569362963	4.10E-08	MAPK8
4449	219157_at	0.570162963	7.83E-06	KLHL2
4450	202125_s_at	0.570355556	6.42E-06	TRAK2
4451	234798_x_at	0.571303704	9.83E-06	
4452	201244_s_at	0.571607407	8.75E-09	RAF1
4453	201178_at	0.573177778	5.08E-07	FBXO7
4454	213185_at	0.573196296	1.61E-06	KIAA0556
4455	210671_x_at	0.574033333	7.61E-06	MAPK8
4456	201322_at	0.574137037	3.66E-06	ATP5B
4457	208870_x_at	0.574325926	4.42E-09	ATP5C1
4458	235399_at	0.5748	7.30E-06	ERCC2
4459	32836_at	0.575307407	9.72E-08	AGPAT1
4460	240988_x_at	0.576381481	1.23E-06	
4461	239197_s_at	0.5764	8.62E-07	EZH1
4462	228254_at	0.577133333	4.87E-06	STAM2
4463	202358_s_at	0.577225926	6.67E-07	SNX19
4464	209563_x_at	0.578374074	3.05E-06	CALM1 /// CALM2 /// CALM3
4465	213766_x_at	0.579677778	1.72E-08	GNA11
4466	207801_s_at	0.580003704	4.96E-07	RNF10
4467	227528_s_at	0.580555556	4.49E-07	KMT2D
4468	218620_s_at	0.581366667	1.41E-06	HEMK1
4469	40489_at	0.581462963	1.62E-06	ATN1
4470	202279_at	0.583007407	2.60E-06	C14orf2
4471	202109_at	0.583496296	3.31E-08	ARFIP2
4472	225131_at	0.584240741	1.07E-08	ZRANB1
4473	218563_at	0.584251852	5.89E-06	NDUFA3
4474	212870_at	0.584514815	1.30E-06	SOS2
4475	40612_at	0.584988889	2.08E-06	DOPEY1
4476	219754_at	0.585474074	4.70E-07	RBM41
4477	207625_s_at	0.585755556	2.52E-08	CBFA2T2
4478	219133_at	0.586240741	6.08E-10	OXSM
4479	203529_at	0.586318519	5.19E-09	PPP6C
4480	227047_x_at	0.586366667	7.04E-07	ZBTB4
4481	219172_at	0.586477778	6.34E-07	UBTD1
4482	212214_at	0.58677037	5.25E-06	OPA1
4483	223155_at	0.586955556	6.82E-07	HDHD2
4484	207667_s_at	0.587022222	6.97E-06	LOC100996792 /// MAP2K3
4485	234611_at	0.588351852	1.51E-06	LGALS8-AS1
4486	1553968_a_at	0.588492593	1.65E-07	ADAT3
4487	213989_x_at	0.588988889	3.77E-06	SETD4

4488	200804_at	0.589325926	7.56E-07	TMBIM6
4489	213366_x_at	0.58987037	9.11E-09	ATP5C1
4490	212908_at	0.591077778	9.77E-09	DNAJC16
4491	217144_at	0.591422222	4.16E-06	UBBP1 /// UBBP1 /// UBBP4 /// UBBP4
4492	224786_at	0.5925	8.80E-07	SCOC
4493	211252_x_at	0.59287037	3.20E-06	PTCRA
4494	228435_at	0.593040741	4.37E-06	AC005523.2
4495	223380_s_at	0.593062963	3.64E-06	LATS2
4496	200854_at	0.59307037	2.56E-06	NCOR1
4497	220671_at	0.593103704	1.31E-06	CCRN4L
4498	213196_at	0.593188889	1.84E-08	ZNF629
4499	1566512_at	0.593511111	4.78E-06	
4500	212627_s_at	0.593588889	2.61E-09	EXOSC7
4501	217782_s_at	0.593892593	2.66E-06	GPS1
4502	1552976_at	0.5951	2.70E-06	
4503	215582_x_at	0.595785185	2.88E-06	MCM3AP
4504	36129_at	0.596037037	3.69E-07	SGSM2
4505	204893_s_at	0.596503704	1.81E-06	ZFYVE9
4506	231974_at	0.596688889	3.34E-07	KMT2D
4507	203303_at	0.597674074	1.61E-08	DYNLT3
4508	201155_s_at	0.597985185	5.02E-06	MFN2
4509	202909_at	0.598118519	3.54E-08	EPM2AIP1
4510	229969_at	0.598811111	4.85E-06	SEC63
4511	214315_x_at	0.5994	2.08E-07	CALR
4512	223135_s_at	0.599481481	6.42E-09	BBX
4513	211852_s_at	0.599548148	3.31E-06	ATRN
4514	229293_at	0.599722222	5.18E-07	
4515	205437_at	0.600162963	4.87E-06	ZNF211
4516	217976_s_at	0.600481481	1.74E-06	DYNC1LI1
4517	210740_s_at	0.600625926	1.63E-06	ITPK1
4518	1558431_at	0.600937037	9.34E-06	NHLRC4
4519	209741_x_at	0.6011	2.08E-07	SCAPER
4520	209452_s_at	0.601318519	1.06E-06	VTI1B
4521	241968_at	0.6023	2.20E-07	C4orf29
4522	206520_x_at	0.602340741	7.39E-06	SIGLEC6
4523	218194_at	0.602707407	7.14E-06	REXO2
4524	223954_x_at	0.603214815	1.16E-06	NECAB3
4525	209322_s_at	0.603755556	5.37E-06	SH2B1
4526	226970_at	0.604162963	5.01E-07	FBXO33
4527	207966_s_at	0.604459259	1.35E-07	GLG1
4528	218184_at	0.604462963	2.37E-07	TULP4
4529	227728_at	0.605674074	1.66E-06	PPM1A
4530	203371_s_at	0.606062963	7.11E-07	NDUFB3

4531	212674_s_at	0.606414815	2.91E-07	DHX30
4532	225672_at	0.607022222	5.47E-08	GOLGA2
4533	213738_s_at	0.607185185	1.64E-06	ATP5A1
4534	208940_at	0.607207407	7.15E-07	SEPHS1
4535	225432_s_at	0.608166667	9.45E-08	CSRP2BP /// PET117
4536	224502_s_at	0.608248148	6.68E-06	KIAA1191
4537	212114_at	0.609433333	4.43E-09	ATXN7L3B
4538	217980_s_at	0.610662963	1.13E-06	MRPL16
4539	226833_at	0.610681481	8.33E-06	CYB5D1
4540	221949_at	0.6107	2.74E-06	UBE2D4
4541	227334_at	0.611925926	5.35E-07	USP54
4542	226324_s_at	0.612077778	4.95E-07	IFT172
4543	202245_at	0.61332963	6.91E-07	LSS
4544	207727_s_at	0.613377778	1.20E-07	MUTYH
4545	1553955_at	0.61342963	7.52E-06	PPP1R21
4546	212760_at	0.613440741	6.36E-06	UBR2
4547	226224_at	0.613640741	7.70E-08	FOXK2
4548	208420_x_at	0.613881481	5.04E-07	SUPT6H
4549	204925_at	0.615755556	5.12E-06	CTNS
4550	226116_at	0.616111111	1.42E-09	DFFA
4551	243492_at	0.616188889	9.51E-07	THEM4
4552	211976_at	0.616455556	3.61E-08	NUDT3
4553	1558568_a_at	0.617185185	1.89E-06	PCNXL4
4554	217904_s_at	0.617711111	8.65E-06	BACE1
4555	225810_at	0.618296296	1.52E-06	MTMR10
4556	224471_s_at	0.618433333	8.87E-07	BTRC
4557	207871_s_at	0.618748148	5.50E-07	MIR6132 /// ST7 /// ST7-OT3
4558	223277_at	0.618833333	1.85E-09	ELP6
4559	34478_at	0.619033333	5.77E-06	RAB11B
4560	201738_at	0.62007037	1.53E-09	EIF1B
4561	238007_at	0.620251852	3.59E-07	ZNF271
4562	221804_s_at	0.620496296	1.14E-09	FAM45A /// FAM45B
4563	227385_at	0.621203704	2.04E-06	PPAPDC2
4564	205711_x_at	0.621325926	1.86E-08	ATP5C1
4565	202702_at	0.622162963	9.32E-07	TRIM26
4566	225745_at	0.622777778	4.86E-06	LRP6
4567	235078_at	0.623803704	5.71E-07	
4568	208238_x_at	0.625177778	3.40E-06	
4569	227219_x_at	0.625307407	2.92E-07	MAP1LC3A
4570	229043_at	0.625337037	3.09E-06	PAPD5
4571	221995_s_at	0.625559259	5.18E-06	
4572	201661_s_at	0.625718519	5.95E-07	ACSL3
4573	202614_at	0.625844444	4.28E-09	SLC30A9

4574	65630_at	0.626022222	6.24E-06	TMEM80
4575	49327_at	0.626074074	2.61E-07	SIRT3
4576	209249_s_at	0.626118519	7.83E-08	GHITM
4577	221486_at	0.626392593	6.66E-06	ENSA
4578	1555833_a_at	0.626674074	1.39E-09	IRGQ
4579	215566_x_at	0.627207407	1.09E-08	LYPLA2
4580	244354_at	0.628437037	9.71E-06	STRN
4581	202739_s_at	0.628633333	1.93E-07	PHKB
4582	226967_at	0.629325926	7.26E-06	FIZ1
4583	228512_at	0.629422222	4.52E-06	PTCD3
4584	205370_x_at	0.629607407	1.45E-06	DBT
4585	203174_s_at	0.629674074	1.01E-06	ARFRP1
4586	202380_s_at	0.630018519	9.70E-06	NKTR
4587	225859_at	0.630285185	7.05E-06	XIAP
4588	226938_at	0.631477778	1.13E-06	DCAF4
4589	216885_s_at	0.631592593	8.33E-07	DCAF8
4590	213944_x_at	0.631814815	5.49E-09	GNA11
4591	230282_at	0.632044444	8.96E-06	TSPAN3
4592	226734_at	0.632277778	4.89E-07	EIF4E2
4593	45526_g_at	0.632348148	6.98E-09	NAA60
4594	239056_at	0.632737037	2.09E-06	SEC22C
4595	225521_at	0.632859259	3.76E-06	ANAPC7
4596	208120_x_at	0.633	6.24E-07	FKSG49
4597	232145_at	0.633281481	4.61E-06	C2orf68
4598	207559_s_at	0.633692593	1.95E-07	ZMYM3
4599	233541_at	0.633966667	6.10E-06	LIMD1-AS1
4600	208847_s_at	0.634066667	5.18E-07	ADH5
4601	208703_s_at	0.634981481	3.80E-07	APLP2
4602	203984_s_at	0.635444444	1.10E-07	CASP9
4603	221516_s_at	0.635985185	6.03E-09	MIEF1
4604	235647_at	0.636122222	7.04E-06	AP4S1
4605	210266_s_at	0.63642963	1.97E-07	TRIM33
4606	212032_s_at	0.6369	9.06E-06	PTOV1
4607	224888_at	0.637022222	2.76E-06	EPT1
4608	224729_s_at	0.637140741	1.42E-07	ATPAF1
4609	209472_at	0.637166667	1.23E-06	CCBL2
4610	223128_at	0.637348148	3.95E-06	FOXRED1
4611	202562_s_at	0.637655556	3.68E-06	C14orf1
4612	226851_at	0.637792593	2.00E-09	LYPLAL1
4613	224378_x_at	0.638977778	3.61E-07	MAP1LC3A
4614	216993_s_at	0.639114815	3.52E-06	COL11A2
4615	229240_at	0.639737037	4.49E-07	ZDHHC21
4616	208804_s_at	0.640177778	9.36E-08	SRSF6

4617	202922_at	0.641425926	9.09E-07	GCLC
4618	201913_s_at	0.641751852	6.23E-07	COASY
4619	227968_at	0.642062963	1.24E-06	PDDC1
4620	204839_at	0.642292593	1.53E-08	POP5
4621	217501_at	0.643322222	8.41E-06	CIAO1
4622	226719_at	0.643325926	2.44E-06	DERL2
4623	226133_s_at	0.6434	6.36E-08	TBC1D10A
4624	204245_s_at	0.643811111	7.87E-08	RPP14
4625	217329_x_at	0.643840741	9.92E-07	COX7BP1 /// COX7BP1
4626	225425_s_at	0.644444444	1.45E-08	MRPL41
4627	202640_s_at	0.644674074	2.77E-07	RANBP3
4628	219583_s_at	0.644988889	6.74E-08	SPATA7
4629	227049_at	0.645151852	2.51E-07	ZADH2
4630	225418_at	0.645611111	5.62E-08	PVRL2
4631	212435_at	0.645640741	3.93E-09	TRIM33
4632	225772_s_at	0.645644444	1.61E-08	COX14
4633	221012_s_at	0.646151852	1.16E-09	TRIM8
4634	219016_at	0.646437037	3.70E-10	FASTKD5
4635	234977_at	0.646711111	5.81E-06	ZADH2
4636	230243_at	0.647014815	2.45E-06	TRMT10A
4637	1553974_at	0.648911111	1.26E-07	C22orf39
4638	216680_s_at	0.649	8.08E-06	EPHB4
4639	225771_at	0.649937037	1.89E-08	AP1G1
4640	213049_at	0.650251852	9.48E-08	RALGAPA1
4641	205417_s_at	0.650451852	2.62E-07	DAG1
4642	231839_at	0.650544444	4.79E-08	PDE12
4643	212493_s_at	0.652059259	6.47E-08	SETD2
4644	213787_s_at	0.65287037	7.97E-06	EBP
4645	225098_at	0.653622222	1.55E-07	ABI2
4646	210638_s_at	0.654159259	3.89E-09	FBXO9
4647	228536_at	0.654366667	3.94E-07	PRMT9
4648	564_at	0.654822222	4.64E-10	GNA11
4649	211630_s_at	0.655862963	1.77E-06	GSS
4650	218511_s_at	0.656837037	1.76E-06	PNPO
4651	1554523_a_at	0.656962963	6.03E-06	CNNM2
4652	228093_at	0.658596296	4.82E-08	ZNF599
4653	202281_at	0.658651852	2.25E-07	GAK
4654	216309_x_at	0.658655556	1.85E-06	JRK
4655	218328_at	0.659074074	1.86E-07	COQ4
4656	227479_at	0.660033333	1.92E-07	
4657	225270_at	0.660503704	8.37E-06	NEO1
4658	240794_at	0.660937037	2.48E-07	NPAS4
4659	219343_at	0.661174074	3.37E-06	CDC37L1

4660	213229_at	0.661307407	1.66E-07	DICER1
4661	204248_at	0.661737037	2.92E-07	GNA11
4662	228944_at	0.66177037	1.70E-06	RP4-773N10.4
4663	203803_at	0.662014815	5.12E-06	PCYOX1
4664	223091_x_at	0.662185185	2.13E-06	MFF
4665	227562_at	0.662214815	5.82E-06	LAMTOR3
4666	212001_at	0.663781481	2.80E-08	SUGP2
4667	210624_s_at	0.664548148	2.89E-08	ILVBL
4668	225743_at	0.665511111	3.22E-08	RPUSD3
4669	223324_s_at	0.66612963	8.47E-08	TRPM7
4670	212911_at	0.666185185	8.10E-08	DNAJC16
4671	226110_at	0.666192593	3.77E-06	PTAR1
4672	203551_s_at	0.66682963	9.12E-07	COX11
4673	218632_at	0.667251852	2.08E-08	HECTD3
4674	226629_at	0.667507407	1.35E-06	SLC43A2
4675	208928_at	0.668081481	3.03E-06	POR
4676	203532_x_at	0.668751852	9.88E-06	CUL5
4677	242028_at	0.669574074	4.55E-06	ZNF709
4678	231153_at	0.669840741	8.68E-06	C16orf86
4679	228125_at	0.669881481	7.68E-07	ZSCAN30
4680	213057_at	0.670281481	7.69E-06	ATPAF2
4681	1555906_s_at	0.671603704	8.55E-08	TCAIM
4682	212216_at	0.671922222	3.43E-06	PREPL
4683	213508_at	0.67302963	1.22E-09	SPTSSA
4684	225499_at	0.673714815	1.51E-06	RALGAPA2
4685	220352_x_at	0.6741	3.57E-06	FLJ42627
4686	226011_at	0.674144444	9.54E-09	CCDC12
4687	226484_at	0.675359259	5.88E-07	ZBTB47
4688	216119_s_at	0.675407407	1.25E-06	SPEF1
4689	226095_s_at	0.675859259	2.81E-08	ATXN1L
4690	206790_s_at	0.675911111	1.01E-09	NDUFB1
4691	225825_at	0.67627037	2.52E-09	C20orf194
4692	206414_s_at	0.677077778	1.61E-06	ASAP2
4693	224620_at	0.678562963	9.42E-06	MAPK1
4694	1552942_at	0.678614815	5.57E-07	LOC149373
4695	239198_at	0.678640741	6.87E-07	EZH1
4696	238777_x_at	0.678696296	1.85E-07	ATMIN
4697	217808_s_at	0.678955556	4.25E-07	MAPKAP1
4698	209163_at	0.679003704	6.61E-07	CYB561
4699	215903_s_at	0.67927037	8.24E-07	MAST2
4700	237419_at	0.67932963	4.84E-06	RP11-722E23.2
4701	225197_at	0.681088889	2.86E-08	RP11-473I1.9
4702	203524_s_at	0.681248148	1.59E-07	MPST

4703	218684_at	0.681292593	2.40E-07	LRRC8D
4704	242989_at	0.681374074	2.86E-07	STRN
4705	226193_x_at	0.681788889	2.79E-07	CBWD1 /// CBWD2 /// CBWD3 /// CBWD5 /// CBWD6 /// CBWD7 /// LOC101060578
4706	203273_s_at	0.682548148	3.97E-11	TUSC2
4707	214924_s_at	0.682744444	9.28E-07	TRAK1
4708	204305_at	0.683122222	5.52E-06	MIPEP
4709	212309_at	0.683218519	8.14E-09	CLASP2
4710	238043_at	0.683614815	3.98E-08	ARID1B
4711	208184_s_at	0.683677778	8.61E-06	LOC102724200 /// TRAPPC10
4712	1566549_at	0.683711111	3.01E-06	
4713	202455_at	0.684162963	5.24E-06	HDAC5
4714	217860_at	0.684492593	9.86E-07	NDUFA10
4715	214785_at	0.684685185	6.91E-06	VPS13A
4716	213149_at	0.684951852	2.49E-06	DLAT
4717	225616_at	0.685396296	1.50E-07	SPRYD4
4718	224886_at	0.685996296	7.03E-06	JMJD8
4719	212817_at	0.687259259	7.27E-06	DNAJB5
4720	233775_x_at	0.687651852	5.98E-06	LOC100289333
4721	218298_s_at	0.688166667	2.92E-07	C14orf159
4722	221692_s_at	0.688177778	3.56E-06	MRPL34
4723	203775_at	0.688307407	1.24E-06	SLC25A13
4724	223292_s_at	0.688503704	1.12E-06	MRPS15
4725	214436_at	0.688585185	1.78E-06	FBXL2
4726	213132_s_at	0.688644444	5.80E-08	MCAT
4727	213242_x_at	0.68877037	7.13E-07	CEP170B
4728	209234_at	0.688814815	6.41E-07	KIF1B
4729	228775_at	0.688825926	1.92E-08	EMC3
4730	201814_at	0.689081481	2.98E-10	TBC1D5
4731	209018_s_at	0.690162963	5.06E-07	PINK1
4732	226957_x_at	0.690244444	2.67E-06	RALBP1
4733	218125_s_at	0.690348148	1.16E-07	CCDC25
4734	213221_s_at	0.690707407	1.14E-06	SIK2
4735	219174_at	0.69112963	2.24E-07	IFT74
4736	214239_x_at	0.691248148	2.74E-06	PCGF2
4737	218121_at	0.691566667	6.70E-07	HMOX2
4738	223473_at	0.691811111	4.55E-06	MPV17L2
4739	213195_at	0.692411111	4.54E-06	LYRM9
4740	226692_at	0.692985185	8.12E-07	HYPK /// MIR1282 /// SERF2
4741	200980_s_at	0.6931	4.57E-07	PDHA1
4742	218290_at	0.693433333	1.77E-08	MIR6789 /// PLEKHJ1
4743	211136_s_at	0.69417037	3.87E-06	CLPTM1
4744	223378_at	0.694537037	1.19E-06	GLIS2

4745	226377_at	0.696744444	2.12E-06	NFIC
4746	204691_x_at	0.697088889	2.90E-09	PLA2G6
4747	222707_s_at	0.697162963	5.18E-11	ACTR8
4748	231061_at	0.698166667	1.14E-06	MIR302B
4749	218502_s_at	0.6985	1.01E-06	TRPS1
4750	202300_at	0.698774074	5.73E-08	LAMTOR5
4751	226369_at	0.699237037	3.52E-06	LINC01089
4752	221656_s_at	0.699792593	7.54E-08	ARHGEF10L
4753	203244_at	0.700296296	2.97E-07	PEX5
4754	220741_s_at	0.700785185	4.82E-09	PPA2
4755	36994_at	0.701048148	7.64E-09	ATP6V0C
4756	213083_at	0.701322222	2.84E-06	SLC35D2
4757	223202_s_at	0.701474074	6.25E-06	LOC101928589 /// TMEM164
4758	1553118_at	0.7016	4.70E-06	THEM4
4759	204355_at	0.702240741	1.16E-10	DHX30
4760	200978_at	0.702559259	2.32E-07	MDH1
4761	58900_at	0.7037	4.91E-09	UBE2D4
4762	209479_at	0.703985185	1.39E-06	CCDC28A
4763	224346_at	0.704148148	5.01E-07	
4764	229852_at	0.704351852	1.66E-08	NMNAT1
4765	215549_x_at	0.70452963	1.78E-06	CTAGE4 /// CTAGE8 /// CTAGE9
4766	215938_s_at	0.704662963	9.81E-06	PLA2G6
4767	236515_at	0.704722222	3.76E-07	
4768	227581_at	0.704892593	4.46E-06	TECPR1
4769	212603_at	0.705377778	5.90E-08	MRPS31
4770	222056_s_at	0.706166667	8.66E-09	FAHD2A
4771	1569679_at	0.706285185	4.64E-06	CDH22
4772	243835_at	0.7063	6.21E-07	ZDHHC21
4773	1556422_at	0.707411111	6.31E-06	AC007228.11 /// BX647249
4774	1560446_at	0.707474074	1.83E-06	IPO5P1
4775	206194_at	0.707914815	1.51E-06	HOXC4
4776	227186_s_at	0.708074074	2.00E-07	MRPL41
4777	213675_at	0.708155556	4.13E-06	PARVA
4778	218739_at	0.708188889	8.49E-08	ABHD5
4779	229762_at	0.70847037	1.48E-06	
4780	221006_s_at	0.70902963	2.15E-07	SNX27
4781	215909_x_at	0.709551852	1.55E-07	MINK1
4782	208909_at	0.709659259	5.62E-09	UQCRFS1
4783	218265_at	0.711333333	5.22E-08	SECISBP2
4784	212669_at	0.711540741	1.51E-08	CAMK2G
4785	212987_at	0.711714815	1.21E-09	FBXO9
4786	225182_at	0.714837037	9.67E-09	TMEM50B
4787	203081_at	0.714851852	3.86E-07	CTNNBIP1

4788	232373_at	0.7151	3.36E-07	NOXA1
4789	223459_s_at	0.715340741	9.77E-08	C1orf56
4790	202000_at	0.716007407	1.67E-09	NDUFA6
4791	218653_at	0.716159259	5.00E-06	SLC25A15
4792	201826_s_at	0.716592593	8.55E-08	SCCPDH
4793	212450_at	0.717588889	9.08E-10	SECISBP2L
4794	223507_at	0.71807037	4.42E-13	CLPX
4795	222220_s_at	0.7188	8.05E-06	TSNAXIP1
4796	212993_at	0.719	9.92E-08	NACC2
4797	228020_at	0.719177778	4.50E-06	PTCD3
4798	226929_at	0.719788889	1.05E-07	MTHFR
4799	214857_at	0.719962963	6.06E-08	RPARP-AS1
4800	226843_s_at	0.720903704	5.37E-06	PAPD5
4801	205233_s_at	0.721203704	1.59E-06	PAFAH2
4802	210695_s_at	0.721655556	1.00E-08	WWOX
4803	230813_at	0.722440741	1.35E-06	LEPREL1
4804	204129_at	0.722825926	2.14E-08	BCL9
4805	218887_at	0.723159259	9.37E-08	MRPL2
4806	221199_at	0.723262963	7.42E-06	GFRA4
4807	236527_at	0.723274074	3.03E-07	ATP6V0E1
4808	213813_x_at	0.725274074	1.58E-06	
4809	215429_s_at	0.72537037	6.97E-08	ZNF428
4810	229986_at	0.725674074	5.47E-07	ZNF717
4811	242969_at	0.726485185	1.10E-06	ZNF780B
4812	215749_s_at	0.726662963	6.52E-09	GORASP1
4813	221504_s_at	0.726737037	6.04E-10	ATP6V1H
4814	244433_at	0.726981481	7.87E-06	
4815	223259_at	0.72717037	2.18E-07	ORMDL3
4816	1555846_a_at	0.727292593	4.57E-09	
4817	202282_at	0.727466667	2.67E-07	HSD17B10
4818	210667_s_at	0.727740741	6.54E-06	C21orf33
4819	223245_at	0.727914815	2.52E-06	STRBP
4820	218908_at	0.728344444	6.43E-07	ASPSCR1
4821	222995_s_at	0.728374074	2.69E-07	RHBDD2
4822	219741_x_at	0.728533333	2.03E-09	ZNF552
4823	33768_at	0.729377778	2.98E-07	DMWD
4824	207124_s_at	0.729481481	4.87E-06	GNB5
4825	225982_at	0.729507407	5.11E-10	UBTF
4826	208745_at	0.729962963	1.60E-07	ATP5L
4827	202255_s_at	0.730022222	6.17E-07	SIPA1L1
4828	209001_s_at	0.730118519	3.34E-07	ANAPC13
4829	212175_s_at	0.730607407	5.52E-07	AK2
4830	229460_at	0.731118519	1.61E-08	FAM126B

4831	227357_at	0.731544444	3.24E-09	TAB3
4832	226586_at	0.73207037	7.14E-08	ANKS6
4833	205633_s_at	0.732614815	1.31E-09	ALAS1
4834	223391_at	0.733159259	2.14E-06	SGPP1
4835	223059_s_at	0.733251852	2.97E-07	FAM107B
4836	236916_at	0.733340741	1.65E-06	
4837	1555916_at	0.734625926	1.18E-10	RPUSD3
4838	230196_x_at	0.735111111	2.97E-06	ARHGAP23
4839	222301_at	0.735262963	7.17E-06	C1orf61
4840	221709_s_at	0.735414815	2.42E-06	ZNF839
4841	219154_at	0.736162963	5.23E-06	TMEM120B
4842	210950_s_at	0.73657037	7.05E-08	FDFT1
4843	218249_at	0.737844444	1.38E-08	ZDHHC6
4844	209002_s_at	0.737925926	1.15E-10	CALCOCO1
4845	226297_at	0.738192593	1.99E-09	HIPK3
4846	212128_s_at	0.738325926	3.64E-08	DAG1
4847	219459_at	0.738481481	4.25E-06	POLR3B
4848	200850_s_at	0.739096296	3.01E-11	AHCYL1
4849	228200_at	0.739818519	6.66E-10	ZNF252P
4850	208297_s_at	0.739903704	8.33E-06	EVI5
4851	38069_at	0.74007037	7.37E-08	CLCN7
4852	218526_s_at	0.740166667	3.83E-10	RANGRF
4853	223515_s_at	0.740459259	3.66E-06	COQ3
4854	203039_s_at	0.740696296	3.03E-07	NDUFS1
4855	201557_at	0.741211111	1.56E-07	VAMP2
4856	228122_at	0.741766667	4.93E-10	CCDC66
4857	225497_at	0.742155556	6.11E-10	ATE1
4858	217622_at	0.742233333	5.90E-06	RHBDD3
4859	208898_at	0.742440741	1.45E-08	ATP6V1D
4860	235195_at	0.743833333	2.86E-07	FBXW2
4861	209100_at	0.743862963	2.26E-06	IFRD2
4862	234111_at	0.743922222	3.33E-08	
4863	208987_s_at	0.744281481	6.58E-06	KDM2A
4864	217377_x_at	0.744403704	6.62E-07	NTRK3
4865	201967_at	0.744881481	1.36E-07	RBM6
4866	236332_at	0.744977778	1.04E-07	DHRS7
4867	202447_at	0.744981481	2.22E-08	DECR1
4868	212425_at	0.745585185	4.67E-07	SCAMP1
4869	212228_s_at	0.746022222	6.31E-07	COQ9
4870	225935_at	0.746314815	7.58E-06	CUX1
4871	228318_s_at	0.746603704	5.81E-06	CRIPAK
4872	219175_s_at	0.746733333	9.29E-08	SLC41A3
4873	225570_at	0.74677037	5.43E-07	SLC41A1

4874	1555735_a_at	0.747511111	1.23E-07	BAP1
4875	1555870_at	0.747840741	2.93E-07	RNF207
4876	209518_at	0.747922222	4.02E-08	SMARCD1
4877	227563_at	0.748166667	4.45E-06	FAM27E2 /// FAM27E3
4878	209375_at	0.748733333	1.17E-09	XPC
4879	226170_at	0.749062963	5.44E-11	EYA3
4880	243615_at	0.749211111	3.23E-07	
4881	212343_at	0.749840741	1.96E-07	YIPF6
4882	242111_at	0.749977778	5.69E-06	METTL3
4883	226956_at	0.750577778	5.04E-09	MTMR3
4884	238536_at	0.750725926	9.82E-07	
4885	222459_at	0.750996296	9.98E-08	AKIRIN1
4886	218641_at	0.751155556	3.34E-06	C11orf95
4887	225651_at	0.751855556	6.67E-07	UBE2E2
4888	208814_at	0.7519	4.23E-07	HSPA4
4889	218494_s_at	0.75202963	2.71E-06	SLC2A4RG
4890	1556464_a_at	0.752055556	1.13E-08	C2orf72
4891	230646_at	0.752185185	3.66E-07	FNDC5
4892	226039_at	0.75232963	3.18E-07	MGAT4A
4893	230656_s_at	0.752603704	5.71E-07	CIRH1A
4894	225346_at	0.752881481	2.04E-06	MTERF2
4895	223008_s_at	0.753703704	7.56E-07	TMEM245
4896	243948_at	0.754192593	1.53E-06	ZC3H14
4897	233252_s_at	0.754266667	6.55E-06	STRBP
4898	218351_at	0.754322222	8.58E-08	COMMD8
4899	1569579_at	0.754388889	4.95E-09	
4900	223649_s_at	0.754440741	1.55E-06	SLC25A39
4901	214923_at	0.754533333	4.34E-06	ATP6V1D
4902	228162_at	0.754937037	1.76E-06	ESD
4903	226892_at	0.754966667	2.40E-07	C10orf12
4904	226703_at	0.755333333	1.63E-06	NEURL4
4905	225112_at	0.755966667	8.29E-08	ABI2
4906	222426_at	0.756325926	1.23E-07	MAPKAP1
4907	213626_at	0.756574074	4.93E-07	CBR4
4908	226859_at	0.756733333	1.73E-08	DNAJC25 /// DNAJC25-GNG10
4909	210870_s_at	0.757085185	3.80E-06	EPM2A
4910	226458_at	0.757103704	3.98E-08	RP1-39G22.7
4911	1554549_a_at	0.757307407	6.89E-06	WDR20
4912	239437_at	0.757440741	3.85E-07	
4913	209119_x_at	0.757496296	2.13E-06	NR2F2
4914	221951_at	0.757825926	4.16E-09	TMEM80
4915	204016_at	0.757988889	4.87E-10	LARS2
4916	242862_x_at	0.758066667	3.46E-08	

4917	230737_s_at	0.758222222	2.73E-07	LOC102724316 /// PTCHD3P1
4918	200903_s_at	0.758703704	1.94E-06	AHCY
4919	226782_at	0.759040741	1.91E-06	SLC25A30
4920	231071_at	0.759392593	3.03E-07	YTHDF3-AS1
4921	218897_at	0.760703704	4.73E-07	TMEM177
4922	239633_at	0.761033333	3.93E-06	
4923	213212_x_at	0.761048148	1.60E-06	GOLGA6L4 /// GOLGA6L5P /// GOLGA6L9 /// LOC102724093
4924	212323_s_at	0.761459259	9.18E-07	VPS13D
4925	201567_s_at	0.761725926	6.26E-12	GOLGA4
4926	212553_at	0.762525926	1.08E-06	RPRD2
4927	226780_s_at	0.762525926	2.20E-07	C7orf55
4928	212731_at	0.762633333	4.92E-07	ANKRD46
4929	225926_at	0.762755556	6.50E-10	VTI1B
4930	202801_at	0.762781481	1.91E-07	PRKACA
4931	239358_at	0.762918519	3.32E-06	
4932	213398_s_at	0.764614815	3.44E-10	SDR39U1
4933	201075_s_at	0.764755556	8.96E-10	SMARCC1
4934	1553397_at	0.765377778	4.61E-06	CCDC13
4935	219205_at	0.765611111	2.52E-08	SRR
4936	242602_x_at	0.7657	2.50E-08	ZNF254
4937	238660_at	0.765737037	1.24E-07	WDFY3
4938	235573_at	0.766174074	3.50E-06	HSPH1
4939	227597_at	0.766722222	7.40E-06	DIS3L2
4940	223196_s_at	0.766814815	3.50E-06	SESN2
4941	225409_at	0.76697037	2.32E-08	COA5
4942	225589_at	0.767277778	1.42E-08	SH3RF1
4943	227945_at	0.767377778	1.35E-09	TBC1D1
4944	218455_at	0.767851852	6.17E-08	NFS1
4945	227932_at	0.76802963	6.06E-10	ARIH2
4946	1555374_at	0.768577778	3.90E-06	TTL
4947	204488_at	0.769751852	8.26E-10	DOLK
4948	213564_x_at	0.769807407	2.86E-11	LDHB
4949	219640_at	0.770514815	1.85E-06	CLDN15
4950	223109_at	0.770714815	8.26E-08	TRUB2
4951	52159_at	0.771703704	5.13E-10	HEMK1
4952	201553_s_at	0.772359259	7.33E-10	LAMP1
4953	239532_at	0.772544444	5.58E-07	RP11-559M23.1
4954	226975_at	0.773003704	1.67E-06	RNPC3
4955	221041_s_at	0.773044444	3.67E-06	SLC17A5
4956	223921_s_at	0.773218519	1.08E-08	GBA2
4957	1566823_a_at	0.77472963	2.75E-06	
4958	202288_at	0.774896296	7.96E-07	MTOR

4959	208950_s_at	0.775792593	1.31E-08	ALDH7A1
4960	201247_at	0.776281481	1.50E-08	SREBF2
4961	209177_at	0.777022222	1.04E-10	NDUFAF3
4962	1555250_a_at	0.777718519	2.30E-06	CPEB3
4963	238467_at	0.777744444	1.89E-07	DYNLL2
4964	212644_s_at	0.777807407	1.53E-10	MAPK1IP1L
4965	232011_s_at	0.777885185	1.52E-08	MAP1LC3A
4966	1558486_at	0.777944444	5.09E-07	ZNF493
4967	237408_at	0.778362963	3.82E-06	DCUN1D1
4968	228084_at	0.778562963	1.07E-06	PLA2G12A
4969	226708_at	0.778818519	9.46E-06	RP11-661A12.9
4970	203272_s_at	0.779137037	7.31E-10	TUSC2
4971	218661_at	0.779874074	6.92E-08	NAA60
4972	220417_s_at	0.780059259	1.23E-07	THAP4
4973	203796_s_at	0.780692593	1.38E-06	BCL7A
4974	204238_s_at	0.781418519	2.04E-06	DNPH1
4975	242092_at	0.781662963	1.27E-06	EPB41L2
4976	228611_s_at	0.782074074	2.21E-08	
4977	208250_s_at	0.782159259	5.75E-06	DMBT1
4978	232053_x_at	0.782840741	2.14E-07	RHBDD2
4979	1557189_at	0.782881481	1.01E-07	DNASE1
4980	201034_at	0.782907407	6.45E-06	ADD3
4981	241933_at	0.783051852	1.72E-07	QRSL1
4982	214170_x_at	0.783485185	9.68E-08	FH
4983	235782_at	0.783555556	4.10E-06	AGO3
4984	227373_at	0.783577778	1.39E-09	ATXN1L
4985	202379_s_at	0.783685185	2.69E-06	NKTR
4986	207300_s_at	0.783933333	1.59E-06	F7
4987	211735_x_at	0.784655556	5.07E-06	SFTPC
4988	1554077_a_at	0.785355556	9.55E-09	TMEM53
4989	221249_s_at	0.7855	2.11E-06	FAM117A
4990	224728_at	0.786207407	2.44E-06	ATPAF1
4991	201919_at	0.786325926	4.05E-09	SLC25A36
4992	220094_s_at	0.787459259	1.08E-09	MCUR1
4993	213551_x_at	0.787511111	1.17E-06	PCGF2
4994	236328_at	0.78862963	3.75E-07	ZNF285 /// ZNF806
4995	239688_at	0.788648148	8.66E-06	SMC1A
4996	1555905_a_at	0.789774074	6.48E-08	TCAIM
4997	233570_at	0.790014815	1.02E-08	
4998	227543_at	0.790622222	3.19E-07	RNASEH2C
4999	1559002_at	0.791133333	1.00E-06	MORF4L2-AS1
5000	206047_at	0.791237037	3.75E-06	GNB3
5001	236580_at	0.791955556	5.37E-06	

5002	205301_s_at	0.792062963	2.25E-07	OGG1
5003	229810_at	0.792218519	9.44E-10	RSPH3
5004	222469_s_at	0.792333333	2.55E-06	TOLLIP
5005	230479_at	0.792462963	6.50E-08	EIF3F
5006	1558345_a_at	0.793588889	4.81E-07	LOC439911
5007	209412_at	0.793837037	1.02E-08	LOC102724200 /// TRAPPC10
5008	1555864_s_at	0.794496296	1.35E-09	PDHA1
5009	215178_x_at	0.794644444	2.02E-06	NAAA
5010	228433_at	0.794944444	1.10E-06	NFYA
5011	234578_at	0.795981481	7.88E-07	
5012	222866_s_at	0.796566667	3.02E-07	FLVCR2
5013	234000_s_at	0.796814815	2.11E-10	PTPLAD1
5014	226097_at	0.797144444	5.85E-07	FNDC5
5015	201410_at	0.798748148	1.08E-09	PLEKHB2
5016	229321_s_at	0.798888889	4.35E-06	DQ570096
5017	211454_x_at	0.799614815	1.25E-08	FKSG49
5018	235635_at	0.799959259	1.28E-06	ARHGAP5
5019	201611_s_at	0.800003704	2.93E-11	ICMT
5020	201729_s_at	0.800048148	2.53E-08	KIAA0100
5021	1553594_a_at	0.800203704	3.07E-06	INSL3
5022	208360_s_at	0.800666667	6.85E-06	ERVH-4
5023	205539_at	0.800774074	3.08E-06	AVIL
5024	203054_s_at	0.800818519	2.91E-07	TCTA
5025	1555845_at	0.800888889	2.61E-06	
5026	217961_at	0.801062963	7.45E-09	SLC25A38
5027	210647_x_at	0.802107407	1.42E-08	PLA2G6
5028	220353_at	0.802244444	9.95E-07	FAM86C1
5029	1569905_at	0.802692593	9.88E-06	HSD11B1L
5030	215210_s_at	0.802881481	3.47E-07	DLST
5031	218942_at	0.8034	4.45E-11	PIP4K2C
5032	241993_x_at	0.803518519	5.35E-06	
5033	224250_s_at	0.803611111	4.10E-09	SECISBP2
5034	238569_at	0.804281481	5.74E-07	GABBR1
5035	200961_at	0.804359259	8.50E-07	SEPHS2
5036	228045_at	0.8045	3.01E-06	SUGT1
5037	208140_s_at	0.805466667	2.71E-06	LRRC48
5038	204985_s_at	0.806048148	1.36E-06	TRAPPC6A
5039	212161_at	0.8062	4.47E-06	AP2A2
5040	225845_at	0.806796296	8.36E-08	ZBTB44
5041	218795_at	0.807114815	7.94E-08	ACP6
5042	225465_at	0.807222222	1.94E-08	MAGI1
5043	1559765_a_at	0.808377778	1.02E-07	LOC101928483
5044	201073_s_at	0.809351852	3.35E-10	SMARCC1

5045	202652_at	0.809492593	1.21E-06	APBB1
5046	218138_at	0.810481481	1.49E-09	MKKS
5047	221088_s_at	0.810955556	9.84E-06	PPP1R9A
5048	1569520_at	0.811148148	1.71E-08	LOC101929747
5049	227348_at	0.811248148	2.86E-09	PARS2
5050	216229_x_at	0.811511111	9.38E-07	
5051	235798_at	0.811577778	7.23E-07	TMEM170B
5052	225628_s_at	0.811992593	6.25E-07	MLLT6
5053	201334_s_at	0.812207407	2.66E-09	ARHGEF12
5054	222280_at	0.812488889	4.31E-06	LOC100506469
5055	215605_at	0.813125926	5.27E-06	NCOA2
5056	1565641_at	0.813459259	3.38E-06	C16orf45
5057	36830_at	0.813937037	2.26E-06	MIPEP
5058	1562228_s_at	0.814211111	7.08E-08	PDE5A
5059	238027_at	0.8146	8.23E-08	SPATA24
5060	219802_at	0.815314815	4.37E-08	PYROXD1
5061	213540_at	0.815407407	8.00E-07	HSD17B8
5062	211984_at	0.815892593	8.71E-09	CALM1 /// CALM2 /// CALM3
5063	209019_s_at	0.815988889	1.74E-07	PINK1
5064	209248_at	0.816014815	2.52E-07	GHITM
5065	202026_at	0.8161	8.01E-08	SDHD
5066	208912_s_at	0.816462963	1.43E-06	CNP
5067	205420_at	0.816666667	2.18E-06	PEX7
5068	242978_x_at	0.816937037	1.32E-07	
5069	1555063_at	0.817311111	2.65E-07	USP32 /// USP6
5070	212326_at	0.817588889	1.57E-07	VPS13D
5071	218078_s_at	0.81762963	7.08E-12	ZDHHC3
5072	213244_at	0.817725926	2.81E-09	SCAMP4
5073	236196_at	0.818388889	4.04E-06	ZNF326
5074	232183_at	0.819092593	1.21E-07	SERAC1
5075	201612_at	0.819433333	3.48E-08	ALDH9A1
5076	208741_at	0.819748148	2.25E-06	SAP18
5077	232366_at	0.819877778	4.88E-07	KIAA0232
5078	223103_at	0.820077778	2.57E-06	STARD10
5079	215054_at	0.820351852	1.38E-06	EPOR /// RGL3
5080	232975_at	0.822492593	1.78E-08	HCG18
5081	217693_x_at	0.823603704	6.39E-06	MAGOH2
5082	225704_at	0.824248148	1.25E-08	FBRSL1
5083	213046_at	0.825007407	2.27E-08	PABPN1
5084	216218_s_at	0.825503704	7.94E-06	PLCL2
5085	203179_at	0.825711111	7.00E-09	GALT
5086	202942_at	0.825714815	1.65E-08	ETFB
5087	225020_at	0.825840741	3.60E-07	DAB2IP

5088	238574_at	0.826062963	1.81E-08	SLC25A51
5089	212775_at	0.826433333	4.06E-06	OBSL1
5090	205201_at	0.826944444	3.65E-07	GLI3
5091	213323_s_at	0.828588889	1.35E-08	ZC3H7B
5092	207069_s_at	0.829766667	4.33E-08	SMAD6
5093	202586_at	0.829914815	2.85E-06	POLR2L
5094	213935_at	0.830166667	9.02E-09	ABHD5
5095	229595_at	0.830211111	2.17E-11	CHCHD4
5096	227466_at	0.830640741	2.22E-11	FAM200B
5097	217016_x_at	0.831637037	3.27E-06	TMEM212
5098	225522_at	0.831662963	3.34E-08	AAK1
5099	212928_at	0.831718519	4.15E-09	TSPYL4
5100	206037_at	0.832151852	1.61E-07	CCBL1
5101	209075_s_at	0.8323	2.01E-09	ISCU
5102	229238_at	0.832433333	1.88E-06	C17orf97
5103	205600_x_at	0.833403704	2.03E-08	HOXB5
5104	205773_at	0.833511111	5.71E-07	CPEB3
5105	219462_at	0.834262963	9.84E-09	TMEM53
5106	229001_at	0.834751852	1.41E-06	PPP1R3E
5107	227559_at	0.834914815	1.88E-07	NDUFAF4
5108	224732_at	0.8358	8.61E-13	CHTF8
5109	226204_at	0.835940741	6.48E-09	C22orf29 /// GNB1L
5110	209513_s_at	0.836218519	3.54E-07	HSDL2
5111	213275_x_at	0.836281481	1.04E-07	CTSB
5112	225598_at	0.836288889	5.44E-08	SLC45A4
5113	200708_at	0.837122222	4.77E-08	GOT2
5114	243309_at	0.837192593	1.66E-07	C15orf65
5115	209726_at	0.837225926	6.68E-06	CA11
5116	203589_s_at	0.837866667	5.15E-06	TFDP2
5117	209262_s_at	0.838303704	5.33E-07	NR2F6
5118	226065_at	0.838933333	8.06E-06	PRICKLE1
5119	235365_at	0.839918519	7.91E-07	DFNB59
5120	202299_s_at	0.840374074	1.43E-09	LAMTOR5
5121	227080_at	0.840396296	1.72E-06	ZNF697
5122	240257_at	0.840511111	1.22E-06	SYNJ2
5123	226123_at	0.841766667	1.63E-06	CHD7
5124	234278_at	0.842577778	5.77E-06	
5125	223554_s_at	0.842914815	4.72E-07	RANGRF
5126	226412_at	0.842922222	1.31E-08	PNISR
5127	236167_at	0.843425926	8.77E-06	
5128	210976_s_at	0.845144444	1.65E-07	PFKM
5129	223178_s_at	0.8453	1.74E-06	NT5DC1
5130	243966_at	0.845385185	3.75E-07	

5131	201591_s_at	0.845588889	4.26E-08	NISCH
5132	225590_at	0.845618519	2.73E-07	SH3RF1
5133	225298_at	0.845655556	2.02E-09	PNKD
5134	218760_at	0.8463	1.47E-10	COQ6
5135	206868_at	0.846344444	3.15E-08	STARD8
5136	226564_at	0.847025926	1.48E-07	ZFAT
5137	202452_at	0.847303704	1.12E-09	ZER1
5138	1555858_at	0.847318519	1.31E-07	THUMPD3-AS1
5139	230001_at	0.84767037	1.78E-07	9-Mar
5140	205530_at	0.847725926	1.05E-06	ETFDH
5141	224186_s_at	0.848362963	1.98E-09	RNF123
5142	202468_s_at	0.849133333	5.66E-06	CTNNAL1
5143	222481_at	0.8498	7.82E-10	TIMM10B
5144	242931_at	0.849851852	5.73E-06	
5145	240205_x_at	0.849888889	6.92E-06	
5146	235133_at	0.850225926	6.19E-07	ZDHHC14
5147	240478_at	0.8509	9.88E-06	
5148	201753_s_at	0.851848148	3.95E-06	ADD3
5149	217806_s_at	0.85232963	9.81E-09	POLDIP2
5150	224903_at	0.852896296	9.13E-08	CIRH1A
5151	1552274_at	0.852955556	1.02E-07	PXK
5152	203588_s_at	0.853059259	2.45E-06	TFDP2
5153	213851_at	0.853918519	8.71E-07	TMEM110
5154	213234_at	0.854144444	2.69E-07	KIAA1467
5155	238762_at	0.854148148	1.26E-07	MTHFD2L
5156	1563138_at	0.854159259	1.93E-06	
5157	209390_at	0.854292593	5.61E-10	TSC1
5158	213052_at	0.854514815	3.47E-14	PRKAR2A
5159	1554542_at	0.855244444	1.61E-06	SLC25A48
5160	225795_at	0.855744444	2.54E-07	SMDT1
5161	213388_at	0.855907407	3.18E-06	LOC100996724 /// LOC100996761 /// LOC101929792 /// LOC101930416 /// PDE4DIP
5162	231222_at	0.856033333	8.55E-08	
5163	230259_at	0.856366667	7.58E-06	FUOM
5164	232527_at	0.856522222	8.97E-06	PSMD6-AS2
5165	216301_at	0.857444444	2.95E-06	
5166	217893_s_at	0.857559259	4.06E-10	AKIRIN1
5167	213158_at	0.8576	8.49E-08	ZBTB20
5168	230211_at	0.858411111	4.70E-06	TRIP11
5169	202825_at	0.85862963	8.29E-06	SLC25A4
5170	234981_x_at	0.858944444	1.15E-06	CMBL
5171	1564498_at	0.859266667	1.17E-07	
5172	209003_at	0.85997037	1.17E-07	SLC25A11

5173	228718_at	0.860722222	8.65E-08	ZNF44
5174	242071_x_at	0.860844444	4.04E-07	ITGA8
5175	218026_at	0.861233333	1.00E-10	COA3
5176	200895_s_at	0.861396296	9.19E-09	FKBP4
5177	214386_at	0.861674074	3.04E-06	
5178	235864_at	0.861937037	4.89E-08	ATP6V1D
5179	238056_at	0.862107407	9.07E-09	SDHC
5180	208532_x_at	0.862866667	8.70E-06	KRTAP5-8
5181	209964_s_at	0.862955556	4.86E-09	ATXN7
5182	209839_at	0.863777778	7.92E-06	DNM3
5183	204703_at	0.863866667	1.42E-06	IFT88
5184	226476_s_at	0.8639	2.11E-07	VPRBP
5185	242065_x_at	0.864785185	6.96E-09	IDI1
5186	201834_at	0.864837037	5.45E-06	PRKAB1
5187	224514_x_at	0.865037037	7.61E-08	IL17RC
5188	229253_at	0.865281481	8.94E-10	THEM4
5189	1554628_at	0.865881481	4.95E-09	ZNF57
5190	216389_s_at	0.866944444	7.29E-10	DCAF11
5191	225286_at	0.86757037	4.78E-06	ARSD
5192	213499_at	0.867603704	1.22E-06	CLCN2
5193	216060_s_at	0.867937037	4.42E-06	DAAM1
5194	216582_at	0.867981481	3.39E-08	POM121L2
5195	226820_at	0.868203704	7.19E-10	ZNF362
5196	213648_at	0.8688	3.20E-11	EXOSC7
5197	201957_at	0.868885185	2.04E-07	PPP1R12B
5198	204872_at	0.869048148	1.92E-06	TLE4
5199	227423_at	0.869185185	1.21E-10	LRRC28
5200	208914_at	0.87157037	4.27E-08	GGA2
5201	239283_at	0.871981481	1.02E-06	TMED5
5202	223338_s_at	0.872418519	4.53E-09	ATPIF1
5203	212876_at	0.87342963	4.12E-06	B4GALT4
5204	225968_at	0.873837037	1.47E-07	PRICKLE2
5205	202387_at	0.874055556	1.73E-09	BAG1
5206	1553775_at	0.874966667	8.56E-07	FLJ31715
5207	1563497_at	0.875177778	7.90E-06	USP25
5208	231936_at	0.875337037	3.47E-08	HOXC9
5209	243318_at	0.875481481	3.59E-06	DCAF8
5210	211774_s_at	0.875666667	1.35E-08	MMACHC
5211	213666_at	0.875833333	1.75E-06	6-Sep
5212	1559991_s_at	0.876148148	5.58E-07	
5213	213034_at	0.876518519	1.07E-11	SIK3
5214	227686_at	0.876840741	2.72E-10	OXNAD1
5215	225330_at	0.877622222	1.92E-07	IGF1R

5216	204921_at	0.878440741	1.05E-07	GAS8
5217	213724_s_at	0.878551852	3.10E-06	PDK2
5218	242535_at	0.879544444	1.49E-06	
5219	228380_at	0.880140741	1.23E-08	SENP2
5220	205011_at	0.880292593	2.53E-06	VWA5A
5221	235013_at	0.880577778	1.75E-06	SLC31A1
5222	241596_at	0.880748148	3.01E-07	NUDT10
5223	1552935_at	0.881581481	4.37E-06	ZCCHC5
5224	216467_s_at	0.881622222	3.61E-07	
5225	220072_at	0.88172963	5.68E-07	CSPP1
5226	204497_at	0.882062963	1.14E-08	ADCY9
5227	236248_x_at	0.88227037	3.32E-08	TADA2B
5228	226687_at	0.883259259	4.70E-08	PRPF40A
5229	219321_at	0.88437037	3.01E-06	MPP5
5230	204157_s_at	0.884392593	1.92E-08	SIK3
5231	231897_at	0.884981481	1.06E-06	PTGR1
5232	226019_at	0.885448148	4.62E-10	OMA1
5233	215543_s_at	0.885488889	6.30E-06	LARGE
5234	204360_s_at	0.886374074	1.43E-08	NAGLU
5235	243074_at	0.88642963	1.35E-10	
5236	1555860_x_at	0.886603704	3.49E-09	THUMPD3-AS1
5237	209213_at	0.886881481	4.29E-06	CBR1
5238	208807_s_at	0.886974074	2.05E-06	CHD3
5239	202366_at	0.887311111	8.86E-06	ACADS
5240	225961_at	0.887592593	3.26E-08	KLHL42
5241	222752_s_at	0.888088889	6.06E-09	TMEM206
5242	222193_at	0.888481481	1.11E-08	C2orf43
5243	209494_s_at	0.888811111	1.02E-09	PATZ1
5244	203632_s_at	0.888837037	9.75E-06	GPRC5B
5245	222405_at	0.889196296	8.85E-12	PTPLAD1
5246	202128_at	0.889255556	5.66E-11	AREL1
5247	209769_s_at	0.889685185	1.46E-06	SEPT5-GP1BB
5248	1558702_at	0.889992593	3.06E-08	TEX10
5249	215038_s_at	0.89037037	2.81E-11	SETD2
5250	235466_s_at	0.891151852	1.37E-09	LOC102724017
5251	236908_at	0.891637037	5.10E-06	
5252	218966_at	0.892114815	2.69E-06	MYO5C
5253	1552615_at	0.892214815	2.56E-10	ACACB
5254	206167_s_at	0.892944444	4.59E-07	ARHGAP6
5255	223195_s_at	0.892966667	1.89E-06	SESN2
5256	213204_at	0.893262963	2.17E-08	CUL9
5257	221913_at	0.893503704	3.84E-07	SIRT3
5258	235728_at	0.893577778	6.96E-07	ZFP3

5259	61874_at	0.893725926	6.03E-12	CACFD1
5260	211693_at	0.89387037	4.92E-07	IGH
5261	227005_at	0.893907407	1.62E-12	RPP14
5262	204511_at	0.894959259	3.07E-06	FARP2
5263	205752_s_at	0.895114815	1.57E-07	GSTM5
5264	242297_at	0.89512963	7.30E-06	RREB1
5265	1569516_at	0.895407407	6.44E-06	
5266	221227_x_at	0.895914815	6.82E-10	COQ3
5267	222531_s_at	0.89592963	2.72E-08	AP5M1
5268	236605_at	0.896177778	5.17E-07	EIF3K
5269	226239_at	0.896318519	7.13E-08	TMEM150A
5270	228450_at	0.896377778	3.40E-06	PLEKHA7
5271	213156_at	0.89652963	1.58E-07	ZBTB20
5272	210407_at	0.896662963	4.85E-09	PPM1A
5273	1487_at	0.897540741	7.14E-09	ESRRA
5274	202893_at	0.897774074	7.55E-07	UNC13B
5275	225882_at	0.897911111	1.31E-08	SLC35B4
5276	235826_at	0.898044444	3.40E-06	LOC101928954
5277	200849_s_at	0.898425926	6.69E-11	AHCYL1
5278	222394_at	0.898662963	2.37E-10	PDCD6IP
5279	222809_x_at	0.898948148	2.64E-06	CCDC85C
5280	228257_at	0.899107407	1.64E-06	ANKRD52
5281	226790_at	0.899340741	1.00E-10	MORN2
5282	228494_at	0.899696296	2.64E-06	PPP1R9A
5283	233168_s_at	0.899940741	3.80E-09	SELO
5284	217773_s_at	0.900107407	1.78E-11	NDUFA4
5285	222006_at	0.900174074	6.26E-10	LETM1
5286	226344_at	0.900188889	3.75E-06	ZMAT1
5287	220183_s_at	0.900955556	8.42E-08	NUDT6
5288	232341_x_at	0.902711111	4.08E-06	HABP4
5289	225000_at	0.903140741	2.48E-15	PRKAR2A
5290	39817_s_at	0.903407407	6.00E-09	DNPH1
5291	211475_s_at	0.903414815	1.48E-08	BAG1
5292	235788_at	0.903455556	2.57E-06	HCG18
5293	228120_at	0.903492593	1.59E-10	AGO1
5294	235962_at	0.903948148	2.17E-10	AZI2
5295	224288_x_at	0.904181481	1.39E-09	FKSG49
5296	225946_at	0.904322222	3.22E-08	RASSF8
5297	235772_at	0.904666667	5.30E-08	PPP3CB-AS1
5298	1563475_s_at	0.905033333	5.15E-07	METTL20
5299	213140_s_at	0.905274074	2.89E-11	SS18L1
5300	201030_x_at	0.9053	4.16E-13	LDHB
5301	212503_s_at	0.905303704	3.40E-06	DIP2C

5302	236356_at	0.906818519	8.36E-06	NDUFS1
5303	242743_at	0.906859259	8.23E-08	
5304	229066_at	0.907159259	6.54E-10	
5305	242229_at	0.907374074	2.53E-07	NAPEPLD
5306	225531_at	0.907518519	1.85E-06	CABLES1
5307	241226_at	0.907581481	2.89E-06	
5308	220642_x_at	0.90822963	2.87E-09	GPR89A /// GPR89B
5309	213279_at	0.908559259	1.94E-07	DHRS1
5310	213353_at	0.908740741	4.02E-06	ABCA5
5311	221620_s_at	0.908840741	1.09E-10	APOO
5312	240615_at	0.910377778	4.71E-06	PTOV1-AS1
5313	224622_at	0.910692593	5.22E-11	TBC1D14
5314	226201_at	0.910907407	2.86E-07	DOT1L
5315	218212_s_at	0.911103704	1.23E-09	MOCS2
5316	227121_at	0.911314815	1.29E-08	ZBTB20
5317	205434_s_at	0.911440741	2.34E-07	AAK1
5318	229535_at	0.912137037	1.36E-13	PTPMT1
5319	210290_at	0.912425926	3.25E-07	ZNF174
5320	1554154_at	0.91267037	1.97E-07	GDAP2
5321	234937_x_at	0.912944444	3.42E-06	ZFP28
5322	238053_at	0.912951852	9.86E-08	DHRSX
5323	217619_x_at	0.913107407	5.40E-07	
5324	212830_at	0.913155556	1.48E-08	MEGF9
5325	204608_at	0.913833333	1.90E-06	ASL
5326	238918_at	0.914177778	8.09E-06	
5327	203933_at	0.914281481	7.60E-08	RAB11FIP3
5328	221556_at	0.914340741	8.41E-06	CDC14B
5329	218658_s_at	0.91462963	4.65E-11	ACTR8
5330	201752_s_at	0.915037037	3.02E-06	ADD3
5331	238058_at	0.915159259	3.98E-08	PRR34-AS1
5332	218540_at	0.915577778	7.20E-09	THTPA
5333	202959_at	0.91562963	2.96E-10	MUT
5334	233417_at	0.915885185	7.98E-06	
5335	226318_at	0.916496296	7.03E-08	TBRG1
5336	243964_at	0.916655556	6.67E-06	
5337	238789_at	0.91687037	7.97E-09	KANK1
5338	243138_at	0.91702963	1.87E-07	
5339	223150_s_at	0.917085185	3.30E-10	PTPN23
5340	239031_at	0.917114815	2.14E-06	SSTR2
5341	217794_at	0.917611111	9.21E-09	PRR13
5342	225997_at	0.918140741	2.13E-11	MOB1B
5343	225494_at	0.919407407	5.24E-11	DYNLL2

5344	214864_s_at	0.919748148	1.71E-07	GRHPR
5345	236001_at	0.919837037	1.01E-07	LINC00675
5346	218722_s_at	0.920088889	1.57E-10	CCDC51
5347	206710_s_at	0.92032963	7.07E-06	EPB41L3
5348	212823_s_at	0.920914815	6.91E-06	PLEKHG3
5349	226331_at	0.921603704	4.38E-10	BBX
5350	227770_at	0.922540741	8.32E-09	COG8
5351	227977_at	0.922777778	5.92E-09	ZADH2
5352	90265_at	0.922885185	1.13E-09	ADAP1
5353	219723_x_at	0.923025926	7.94E-07	AGPAT3
5354	244301_at	0.924188889	5.89E-06	
5355	208242_at	0.926337037	1.19E-06	RAX
5356	235483_at	0.92717037	4.78E-06	STX3
5357	227082_at	0.928174074	1.02E-07	ZBTB20
5358	243036_at	0.928185185	3.58E-07	CCDC30
5359	228090_at	0.928807407	1.82E-08	NMNAT3
5360	230714_s_at	0.929218519	6.18E-06	
5361	215269_at	0.93	5.35E-06	TRAPPC10
5362	239499_at	0.930125926	4.64E-06	DNAH2
5363	204263_s_at	0.930803704	6.12E-07	CPT2
5364	226356_at	0.931248148	9.08E-08	FAM73B
5365	1570511_at	0.931640741	7.89E-06	ARHGEF10L
5366	216698_x_at	0.931688889	3.83E-08	OR7E12P
5367	225217_s_at	0.932525926	8.52E-11	BRPF3
5368	201032_at	0.933281481	3.37E-11	BLCAP
5369	235043_at	0.934881481	4.30E-07	FAM122A
5370	241074_at	0.934951852	1.94E-06	
5371	228904_at	0.935488889	4.10E-06	HOXB3
5372	201536_at	0.935492593	3.93E-09	DUSP3
5373	225150_s_at	0.93582963	9.49E-07	RTKN
5374	229829_at	0.936381481	5.01E-09	LINC00526
5375	214277_at	0.936792593	8.99E-07	COX11
5376	217845_x_at	0.936944444	6.31E-11	HIGD1A
5377	225377_at	0.937003704	3.09E-10	RABL6
5378	203723_at	0.938188889	7.68E-07	ITPKB
5379	227988_s_at	0.938744444	1.09E-06	VPS13A
5380	210201_x_at	0.938814815	2.75E-07	BIN1
5381	77508_r_at	0.940207407	3.16E-11	RABEP2
5382	237706_at	0.940281481	6.52E-07	STXBP4
5383	203408_s_at	0.940388889	1.79E-08	SATB1
5384	232702_at	0.940992593	1.04E-08	RABGAP1L
5385	243760_at	0.941062963	1.58E-07	MIPEPP3
5386	1555886_at	0.941151852	3.09E-10	PDSS2

5387	215186_at	0.942133333	1.45E-06	TBC1D30
5388	214835_s_at	0.942266667	2.33E-10	SUCLG2
5389	217939_s_at	0.942451852	1.95E-09	AFTPH
5390	212445_s_at	0.942466667	6.98E-09	NEDD4L
5391	204573_at	0.943111111	3.62E-10	CROT
5392	239602_at	0.943237037	1.46E-07	BRWD1-IT2
5393	233177_s_at	0.943344444	3.78E-08	PNKD
5394	223132_s_at	0.943525926	1.10E-11	TRIM8
5395	226717_at	0.943792593	6.77E-08	LRTOMT
5396	227547_at	0.944692593	1.73E-10	RP5-1085F17.3
5397	203500_at	0.944848148	1.62E-10	GCDH
5398	202587_s_at	0.944877778	4.65E-08	AK1
5399	235024_at	0.945325926	2.81E-09	JADE1
5400	203387_s_at	0.945925926	1.37E-06	TBC1D4
5401	229054_at	0.947744444	3.03E-08	
5402	237494_at	0.947981481	3.84E-08	RP11-548M13.1
5403	225923_at	0.948214815	1.94E-06	VAPB
5404	215253_s_at	0.949018519	4.98E-06	RCAN1
5405	207408_at	0.949251852	1.48E-07	SLC22A14
5406	221069_s_at	0.949711111	2.21E-10	TACO1
5407	222651_s_at	0.950485185	1.72E-09	TRPS1
5408	1557081_at	0.950674074	6.30E-07	RBM25
5409	204440_at	0.951077778	2.38E-06	CD83
5410	240616_at	0.951866667	2.46E-10	
5411	229366_at	0.952451852	1.24E-09	RP11-97C16.1
5412	204156_at	0.952618519	4.37E-09	SIK3
5413	223165_s_at	0.9527	3.38E-10	IP6K2
5414	236358_at	0.9531	1.05E-10	
5415	242370_at	0.953977778	1.78E-06	MTHFD2L
5416	223087_at	0.954166667	5.46E-12	ECHDC1
5417	202564_x_at	0.954548148	2.13E-11	ARL2
5418	219307_at	0.954655556	1.19E-11	PDSS2
5419	206303_s_at	0.954677778	3.09E-07	NUDT4 /// NUDT4P1 /// NUDT4P2
5420	244687_at	0.955718519	3.25E-10	DBT
5421	236311_at	0.95577037	7.27E-08	LOH12CR2
5422	224784_at	0.95587037	1.35E-10	MLLT6
5423	203682_s_at	0.956148148	3.39E-09	IVD
5424	224627_at	0.956311111	2.10E-09	GBA2
5425	208911_s_at	0.956455556	2.26E-10	PDHB
5426	202289_s_at	0.956985185	7.42E-07	TACC2
5427	51774_s_at	0.959144444	2.54E-11	UBE2D4
5428	1555790_a_at	0.959477778	1.01E-07	TMEM192 /// ZNF320
5429	215374_at	0.959696296	2.63E-06	PAPOLA

5430	221760_at	0.959937037	4.48E-06	MAN1A1
5431	200661_at	0.960144444	2.06E-07	CTSA
5432	239070_at	0.960381481	8.77E-06	
5433	204287_at	0.960625926	3.76E-06	SYNGR1
5434	229573_at	0.960662963	6.88E-10	USP9X
5435	238199_x_at	0.960911111	7.59E-10	COX3 /// LOC101929612
5436	228458_at	0.961240741	1.82E-11	C6orf226
5437	215479_at	0.96147037	9.84E-06	
5438	1560817_at	0.961559259	1.48E-08	
5439	240398_at	0.961962963	3.48E-07	ITPKB-IT1
5440	1563707_at	0.962344444	6.08E-06	AL831948 /// RP1-197B17.3
5441	244020_at	0.963640741	1.31E-06	
5442	220107_s_at	0.9639	9.09E-08	ZC2HC1C
5443	235112_at	0.96397037	1.05E-07	
5444	217367_s_at	0.964522222	1.26E-08	ZHX3
5445	225930_at	0.966081481	2.18E-10	NKIRAS1
5446	228429_x_at	0.966225926	1.88E-08	KIF9
5447	230871_at	0.966733333	3.06E-10	DHX30
5448	218471_s_at	0.966762963	3.62E-10	BBS1
5449	228983_at	0.966911111	2.40E-08	C17orf85
5450	225689_at	0.967225926	2.20E-12	POMGNT2
5451	218548_x_at	0.967785185	7.13E-12	TEX264
5452	218980_at	0.967848148	4.01E-06	FHOD3
5453	1555982_at	0.968044444	7.01E-07	ZFYVE16
5454	218019_s_at	0.968977778	9.69E-07	PDXK
5455	223006_s_at	0.969962963	2.45E-08	TMEM245
5456	201282_at	0.970781481	1.91E-08	OGDH
5457	225587_at	0.970918519	6.51E-09	TMEM129
5458	228381_at	0.971592593	5.24E-10	ATF7IP2
5459	242932_at	0.971651852	1.30E-06	
5460	225796_at	0.972359259	5.70E-09	PXK
5461	202660_at	0.972611111	1.81E-08	ITPR2
5462	243618_s_at	0.972755556	4.88E-08	ZNF827
5463	1554582_a_at	0.972792593	8.07E-07	METTL20
5464	240015_at	0.972955556	2.10E-06	PRKCQ-AS1
5465	224500_s_at	0.97297037	2.44E-09	MON1A
5466	229384_at	0.973048148	2.20E-11	CTC-429P9.3
5467	203966_s_at	0.974062963	5.27E-09	PPM1A
5468	240165_at	0.974514815	5.66E-06	
5469	242877_at	0.976074074	3.59E-06	
5470	224871_at	0.976155556	5.96E-10	TPRG1L
5471	202931_x_at	0.976807407	2.61E-07	BIN1
5472	221717_at	0.97777037	3.41E-07	

5473	231952_at	0.977892593	1.88E-06	
5474	228216_at	0.978118519	2.82E-11	ZBTB37
5475	231199_at	0.978140741	4.31E-06	RP11-271C24.3
5476	242705_x_at	0.97852963	7.80E-07	LRPAP1
5477	221974_at	0.979140741	1.10E-07	IPW /// LOC101930404 /// PWARSN /// SNORD107 /// SNORD115-13 /// SNORD115-26 /// SNORD115-7 /// SNORD116-22 /// SNORD116-28 /// SNORD116-4 /// SNRPN
5478	232376_at	0.979374074	1.72E-07	PCCA
5479	236769_at	0.97982963	3.08E-06	LOC158402
5480	207309_at	0.980322222	1.78E-06	NOS1
5481	225358_at	0.981192593	3.28E-08	DNAJC19
5482	230320_at	0.98132963	4.42E-12	TBRG1
5483	235211_at	0.982240741	3.48E-09	
5484	212652_s_at	0.982537037	1.93E-09	SNX4
5485	216230_x_at	0.983044444	1.18E-06	SMPD1
5486	215930_s_at	0.983274074	6.00E-08	CTAGE5
5487	218342_s_at	0.984792593	4.37E-08	ERMP1
5488	225922_at	0.985133333	1.13E-08	FNIP2
5489	209361_s_at	0.985785185	9.68E-11	PCBP4
5490	200659_s_at	0.986074074	6.73E-08	PHB
5491	244193_at	0.986092593	2.19E-08	DNAJC22
5492	210480_s_at	0.986418519	3.38E-06	MYO6
5493	242264_at	0.986818519	1.13E-06	
5494	224869_s_at	0.986966667	6.67E-13	MRPS25
5495	215247_at	0.987692593	8.58E-07	LOC100288570
5496	242800_at	0.987777778	1.59E-07	NHS
5497	212757_s_at	0.987903704	5.40E-10	CAMK2G
5498	214070_s_at	0.98802963	2.20E-06	ATP10B
5499	227433_at	0.988451852	2.35E-12	KIAA2018
5500	1554475_a_at	0.989062963	2.03E-07	C19orf47
5501	238554_at	0.98987037	4.02E-10	CYB5B
5502	202772_at	0.990411111	4.59E-09	HMGCL
5503	205166_at	0.990885185	3.97E-06	CAPN5
5504	222025_s_at	0.991233333	9.22E-08	OPLAH
5505	234206_at	0.991807407	3.64E-06	
5506	211023_at	0.992592593	1.41E-14	PDHB
5507	212410_at	0.993114815	2.61E-12	MICU2
5508	221575_at	0.995081481	4.24E-07	SCLY
5509	226252_at	0.995244444	2.48E-08	ZBTB20
5510	212383_at	0.995548148	9.28E-09	ATP6V0A1
5511	231943_at	0.995677778	1.05E-06	ZFP28
5512	226849_at	0.996518519	5.85E-10	DENND1A

5513	1556048_at	0.996659259	1.30E-08	LOC100507564
5514	218476_at	0.997381481	2.42E-11	POMT1
5515	1556580_a_at	0.997974074	9.44E-06	RP11-981G7.6
5516	1557114_a_at	0.998603704	3.09E-11	C19orf82
5517	226726_at	0.999007407	1.22E-08	MBOAT2
5518	229744_at	0.999196296	5.12E-08	SSFA2
5519	206846_s_at	1.000092593	1.74E-08	HDAC6
5520	210202_s_at	1.000288889	1.87E-06	BIN1
5521	213129_s_at	1.000396296	1.30E-08	GCSH /// LOC101060817
5522	238485_at	1.0004	4.86E-06	IQCH-AS1
5523	241472_at	1.000914815	1.60E-06	DMXL1
5524	202871_at	1.001048148	8.57E-09	TRAF4
5525	226848_at	1.001081481	9.13E-08	
5526	233489_at	1.001203704	2.38E-06	TMEM43
5527	203647_s_at	1.0013	5.57E-09	FDX1
5528	1554671_a_at	1.002344444	6.05E-07	SRRM2
5529	222598_s_at	1.002837037	3.75E-07	NAV2
5530	227701_at	1.002877778	1.38E-08	CCDC186
5531	227120_at	1.004337037	5.37E-07	FOXP4
5532	209531_at	1.004785185	1.86E-08	GSTZ1
5533	203020_at	1.004877778	3.07E-13	RABGAP1L
5534	38918_at	1.005159259	3.74E-10	SOX13
5535	200914_x_at	1.005388889	3.93E-12	KTN1
5536	230701_x_at	1.005518519	1.29E-06	KIF9
5537	243933_at	1.006996296	6.51E-06	
5538	204072_s_at	1.007466667	9.28E-06	FRY
5539	228557_at	1.0075	6.86E-06	L3MBTL4
5540	231868_at	1.007988889	3.90E-07	HOMEZ
5541	222140_s_at	1.00807037	5.62E-09	GPR89A /// GPR89B
5542	201074_at	1.008240741	1.62E-10	SMARCC1
5543	211177_s_at	1.008403704	1.28E-09	TXNRD2
5544	243372_at	1.010244444	1.52E-06	HSPD1
5545	210515_at	1.010348148	1.94E-06	HNF1A
5546	229515_at	1.01037037	1.40E-09	PAWR
5547	227009_at	1.010588889	3.48E-10	LOC100507431
5548	233221_at	1.010655556	2.15E-06	DEFB118
5549	215322_at	1.010911111	1.61E-06	LONRF1
5550	230792_at	1.011262963	6.04E-06	FAAH2
5551	211776_s_at	1.011507407	2.09E-07	EPB41L3
5552	1553693_s_at	1.011544444	2.80E-07	CBR4
5553	43427_at	1.012622222	2.21E-08	ACACB
5554	211941_s_at	1.01352963	3.05E-11	PEBP1
5555	213306_at	1.013862963	4.09E-08	MPDZ

5556	231110_at	1.013988889	9.46E-06	
5557	244779_at	1.014355556	3.30E-06	ZDHHC2
5558	1566157_x_at	1.014840741	6.51E-06	
5559	217761_at	1.014892593	2.05E-06	ADI1
5560	209818_s_at	1.015033333	5.59E-10	HABP4
5561	34697_at	1.015088889	9.91E-11	LRP6
5562	229694_at	1.015248148	7.03E-06	WDR11
5563	226602_s_at	1.015655556	2.17E-07	BCR
5564	227899_at	1.015659259	3.06E-06	VIT
5565	234321_x_at	1.015777778	3.21E-10	NHSL1
5566	215848_at	1.016325926	2.46E-07	SCAPER
5567	236514_at	1.016362963	3.50E-08	ACOT8
5568	225463_x_at	1.0167	4.50E-10	GPR89A /// GPR89B
5569	232493_at	1.017055556	1.86E-10	
5570	221979_at	1.017833333	3.29E-08	TOPORS-AS1
5571	204625_s_at	1.018625926	5.05E-06	ITGB3
5572	211992_at	1.018781481	4.35E-10	WNK1
5573	242123_at	1.021211111	5.90E-09	PAQR7
5574	242356_at	1.021377778	7.10E-11	VTI1A
5575	217187_at	1.022403704	3.92E-07	MUC5AC
5576	230740_at	1.02262963	3.11E-07	EHD3
5577	49679_s_at	1.022811111	4.26E-10	MMP24-AS1
5578	229500_at	1.023562963	2.81E-08	SLC30A9
5579	214148_at	1.024166667	2.65E-06	ITFG2 /// LOC100507424
5580	231190_at	1.024296296	8.26E-06	POM121L10P
5581	219573_at	1.024407407	3.30E-12	LRRC16A
5582	59697_at	1.025085185	1.01E-07	RAB15
5583	226013_at	1.025107407	9.06E-10	TRAK1
5584	221214_s_at	1.025162963	1.37E-06	MIR7114 /// NSMF
5585	227252_at	1.025366667	8.30E-09	LRP10
5586	205882_x_at	1.025574074	4.76E-07	ADD3
5587	230006_s_at	1.025766667	2.42E-07	SVIP
5588	217293_at	1.0262	1.09E-06	
5589	225794_s_at	1.026251852	1.84E-10	SMDT1
5590	235630_at	1.026392593	5.27E-10	
5591	236473_at	1.027581481	5.23E-07	CC2D2A
5592	235531_at	1.027744444	1.41E-07	RP11-884K10.7
5593	1560689_s_at	1.027748148	4.75E-06	AKT2
5594	206870_at	1.027874074	4.16E-07	PPARA
5595	228349_at	1.027892593	2.83E-08	KIAA1958
5596	237680_at	1.029277778	3.29E-06	LOC102723542
5597	213133_s_at	1.02972963	4.42E-08	GCSH /// LOC101060817
5598	203116_s_at	1.030096296	1.66E-11	FECH

5599	228312_at	1.030211111	3.04E-06	PI16
5600	219077_s_at	1.032714815	1.80E-09	WWOX
5601	234941_s_at	1.032911111	2.66E-09	GPHN
5602	201432_at	1.033392593	2.50E-09	CAT
5603	244220_at	1.033722222	3.32E-08	
5604	1562256_at	1.033748148	1.52E-08	NLRP1
5605	226590_at	1.033951852	1.21E-08	ZNF618
5606	211918_x_at	1.034151852	4.48E-06	PAPPA2
5607	239268_at	1.034151852	6.83E-06	NDUFS1
5608	225747_at	1.034255556	3.52E-09	COQ10A
5609	224135_at	1.034637037	2.31E-07	WDR87
5610	237476_at	1.034644444	3.81E-06	
5611	224015_s_at	1.035066667	3.90E-09	MRPS25
5612	228568_at	1.035225926	5.72E-07	MYZAP
5613	202960_s_at	1.035744444	6.23E-14	MUT
5614	202850_at	1.035885185	4.96E-10	ABCD3
5615	226020_s_at	1.036107407	8.34E-09	DAB1 /// OMA1
5616	215440_s_at	1.036114815	1.89E-06	BEX4
5617	204547_at	1.036525926	2.16E-07	RAB40B
5618	219327_s_at	1.036655556	2.10E-06	GPRC5C
5619	238573_at	1.036896296	9.10E-08	OTUD7B
5620	239396_at	1.03707037	8.82E-06	
5621	235433_at	1.037407407	7.79E-12	APOOL
5622	232500_at	1.037507407	6.38E-07	RALGAPA2
5623	228480_at	1.038366667	2.52E-09	VAPA
5624	202178_at	1.040237037	4.23E-09	PRKCZ
5625	241344_at	1.040540741	3.84E-14	
5626	207921_x_at	1.040762963	3.35E-06	PAX8
5627	213228_at	1.041696296	2.05E-06	PDE8B
5628	214106_s_at	1.041755556	5.63E-09	GMDS
5629	1553030_a_at	1.041844444	5.68E-11	SUOX
5630	219259_at	1.042074074	2.90E-06	SEMA4A
5631	1554003_at	1.042503704	4.92E-06	ARHGEF28
5632	235667_at	1.042977778	3.18E-06	USP46-AS1
5633	217734_s_at	1.04312963	1.58E-10	WDR6
5634	207323_s_at	1.043303704	2.66E-09	MBP
5635	204979_s_at	1.0443	4.17E-10	SH3BGR
5636	239857_at	1.044611111	8.73E-08	
5637	1562639_at	1.044622222	4.62E-06	KIF6
5638	218935_at	1.044762963	3.52E-06	EHD3
5639	213215_at	1.044818519	6.29E-08	AP3S2 /// C15orf38-AP3S2
5640	36475_at	1.045981481	3.34E-08	GCAT
5641	203826_s_at	1.046362963	6.14E-11	PITPNM1

5642	209794_at	1.046422222	1.47E-06	SRGAP3
5643	216347_s_at	1.046840741	7.38E-10	PPP1R13B
5644	229278_at	1.048181481	1.05E-07	USP51
5645	62987_r_at	1.048377778	2.18E-07	CACNG4
5646	222496_s_at	1.048655556	1.09E-07	RBM47
5647	225237_s_at	1.049266667	3.07E-07	MSI2
5648	214523_at	1.049525926	9.45E-06	CEBPE
5649	231846_at	1.049707407	6.07E-07	FOXRED2
5650	201886_at	1.05	9.05E-13	DCAF11
5651	215246_at	1.050288889	1.31E-07	LARP7
5652	211497_x_at	1.050711111	5.16E-07	NKX3-1
5653	230987_at	1.050722222	1.35E-06	
5654	237107_at	1.051362963	4.39E-07	PRKRA
5655	223387_at	1.05162963	2.03E-14	ZFYVE1
5656	239082_at	1.051862963	1.98E-09	FZD3
5657	202292_x_at	1.051907407	1.06E-13	LYPLA2
5658	48031_r_at	1.052048148	4.61E-07	FAXDC2
5659	224655_at	1.052118519	1.69E-11	AK3
5660	1557586_s_at	1.052285185	5.63E-06	ATP6V1H
5661	212602_at	1.052414815	2.16E-09	WDFY3
5662	204604_at	1.052462963	1.75E-08	CDK14
5663	213240_s_at	1.053548148	9.31E-06	KRT4
5664	237619_at	1.054096296	2.99E-08	FAM217A
5665	235984_at	1.05412963	5.89E-07	
5666	235092_at	1.054148148	6.53E-06	
5667	241718_x_at	1.054155556	1.30E-06	
5668	215712_s_at	1.054774074	2.27E-07	IGFALS
5669	230762_at	1.054918519	2.46E-06	
5670	207162_s_at	1.055225926	2.62E-06	CACNA1B
5671	237218_at	1.055533333	1.86E-06	
5672	205288_at	1.055892593	1.91E-07	CDC14A
5673	217495_x_at	1.056307407	1.60E-07	CALCA
5674	214073_at	1.056422222	7.86E-08	CTTN
5675	218139_s_at	1.056444444	1.68E-12	AP5M1
5676	226460_at	1.057311111	2.57E-08	FNIP2
5677	232773_at	1.057422222	7.27E-08	
5678	220141_at	1.05752963	9.28E-06	C11orf63
5679	235428_at	1.057551852	3.53E-07	LOC100507316
5680	203433_at	1.058081481	3.07E-09	MTHFS /// ST20-MTHFS
5681	220702_at	1.058433333	4.96E-06	
5682	243630_at	1.05857037	1.08E-09	NDUFB1
5683	225804_at	1.059544444	3.24E-10	CYB5D2
5684	231861_at	1.060862963	1.21E-10	LRP10

5685	202590_s_at	1.061366667	8.55E-08	PDK2
5686	240508_at	1.061940741	9.54E-07	
5687	205601_s_at	1.062533333	2.02E-09	HOXB5
5688	230375_at	1.063051852	7.04E-06	PNISR
5689	231695_at	1.063292593	1.15E-07	
5690	203110_at	1.063540741	3.77E-07	PTK2B
5691	242300_at	1.063551852	1.78E-09	
5692	215119_at	1.063611111	5.55E-08	MYO16
5693	203628_at	1.063822222	2.13E-07	IGF1R
5694	1557338_x_at	1.063966667	2.96E-10	SUCLG2-AS1
5695	209740_s_at	1.064711111	4.25E-06	PNPLA4
5696	201708_s_at	1.064725926	1.17E-08	NIPSNAP1
5697	201599_at	1.065007407	5.02E-10	OAT
5698	218910_at	1.065088889	2.93E-12	ANO10
5699	203792_x_at	1.065114815	1.68E-09	PCGF2
5700	213591_at	1.065492593	1.14E-06	ALDH7A1
5701	230285_at	1.065892593	2.96E-09	SVIP
5702	219076_s_at	1.066762963	5.06E-06	PXMP2
5703	227582_at	1.067218519	5.64E-06	KLHDC9
5704	206818_s_at	1.067555556	6.52E-11	CNNM2
5705	222236_s_at	1.067703704	1.37E-07	ASAP3
5706	1552275_s_at	1.067962963	8.03E-06	PXK
5707	243992_at	1.068181481	5.33E-07	
5708	217040_x_at	1.068262963	9.27E-09	SOX15
5709	216028_at	1.068348148	6.15E-15	
5710	222134_at	1.06907037	2.42E-08	DDO
5711	223531_x_at	1.069211111	9.91E-11	GPR89A /// GPR89B
5712	218440_at	1.069425926	6.96E-09	MCCC1
5713	201910_at	1.069459259	4.74E-09	FARP1
5714	203271_s_at	1.06962963	8.93E-06	UNC119
5715	204824_at	1.06962963	7.34E-07	ENDOG
5716	233350_s_at	1.070474074	4.67E-14	TEX264
5717	201972_at	1.071614815	3.43E-11	ATP6V1A
5718	204167_at	1.071614815	1.26E-12	BTD
5719	228159_at	1.0725	7.98E-13	WDFY2
5720	225240_s_at	1.073637037	3.48E-10	MSI2
5721	205371_s_at	1.0738	8.68E-10	DBT
5722	218633_x_at	1.074322222	3.26E-10	ABHD10
5723	239611_at	1.074781481	4.67E-06	
5724	233912_x_at	1.075044444	2.34E-08	
5725	1555993_at	1.075859259	2.68E-08	CACNA1D
5726	222400_s_at	1.076192593	4.37E-08	ADI1
5727	233539_at	1.076825926	9.24E-08	NAPEPLD

5728	234694_at	1.076907407	9.01E-06	CNTROB
5729	1555446_s_at	1.077403704	3.23E-06	TRAPPC10
5730	220584_at	1.077614815	8.56E-08	FLJ22184
5731	226171_at	1.077892593	7.99E-13	ZDHHC3
5732	239562_at	1.078040741	1.59E-07	MTHFD2L
5733	200924_s_at	1.078162963	4.96E-07	SLC3A2
5734	227435_at	1.078251852	3.42E-12	KIAA2018
5735	213321_at	1.078751852	7.55E-06	BCKDHB
5736	228391_at	1.079781481	1.57E-06	CYP4V2
5737	233974_s_at	1.081103704	4.65E-09	FAM129B
5738	227576_at	1.081151852	6.92E-06	
5739	203216_s_at	1.0812	3.81E-08	MYO6
5740	241741_at	1.082114815	3.67E-07	CRLS1
5741	235321_at	1.082581481	1.11E-07	NDUFS1
5742	234753_x_at	1.082722222	6.50E-06	
5743	232218_at	1.083385185	1.48E-11	RP11-339B21.15
5744	209600_s_at	1.083755556	1.74E-10	ACOX1
5745	232455_x_at	1.085355556	7.95E-10	LOC340085
5746	229323_at	1.085562963	9.57E-10	LINC00959
5747	1569111_at	1.085744444	1.07E-06	SOX13
5748	239528_at	1.086144444	9.70E-09	PROM2
5749	230715_at	1.086159259	1.77E-06	ZNF518B
5750	216903_s_at	1.086222222	9.65E-11	MICU1
5751	206709_x_at	1.086318519	2.03E-09	GPT
5752	200965_s_at	1.086551852	3.49E-08	ABLIM1
5753	223405_at	1.086622222	1.91E-06	NPL
5754	227987_at	1.086648148	7.19E-08	VPS13A
5755	243431_at	1.087325926	3.87E-07	
5756	242362_at	1.08787037	3.98E-07	
5757	223964_x_at	1.088285185	1.17E-14	
5758	1556129_at	1.088481481	5.66E-09	LOC642533
5759	238619_at	1.088555556	3.43E-06	RP11-138A9.1
5760	216051_x_at	1.089466667	2.23E-07	
5761	225924_at	1.089648148	4.52E-09	FNIP2
5762	244341_at	1.090540741	3.90E-06	
5763	209286_at	1.090614815	2.35E-06	CDC42EP3
5764	213713_s_at	1.09067037	6.89E-06	GLB1L2
5765	226722_at	1.09077037	6.80E-06	FAM20C
5766	201347_x_at	1.090948148	2.04E-08	GRHPR
5767	213556_at	1.091777778	1.35E-08	PINLYP
5768	232169_x_at	1.092207407	3.06E-10	MIR4691 /// MIR7113 /// NDUFS8
5769	235652_at	1.093151852	2.69E-06	
5770	224254_x_at	1.09342963	5.83E-06	

5771	229826_at	1.093566667	1.67E-10	SMIM4
5772	215792_s_at	1.094074074	1.13E-11	DNAJC11
5773	212229_s_at	1.094933333	5.22E-11	FBXO21
5774	229962_at	1.095151852	2.72E-12	LRRC37A3
5775	212614_at	1.095855556	6.36E-08	ARID5B
5776	238191_at	1.096366667	4.81E-11	SDHC
5777	212181_s_at	1.097207407	2.45E-10	NUDT4 /// NUDT4P1 /// NUDT4P2
5778	238806_at	1.097566667	2.74E-08	
5779	226494_at	1.097655556	6.84E-06	CAMSAP3
5780	236855_at	1.097774074	1.34E-09	C10orf85
5781	40837_at	1.098103704	9.52E-07	TLE2
5782	221953_s_at	1.0997	8.04E-08	MMP24-AS1
5783	227702_at	1.099948148	6.77E-06	CYP4X1
5784	238040_at	1.100155556	6.40E-06	
5785	217645_at	1.100240741	4.37E-12	COX16 /// SYNJ2BP-COX16
5786	214117_s_at	1.100244444	5.98E-10	BTD
5787	241595_at	1.100381481	2.81E-06	
5788	222731_at	1.100481481	7.74E-06	ZDHHC2
5789	224391_s_at	1.100511111	2.67E-08	SIAE
5790	226688_at	1.1007	4.94E-15	TCAIM
5791	204224_s_at	1.10092963	7.53E-08	GCH1
5792	240168_at	1.101018519	2.58E-06	XPO7
5793	211423_s_at	1.10132963	1.80E-09	SC5D
5794	230791_at	1.101703704	1.88E-06	
5795	223366_at	1.101855556	9.47E-06	ZNF704
5796	1559075_s_at	1.101859259	6.28E-06	BAHCC1
5797	235990_at	1.102066667	4.74E-09	LOC100130987
5798	212231_at	1.1021	6.07E-11	FBXO21
5799	206417_at	1.102355556	2.29E-06	CNGA1
5800	201522_x_at	1.102411111	9.09E-10	SNRPN /// SNURF
5801	206993_at	1.102911111	5.45E-11	ATP5S
5802	239474_at	1.103462963	5.82E-06	
5803	201906_s_at	1.103603704	2.28E-09	CTDSPL
5804	220538_at	1.104440741	5.64E-06	ADM2
5805	217337_at	1.104577778	5.60E-09	RP1-101G11.2
5806	241490_s_at	1.104951852	1.98E-06	PGBD2
5807	221966_at	1.106655556	1.85E-06	GPR137
5808	208903_at	1.107677778	2.65E-07	RPS28
5809	227781_x_at	1.108244444	4.29E-06	FAM57B /// LOC100996332
5810	225263_at	1.108333333	5.10E-10	HS6ST1
5811	201709_s_at	1.108985185	1.67E-09	NIPSNAP1
5812	213546_at	1.109122222	2.00E-07	DKFZP586I1420
5813	242310_at	1.109151852	9.67E-07	

5814	219194_at	1.109555556	2.01E-06	SEMA4G
5815	209623_at	1.109681481	3.11E-09	MCCC2
5816	203488_at	1.110918519	4.64E-06	LPHN1
5817	214332_s_at	1.1126	2.38E-06	TSFM
5818	204201_s_at	1.112618519	5.25E-08	PTPN13
5819	235974_at	1.112925926	1.09E-07	EXOC4
5820	226461_at	1.113118519	1.01E-08	HOXB9
5821	242851_at	1.114648148	9.84E-06	KIAA1919
5822	229848_at	1.114703704	7.78E-07	ZNF10
5823	237092_at	1.115774074	6.63E-07	
5824	212456_at	1.115966667	8.99E-10	CLUH
5825	234032_at	1.116203704	4.96E-06	
5826	229444_at	1.116214815	5.66E-10	LOC101929243
5827	206554_x_at	1.11632963	7.36E-14	SETMAR
5828	211994_at	1.116896296	1.18E-13	WNK1
5829	205933_at	1.117240741	1.38E-08	SETBP1
5830	219114_at	1.117396296	5.96E-07	C3orf18
5831	209681_at	1.118259259	1.90E-10	SLC19A2
5832	206229_x_at	1.119437037	5.50E-08	PAX2
5833	242108_at	1.120596296	2.28E-07	
5834	214241_at	1.120951852	7.79E-08	NDUFB8
5835	202250_s_at	1.122285185	1.25E-11	DCAF8
5836	205050_s_at	1.1223	2.11E-08	MAPK8IP2
5837	203870_at	1.122314815	8.28E-12	USP46
5838	221063_x_at	1.1224	9.57E-14	RNF123
5839	209502_s_at	1.122492593	9.96E-06	BAIAP2
5840	224828_at	1.122692593	9.68E-08	CPEB4
5841	238172_at	1.1234	8.00E-09	
5842	221004_s_at	1.123907407	3.41E-06	ITM2C
5843	224558_s_at	1.126040741	1.09E-08	MALAT1
5844	232096_x_at	1.127351852	1.12E-07	FOXP1-IT1
5845	244047_at	1.127577778	8.78E-06	
5846	211922_s_at	1.127981481	8.06E-09	CAT
5847	231254_at	1.128885185	1.24E-06	
5848	225211_at	1.128918519	9.11E-10	PVRL1
5849	206128_at	1.12892963	2.39E-07	ADRA2C
5850	231089_at	1.129222222	9.09E-06	LOC100505664
5851	236703_at	1.1311	3.64E-07	
5852	223266_at	1.131296296	1.22E-10	STRADB
5853	227627_at	1.131344444	2.96E-09	C8orf44-SGK3 /// SGK3
5854	213198_at	1.131566667	3.57E-12	ACVR1B
5855	207776_s_at	1.131977778	3.54E-06	CACNB2
5856	209512_at	1.132592593	2.31E-08	HSDL2

5857	244546_at	1.132840741	4.31E-08	CYCS
5858	204977_at	1.133174074	1.00E-07	DDX10
5859	231959_at	1.134185185	1.61E-10	LIN52
5860	1555653_at	1.134733333	1.30E-09	
5861	208951_at	1.135185185	3.68E-11	ALDH7A1
5862	236826_at	1.135833333	5.41E-08	TTC39B
5863	205058_at	1.136792593	3.51E-08	SLC26A1
5864	242346_x_at	1.139107407	2.51E-07	
5865	218417_s_at	1.139277778	1.22E-12	SLC48A1
5866	228528_at	1.139744444	8.51E-07	MIR29B2 /// MIR29C
5867	224812_at	1.140048148	3.05E-08	HIBADH
5868	1554050_at	1.140174074	4.31E-08	SMPDL3B
5869	1556595_at	1.140292593	2.68E-06	RP11-319G9.3
5870	229084_at	1.140714815	5.92E-08	CNTN4
5871	221879_at	1.140888889	5.22E-06	CALML4
5872	227679_at	1.141214815	1.24E-12	HDAC11
5873	243249_at	1.141325926	5.16E-08	
5874	1562719_at	1.141492593	3.77E-10	LOC101928476
5875	1554161_at	1.1423	1.89E-06	SLC25A27
5876	65585_at	1.143351852	3.38E-11	FAM86B1 /// FAM86B2 /// FAM86C1 /// FAM86DP /// FAM86FP
5877	215772_x_at	1.143485185	3.02E-12	SUCLG2
5878	235722_at	1.143659259	3.73E-14	SYNJ2BP /// SYNJ2BP-COX16
5879	201072_s_at	1.14367037	2.79E-14	SMARCC1
5880	205125_at	1.143822222	1.01E-15	PLCD1
5881	208322_s_at	1.143911111	5.24E-08	ST3GAL1
5882	206256_at	1.144074074	3.33E-06	CPN1
5883	235010_at	1.144262963	1.00E-09	ZBED5-AS1
5884	236217_at	1.145114815	2.06E-10	SLC31A1
5885	238093_at	1.145614815	4.79E-08	LOC100129722
5886	224707_at	1.145796296	6.58E-12	CYSTM1
5887	207006_s_at	1.145892593	2.12E-06	CCDC106
5888	232161_x_at	1.146077778	2.42E-06	PTPN4
5889	226506_at	1.146751852	2.99E-08	THSD4
5890	242099_at	1.146851852	7.93E-06	
5891	229867_at	1.147388889	3.75E-10	BTBD9
5892	204811_s_at	1.147403704	1.60E-07	CACNA2D2
5893	233021_at	1.147425926	9.16E-08	RBM26-AS1
5894	229338_at	1.147840741	5.49E-10	LOC100289361
5895	235053_at	1.147966667	5.85E-10	
5896	219150_s_at	1.148344444	1.20E-08	ADAP1
5897	241458_at	1.149285185	3.54E-07	
5898	224151_s_at	1.149648148	1.88E-11	AK3

5899	238624_at	1.150044444	2.49E-10	LOC102724517 /// NLK
5900	233369_at	1.1503	4.46E-08	
5901	224415_s_at	1.150518519	1.45E-10	HINT2
5902	1557645_at	1.150662963	5.40E-06	
5903	240241_at	1.150777778	1.89E-06	
5904	242657_at	1.151274074	3.88E-11	
5905	34764_at	1.15217037	1.12E-16	LARS2
5906	231276_at	1.152181481	7.62E-06	
5907	232037_at	1.152188889	7.78E-07	IGDCC3
5908	228572_at	1.152959259	2.43E-06	GRB2
5909	212459_x_at	1.153181481	4.48E-12	SUCLG2
5910	238207_at	1.153737037	5.08E-08	SMIM4
5911	241466_at	1.153818519	1.81E-07	
5912	244674_at	1.15387037	5.62E-06	
5913	215755_at	1.154177778	2.49E-06	RN7SKP150
5914	234650_at	1.154325926	1.35E-06	
5915	225436_at	1.154518519	5.85E-10	ABHD17C
5916	237116_at	1.156611111	3.09E-07	LOC646903
5917	202204_s_at	1.157107407	3.03E-14	AMFR
5918	202700_s_at	1.157166667	2.92E-10	TMEM63A
5919	232977_x_at	1.157174074	3.71E-06	MYH14
5920	213447_at	1.157577778	5.61E-11	IPW /// LOC101930404 /// PWARSN /// SNORD107 /// SNORD115-13 /// SNORD115-26 /// SNORD115-7 /// SNORD116-22 /// SNORD116-28 /// SNORD116-4 /// SNRPN
5921	213501_at	1.157840741	6.29E-11	ACOX1
5922	243528_at	1.158625926	2.44E-10	
5923	244398_x_at	1.158737037	5.28E-08	ZNF684
5924	212329_at	1.159722222	1.91E-14	SCAP
5925	219986_s_at	1.160592593	2.05E-11	ACAD10
5926	216308_x_at	1.160725926	3.44E-09	GRHPR
5927	1560001_at	1.160759259	1.00E-07	LOC100131581
5928	224712_x_at	1.161196296	2.14E-09	SMIM7
5929	244046_at	1.161933333	6.92E-06	URGCP
5930	230446_at	1.16232963	7.29E-06	LOC101927811
5931	226592_at	1.163444444	2.38E-08	ZNF618
5932	221896_s_at	1.163692593	2.38E-10	HIGD1A
5933	244374_at	1.164485185	2.78E-06	TINCR
5934	224873_s_at	1.165085185	1.86E-12	MRPS25
5935	217223_s_at	1.166611111	1.01E-09	BCR
5936	232890_at	1.166962963	3.24E-07	
5937	218921_at	1.168240741	7.19E-08	SIGIRR
5938	203264_s_at	1.168318519	1.39E-11	ARHGEF9

5939	216143_at	1.168407407	1.08E-09	
5940	238589_s_at	1.168448148	9.88E-07	
5941	226829_at	1.168711111	9.30E-08	AFAP1L2
5942	224882_at	1.168914815	3.17E-08	ACSS1
5943	230393_at	1.169733333	4.39E-10	CUL5
5944	1554522_at	1.170611111	8.80E-08	CNNM2
5945	213622_at	1.171011111	1.13E-10	COL9A2
5946	203425_s_at	1.171974074	1.00E-06	IGFBP5
5947	212771_at	1.172392593	9.09E-07	FAM171A1
5948	212699_at	1.172507407	5.27E-11	SCAMP5
5949	227455_at	1.174974074	7.06E-15	C6orf136
5950	222603_at	1.17522963	4.68E-10	ERMP1
5951	1569847_at	1.176403704	1.16E-06	CGNL1 /// LOC101930344 /// LOC101930349
5952	222403_at	1.176411111	4.97E-11	MTCH2
5953	224140_at	1.176537037	8.94E-06	NPCDR1
5954	220282_at	1.177881481	3.48E-06	RIC3
5955	219946_x_at	1.178237037	8.54E-07	MYH14
5956	205606_at	1.17832963	2.79E-09	LRP6
5957	207486_x_at	1.17837037	5.14E-08	CHN2
5958	241890_at	1.178874074	3.04E-06	
5959	1561589_a_at	1.178892593	2.07E-07	NBEAL1
5960	234963_s_at	1.179018519	1.81E-07	FA2H
5961	220357_s_at	1.179381481	3.75E-09	SGK2
5962	225306_s_at	1.180822222	5.22E-11	SLC25A29
5963	227482_at	1.180981481	2.02E-10	ADCK1
5964	238885_at	1.181174074	3.75E-09	KIAA1549
5965	242103_at	1.182033333	1.84E-10	TMEM86A
5966	1559754_at	1.182222222	2.97E-06	LTB
5967	204867_at	1.182574074	3.44E-06	GCHFR
5968	242956_at	1.182711111	5.91E-06	IDH1
5969	233435_at	1.182740741	9.14E-08	
5970	226249_at	1.184511111	3.59E-07	SNX30
5971	221555_x_at	1.185155556	1.76E-09	CDC14B
5972	227570_at	1.185885185	6.80E-08	TMEM86A
5973	213758_at	1.186077778	1.21E-06	COX4I1
5974	230409_at	1.187988889	2.18E-08	MAGI3
5975	218552_at	1.189292593	2.31E-08	ECHDC2
5976	212736_at	1.189455556	3.75E-13	C16orf45
5977	214696_at	1.190437037	6.77E-08	MIR22 /// MIR22HG
5978	213309_at	1.190507407	8.89E-10	PLCL2
5979	208022_s_at	1.191103704	2.59E-10	CDC14B
5980	202071_at	1.191159259	1.58E-08	SDC4
5981	236742_at	1.1915	2.18E-06	

5982	235822_at	1.191825926	3.34E-09	COQ9
5983	205847_at	1.192122222	2.27E-10	PRSS22
5984	223184_s_at	1.192292593	5.23E-12	AGPAT3
5985	241954_at	1.192659259	6.62E-06	FDFT1
5986	209447_at	1.192792593	7.02E-09	SYNE1
5987	226360_at	1.193066667	2.11E-09	ZNRF3
5988	241735_at	1.193077778	1.13E-07	RASSF8
5989	226010_at	1.193125926	5.50E-08	SLC25A23
5990	227847_at	1.193211111	5.17E-15	EPM2AIP1
5991	219616_at	1.193537037	1.08E-08	ACSS3
5992	1559471_s_at	1.194155556	6.10E-06	D21S2088E
5993	239250_at	1.194418519	5.55E-06	ZNF542P
5994	244511_at	1.194992593	1.45E-06	
5995	231024_at	1.195374074	1.07E-09	LOC101929886 /// PGM5-AS1
5996	229180_at	1.195581481	8.21E-06	WWC1
5997	237202_at	1.195603704	6.68E-06	PGPEP1
5998	204155_s_at	1.197603704	8.46E-16	SIK3
5999	201903_at	1.198144444	1.68E-14	UQCRC1
6000	244646_at	1.198351852	3.45E-07	
6001	228891_at	1.198625926	4.18E-07	SEMA4D
6002	236363_at	1.199637037	7.18E-09	LSM3
6003	1565886_at	1.200266667	2.77E-07	
6004	206059_at	1.200748148	2.04E-13	ZNF91
6005	243153_at	1.201162963	8.79E-06	CDK5RAP2
6006	237190_at	1.201566667	1.23E-10	BTD
6007	229498_at	1.202292593	5.33E-06	MBNL3
6008	227959_at	1.202344444	2.40E-07	ANKRD9
6009	219013_at	1.203133333	2.61E-08	GALNT11
6010	204793_at	1.203481481	7.03E-06	GPRASP1
6011	235796_at	1.203818519	1.39E-09	
6012	230329_s_at	1.204088889	1.74E-13	NUDT6
6013	212951_at	1.204262963	3.24E-06	GPR116
6014	242245_at	1.205114815	6.51E-06	
6015	227451_s_at	1.205292593	2.22E-12	MCUR1
6016	204231_s_at	1.205488889	2.79E-06	FAAH
6017	208416_s_at	1.206137037	6.56E-06	SPTB
6018	214734_at	1.206185185	2.48E-06	EXPH5
6019	203402_at	1.206540741	6.66E-07	KCNAB2
6020	206576_s_at	1.208733333	4.08E-10	CEACAM1
6021	232921_at	1.209025926	1.70E-06	KIAA1549
6022	1561502_x_at	1.209611111	2.91E-07	CTU2
6023	218501_at	1.210244444	2.69E-10	ARHGEF3
6024	226636_at	1.210877778	1.57E-08	PLD1

6025	216791_at	1.211014815	1.02E-07	
6026	244456_at	1.211140741	3.21E-07	
6027	220987_s_at	1.2112	1.07E-06	AKIP1 /// NUAK2
6028	204503_at	1.211433333	1.56E-06	EVPL
6029	242131_at	1.211837037	1.97E-09	ATP6
6030	1559806_at	1.211840741	2.37E-07	RP1-155D22.2
6031	32091_at	1.212318519	7.00E-12	SLC25A44
6032	212683_at	1.212555556	1.19E-13	SLC25A44
6033	223182_s_at	1.213514815	5.69E-09	AGPAT3
6034	220944_at	1.213537037	1.73E-06	PGLYRP4
6035	240032_at	1.213848148	8.16E-06	RP11-318A15.2
6036	244358_at	1.214107407	2.04E-06	
6037	202982_s_at	1.214977778	5.06E-09	ACOT1 /// ACOT2
6038	1559759_at	1.215140741	5.47E-06	KIFC3
6039	202217_at	1.215222222	1.10E-09	C21orf33
6040	242810_x_at	1.216125926	3.52E-06	LOC101930026
6041	219632_s_at	1.216251852	1.14E-09	SHPK /// TRPV1
6042	242518_at	1.218137037	4.04E-10	RAD51-AS1
6043	240111_at	1.218222222	3.96E-07	RHOBTB3
6044	229545_at	1.218455556	9.59E-11	FERMT1
6045	225305_at	1.21867037	2.70E-12	SLC25A29
6046	217977_at	1.219814815	3.17E-08	MSRB1
6047	203658_at	1.2206	1.85E-12	SLC25A20
6048	210825_s_at	1.221222222	1.55E-10	PEBP1
6049	214762_at	1.221622222	6.72E-09	ATP6V1G2
6050	219991_at	1.221722222	5.75E-06	SLC2A9
6051	238116_at	1.221748148	3.75E-06	DYNLRB2
6052	221932_s_at	1.222196296	2.53E-14	GLRX5
6053	205079_s_at	1.222792593	2.23E-11	MPDZ
6054	242885_at	1.224381481	8.80E-09	
6055	211959_at	1.224837037	1.01E-06	IGFBP5
6056	227467_at	1.224888889	6.68E-09	RDH10
6057	236725_at	1.224911111	2.68E-07	WWC1
6058	228950_s_at	1.225196296	3.88E-09	WLS
6059	212336_at	1.226096296	6.90E-10	EPB41L1
6060	212838_at	1.227477778	4.58E-12	DNMBP
6061	244062_at	1.228192593	1.18E-06	DAAM1
6062	202002_at	1.228437037	6.64E-08	ACAA2
6063	215577_at	1.228918519	3.22E-08	
6064	239086_at	1.231248148	1.37E-08	IDNK
6065	242437_at	1.231503704	8.83E-11	
6066	213288_at	1.231511111	2.49E-10	MBOAT2
6067	49452_at	1.232166667	3.91E-09	ACACB

6068	241516_at	1.232959259	3.93E-06	
6069	236147_at	1.233251852	9.24E-07	
6070	226380_at	1.233255556	3.29E-11	PTPN21
6071	209874_x_at	1.233366667	5.75E-10	CNNM2
6072	242809_at	1.233696296	3.70E-07	IL1RL1
6073	215015_at	1.2337	2.05E-06	CCDC64
6074	203115_at	1.234833333	1.78E-08	FECH
6075	239764_at	1.235048148	9.06E-08	ITPR1-AS1
6076	203680_at	1.235096296	6.49E-06	PRKAR2B
6077	227632_at	1.235122222	3.01E-09	TBC1D24
6078	238722_x_at	1.235681481	1.35E-09	NAPEPLD
6079	211695_x_at	1.236325926	1.26E-07	MUC1
6080	201284_s_at	1.236537037	5.46E-15	APEH
6081	235061_at	1.236844444	7.67E-09	PPM1K
6082	209710_at	1.237118519	3.20E-09	GATA2
6083	241703_at	1.237862963	3.00E-07	RUNDC3B
6084	223681_s_at	1.2379	8.45E-06	INADL
6085	220977_x_at	1.238044444	9.44E-08	EPB41L5
6086	235706_at	1.238677778	4.42E-06	CPM
6087	216647_at	1.238840741	7.83E-08	TCF3
6088	231457_at	1.239207407	6.68E-08	
6089	1558412_at	1.239637037	7.75E-14	LOC113230 /// MIR1199
6090	201015_s_at	1.23972963	1.15E-08	JUP
6091	229849_at	1.239762963	1.85E-07	WIPF3
6092	241020_at	1.24037037	9.54E-07	
6093	1558740_s_at	1.240462963	4.56E-07	
6094	243302_at	1.241151852	6.92E-06	RP11-250B2.6
6095	224370_s_at	1.24227037	1.08E-08	CAPS2
6096	235693_at	1.244040741	1.02E-06	
6097	1555617_x_at	1.244781481	1.63E-06	AC010524.4
6098	215390_at	1.244981481	1.45E-06	
6099	212350_at	1.245388889	1.13E-16	TBC1D1
6100	203747_at	1.246074074	3.91E-07	AQP3
6101	205993_s_at	1.246314815	7.05E-07	TBX2
6102	227917_at	1.246766667	1.99E-06	LOC100506990
6103	243579_at	1.247548148	3.36E-12	MSI2
6104	231919_at	1.24837037	1.65E-11	DBT
6105	240188_at	1.248585185	3.11E-07	
6106	228876_at	1.248651852	8.82E-07	BAIAP2L2
6107	229840_at	1.248651852	1.91E-06	IQSEC2
6108	239653_at	1.248925926	3.54E-08	
6109	239597_at	1.249774074	8.05E-07	
6110	226305_at	1.249922222	1.40E-07	LYNX1

6111	236317_at	1.250055556	2.80E-11	
6112	225864_at	1.251848148	9.95E-07	FAM84B
6113	222570_at	1.253166667	5.41E-07	NCS1
6114	209426_s_at	1.253385185	5.84E-06	AMACR /// C1QTNF3-AMACR
6115	242688_at	1.25427037	9.94E-06	
6116	231906_at	1.255144444	3.22E-09	HOXD8
6117	232656_at	1.255162963	3.87E-08	
6118	238751_at	1.255225926	7.69E-07	
6119	202792_s_at	1.255244444	5.10E-10	PPP6R2
6120	219891_at	1.255277778	3.28E-11	PGPEP1
6121	243841_at	1.255488889	6.48E-08	SYNE2
6122	226470_at	1.25552963	8.84E-08	GGT7
6123	241071_at	1.256803704	1.04E-07	
6124	227240_at	1.257318519	3.43E-06	NGEF
6125	230039_at	1.257762963	8.71E-06	
6126	244176_at	1.25787037	3.93E-06	LOC401913
6127	211248_s_at	1.258125926	4.55E-09	CHRD
6128	226589_at	1.258851852	2.34E-12	TMEM192
6129	224829_at	1.258874074	4.87E-11	CPEB4
6130	222365_at	1.258918519	1.05E-06	TBCEL
6131	236610_at	1.259781481	6.92E-06	
6132	210807_s_at	1.260488889	2.70E-08	SLC16A7
6133	232052_at	1.261	9.23E-12	THUMPD3-AS1
6134	220751_s_at	1.262222222	4.50E-06	FAXDC2
6135	240574_at	1.263881481	4.38E-07	DNAJC3-AS1
6136	207547_s_at	1.264148148	6.30E-07	FAM107A
6137	206353_at	1.2648	2.36E-09	COX6A2
6138	216848_at	1.265181481	7.25E-07	
6139	1569834_at	1.265318519	2.48E-07	RP11-65L19.4
6140	38340_at	1.265644444	6.00E-13	HIP1R
6141	244011_at	1.265833333	3.67E-09	PPM1K
6142	242454_at	1.266596296	3.86E-09	
6143	230182_at	1.266777778	1.05E-06	PDCD7
6144	219572_at	1.267955556	6.14E-10	CADPS2
6145	230647_at	1.268059259	1.99E-06	LOC102723990 /// TMEM53
6146	224005_at	1.268374074	2.59E-07	
6147	202315_s_at	1.268533333	1.60E-12	BCR
6148	242945_at	1.268844444	7.45E-07	FAM20A
6149	212319_at	1.270285185	1.60E-06	SGSM2
6150	204565_at	1.27037037	3.74E-12	ACOT13
6151	241489_at	1.270448148	8.43E-06	
6152	202053_s_at	1.270514815	9.59E-08	ALDH3A2
6153	1557232_at	1.271192593	5.57E-09	LOC102725017

6154	234626_at	1.271255556	7.98E-06	OR51I1
6155	224432_at	1.271374074	3.56E-07	SH3GLB2
6156	231319_x_at	1.271685185	1.00E-12	KIF9
6157	207984_s_at	1.272751852	3.61E-07	MPP2
6158	47069_at	1.273140741	2.80E-11	PRR5
6159	1564463_at	1.273507407	3.17E-06	KLF13 /// LOC100499221
6160	208813_at	1.27417037	1.02E-08	GOT1
6161	242015_x_at	1.274266667	7.53E-09	APEH
6162	243045_at	1.274925926	1.08E-09	SMYD1
6163	226092_at	1.275540741	2.65E-12	MPP5
6164	208707_at	1.275592593	5.68E-11	EIF5
6165	204365_s_at	1.275881481	4.24E-07	REEP1
6166	221928_at	1.276225926	7.67E-09	ACACB
6167	214506_at	1.27637037	2.82E-06	GPR182
6168	64408_s_at	1.27762963	7.86E-06	CALML4
6169	232058_at	1.277737037	7.89E-07	
6170	221552_at	1.278118519	2.96E-07	ABHD6
6171	235054_at	1.279718519	4.12E-12	NUDT16
6172	226373_at	1.280159259	6.72E-13	SFXN5
6173	239232_at	1.280218519	5.86E-06	MSI2
6174	228274_at	1.280944444	2.51E-10	SDSL
6175	231034_s_at	1.282462963	3.19E-06	
6176	230503_at	1.283144444	7.27E-07	
6177	1553359_at	1.284181481	1.70E-06	FBXL18
6178	219527_at	1.284318519	6.74E-07	2-Mar
6179	227172_at	1.284933333	5.37E-12	TMEM116
6180	1558487_a_at	1.286522222	1.09E-07	TMED4
6181	219396_s_at	1.286774074	2.40E-09	MIR631 /// NEIL1
6182	236553_at	1.286885185	2.98E-07	LOC100507520
6183	211207_s_at	1.287166667	6.68E-06	ACSL6
6184	229696_at	1.287418519	1.52E-12	FECH
6185	218699_at	1.288481481	6.96E-12	RAB29
6186	230806_s_at	1.288481481	1.64E-07	FAM65A
6187	204000_at	1.289403704	3.21E-15	GNB5
6188	205236_x_at	1.290703704	4.02E-10	SOD3
6189	1559255_a_at	1.291588889	5.84E-06	
6190	212183_at	1.292348148	7.34E-08	NUDT4 /// NUDT4P1
6191	243292_at	1.292418519	1.94E-08	FAM132A
6192	231710_at	1.292666667	4.04E-06	CAPS
6193	1559629_at	1.292685185	7.13E-08	LOC101927710
6194	236010_at	1.293377778	1.85E-07	
6195	239171_at	1.293888889	4.96E-07	
6196	1569202_x_at	1.293896296	8.50E-06	

6197	231296_at	1.293985185	3.25E-09	
6198	227456_s_at	1.294607407	2.82E-15	C6orf136
6199	219156_at	1.294744444	2.36E-13	SYNJ2BP /// SYNJ2BP-COX16
6200	233984_at	1.295251852	2.01E-07	
6201	227524_at	1.295325926	2.61E-09	FZD3
6202	236331_at	1.295414815	9.61E-07	CDKL2
6203	225344_at	1.29552963	2.91E-08	NCOA7
6204	210610_at	1.295985185	8.45E-10	CEACAM1
6205	230054_at	1.296055556	6.43E-07	LOC100507547 /// PRRT1
6206	235955_at	1.29627037	5.67E-06	MARVELD2
6207	205225_at	1.297644444	8.45E-08	ESR1
6208	234079_at	1.297648148	7.50E-07	
6209	239600_at	1.297825926	2.08E-06	
6210	202327_s_at	1.297837037	6.58E-07	LOC101930075 /// PKD1
6211	40560_at	1.299066667	7.82E-09	TBX2
6212	235282_at	1.299385185	1.82E-09	LOC100506325 /// LOC102724532
6213	230780_at	1.299537037	5.38E-07	LINC00886
6214	220372_at	1.299540741	2.07E-09	DNAJC28
6215	214116_at	1.299692593	1.25E-08	BTD
6216	241843_at	1.300022222	2.71E-06	EIF5 /// SNORA28
6217	241787_at	1.300618519	3.30E-08	LOC101927703
6218	226352_at	1.301251852	2.48E-09	JMY
6219	235731_at	1.301540741	2.54E-06	AIPL1
6220	243016_at	1.304822222	1.55E-06	
6221	1561135_at	1.304955556	1.67E-10	
6222	218035_s_at	1.305196296	7.86E-08	RBM47
6223	208369_s_at	1.305411111	1.49E-09	GCDH
6224	225311_at	1.305462963	2.24E-12	IVD
6225	234481_at	1.305518519	1.90E-06	
6226	203546_at	1.305525926	7.40E-12	IPO13
6227	223211_at	1.305588889	2.67E-13	HACL1
6228	227341_at	1.306181481	2.08E-07	BEND7
6229	218789_s_at	1.306507407	3.29E-10	C11orf71
6230	227710_s_at	1.306696296	2.86E-08	TPT1-AS1
6231	201904_s_at	1.307222222	2.25E-11	CTDSPL
6232	244132_x_at	1.308214815	2.21E-08	ZNF518A
6233	212946_at	1.308444444	1.51E-12	VWA8
6234	48106_at	1.308788889	1.77E-15	SLC48A1
6235	217264_s_at	1.309411111	3.21E-06	SCNN1A
6236	218678_at	1.310111111	5.53E-06	NES
6237	1558537_x_at	1.310133333	2.89E-06	ZNF844
6238	215761_at	1.310581481	8.34E-06	DMXL2
6239	211416_x_at	1.311014815	7.07E-06	GGTLC1

6240	218675_at	1.311077778	3.69E-06	SLC22A17
6241	213276_at	1.311348148	8.61E-06	CAMK2B
6242	243010_at	1.311607407	1.02E-06	MSI2
6243	215203_at	1.312392593	3.86E-06	GOLGA4
6244	1552682_a_at	1.312611111	2.54E-07	CASC5
6245	232333_at	1.312792593	1.33E-07	
6246	242469_at	1.313118519	1.73E-06	
6247	238001_at	1.314359259	4.94E-11	KCTD6
6248	221344_at	1.314533333	7.62E-06	OR12D2
6249	225759_x_at	1.314551852	2.90E-09	CLMN
6250	228346_at	1.314762963	7.45E-06	ZNF844
6251	238616_at	1.315085185	5.10E-10	QDPR
6252	227804_at	1.31617037	1.21E-06	TLCD1
6253	214434_at	1.317681481	2.84E-07	HSPA12A
6254	222653_at	1.319466667	4.52E-09	PNPO
6255	226157_at	1.320559259	7.35E-10	TFDP2
6256	225034_at	1.321962963	2.56E-10	ST3GAL1
6257	1554181_at	1.322414815	3.76E-08	SNX32
6258	238744_at	1.322540741	5.19E-06	DPH6
6259	223296_at	1.323144444	2.35E-10	SLC25A33
6260	204042_at	1.323525926	1.01E-07	WASF3
6261	205231_s_at	1.323922222	2.00E-11	EPM2A
6262	203713_s_at	1.324281481	1.14E-12	LLGL2
6263	206820_at	1.326392593	1.99E-06	AGFG2
6264	221627_at	1.326685185	1.63E-06	TRIM10
6265	229222_at	1.327596296	1.35E-08	ACSS3
6266	230113_at	1.327648148	1.46E-06	MBNL3
6267	229455_at	1.328125926	1.98E-09	AC083843.1
6268	212400_at	1.328381481	2.02E-10	FAM102A
6269	214823_at	1.328407407	3.18E-07	ZNF204P
6270	207847_s_at	1.330166667	9.04E-06	MUC1
6271	200979_at	1.3313	9.90E-14	PDHA1
6272	244689_at	1.331696296	1.44E-10	PPARA
6273	224746_at	1.332822222	1.22E-10	KIAA1522
6274	222310_at	1.332911111	1.58E-06	SCAF4
6275	241459_at	1.333359259	8.67E-07	
6276	226741_at	1.335611111	2.93E-12	SLC12A6
6277	227055_at	1.337277778	8.10E-07	METTL7B
6278	201425_at	1.337433333	1.29E-08	ALDH2
6279	225440_at	1.337966667	6.91E-09	AGPAT3
6280	236813_at	1.338233333	1.25E-10	MORN4
6281	218807_at	1.338644444	1.03E-06	VAV3
6282	1553994_at	1.340311111	8.33E-08	NT5E

6283	227412_at	1.341218519	6.68E-11	PPP1R3E
6284	234300_s_at	1.341925926	3.17E-09	ZFP28
6285	205366_s_at	1.342022222	4.74E-10	HOXB6
6286	239999_at	1.342074074	2.40E-07	LINC00478
6287	235505_s_at	1.342814815	8.13E-09	LRPAP1
6288	213093_at	1.343455556	6.41E-09	PRKCA
6289	219501_at	1.345262963	8.36E-09	ENOX1
6290	239711_at	1.345277778	8.18E-11	ADAL
6291	226246_at	1.345562963	2.79E-08	KCTD1
6292	216635_at	1.345622222	5.67E-07	
6293	238155_at	1.345881481	4.44E-11	RP11-140I16.3
6294	242169_at	1.346196296	7.48E-06	BHMT2
6295	240842_at	1.346218519	4.22E-06	LOC101927020
6296	226101_at	1.346796296	3.92E-10	PRKCE
6297	1552877_s_at	1.347503704	4.50E-07	LINC00334
6298	217080_s_at	1.347718519	1.18E-06	HOMER2
6299	213849_s_at	1.348125926	8.94E-13	PPP2R2B
6300	239252_at	1.348133333	2.89E-07	COX7B
6301	229427_at	1.3484	3.06E-10	SEMA5A
6302	242143_at	1.348437037	5.97E-09	
6303	201035_s_at	1.349592593	1.11E-12	HADH
6304	242022_at	1.351977778	4.39E-09	
6305	213912_at	1.352596296	1.15E-06	TBC1D30
6306	226041_at	1.352851852	5.98E-11	NAPEPLD
6307	229523_at	1.3529	6.86E-06	TMEM200C
6308	208557_at	1.354066667	7.22E-10	HOXA6
6309	235883_at	1.354274074	1.99E-06	LOC101928068
6310	219113_x_at	1.354918519	1.91E-06	HSD17B14
6311	218688_at	1.35622963	2.12E-11	DAK
6312	235605_at	1.356277778	3.02E-08	CASZ1
6313	223915_at	1.357033333	1.08E-08	BCOR
6314	203995_at	1.357277778	5.18E-06	C21orf2
6315	239757_at	1.357296296	6.13E-06	ZFAND6
6316	212151_at	1.357640741	7.45E-09	PBX1
6317	218394_at	1.358718519	4.67E-11	ROGDI
6318	1558760_at	1.358737037	3.97E-06	
6319	212814_at	1.358840741	6.76E-10	AHCYL2
6320	225726_s_at	1.359218519	3.44E-10	PLEKHH1
6321	121_at	1.359485185	3.01E-09	PAX8
6322	204813_at	1.359518519	1.19E-13	MAPK10
6323	241839_at	1.361951852	1.08E-06	DLG3-AS1
6324	243280_at	1.362737037	3.43E-06	
6325	232167_at	1.362918519	2.51E-10	SLC2A11

6326	238824_at	1.363037037	6.12E-11	RPS29
6327	219168_s_at	1.363403704	3.05E-09	PRR5
6328	1558631_at	1.364622222	6.35E-11	PPARA
6329	206847_s_at	1.364674074	2.52E-09	HOXA7
6330	238472_at	1.364766667	4.11E-10	FBXO9
6331	237172_at	1.365111111	5.47E-12	
6332	213264_at	1.365644444	1.70E-11	PCBP2
6333	1561079_at	1.366059259	2.84E-08	ANKRD28
6334	1563800_at	1.366574074	3.35E-09	LOC100506870 /// LOC283140
6335	233696_at	1.366885185	3.67E-10	
6336	218700_s_at	1.367614815	2.70E-12	RAB29
6337	1556195_a_at	1.3683	1.49E-06	RP4-798A10.7
6338	220178_at	1.368844444	5.07E-09	MFSD12
6339	242188_at	1.368911111	2.41E-08	
6340	213355_at	1.36947037	3.36E-07	ST3GAL6
6341	216579_at	1.36952963	1.80E-07	GJB4
6342	238077_at	1.370277778	3.91E-13	KCTD6
6343	220035_at	1.37207037	9.83E-06	NUP210
6344	220573_at	1.37322963	4.38E-08	KLK14
6345	237035_at	1.373285185	7.94E-08	RP11-319G9.3
6346	236998_at	1.3742	4.17E-10	COA3
6347	1568796_at	1.375196296	3.97E-07	CCDC157
6348	228848_at	1.377040741	9.78E-06	ABTB1
6349	222941_at	1.377125926	1.83E-06	USP46
6350	244362_at	1.377266667	4.63E-10	RP11-672L10.6
6351	225779_at	1.377733333	9.39E-10	SLC27A4
6352	228538_at	1.377811111	7.47E-08	ZNF662
6353	239000_at	1.378837037	4.07E-06	BRD4
6354	239297_at	1.378962963	1.35E-06	KIAA1456 /// LOC101927137
6355	222520_s_at	1.379014815	1.86E-09	IFT57
6356	227775_at	1.379244444	1.42E-12	CELF6
6357	239238_at	1.379688889	3.81E-11	
6358	213183_s_at	1.379933333	5.55E-10	
6359	234446_at	1.380611111	7.64E-06	
6360	1564308_a_at	1.381207407	4.85E-09	MPP7
6361	204348_s_at	1.382103704	1.62E-07	AK4 /// LOC100507855
6362	229424_s_at	1.38292963	6.56E-06	ARHGAP27
6363	227756_at	1.383203704	5.25E-07	FAM81A
6364	233599_at	1.383207407	1.29E-06	LOC728061
6365	222764_at	1.383277778	5.86E-07	ASRGL1
6366	242416_at	1.383318519	8.27E-09	
6367	215321_at	1.383348148	3.37E-06	RUNDC3B
6368	235753_at	1.383681481	6.91E-09	HOXA7

6369	239222_at	1.384159259	1.09E-06	C9orf9
6370	224494_x_at	1.384455556	1.36E-06	HSD17B14
6371	217133_x_at	1.384788889	8.54E-08	CYP2B6
6372	204462_s_at	1.386655556	5.33E-09	SLC16A2
6373	1558404_at	1.387192593	1.19E-07	LINC00622
6374	238282_at	1.387440741	5.01E-07	
6375	227829_at	1.387733333	1.25E-11	GYLTL1B
6376	233158_at	1.387792593	3.02E-06	KRT82
6377	231050_at	1.387988889	2.00E-09	HRASLS5
6378	239161_at	1.388111111	1.86E-11	FDX1
6379	223426_s_at	1.38877037	7.97E-11	EPB41L4B
6380	223800_s_at	1.388825926	3.37E-07	LIMS3 /// LIMS3L
6381	201900_s_at	1.388981481	8.35E-11	AKR1A1
6382	220089_at	1.388992593	1.87E-12	L2HGDH
6383	215945_s_at	1.389007407	1.01E-06	TRIM2
6384	208868_s_at	1.389718519	3.44E-09	GABARAPL1
6385	244509_at	1.389811111	1.33E-07	GPR155
6386	219395_at	1.389825926	1.40E-10	ESRP2 /// MIR6773
6387	232017_at	1.390562963	1.85E-06	TJP2
6388	228235_at	1.39207037	1.35E-08	MIR424 /// MIR503HG
6389	233036_at	1.392814815	1.43E-07	
6390	241584_at	1.392940741	2.87E-06	
6391	244730_x_at	1.392959259	2.04E-07	ZSCAN16-AS1
6392	1552303_a_at	1.395237037	1.40E-11	TMEM106A
6393	231324_at	1.395266667	1.47E-06	
6394	228195_at	1.396392593	9.31E-06	C2orf88
6395	229415_at	1.397403704	4.28E-11	CYCS
6396	229780_at	1.397403704	3.68E-06	
6397	230911_at	1.399392593	2.27E-08	SIX1
6398	225874_at	1.3995	1.09E-07	UBALD1
6399	223183_at	1.39987037	3.90E-13	AGPAT3
6400	222333_at	1.400311111	4.43E-13	ALS2CL
6401	212991_at	1.400644444	3.63E-09	FBXO9
6402	214748_at	1.40107037	7.19E-09	N4BP2L2
6403	230305_at	1.401622222	5.10E-09	DNAJC15
6404	201860_s_at	1.401674074	1.96E-06	PLAT
6405	1565863_at	1.402059259	1.53E-07	
6406	222350_at	1.402240741	6.17E-07	
6407	238124_at	1.40247037	4.94E-08	MYOM3
6408	219223_at	1.402714815	2.71E-11	CACFD1
6409	238515_at	1.403237037	6.60E-12	NUDT16
6410	231619_at	1.403396296	4.15E-06	
6411	214187_x_at	1.403725926	9.88E-06	CTDSPL

6412	229636_at	1.403807407	3.80E-06	CYB561D2
6413	217076_s_at	1.405062963	3.87E-06	HOXD3 /// HOXD4 /// LOC401021
6414	1558154_at	1.405637037	4.04E-07	AF289551
6415	244084_at	1.406011111	7.18E-06	AIFM3
6416	222830_at	1.406455556	2.60E-08	GRHL1
6417	204389_at	1.406566667	5.63E-07	MAOA
6418	243362_s_at	1.407318519	9.75E-07	LEF1-AS1
6419	238081_at	1.409066667	1.66E-08	WDFY3-AS2
6420	224821_at	1.4098	3.98E-13	ABHD14B
6421	242645_at	1.410385185	1.08E-09	
6422	240282_at	1.410574074	2.65E-07	WDR1
6423	228656_at	1.410781481	4.10E-06	PROX1
6424	1554017_at	1.411407407	8.69E-06	RILPL1 /// SNRNP35
6425	216958_s_at	1.411537037	4.15E-14	IVD
6426	1569683_at	1.41167037	1.90E-15	XYLB
6427	230840_at	1.412148148	3.69E-08	SMIM1
6428	243379_at	1.412737037	1.63E-07	
6429	237895_at	1.413388889	7.05E-06	
6430	227944_at	1.413648148	1.70E-07	PTPN3
6431	220894_x_at	1.414881481	9.22E-07	PRDM12
6432	1555624_a_at	1.415007407	3.24E-07	SSH1
6433	205112_at	1.416718519	4.56E-08	PLCE1
6434	1557336_at	1.417685185	9.37E-07	SUCLG2-AS1
6435	236885_at	1.417766667	2.87E-06	MEX3A
6436	235688_s_at	1.418	3.40E-08	TRAF4
6437	1555922_at	1.418048148	2.73E-06	CASC10
6438	205843_x_at	1.418074074	5.82E-06	CRAT
6439	205012_s_at	1.418911111	1.15E-10	HAGH
6440	239452_at	1.41932963	3.08E-10	
6441	202342_s_at	1.41977037	9.26E-08	TRIM2
6442	215584_at	1.420322222	8.26E-09	HECW1
6443	220824_at	1.421025926	6.60E-06	
6444	242417_at	1.421544444	3.81E-07	LOC283278 /// PLEKHA7
6445	229396_at	1.422111111	6.40E-10	OVOL1
6446	210045_at	1.422185185	1.36E-11	IDH2
6447	1558613_at	1.422455556	2.60E-07	
6448	228411_at	1.423007407	1.09E-08	PARD3B
6449	242863_at	1.424662963	1.95E-06	
6450	217509_x_at	1.424818519	1.13E-08	GRIK5
6451	221141_x_at	1.425255556	9.65E-06	EPN1
6452	218489_s_at	1.425637037	1.66E-10	ALAD
6453	1558052_at	1.425737037	2.04E-08	TMED4
6454	206629_at	1.42662963	3.29E-07	ADAMTSL2

6455	228646_at	1.42687037	1.02E-09	PPP1R1C
6456	239179_at	1.427385185	3.22E-06	
6457	221669_s_at	1.42797037	1.38E-13	ACAD8
6458	209703_x_at	1.4281	3.02E-06	METTL7A
6459	203159_at	1.428348148	1.93E-08	GLS
6460	226534_at	1.428448148	5.76E-08	KITLG
6461	233520_s_at	1.429177778	5.30E-06	CMYA5
6462	243791_at	1.4293	3.80E-09	
6463	229534_at	1.430422222	3.24E-07	ACOT4
6464	244227_at	1.430866667	4.75E-10	SYT6
6465	232439_at	1.431066667	4.26E-07	
6466	202780_at	1.431192593	4.47E-08	OXCT1
6467	202017_at	1.43127037	3.25E-09	EPHX1
6468	213739_at	1.431433333	2.86E-10	LOC103344931
6469	235017_s_at	1.431940741	4.68E-09	CSRNP3
6470	1561615_s_at	1.43312963	5.25E-07	SLC8A1
6471	226855_at	1.433314815	1.41E-16	PDP2
6472	240440_at	1.433918519	6.76E-07	NPL
6473	1553499_s_at	1.434211111	3.67E-06	SERPINA9
6474	241928_at	1.434485185	2.21E-08	
6475	204613_at	1.434922222	4.44E-11	PLCG2
6476	214231_s_at	1.435248148	4.29E-11	VWA8
6477	207024_at	1.435455556	8.41E-06	CHRND
6478	213263_s_at	1.435851852	1.25E-10	PCBP2
6479	211883_x_at	1.435885185	1.00E-08	CEACAM1
6480	203608_at	1.436355556	1.16E-07	ALDH5A1
6481	203157_s_at	1.436425926	5.30E-08	GLS
6482	213067_at	1.436796296	1.89E-09	MYH10
6483	241201_at	1.4369	8.09E-06	UVSSA
6484	229692_at	1.436981481	3.63E-06	
6485	243266_at	1.437877778	2.72E-07	
6486	231734_at	1.437981481	1.91E-06	RBP2
6487	221958_s_at	1.438140741	1.15E-10	WLS
6488	235086_at	1.438259259	7.88E-06	THBS1
6489	242033_at	1.438355556	8.65E-08	RNF180
6490	213929_at	1.439181481	9.06E-09	EXPH5
6491	209420_s_at	1.439803704	5.97E-13	SMPD1
6492	230221_at	1.440122222	6.25E-06	ABHD16A
6493	228888_at	1.440125926	1.45E-08	STAC2
6494	239017_at	1.441344444	4.90E-06	
6495	240598_at	1.441840741	3.64E-07	RP11-1151B14.3
6496	233198_at	1.442925926	9.07E-10	GOLGA2P5
6497	208251_at	1.445281481	3.40E-07	KCNC4

6498	205353_s_at	1.446018519	2.01E-11	PEBP1
6499	223442_at	1.447233333	1.38E-15	NICN1
6500	226647_at	1.4479	1.26E-08	TMEM25
6501	226198_at	1.447925926	2.09E-15	TOM1L2
6502	234034_at	1.448344444	3.00E-06	
6503	1556413_a_at	1.44947037	6.79E-08	
6504	239861_at	1.449614815	4.55E-06	
6505	228952_at	1.4497	3.43E-06	ENPP1
6506	1560291_at	1.449881481	5.17E-06	RIPPLY1
6507	205222_at	1.449885185	7.98E-06	EHHADH
6508	226469_s_at	1.450040741	5.51E-06	GGT7
6509	230677_at	1.450188889	5.89E-06	EXOC3L4
6510	230449_x_at	1.451718519	2.69E-11	RP11-410L14.2
6511	214192_at	1.4518	5.51E-06	NUP88
6512	243158_at	1.452225926	3.94E-07	
6513	219509_at	1.452448148	5.12E-08	MYOZ1
6514	207071_s_at	1.453511111	1.54E-10	ACO1
6515	202502_at	1.454240741	2.22E-11	ACADM
6516	243066_at	1.454651852	1.96E-07	NPL
6517	230743_at	1.45487037	4.83E-11	HOXB-AS3
6518	237117_at	1.455507407	5.41E-10	AC112198.1
6519	219474_at	1.456377778	1.74E-06	C3orf52
6520	228949_at	1.456544444	8.86E-11	WLS
6521	236635_at	1.457222222	1.16E-07	ZNF667
6522	223437_at	1.458111111	7.97E-14	PPARA
6523	203790_s_at	1.458837037	3.26E-07	HRSP12
6524	214308_s_at	1.460274074	3.80E-06	HGD
6525	234435_at	1.460325926	3.46E-09	
6526	242320_at	1.460418519	2.12E-08	
6527	1553923_at	1.461274074	8.55E-07	SLC22A24
6528	217775_s_at	1.463248148	9.79E-16	RDH11
6529	215277_at	1.463744444	7.96E-06	PCDH1
6530	243053_x_at	1.464555556	4.47E-06	LOC100128079
6531	227296_at	1.464644444	2.42E-08	MFSD3
6532	220105_at	1.464740741	7.93E-06	RTDR1
6533	211531_x_at	1.465392593	8.52E-08	PRB1
6534	45288_at	1.466685185	6.65E-09	ABHD6
6535	214969_at	1.468222222	2.80E-06	MAP3K9
6536	242341_x_at	1.468818519	4.40E-13	GLYCTK
6537	241563_at	1.469085185	3.90E-06	RP11-384L8.1
6538	244172_at	1.469892593	1.46E-06	
6539	209737_at	1.470011111	7.28E-12	MAGI2
6540	239097_at	1.470055556	6.96E-06	FRRS1L

6541	209577_at	1.470281481	2.72E-10	PCYT2
6542	1560741_at	1.470285185	7.17E-07	SNRPN /// SNURF
6543	203335_at	1.470318519	1.10E-11	PHYH
6544	239816_at	1.47082963	1.81E-06	POLD3
6545	216083_s_at	1.471896296	4.78E-06	NEU3
6546	225458_at	1.472959259	5.84E-07	PP7080
6547	218025_s_at	1.473637037	2.10E-13	ECI2
6548	244457_at	1.47382963	3.84E-10	
6549	232859_s_at	1.474992593	1.24E-12	MAGI1
6550	230174_at	1.475274074	7.04E-13	LYPLAL1
6551	232990_at	1.476044444	2.25E-09	FAM104B
6552	1559591_s_at	1.476644444	3.69E-08	CHDH
6553	209123_at	1.477137037	1.58E-11	QDPR
6554	242359_at	1.478625926	6.46E-06	
6555	236874_at	1.479118519	6.30E-07	
6556	230481_at	1.479588889	3.40E-06	ACY3
6557	241356_at	1.479981481	5.95E-06	SDR42E1
6558	225342_at	1.480814815	1.76E-08	AK4 /// LOC100507855
6559	1569601_at	1.480977778	4.54E-06	
6560	233161_at	1.481603704	1.76E-07	
6561	242053_at	1.481688889	2.39E-07	
6562	229223_at	1.482240741	1.68E-07	RP11-96D1.11
6563	241230_at	1.483207407	9.48E-06	CA12
6564	205052_at	1.48322963	4.11E-09	AUH
6565	219689_at	1.485255556	4.44E-08	SEMA3G
6566	1556891_at	1.485696296	2.57E-06	SORCS1
6567	1555868_at	1.486544444	6.18E-08	LOC100507477
6568	209692_at	1.486840741	7.51E-06	EYA2
6569	220116_at	1.487092593	1.39E-06	KCNN2
6570	207761_s_at	1.487603704	1.31E-06	METTL7A
6571	241975_at	1.488433333	8.07E-06	
6572	205512_s_at	1.488481481	9.57E-10	AIFM1
6573	213664_at	1.491640741	9.37E-07	SLC1A1
6574	219682_s_at	1.492618519	2.36E-07	TBX3
6575	1553857_at	1.492981481	1.95E-06	IGSF22
6576	228877_at	1.493633333	2.84E-08	RGL3
6577	1566571_at	1.494125926	9.02E-08	
6578	229385_s_at	1.49462963	2.66E-07	TINCR
6579	227234_at	1.494781481	6.14E-08	IPO5P1
6580	214489_at	1.495244444	4.94E-07	FSHB
6581	209559_at	1.495311111	2.64E-07	HIP1R
6582	229909_at	1.495933333	3.23E-07	B4GALNT3

6583	1569607_s_at	1.495996296	4.77E-08	ANKRD20A1 /// ANKRD20A11P /// ANKRD20A2 /// ANKRD20A3 /// ANKRD20A4 /// ANKRD20A5P /// ANKRD20A9P /// LOC102723891 /// LOC102725051
6584	229647_at	1.496074074	1.13E-14	NDUFS1
6585	1556205_at	1.496707407	5.19E-09	
6586	202524_s_at	1.497059259	1.35E-08	SPOCK2
6587	217776_at	1.497159259	3.90E-15	RDH11
6588	211347_at	1.497203704	1.49E-06	CDC14B
6589	237400_at	1.497625926	2.71E-10	RP11-247L20.4
6590	1559820_at	1.497840741	8.58E-08	
6591	227446_s_at	1.498466667	5.73E-10	DHRS4-AS1
6592	208851_s_at	1.498825926	1.15E-09	THY1
6593	242327_x_at	1.499155556	1.54E-07	
6594	202661_at	1.499474074	2.08E-13	ITPR2
6595	240458_at	1.500018519	3.01E-09	
6596	1553361_x_at	1.500166667	1.43E-08	FBXL18
6597	206548_at	1.500333333	1.32E-06	
6598	1553721_at	1.502333333	1.12E-07	RNF152
6599	221262_s_at	1.502848148	1.84E-12	SLC2A11
6600	217914_at	1.503096296	8.95E-09	TPCN1
6601	227274_at	1.503096296	1.69E-17	SYNJ2BP /// SYNJ2BP-COX16
6602	208581_x_at	1.503981481	1.48E-06	MT1X
6603	208435_s_at	1.504818519	4.15E-06	AQP6
6604	1557825_at	1.505033333	9.95E-07	RP11-1E4.1
6605	241898_at	1.506074074	5.89E-06	LIPH
6606	204586_at	1.508344444	7.68E-06	BSN
6607	241950_at	1.508351852	8.38E-09	WWC1
6608	232819_s_at	1.508548148	1.43E-06	LTBR
6609	223469_at	1.509137037	1.23E-10	PGPEP1
6610	226433_at	1.51072963	7.99E-08	RNF157
6611	211889_x_at	1.511237037	3.17E-07	CEACAM1
6612	241897_at	1.511322222	6.64E-07	
6613	228096_at	1.511422222	1.70E-10	MINOS1
6614	229246_at	1.511703704	6.25E-07	SRSF1
6615	205977_s_at	1.511811111	2.84E-08	EPHA1
6616	209957_s_at	1.512266667	1.00E-08	NPPA
6617	1554094_at	1.513851852	4.22E-09	ENTPD5
6618	1559412_at	1.513974074	7.41E-06	LINC00478
6619	210653_s_at	1.514107407	1.65E-10	BCKDHB
6620	216316_x_at	1.514544444	2.92E-08	RP11-548H18.2
6621	240110_at	1.515244444	2.55E-09	HMGCS2
6622	228601_at	1.515614815	5.81E-06	HAGLR

6623	206242_at	1.516022222	6.14E-06	TM4SF5
6624	227195_at	1.516781481	7.46E-10	ZNF503
6625	238721_at	1.517962963	2.52E-07	MDH1B
6626	217421_at	1.518222222	1.40E-07	PIWIL2
6627	242407_at	1.518251852	4.05E-09	
6628	222831_at	1.518514815	2.36E-07	SAP30L
6629	238753_at	1.518825926	6.92E-08	NCS1
6630	238228_at	1.519085185	8.04E-08	
6631	214417_s_at	1.519874074	4.42E-07	
6632	225739_at	1.521637037	2.62E-09	RAB11FIP4
6633	229107_at	1.523766667	2.13E-09	
6634	205632_s_at	1.524151852	8.99E-12	PIP5K1B
6635	203642_s_at	1.524307407	4.59E-08	COBLL1
6636	234312_s_at	1.524785185	8.55E-13	ACSS2
6637	219215_s_at	1.525218519	1.24E-07	SLC39A4
6638	217600_at	1.525414815	8.08E-06	SCUBE3
6639	207493_x_at	1.525503704	1.42E-06	SSX2 /// SSX2B
6640	231552_at	1.525507407	1.68E-07	
6641	237849_at	1.525796296	2.54E-08	
6642	203711_s_at	1.525918519	1.25E-11	HIBCH
6643	242354_at	1.525985185	1.99E-09	RP11-532F12.5
6644	237498_at	1.527511111	1.36E-07	
6645	206338_at	1.527944444	4.31E-08	ELAVL3
6646	210852_s_at	1.528285185	2.48E-08	AASS
6647	212694_s_at	1.528403704	9.39E-13	PCCB
6648	1556355_x_at	1.528937037	6.51E-07	RGL3
6649	234948_at	1.528962963	5.64E-06	SLC27A5
6650	238715_at	1.529581481	2.50E-06	LOC646014
6651	1557207_s_at	1.529781481	1.05E-07	LOC283177
6652	240249_at	1.530385185	2.80E-06	
6653	208429_x_at	1.5305	3.70E-06	HNF4A
6654	202890_at	1.530592593	1.83E-06	MAP7
6655	238163_at	1.530707407	2.58E-11	LOC102725343
6656	230475_at	1.531614815	9.86E-10	C15orf59
6657	223315_at	1.531885185	7.78E-06	NTN4
6658	232484_at	1.531977778	2.77E-06	
6659	231161_x_at	1.531985185	7.15E-06	TRIM8
6660	221655_x_at	1.53237037	2.65E-06	EPS8L1
6661	241401_at	1.532685185	9.33E-06	WDFY3-AS2
6662	209552_at	1.533366667	3.09E-07	PAX8
6663	230764_at	1.534311111	5.01E-08	
6664	230803_s_at	1.53512963	2.72E-07	ARHGAP24
6665	1552261_at	1.535207407	2.91E-07	WFDC2

6666	229245_at	1.535307407	1.21E-09	PLEKHA6
6667	215700_x_at	1.535748148	4.37E-06	CPNE6
6668	202054_s_at	1.535792593	4.97E-09	ALDH3A2
6669	203671_at	1.536377778	2.80E-09	TPMT
6670	238999_at	1.536903704	9.31E-06	
6671	235525_at	1.53692963	1.29E-06	
6672	203586_s_at	1.537192593	3.13E-11	ARL4D
6673	205826_at	1.537211111	7.07E-08	MYOM2
6674	243430_at	1.537855556	3.13E-07	SEZ6
6675	1557239_at	1.537992593	6.07E-07	BBX
6676	236997_at	1.539362963	3.31E-06	LOC101928069
6677	231031_at	1.539714815	4.36E-07	KGFLP2
6678	233265_at	1.539914815	9.89E-06	
6679	234495_at	1.539937037	4.64E-06	KLK15
6680	218541_s_at	1.540674074	1.02E-06	C8orf4
6681	212741_at	1.541314815	7.17E-07	MAOA
6682	242358_at	1.541666667	1.08E-09	RASSF8-AS1
6683	220462_at	1.541892593	5.99E-06	CSRNP3
6684	226292_at	1.542937037	2.23E-09	CAPN5
6685	1562953_s_at	1.543244444	3.03E-11	WDFY3-AS2
6686	229963_at	1.543677778	2.58E-07	BEX5
6687	236621_at	1.544177778	6.13E-10	RPS27
6688	216022_at	1.545181481	1.78E-08	
6689	239349_at	1.546959259	1.47E-09	C1QTNF7
6690	236004_at	1.547507407	5.17E-18	AP5M1
6691	211431_s_at	1.547759259	6.41E-11	TYRO3
6692	225627_s_at	1.548674074	1.38E-07	CACHD1
6693	1557400_at	1.5492	2.31E-09	
6694	213558_at	1.549525926	8.31E-06	PCLO
6695	220574_at	1.549981481	1.26E-07	SEMA6D
6696	240039_at	1.550237037	1.39E-09	PLA2R1
6697	234066_at	1.550544444	3.27E-06	IL1RL1
6698	1556078_at	1.550648148	1.93E-07	
6699	40273_at	1.550659259	2.11E-16	SPHK2
6700	240760_at	1.551085185	5.05E-06	CDRT15
6701	211600_at	1.551725926	1.82E-09	PTPRO
6702	201397_at	1.552162963	8.68E-07	PHGDH
6703	201578_at	1.553681481	2.67E-08	PODXL
6704	239847_at	1.553840741	6.19E-09	
6705	1560652_at	1.554185185	8.95E-06	RP6-24A23.7
6706	1558815_at	1.554603704	6.46E-07	SORBS2
6707	222368_at	1.554703704	1.05E-07	
6708	1557292_a_at	1.556018519	8.51E-07	MCOLN3

6709	239091_at	1.556711111	6.18E-06	
6710	241351_at	1.557055556	5.55E-07	
6711	242752_at	1.557240741	8.40E-06	PPM1K
6712	239666_at	1.557648148	6.06E-06	PYGO2
6713	227494_at	1.558740741	1.46E-09	NR6A1
6714	1555198_x_at	1.558877778	6.59E-07	C21orf58
6715	209094_at	1.559614815	4.31E-08	DDAH1
6716	226773_at	1.560040741	9.61E-15	PPM1K
6717	226582_at	1.560422222	1.23E-07	LOC400043
6718	213965_s_at	1.560433333	4.00E-06	CHD5
6719	1564757_a_at	1.561362963	5.61E-07	CCDC148
6720	224818_at	1.562155556	1.66E-12	SORT1
6721	232128_s_at	1.562433333	5.62E-08	CLCN5
6722	1553041_at	1.5625	1.11E-06	HTR3C
6723	220333_at	1.562959259	8.64E-06	PAQR5
6724	220951_s_at	1.563007407	4.06E-06	A1CF
6725	205405_at	1.56412963	8.90E-11	SEMA5A
6726	232979_at	1.564762963	2.55E-08	MIR10A
6727	229729_at	1.565255556	3.02E-07	TMEM8B
6728	231542_at	1.566755556	2.73E-06	SPAG5-AS1
6729	223438_s_at	1.566974074	1.90E-13	PPARA
6730	226978_at	1.567374074	1.20E-12	PPARA
6731	210063_at	1.567988889	1.43E-07	SARDH
6732	203145_at	1.568262963	2.15E-07	SPAG5
6733	239684_at	1.568637037	4.33E-08	TRPM3
6734	205051_s_at	1.568796296	5.59E-07	KIT
6735	239484_at	1.568837037	9.88E-08	TRPM7
6736	227113_at	1.569022222	1.43E-11	ADHFE1
6737	221163_s_at	1.569514815	5.26E-07	MLXIPL
6738	1554677_s_at	1.570159259	1.45E-09	CMTM4
6739	204019_s_at	1.570733333	4.07E-07	SH3YL1
6740	223422_s_at	1.571144444	1.75E-08	ARHGAP24
6741	241823_at	1.571144444	5.85E-14	
6742	1556510_at	1.571411111	5.70E-06	NFIA-AS2
6743	1553589_a_at	1.571888889	1.49E-06	PDZK1IP1
6744	205498_at	1.572007407	3.00E-06	GHR
6745	227997_at	1.574451852	3.14E-08	IL17RD
6746	52940_at	1.574988889	4.56E-11	SIGIRR
6747	219630_at	1.575211111	2.55E-06	PDZK1IP1
6748	211778_s_at	1.575437037	5.28E-13	OVOL2
6749	239246_at	1.575481481	3.34E-08	FARP1
6750	1558077_s_at	1.576007407	9.18E-09	MDH1B
6751	241397_at	1.576433333	7.63E-06	

6752	1555216_a_at	1.576877778	1.71E-10	LOC101060604 /// SLC7A5P1 /// SLC7A5P2
6753	243774_at	1.577718519	1.81E-06	MUC20
6754	236587_at	1.57787037	6.47E-12	LRRC6
6755	227591_at	1.578037037	4.27E-09	SH3BP5-AS1
6756	1558748_at	1.578485185	1.83E-07	
6757	240216_at	1.578988889	1.71E-07	
6758	1552504_a_at	1.579014815	3.10E-06	BRSK1
6759	231541_s_at	1.579311111	4.82E-08	SPAG5-AS1
6760	1567101_at	1.579618519	5.18E-06	
6761	205066_s_at	1.579714815	3.21E-06	ENPP1
6762	212372_at	1.579862963	1.48E-10	MYH10
6763	219305_x_at	1.579911111	2.93E-12	FBXO2
6764	205104_at	1.580888889	1.16E-07	SNPH
6765	222293_at	1.582248148	3.77E-08	CADM4
6766	233550_s_at	1.582348148	4.33E-09	SLC4A11
6767	209498_at	1.583177778	1.32E-07	CEACAM1
6768	223339_at	1.583803704	1.37E-13	ATPIF1
6769	236660_at	1.584055556	6.40E-09	
6770	1565661_x_at	1.584455556	1.59E-07	FUT6
6771	227949_at	1.58452963	1.50E-06	PHACTR3
6772	216434_at	1.585451852	1.11E-06	TTC38
6773	228730_s_at	1.585451852	7.18E-10	SCRN2
6774	228407_at	1.585625926	2.02E-07	SCUBE3
6775	236097_at	1.585940741	1.62E-13	
6776	242541_at	1.586237037	2.17E-07	ABCA9
6777	232192_at	1.586692593	2.05E-06	LOC153811 /// RNF130
6778	208048_at	1.588881481	2.55E-06	TACR1
6779	231705_at	1.589140741	2.70E-08	HRSP12
6780	1566491_at	1.589862963	9.77E-07	
6781	243291_at	1.590296296	8.24E-06	
6782	1563473_at	1.590525926	2.39E-07	
6783	236458_at	1.590688889	3.77E-14	
6784	241853_at	1.591125926	2.88E-06	
6785	205111_s_at	1.5921	2.93E-09	PLCE1
6786	214920_at	1.5922	1.36E-06	THSD7A
6787	228370_at	1.593385185	4.40E-07	IPW /// LOC101930404 /// PWARSN /// SNORD107 /// SNORD115-13 /// SNORD115-26 /// SNORD115-7 /// SNORD116-22 /// SNORD116-28 /// SNORD116-4
6788	226490_at	1.59577037	1.44E-12	NHSL1
6789	220150_s_at	1.595840741	2.00E-13	FAM184A
6790	241835_at	1.595948148	3.22E-08	
6791	232568_at	1.596381481	4.50E-07	MGC24103

6792	229258_at	1.596422222	1.35E-06	KIF12
6793	241140_at	1.596674074	2.35E-06	LMO7
6794	210006_at	1.597451852	1.31E-12	ABHD14A /// ACY1
6795	236428_at	1.597925926	5.59E-06	
6796	230003_at	1.597966667	1.09E-07	SLC16A7
6797	242680_at	1.598014815	5.51E-06	AVPR1A
6798	220740_s_at	1.598651852	5.97E-13	SLC12A6
6799	226245_at	1.598922222	2.50E-09	KCTD1
6800	228463_at	1.599392593	8.34E-07	FOXA3
6801	1553998_at	1.599474074	9.15E-13	DMRTC1 /// DMRTC1B
6802	37117_at	1.60107037	4.55E-08	ARHGAP8 /// PRR5-ARHGAP8
6803	207742_s_at	1.601325926	7.00E-08	NR6A1
6804	202962_at	1.601466667	4.42E-15	KIF13B
6805	229130_at	1.60277037	3.29E-08	AX746755 /// RP11-774O3.3
6806	1558780_a_at	1.602840741	3.29E-06	CTC-203F4.2 /// H2AFY
6807	244579_at	1.6037	5.21E-07	
6808	1556730_at	1.604103704	4.02E-06	LOC652993
6809	232531_at	1.606911111	2.51E-06	EMX2OS
6810	1560697_at	1.6073	5.90E-12	TRHDE-AS1
6811	237029_at	1.607485185	8.10E-06	HGD
6812	213385_at	1.607755556	3.16E-11	CHN2
6813	235141_at	1.608907407	2.08E-08	MARVELD2
6814	210358_x_at	1.609151852	2.21E-09	GATA2
6815	223377_x_at	1.609896296	5.04E-14	CISH
6816	203474_at	1.609940741	8.43E-09	IQGAP2
6817	236543_at	1.610044444	2.42E-06	
6818	214091_s_at	1.611507407	6.13E-07	GPX3
6819	232692_at	1.611855556	2.26E-06	TDRD6
6820	232592_at	1.61187037	1.53E-07	
6821	218416_s_at	1.612218519	4.36E-11	SLC48A1
6822	220910_at	1.612511111	6.49E-07	FRAS1
6823	237128_at	1.61267037	8.30E-07	
6824	225033_at	1.613666667	3.28E-11	ST3GAL1
6825	239772_x_at	1.613877778	1.71E-06	DHX30
6826	209558_s_at	1.614007407	2.00E-07	HIP1R
6827	220773_s_at	1.614522222	8.77E-13	GPHN
6828	226625_at	1.615803704	1.80E-08	TGFBR3
6829	217066_s_at	1.616474074	1.86E-06	DMPK
6830	228882_at	1.616514815	6.67E-07	TUB
6831	242317_at	1.616533333	1.57E-14	HIGD1A
6832	232276_at	1.617259259	1.40E-07	HS6ST3
6833	225671_at	1.617562963	1.11E-12	SPNS2
6834	229380_at	1.617637037	2.24E-11	ILDR2

6835	1562406_at	1.6183	1.48E-06	
6836	218677_at	1.618362963	2.23E-08	S100A14
6837	242110_at	1.618603704	3.77E-09	
6838	1553292_s_at	1.618848148	8.80E-07	SGK494
6839	225855_at	1.618911111	1.40E-11	EPB41L5
6840	225474_at	1.619562963	4.25E-12	MAGI1
6841	239173_at	1.619944444	9.49E-09	INADL
6842	244477_at	1.620440741	5.46E-09	
6843	211598_x_at	1.62112963	4.27E-06	VIPR2
6844	213169_at	1.621544444	1.20E-08	SEMA5A
6845	230578_at	1.621711111	3.56E-06	ZNF471
6846	236520_at	1.621777778	7.23E-09	
6847	235958_at	1.622048148	1.34E-07	PLA2G4F
6848	243792_x_at	1.623014815	1.37E-08	PTPN13
6849	1553977_a_at	1.623422222	7.91E-11	CYP39A1
6850	213869_x_at	1.624211111	4.07E-10	THY1
6851	214376_at	1.625392593	2.59E-09	MAPK10
6852	244744_at	1.625444444	2.53E-06	LOC100130502
6853	1565833_at	1.62577037	4.29E-11	
6854	235742_at	1.625774074	1.42E-07	RHOC
6855	201135_at	1.62602963	7.60E-15	ECHS1
6856	244827_at	1.626592593	4.34E-06	
6857	204154_at	1.627896296	9.25E-08	CDO1
6858	230268_at	1.628674074	4.10E-06	
6859	242993_at	1.628851852	3.89E-06	
6860	219107_at	1.629640741	7.43E-06	BCAN
6861	235977_at	1.629781481	1.04E-06	LONRF2
6862	213569_at	1.629796296	5.39E-08	LOC100506603
6863	232244_at	1.632144444	5.56E-14	KIAA1161
6864	204509_at	1.632203704	6.98E-09	CA12
6865	230300_at	1.632437037	2.46E-06	PSMA5
6866	212339_at	1.632803704	5.25E-11	EPB41L1
6867	206597_at	1.633103704	3.74E-06	NRL
6868	37590_g_at	1.63342963	2.78E-09	AK055981 /// RP11-617F23.1
6869	224460_s_at	1.633562963	1.57E-08	L2HGDH
6870	1552477_a_at	1.633751852	1.76E-06	IRF6
6871	230717_at	1.634103704	5.90E-08	LCN12
6872	219429_at	1.634718519	2.35E-06	FA2H
6873	242343_x_at	1.635285185	6.41E-07	
6874	205257_s_at	1.636081481	7.01E-07	AMPH
6875	213927_at	1.636203704	2.37E-14	MAP3K9
6876	232565_at	1.636940741	5.30E-08	
6877	225457_s_at	1.637148148	1.92E-09	LINC00263 /// PP7080

6878	1553130_at	1.637166667	4.52E-06	LOC652276
6879	224367_at	1.638918519	1.48E-06	BEX2
6880	213587_s_at	1.638959259	1.34E-11	ATP6V0E2
6881	237328_at	1.639922222	5.72E-07	C14orf105
6882	228448_at	1.639955556	3.06E-07	MAP6
6883	225746_at	1.641385185	1.50E-07	RAB11FIP4
6884	210247_at	1.641485185	1.85E-08	SYN2
6885	236752_at	1.642681481	3.91E-09	
6886	242920_at	1.643651852	9.45E-11	
6887	221042_s_at	1.644207407	2.97E-07	CLMN
6888	222302_at	1.645040741	1.64E-06	RP11-82L18.2
6889	233611_at	1.64527037	1.88E-06	
6890	240322_at	1.645374074	3.06E-06	
6891	1553396_a_at	1.645388889	2.51E-07	CCDC13
6892	240873_x_at	1.645585185	3.73E-08	DAB2 /// LOC101926921
6893	211417_x_at	1.645703704	2.25E-06	GGT1 /// GGT2 /// GGTLC1 /// GGTLC2
6894	239089_at	1.646262963	3.84E-08	
6895	207344_at	1.649144444	3.55E-07	AKAP3
6896	241613_at	1.651507407	1.24E-07	
6897	244310_at	1.651814815	6.47E-08	
6898	243184_at	1.652022222	2.47E-06	
6899	219132_at	1.653981481	2.07E-09	PELI2
6900	242404_at	1.653988889	9.14E-08	GNB5
6901	220948_s_at	1.654418519	8.37E-18	ATP1A1
6902	235672_at	1.65547037	1.53E-08	MAP6
6903	223319_at	1.65562963	7.58E-14	GPHN
6904	243980_at	1.656455556	2.57E-06	ZNF594
6905	219820_at	1.656514815	5.56E-07	SLC6A16
6906	206492_at	1.656933333	1.92E-16	FHIT
6907	225775_at	1.657714815	1.08E-10	TSPAN33
6908	215423_at	1.658207407	6.33E-07	6-Mar
6909	213417_at	1.65837037	3.53E-06	TBX2
6910	205293_x_at	1.660066667	5.55E-06	BAIAP2
6911	202295_s_at	1.660081481	4.18E-14	CTSH
6912	226649_at	1.660081481	1.29E-08	PANK1
6913	1562920_at	1.660274074	8.26E-09	LOC441086
6914	207131_x_at	1.660514815	5.09E-06	GGT1 /// GGT2 /// GGTLC1 /// GGTLC2
6915	244771_at	1.661651852	1.63E-06	KBTBD12
6916	244222_at	1.661837037	2.22E-06	
6917	226974_at	1.663122222	4.49E-11	NEDD4L
6918	207110_at	1.664848148	2.75E-06	KCNJ12 /// KCNJ18 /// LOC100996843
6919	213260_at	1.665251852	3.13E-10	FOXC1
6920	231796_at	1.665418519	1.97E-08	EPHA8

6921	220819_at	1.665777778	6.70E-07	FRMD1
6922	237028_at	1.667107407	3.47E-06	ENO1-AS1
6923	213874_at	1.667640741	8.27E-07	SERPINA4
6924	232552_at	1.668003704	8.58E-06	DAAM1
6925	235501_at	1.668707407	3.65E-08	
6926	212703_at	1.669414815	1.65E-11	TLN2
6927	240151_at	1.670674074	7.85E-06	HOXB-AS3
6928	228469_at	1.670885185	3.96E-09	
6929	1556331_a_at	1.672559259	3.40E-06	
6930	1562059_at	1.673314815	1.65E-06	
6931	205554_s_at	1.673359259	1.07E-06	DNASE1L3
6932	230630_at	1.674122222	6.44E-08	AK4 /// LOC100507855
6933	212925_at	1.674503704	3.60E-08	MISP
6934	219534_x_at	1.677040741	2.56E-11	CDKN1C
6935	213181_s_at	1.678477778	9.57E-08	MOCS1
6936	231555_at	1.678848148	5.52E-07	
6937	223597_at	1.679196296	1.60E-06	ITLN1
6938	237263_at	1.679262963	6.86E-09	
6939	1567255_at	1.680014815	2.36E-07	OR10D1P
6940	229657_at	1.680796296	7.19E-11	THRB
6941	231644_at	1.681225926	1.15E-07	
6942	235920_at	1.682292593	1.49E-08	CLUHP3 /// ZNF720
6943	204720_s_at	1.682340741	7.27E-10	DNAJC6
6944	229654_at	1.68237037	2.53E-07	
6945	220234_at	1.682688889	6.76E-06	CA8
6946	221990_at	1.682792593	4.33E-08	PAX8
6947	220420_at	1.683325926	1.16E-07	LMAN1L
6948	241782_at	1.683385185	1.80E-08	NEBL
6949	216894_x_at	1.684907407	8.50E-11	CDKN1C
6950	225379_at	1.68517037	7.15E-06	MAPT
6951	232083_at	1.685285185	3.27E-13	KIF16B
6952	211303_x_at	1.685303704	1.02E-06	FOLH1B
6953	206600_s_at	1.685514815	5.99E-08	SLC16A5
6954	203908_at	1.685759259	3.98E-06	SLC4A4
6955	220543_at	1.685818519	3.73E-06	C21orf62
6956	221217_s_at	1.686214815	4.54E-06	RBFOX1
6957	203560_at	1.686759259	4.04E-10	GGH
6958	215304_at	1.687655556	5.26E-07	
6959	238830_at	1.688022222	7.20E-07	SOS2
6960	235251_at	1.688651852	3.62E-07	DQ592230 /// RP4-555D20.2
6961	215603_x_at	1.688844444	7.35E-06	GGT1 /// GGT2 /// GGT3P /// GGTLC1 /// GGTLC2 /// LOC100132705 /// LOC102724197
6962	238786_at	1.689818519	3.70E-06	ANK3

6963	239557_at	1.691185185	2.95E-07	
6964	226988_s_at	1.691581481	2.31E-07	MYH14
6965	227094_at	1.691644444	1.61E-11	DHTKD1
6966	231008_at	1.691837037	6.89E-07	UNC5CL
6967	228831_s_at	1.6926	5.09E-13	GNG7
6968	230488_s_at	1.692859259	1.30E-08	DBH-AS1
6969	214343_s_at	1.692940741	4.62E-06	ATXN7L1
6970	223636_at	1.693385185	2.50E-07	ZMYND12
6971	223599_at	1.69447037	3.98E-07	TRIM6
6972	221256_s_at	1.694496296	3.61E-13	HDHD3
6973	220611_at	1.695218519	1.18E-06	DAB1
6974	229513_at	1.695222222	5.60E-08	STRBP
6975	231136_at	1.695607407	8.95E-10	
6976	215506_s_at	1.696733333	1.27E-09	DIRAS3
6977	229866_at	1.697218519	4.70E-07	STK32A
6978	229824_at	1.699974074	4.24E-07	SHC3
6979	235512_at	1.701296296	3.85E-08	CDKL1
6980	230624_at	1.701503704	5.35E-09	SLC25A27
6981	221948_s_at	1.701692593	1.44E-07	KLHL22
6982	208869_s_at	1.702533333	3.09E-15	GABARAPL1
6983	235664_at	1.703296296	3.04E-06	
6984	220654_at	1.703574074	8.94E-06	PPY2
6985	215912_at	1.704585185	1.70E-06	GNAO1
6986	205372_at	1.705196296	6.43E-09	PLAG1
6987	229616_s_at	1.705959259	3.87E-08	GRAMD2
6988	205998_x_at	1.706103704	1.24E-06	CYP3A4
6989	37996_s_at	1.707122222	5.39E-13	DMPK
6990	230412_at	1.707296296	3.24E-06	NPAS3
6991	218021_at	1.707362963	1.51E-09	DHRS4 /// DHRS4L2
6992	241218_at	1.707522222	3.51E-08	
6993	215578_at	1.708307407	4.90E-06	
6994	242003_at	1.709151852	2.87E-06	ERICH1
6995	214014_at	1.709577778	4.00E-06	CDC42EP2
6996	203854_at	1.709692593	4.33E-06	CFI
6997	228214_at	1.710381481	1.74E-07	SOX6
6998	244833_at	1.711259259	3.38E-06	CCDC63
6999	221223_x_at	1.711266667	1.21E-12	CISH
7000	1553062_at	1.712214815	2.61E-07	MOGAT1
7001	1554060_s_at	1.71402963	1.70E-06	SETMAR
7002	214238_at	1.714048148	1.17E-07	AC012065.7
7003	230763_at	1.714485185	2.25E-10	SPATA17
7004	1559020_a_at	1.714844444	7.13E-06	
7005	227657_at	1.715192593	1.25E-11	RNF150

7006	209980_s_at	1.715303704	3.12E-07	MIR6778 /// SHMT1
7007	205919_at	1.7156	1.51E-06	HBE1
7008	230611_at	1.716133333	6.30E-13	SYPL2
7009	234298_at	1.716377778	4.21E-06	
7010	1566690_at	1.716525926	2.05E-07	
7011	229667_s_at	1.717588889	1.70E-07	HOXB8
7012	240710_at	1.717633333	3.32E-06	
7013	241542_at	1.717714815	9.03E-07	
7014	230309_at	1.718177778	1.44E-08	BHMT2
7015	213547_at	1.718192593	3.95E-12	CAND2
7016	240640_at	1.718448148	3.90E-06	
7017	240934_at	1.718855556	3.56E-08	
7018	1561121_at	1.718933333	8.36E-06	
7019	241640_at	1.719596296	6.26E-09	BCAP29
7020	1556221_a_at	1.721181481	4.16E-07	RP11-757F18.5
7021	215785_s_at	1.721344444	4.99E-11	CYFIP2
7022	244231_at	1.721348148	1.43E-10	BPI /// LOC149684
7023	239702_x_at	1.721874074	2.02E-07	
7024	206599_at	1.722140741	1.50E-06	SLC16A5
7025	1570035_at	1.7235	3.74E-06	TBC1D10A
7026	242775_at	1.723514815	5.27E-13	
7027	205317_s_at	1.724488889	7.98E-13	SLC15A2
7028	1556797_at	1.725722222	2.07E-06	RNF144A-AS1
7029	230074_s_at	1.726633333	7.38E-10	C16orf58
7030	1565834_a_at	1.7276	1.12E-06	
7031	223427_s_at	1.727822222	6.36E-07	EPB41L4B
7032	238673_at	1.728651852	9.47E-06	SAMD12
7033	1565598_at	1.728737037	2.45E-10	
7034	205500_at	1.72902963	8.59E-15	C5
7035	1555834_at	1.729544444	1.82E-06	UCHL1
7036	240263_at	1.730155556	1.95E-09	
7037	233273_at	1.730492593	4.11E-07	RP11-506O24.2
7038	1558053_s_at	1.732137037	9.32E-08	TMED4
7039	237377_at	1.733225926	8.76E-10	
7040	1556160_a_at	1.733951852	4.58E-09	
7041	213917_at	1.734048148	2.72E-08	PAX8
7042	231073_at	1.734111111	1.04E-07	C1orf168
7043	220024_s_at	1.734618519	6.30E-07	PRX
7044	214307_at	1.734877778	3.59E-06	HGD
7045	238450_at	1.735244444	9.38E-07	PFKFB2
7046	205442_at	1.735348148	8.64E-07	LOC101928198 /// MFAP3L
7047	44696_at	1.736248148	1.49E-15	TBC1D13
7048	233964_at	1.736907407	1.42E-07	

7049	226737_at	1.736981481	2.70E-08	SLC25A42
7050	237044_s_at	1.737837037	4.15E-10	ZNF503-AS2
7051	201982_s_at	1.737988889	2.18E-10	PAPPA
7052	237925_at	1.738366667	1.62E-06	
7053	202851_at	1.739037037	2.99E-06	AAGAB
7054	210728_s_at	1.739111111	2.53E-07	CALCA
7055	217680_x_at	1.740292593	3.75E-07	RPL10 /// SNORA70
7056	209645_s_at	1.740933333	3.93E-09	ALDH1B1
7057	201695_s_at	1.741374074	6.90E-15	PNP
7058	1554805_at	1.741914815	1.51E-08	CLDN19
7059	229095_s_at	1.742066667	1.23E-06	LIMS3-LOC440895 /// LOC100288570 /// LOC100507334 /// LOC440895
7060	230551_at	1.742196296	7.35E-09	KSR2
7061	213590_at	1.743381481	2.29E-09	SLC16A5
7062	236315_at	1.743940741	7.12E-06	XXyac-YX155B6.7
7063	235663_at	1.744722222	8.50E-07	RP11-465B22.8
7064	206827_s_at	1.744788889	2.62E-06	TRPV6
7065	236534_at	1.745225926	1.90E-06	BNIPL /// C1orf56
7066	222068_s_at	1.745444444	4.09E-06	DNAAF1
7067	205412_at	1.747037037	1.95E-11	ACAT1
7068	222357_at	1.748481481	1.02E-09	ZBTB20
7069	243014_at	1.749144444	5.07E-06	
7070	220365_at	1.749166667	6.30E-08	ALLC
7071	204731_at	1.749403704	1.94E-09	TGFBR3
7072	219442_at	1.7495	2.56E-09	CLUHP3
7073	221810_at	1.749548148	7.64E-06	RAB15
7074	244313_at	1.749907407	2.14E-06	CR1
7075	239752_at	1.750948148	8.30E-07	CECR2
7076	204519_s_at	1.7521	5.58E-08	PLLP
7077	213050_at	1.7521	5.31E-06	COBL
7078	209916_at	1.752740741	3.68E-09	DHTKD1
7079	242626_at	1.753362963	5.35E-06	SAMD5
7080	1562828_at	1.753714815	5.05E-07	
7081	211715_s_at	1.753914815	1.45E-07	BDH1
7082	213247_at	1.754462963	8.43E-06	SVEP1
7083	214248_s_at	1.756085185	1.15E-06	TRIM2
7084	238105_x_at	1.756448148	1.46E-07	WNT7B
7085	205673_s_at	1.756522222	6.25E-12	ASB9
7086	229518_at	1.757688889	2.90E-06	FAM46B
7087	242443_at	1.757744444	1.15E-08	ZC3H14
7088	243586_at	1.757759259	2.65E-07	
7089	204364_s_at	1.757859259	1.03E-08	REEP1
7090	228836_at	1.758381481	1.49E-07	SLC25A35

7091	242846_at	1.758644444	8.70E-06	
7092	232275_s_at	1.759303704	4.18E-06	HS6ST3
7093	1555723_at	1.76112963	2.46E-07	
7094	229940_at	1.761240741	2.61E-07	SETD3
7095	243713_at	1.761607407	2.28E-07	
7096	242658_at	1.761762963	7.35E-09	
7097	243216_x_at	1.763503704	1.91E-08	
7098	1563104_at	1.764362963	3.27E-09	
7099	224048_at	1.76517037	5.41E-08	USP44
7100	205522_at	1.765244444	7.14E-13	
7101	229205_at	1.7665	1.15E-06	LOC101927720
7102	207463_x_at	1.7666	3.71E-06	PRSS3
7103	1569354_at	1.766774074	1.69E-06	
7104	241229_at	1.767444444	1.77E-06	
7105	226411_at	1.767966667	1.24E-09	EVI5L
7106	239248_at	1.768203704	1.36E-06	SDCBP2-AS1
7107	204213_at	1.768659259	1.46E-07	PIGR
7108	228684_at	1.768803704	6.46E-07	ZNF503
7109	232512_at	1.769807407	2.03E-07	AC013463.2
7110	1568617_a_at	1.770292593	1.48E-07	CAMSAP3
7111	220324_at	1.770733333	1.70E-11	LINC00472
7112	1559977_a_at	1.770788889	2.77E-09	SLC25A34
7113	218756_s_at	1.770981481	1.77E-13	DHRS11
7114	234974_at	1.772255556	2.32E-14	GALM
7115	1555923_a_at	1.772855556	3.64E-09	CASC10
7116	218546_at	1.772937037	2.74E-10	C1orf115
7117	226424_at	1.773062963	6.47E-11	CAPS
7118	210074_at	1.77377037	4.46E-13	CTSV
7119	226770_at	1.773777778	1.46E-14	MAGI3
7120	224998_at	1.773996296	1.48E-14	CMTM4
7121	202986_at	1.775837037	5.41E-07	ARNT2
7122	1560512_at	1.778340741	7.54E-08	
7123	219902_at	1.77837037	1.91E-08	BHMT2
7124	229948_at	1.780348148	2.66E-16	
7125	225009_at	1.78092963	1.48E-15	CMTM4
7126	241154_x_at	1.781351852	1.65E-07	
7127	219916_s_at	1.781462963	5.69E-07	RNF39
7128	229012_at	1.783737037	4.42E-06	C9orf24
7129	227721_at	1.785703704	4.48E-10	CPAMD8
7130	228583_at	1.786155556	3.10E-14	LIN52
7131	236131_at	1.786685185	7.00E-07	RP11-2E11.9
7132	239806_at	1.787555556	1.06E-06	
7133	244797_at	1.789914815	1.29E-06	LINC01159

7134	235334_at	1.790640741	2.22E-09	ST6GALNAC3
7135	209985_s_at	1.791248148	3.48E-06	ASCL1
7136	236960_at	1.79147037	1.27E-06	
7137	227962_at	1.791551852	5.43E-16	ACOX1
7138	204311_at	1.792277778	5.46E-08	ATP1B2
7139	204253_s_at	1.793051852	1.52E-08	VDR
7140	229170_s_at	1.79327037	3.12E-06	TTC18
7141	229952_at	1.794322222	1.38E-08	PLEKHG3
7142	224324_at	1.794585185	2.90E-06	MRO
7143	241302_at	1.795440741	2.93E-06	
7144	1553613_s_at	1.796937037	3.05E-09	FOXC1
7145	212448_at	1.797418519	4.77E-10	NEDD4L
7146	236586_at	1.79782963	1.66E-09	
7147	235256_s_at	1.79807037	9.17E-13	GALM
7148	227908_at	1.798666667	6.34E-15	TBC1D24
7149	206085_s_at	1.799611111	5.87E-10	CTH
7150	223961_s_at	1.799674074	1.30E-11	CISH
7151	1561624_at	1.800103704	6.39E-08	LOC101927537
7152	205760_s_at	1.800440741	3.02E-12	OGG1
7153	1552713_a_at	1.801992593	5.23E-09	SLC4A1
7154	1559266_s_at	1.802322222	7.83E-10	SKIDA1
7155	210331_at	1.803225926	1.15E-09	HECW1
7156	239738_at	1.807162963	2.72E-09	DACH2
7157	206794_at	1.807344444	3.94E-09	ERBB4
7158	1557126_a_at	1.807559259	3.42E-08	PLD1
7159	242058_at	1.808403704	2.92E-09	
7160	211432_s_at	1.808411111	1.09E-09	TYRO3
7161	235924_at	1.808977778	1.23E-11	FRK
7162	213695_at	1.809122222	1.05E-07	PON3
7163	241368_at	1.8094	4.18E-12	PLIN5
7164	208284_x_at	1.810192593	6.80E-07	GGT1 /// GGT2 /// GGTLC1 /// GGTLC2
7165	227182_at	1.810277778	6.15E-10	SUSD3
7166	229851_s_at	1.812562963	2.13E-07	C11orf54
7167	228237_at	1.813377778	6.29E-07	PAPPA2
7168	229242_at	1.815559259	7.50E-11	TNFSF15
7169	1554006_a_at	1.815955556	3.03E-09	LLGL2
7170	210414_at	1.816188889	8.92E-08	FLRT1
7171	214623_at	1.816607407	6.10E-07	FBXW4P1
7172	213374_x_at	1.819544444	1.87E-14	HIBCH
7173	1559949_at	1.822074074	4.18E-07	
7174	201525_at	1.823062963	1.85E-06	APOD
7175	233275_at	1.823292593	1.66E-07	
7176	224006_at	1.823414815	1.44E-06	LOC100507377

7177	243974_at	1.8237	9.22E-07	
7178	213658_at	1.824711111	2.58E-11	AK055981
7179	239242_at	1.824785185	1.50E-12	SLC25A5-AS1
7180	235355_at	1.824903704	8.98E-08	CSRNP3
7181	210082_at	1.826055556	1.14E-07	ABCA4
7182	1563340_at	1.8282	4.01E-07	CTD-3193O13.1
7183	237397_at	1.828766667	4.79E-08	
7184	1562844_at	1.828833333	6.79E-06	LINC01115
7185	224839_s_at	1.828914815	3.04E-08	GPT2
7186	1557879_at	1.828966667	1.93E-06	LOC100129175
7187	204743_at	1.829659259	1.39E-08	TAGLN3
7188	238463_at	1.829733333	3.79E-06	LOC100506834
7189	219313_at	1.830011111	5.01E-07	GRAMD1C
7190	226487_at	1.830459259	1.61E-14	FAM222A
7191	208100_x_at	1.831207407	1.75E-07	SEMA6C
7192	230991_at	1.831640741	1.55E-06	LOC102724156
7193	233634_at	1.831955556	5.39E-06	MARVELD3
7194	218704_at	1.832155556	5.59E-09	RNF43
7195	232144_at	1.832159259	1.92E-09	
7196	227417_at	1.832733333	4.99E-09	2-Mar
7197	242011_at	1.833985185	3.94E-07	
7198	1553844_a_at	1.834118519	1.74E-06	C10orf67
7199	238910_at	1.834922222	4.95E-07	CLUHP3
7200	203814_s_at	1.835040741	3.86E-14	NQO2
7201	39248_at	1.835133333	1.85E-06	AQP3
7202	1569001_at	1.835959259	1.82E-06	BMP1
7203	211348_s_at	1.837081481	1.59E-08	CDC14B
7204	211569_s_at	1.837977778	9.75E-17	HADH
7205	1563277_at	1.838662963	4.92E-06	
7206	57539_at	1.839877778	5.93E-11	LIME1 /// ZGPAT
7207	212859_x_at	1.840303704	4.64E-07	MT1E
7208	219054_at	1.841392593	1.18E-06	NPR3
7209	210945_at	1.842162963	3.13E-06	COL4A6
7210	203215_s_at	1.842759259	3.88E-11	MYO6
7211	220753_s_at	1.842807407	1.88E-08	CRYL1
7212	235658_at	1.843544444	1.17E-09	
7213	205845_at	1.844396296	7.48E-06	CACNA1H
7214	1563635_at	1.844551852	6.44E-06	ITGA9-AS1
7215	209975_at	1.8447	1.88E-06	CYP2E1
7216	203397_s_at	1.844840741	1.21E-07	GALNT3
7217	202341_s_at	1.844944444	1.73E-10	TRIM2
7218	203009_at	1.8466	5.48E-11	BCAM
7219	1561595_x_at	1.847255556	3.58E-06	OR7D2

7220	1556764_s_at	1.847674074	1.67E-08	
7221	238428_at	1.847892593	9.32E-07	KCNJ15
7222	214829_at	1.848703704	7.05E-08	AASS
7223	39249_at	1.84887037	6.71E-09	AQP3
7224	240382_at	1.849592593	4.21E-06	LOC101928076
7225	215472_at	1.849892593	3.19E-12	PACRG
7226	204326_x_at	1.850737037	2.38E-07	MT1X
7227	215966_x_at	1.851644444	8.51E-10	GK3P
7228	210328_at	1.852255556	3.01E-06	GNMT
7229	229292_at	1.852737037	3.77E-08	EPB41L5
7230	244627_at	1.8536	2.06E-13	DAK
7231	214147_at	1.855122222	2.31E-06	MROH7
7232	236081_at	1.855781481	5.84E-07	SNCA
7233	210751_s_at	1.856625926	1.88E-09	RGN
7234	240587_x_at	1.857862963	1.29E-11	
7235	227336_at	1.858022222	1.34E-07	DTX1
7236	1563963_at	1.858537037	1.75E-06	
7237	229620_at	1.86012963	1.69E-06	SEPP1
7238	1554618_at	1.860392593	3.10E-06	AGFG2
7239	242280_x_at	1.860681481	1.57E-10	CPEB4
7240	1554559_at	1.862362963	3.14E-08	GPR62
7241	204254_s_at	1.864337037	3.92E-11	VDR
7242	244740_at	1.865103704	5.98E-11	
7243	1552827_s_at	1.865722222	2.93E-09	SLC26A7
7244	229169_at	1.866851852	8.98E-07	TTC18
7245	203911_at	1.869325926	4.61E-10	RAP1GAP
7246	223754_at	1.870222222	1.18E-06	C2orf88
7247	218931_at	1.870396296	1.30E-06	RAB17
7248	228241_at	1.872188889	8.94E-07	AGR3
7249	204255_s_at	1.873122222	3.07E-12	VDR
7250	220331_at	1.873937037	2.47E-06	CYP46A1
7251	219671_at	1.873966667	7.17E-09	HPCAL4
7252	219144_at	1.874685185	1.17E-11	DUSP26
7253	236612_at	1.875381481	8.79E-07	
7254	238766_at	1.87672963	6.83E-08	
7255	216898_s_at	1.877744444	1.68E-10	COL4A3
7256	243435_at	1.877992593	7.61E-07	KCNQ1OT1 /// LOC101927338
7257	207823_s_at	1.878811111	9.77E-07	AIF1
7258	207950_s_at	1.881622222	1.94E-07	ANK3
7259	230402_at	1.882066667	1.13E-10	DUSP15
7260	205355_at	1.882451852	8.52E-14	ACADSB
7261	232156_at	1.883459259	5.13E-19	AP5M1
7262	238805_at	1.88352963	2.02E-09	C11orf52 /// HSPB2 /// HSPB2- C11orf52

7263	210377_at	1.883803704	3.63E-10	ACSM3
7264	40093_at	1.883803704	1.30E-16	BCAM
7265	231790_at	1.885577778	2.34E-08	DMGDH
7266	216929_x_at	1.885725926	2.30E-07	ABO
7267	233765_at	1.887477778	7.35E-07	
7268	1565660_at	1.88792963	1.99E-11	FUT6
7269	205221_at	1.887955556	1.72E-07	HGD
7270	214798_at	1.888385185	2.11E-07	ATP2C2
7271	203178_at	1.889003704	1.13E-08	GATM
7272	1562412_at	1.88902963	6.07E-06	
7273	226766_at	1.889055556	2.11E-06	ROBO2
7274	226226_at	1.8891	1.61E-07	TMEM45B
7275	221884_at	1.889203704	1.87E-11	MECOM
7276	229096_at	1.8894	5.26E-06	LIMS3-LOC440895 /// LOC100288570 /// LOC100507334 /// LOC440895
7277	205614_x_at	1.890196296	3.87E-08	MST1
7278	215265_at	1.890618519	1.46E-06	
7279	217623_at	1.891111111	7.71E-07	MYLK3
7280	1564358_at	1.891940741	4.01E-06	
7281	232929_at	1.892248148	2.07E-07	ZBTB20
7282	240318_at	1.892633333	1.58E-06	AFMID
7283	203860_at	1.892785185	5.28E-10	PCCA
7284	236608_at	1.893248148	1.44E-06	GPR113
7285	232784_at	1.895488889	8.04E-08	
7286	211682_x_at	1.896033333	1.62E-07	UGT2B28
7287	229887_at	1.896285185	5.67E-15	ALS2CL
7288	211821_x_at	1.896796296	9.83E-06	GYPA
7289	216495_x_at	1.898818519	4.47E-08	
7290	1555929_s_at	1.898940741	2.25E-06	
7291	229779_at	1.900103704	1.08E-08	COL4A4
7292	207057_at	1.900322222	1.18E-09	SLC16A7
7293	207958_at	1.900692593	7.67E-07	UGT2A1 /// UGT2A2
7294	240827_at	1.901281481	2.40E-08	GATA3-AS1
7295	230096_at	1.901414815	5.10E-10	SAPCD1-AS1 /// SAPCD1-AS1 /// XXbac-BPG32J3.18
7296	1556375_at	1.901659259	2.02E-06	
7297	236205_at	1.902566667	2.91E-06	ABCC6P1
7298	206768_at	1.90272963	1.59E-06	RPL3L
7299	232549_at	1.902785185	1.21E-06	RBM11
7300	236617_at	1.90357037	2.34E-07	
7301	1558728_at	1.903588889	5.64E-08	VPS9D1-AS1
7302	237798_at	1.903707407	5.56E-07	

7303	1553730_x_at	1.904474074	5.71E-07	LRRC43
7304	237087_at	1.906022222	5.03E-06	
7305	216336_x_at	1.906051852	7.24E-08	MT1E
7306	238003_at	1.907462963	9.68E-07	HEPACAM /// HEPN1
7307	232000_at	1.907574074	1.04E-11	TTC39B
7308	231047_at	1.907714815	1.68E-14	
7309	1556583_a_at	1.910159259	8.34E-10	SLC8A1
7310	213657_s_at	1.910718519	6.62E-11	AK055981 /// RP11-617F23.1
7311	232430_at	1.910903704	1.96E-06	LOC148696
7312	229598_at	1.913885185	1.73E-09	COBLL1
7313	244753_at	1.914833333	6.36E-10	
7314	233691_at	1.915407407	2.19E-06	
7315	221636_s_at	1.916240741	1.77E-09	2-Mar
7316	205848_at	1.9164	3.90E-06	GAS2
7317	228802_at	1.918251852	4.56E-08	RBPMS2
7318	231701_s_at	1.918918519	5.56E-08	SHMT1
7319	228825_at	1.921144444	7.12E-14	PTGR1
7320	217700_at	1.921307407	6.54E-06	CNPY4
7321	228192_at	1.921533333	2.70E-07	MIR3934 /// UQCC2
7322	204067_at	1.922651852	1.37E-16	SUOX
7323	227875_at	1.92297037	3.42E-11	KLHL13
7324	239955_at	1.923540741	1.06E-08	
7325	231214_at	1.924155556	2.21E-06	
7326	239929_at	1.924222222	6.64E-08	PM20D1
7327	216320_x_at	1.924240741	9.29E-09	MST1
7328	239683_at	1.924348148	1.21E-12	CLYBL
7329	230641_at	1.9247	9.46E-06	LOC100505938
7330	237413_at	1.925177778	1.38E-07	MAPK10
7331	226611_s_at	1.9252	3.19E-08	CENPV
7332	235771_at	1.926448148	1.64E-14	LINC00472
7333	210739_x_at	1.926833333	1.38E-09	SLC4A4
7334	211032_at	1.927022222	1.24E-06	COBLL1
7335	212906_at	1.930114815	6.10E-11	GRAMD1B
7336	221848_at	1.930755556	1.07E-10	ZGPAT
7337	225212_at	1.931059259	3.78E-13	SLC25A25
7338	222484_s_at	1.932311111	2.54E-06	CXCL14
7339	244547_at	1.933774074	9.97E-09	SGK494
7340	207415_at	1.936525926	1.76E-08	PLA2R1
7341	242384_at	1.936544444	7.53E-09	
7342	216103_at	1.936566667	5.51E-10	ACOT11
7343	239470_at	1.9366	9.50E-06	C15orf56
7344	233478_at	1.937151852	4.91E-07	
7345	231014_at	1.938211111	1.67E-07	TRIM50

7346	206556_at	1.939922222	4.01E-08	CLUL1
7347	235383_at	1.940566667	3.03E-06	MYO7B
7348	227996_at	1.941059259	3.21E-09	FARP1
7349	215787_at	1.942733333	1.48E-06	ACTA2
7350	226145_s_at	1.94317037	1.08E-06	FRAS1
7351	240180_at	1.943340741	1.48E-07	
7352	229725_at	1.943848148	1.75E-08	ACSL6
7353	33767_at	1.94392963	2.94E-08	NEFH
7354	204294_at	1.944866667	1.84E-12	AMT
7355	235871_at	1.945485185	5.50E-07	LIPH
7356	244056_at	1.945933333	4.64E-06	SFTA2
7357	1556199_a_at	1.946340741	2.55E-06	RGS9BP
7358	203859_s_at	1.948333333	9.65E-14	PALM
7359	220149_at	1.949651852	2.10E-06	C2orf54
7360	1565337_at	1.950155556	3.05E-06	DNAH6
7361	239987_at	1.950155556	5.36E-06	
7362	224870_at	1.950251852	2.63E-12	DANCR
7363	211203_s_at	1.952748148	5.71E-06	CNTN1
7364	230008_at	1.952981481	2.49E-09	THSD7A
7365	223233_s_at	1.954074074	7.60E-10	CGN
7366	236064_at	1.954885185	1.12E-08	SLC25A35
7367	232995_at	1.955181481	1.59E-06	
7368	232027_at	1.955751852	3.56E-06	SYNE1
7369	206204_at	1.95627037	1.28E-06	GRB14
7370	215943_at	1.956737037	1.30E-06	KIAA1661
7371	208142_at	1.956859259	9.83E-07	EDDM3A
7372	1570031_at	1.957048148	2.68E-06	
7373	223823_at	1.957359259	4.33E-06	KCNMB2
7374	1563331_at	1.958925926	5.01E-06	
7375	218523_at	1.95937037	1.20E-14	LHPP
7376	211494_s_at	1.959381481	5.23E-08	SLC4A4
7377	207914_x_at	1.960988889	8.80E-12	EVX1
7378	235979_at	1.961118519	1.91E-06	C7
7379	213182_x_at	1.961611111	5.57E-10	CDKN1C
7380	219046_s_at	1.963351852	4.10E-08	PKNOX2
7381	234413_at	1.963903704	3.68E-06	RP4-633H17.2
7382	229155_at	1.964674074	2.27E-10	GSTO2
7383	227554_at	1.965985185	6.04E-11	MAGI2-AS3
7384	213486_at	1.967533333	4.79E-14	COPG2IT1
7385	229526_at	1.968925926	1.51E-08	AQP11
7386	217551_at	1.969322222	2.80E-09	OR7E14P
7387	211538_s_at	1.969325926	8.99E-08	HSPA2
7388	207705_s_at	1.9696	2.87E-14	NINL

7389	219702_at	1.969807407	9.99E-08	PLAC1
7390	217127_at	1.976077778	6.88E-11	CTH
7391	1558281_a_at	1.976555556	4.50E-06	TMEM184A
7392	207766_at	1.978062963	4.06E-06	CDKL1
7393	238312_s_at	1.979040741	3.77E-06	B4GALT3
7394	212701_at	1.979114815	1.16E-12	TLN2
7395	224323_s_at	1.979277778	8.07E-11	MRO
7396	205939_at	1.98017037	1.53E-06	CYP3A7 /// CYP3A7-CYP3AP1
7397	37201_at	1.980692593	2.02E-07	ITIH4
7398	226587_at	1.98137037	1.99E-12	PWAR6
7399	236085_at	1.981522222	7.11E-06	CAPSL
7400	228796_at	1.981548148	3.53E-08	CPNE4
7401	204542_at	1.982714815	3.48E-07	ST6GALNAC2
7402	1556136_at	1.982759259	4.33E-06	MYLK4
7403	235937_at	1.983496296	9.43E-06	OCLN
7404	229480_at	1.983674074	2.50E-09	MAGI2-AS3
7405	204740_at	1.984648148	3.40E-17	CNKSR1
7406	239606_at	1.986511111	2.18E-08	
7407	231310_at	1.986996296	5.95E-08	TRIM71
7408	243127_x_at	1.988062963	1.25E-06	DNASE1
7409	1554491_a_at	1.988896296	8.26E-07	SERPINC1
7410	232127_at	1.988925926	6.65E-10	CLCN5
7411	215977_x_at	1.990344444	3.61E-10	GK
7412	1556508_s_at	1.991707407	5.62E-06	LINC01210
7413	207789_s_at	1.991707407	5.50E-10	DPP6
7414	223268_at	1.991777778	3.97E-08	C11orf54
7415	212686_at	1.992281481	2.17E-15	PPM1H
7416	1563022_at	1.992366667	3.78E-06	CCDC160
7417	241418_at	1.992718519	1.26E-08	LOC344887
7418	218285_s_at	1.992837037	3.94E-12	BDH2
7419	219876_s_at	1.993844444	1.43E-06	GOLGA2P5
7420	222939_s_at	1.994155556	7.83E-11	SLC16A10
7421	238266_at	1.994507407	1.08E-08	RP11-433A10.3
7422	220354_at	1.994614815	3.61E-07	MCF2L-AS1
7423	228716_at	1.994625926	7.48E-11	THRB
7424	207015_s_at	1.996081481	1.35E-06	ALDH1A2
7425	221084_at	1.996992593	5.50E-07	HTR3B
7426	231084_at	1.9991	5.04E-06	WDR96
7427	1564017_at	2.000666667	1.16E-06	COL18A1-AS1
7428	218596_at	2.00177037	1.26E-10	TBC1D13
7429	231166_at	2.002133333	7.66E-12	GPR155
7430	209605_at	2.0026	2.35E-11	TST
7431	240811_at	2.002714815	1.31E-06	

7432	1552657_a_at	2.004937037	2.80E-06	TXNDC2
7433	240284_x_at	2.006111111	2.59E-07	U47924.27
7434	205980_s_at	2.006444444	2.97E-06	ARHGAP8 /// PRR5-ARHGAP8
7435	AFFX-	2.00687037	3.89E-07	
7436	1560089_at	2.007033333	3.95E-13	LOC100289019
7437	1560305_x_at	2.007140741	2.18E-07	FKBP4
7438	210834_s_at	2.007874074	1.78E-08	PTGER3
7439	235316_at	2.007903704	4.24E-08	NAT8L
7440	213834_at	2.00802963	1.81E-06	IQSEC3
7441	215005_at	2.008048148	3.88E-06	NECAB2
7442	205342_s_at	2.009996296	4.20E-07	SULT1C2
7443	226420_at	2.010003704	5.92E-12	MECOM
7444	238862_at	2.010359259	1.47E-08	MFSD4
7445	240380_at	2.011303704	2.08E-08	LOC728040
7446	204687_at	2.012374074	8.23E-15	PARM1
7447	220999_s_at	2.01262963	3.37E-11	CYFIP2
7448	205043_at	2.012866667	6.32E-06	CFTR
7449	226096_at	2.013259259	2.16E-06	FNDC5
7450	1565329_at	2.013688889	7.20E-07	POLE4
7451	237869_at	2.014677778	1.27E-06	GGACT
7452	228503_at	2.015096296	1.59E-09	RPS6KA6
7453	226614_s_at	2.015151852	1.48E-13	FAM167A
7454	1558577_at	2.017811111	2.28E-06	LOC148709
7455	231686_at	2.018940741	1.47E-07	GATM
7456	219188_s_at	2.019074074	2.38E-14	MACROD1
7457	220270_at	2.020459259	2.86E-07	RNF17
7458	231140_at	2.020651852	2.01E-07	TPPP2
7459	207387_s_at	2.021288889	5.72E-11	GK
7460	204187_at	2.025240741	5.26E-12	GMPR
7461	242409_at	2.025255556	8.88E-07	
7462	217874_at	2.026744444	6.72E-16	SUCLG1
7463	223467_at	2.026803704	1.01E-07	RASD1
7464	207367_at	2.026881481	6.78E-07	ATP12A
7465	1563874_at	2.02712963	2.11E-08	WDR72
7466	1552362_a_at	2.028166667	3.80E-07	LEAP2
7467	229358_at	2.0288	4.68E-08	IHH
7468	240745_at	2.030722222	9.71E-07	LOC101927760
7469	216481_at	2.030759259	1.80E-09	GRIP2
7470	219541_at	2.031451852	5.90E-08	LIME1
7471	232428_at	2.031488889	5.28E-08	MOGAT2
7472	1552510_at	2.03277037	6.84E-14	SLC34A3
7473	213894_at	2.033003704	5.34E-09	THSD7A
7474	235617_x_at	2.0348	4.06E-08	LOC100507537

7475	242871_at	2.035577778	3.24E-13	PAQR5
7476	238498_at	2.03657037	5.06E-08	RP3-406A7.7
7477	220415_at	2.03662963	1.38E-06	FPGT-TNNI3K /// TNNI3K
7478	226210_s_at	2.038292593	3.50E-06	MEG3
7479	210130_s_at	2.039074074	3.45E-11	TM7SF2
7480	1559861_at	2.039462963	2.90E-06	LOC101928099
7481	211806_s_at	2.041151852	5.25E-08	KCNJ15
7482	218792_s_at	2.042466667	1.43E-08	BSPRY
7483	1553915_at	2.0426	2.05E-06	C10orf126
7484	203088_at	2.044940741	4.47E-10	FBLN5
7485	234098_at	2.045033333	2.05E-06	SOBP
7486	233648_at	2.045140741	4.89E-08	
7487	229032_at	2.045533333	4.79E-08	WSCD2
7488	209994_s_at	2.046385185	3.56E-10	ABCB1 /// ABCB4
7489	243990_at	2.046385185	2.51E-06	
7490	244427_at	2.046437037	4.18E-08	KIF23
7491	231009_at	2.046696296	8.73E-08	PLA2G12B
7492	240159_at	2.047762963	1.83E-12	SLC15A2
7493	231180_at	2.04807037	6.67E-07	
7494	1560698_a_at	2.04977037	2.19E-07	TRHDE-AS1
7495	206263_at	2.050988889	9.87E-13	FMO4
7496	219615_s_at	2.051981481	1.19E-08	KCNK5
7497	220186_s_at	2.052937037	1.27E-09	CDHR2
7498	209442_x_at	2.053988889	2.70E-10	ANK3
7499	1552302_at	2.054192593	3.28E-12	TMEM106A
7500	209016_s_at	2.055596296	1.93E-07	KRT7
7501	242373_at	2.056248148	7.96E-12	
7502	227197_at	2.058459259	1.02E-10	ARHGEF26
7503	221868_at	2.058581481	2.07E-06	PAIP2B
7504	207010_at	2.060040741	4.08E-07	GABRB1
7505	236949_at	2.060159259	8.81E-07	
7506	218024_at	2.060355556	1.32E-14	MPC1
7507	243456_at	2.060581481	1.11E-06	ZNF214
7508	218934_s_at	2.060611111	2.21E-08	HSPB7
7509	212148_at	2.061814815	1.06E-09	PBX1
7510	218275_at	2.062655556	1.25E-12	SLC25A10
7511	229335_at	2.063281481	9.43E-10	CADM4
7512	229178_at	2.063711111	4.09E-07	PRTG
7513	204044_at	2.064559259	2.52E-09	QPRT
7514	238454_at	2.065844444	1.06E-07	ZNF540
7515	218272_at	2.067522222	5.18E-11	TTC38
7516	202025_x_at	2.06757037	9.94E-17	ACAA1
7517	239148_at	2.067807407	1.63E-08	MARVELD3

7518	205942_s_at	2.068040741	8.89E-10	ACSM3
7519	243932_at	2.068177778	3.55E-07	
7520	214763_at	2.068225926	3.14E-12	ACOT11
7521	1556737_at	2.068307407	8.41E-09	LINC00671
7522	204758_s_at	2.068759259	1.26E-08	C2CD2L
7523	225272_at	2.069922222	2.99E-16	SAT2
7524	206371_at	2.070251852	1.25E-13	FOLR3
7525	232720_at	2.070681481	6.39E-13	LINGO2
7526	237995_at	2.070718519	9.32E-07	RIMBP2
7527	205331_s_at	2.07132963	8.11E-10	REEP2
7528	234605_at	2.071618519	1.33E-08	CDC14B
7529	229901_at	2.075137037	1.40E-08	ZNF488
7530	224954_at	2.075485185	2.03E-10	MIR6778 /// SHMT1
7531	224411_at	2.076707407	3.79E-06	PLA2G12B
7532	228978_at	2.076903704	2.58E-07	RP11-332H18.4
7533	226273_at	2.078159259	6.60E-12	CLCN5
7534	221030_s_at	2.078696296	9.36E-08	ARHGAP24
7535	238835_at	2.078914815	7.46E-07	AVPR1A
7536	201036_s_at	2.07997037	7.82E-17	HADH
7537	216381_x_at	2.080066667	3.39E-11	AKR7A3
7538	235538_at	2.080285185	8.83E-12	
7539	214033_at	2.080981481	1.52E-08	ABCC6 /// ABCC6P1 /// ABCC6P2 /// LOC101930322
7540	234219_at	2.082218519	8.24E-09	LOC101928820
7541	221624_at	2.082785185	4.32E-09	TCL6
7542	233482_at	2.083248148	7.60E-08	C15orf59
7543	1564679_at	2.08337037	5.43E-06	ASB15
7544	217167_x_at	2.08377037	8.61E-11	GK
7545	228880_at	2.084111111	6.08E-10	NAT8L
7546	235182_at	2.086107407	7.83E-08	ISM1
7547	238726_at	2.089192593	1.01E-09	
7548	222967_at	2.089196296	1.82E-06	SLC5A7
7549	206704_at	2.089244444	1.78E-10	CLCN5
7550	201839_s_at	2.09112963	1.55E-06	EPCAM
7551	237572_at	2.093059259	1.95E-06	UGT3A1
7552	227000_at	2.093711111	7.58E-12	MTURN
7553	240886_at	2.094048148	4.42E-09	CASR
7554	1556927_at	2.095222222	8.18E-06	KCNK10
7555	241181_x_at	2.096155556	1.08E-11	
7556	208396_s_at	2.097251852	6.17E-07	PDE1A
7557	213348_at	2.097496296	1.69E-10	CDKN1C
7558	237716_at	2.097707407	3.94E-10	
7559	209504_s_at	2.098103704	7.50E-09	PLEKHB1

7560	244387_at	2.098103704	5.43E-07	
7561	214423_x_at	2.098240741	6.63E-17	ALDOB
7562	227727_at	2.099081481	2.98E-09	MRGPRF
7563	240389_at	2.100414815	3.08E-07	TRPM6
7564	206048_at	2.101	2.08E-07	OVOL2
7565	1560384_a_at	2.101296296	5.29E-10	BC042091 /// RP11-752D24.2
7566	244741_s_at	2.10152963	7.19E-11	ZNF667-AS1
7567	233279_at	2.101740741	3.18E-06	
7568	1560834_a_at	2.104788889	9.05E-08	RMST
7569	230067_at	2.105633333	2.65E-09	FAM124A
7570	224053_s_at	2.107118519	9.38E-11	SLC4A9
7571	241859_at	2.107225926	1.93E-11	PLCL1
7572	1565733_at	2.108074074	1.55E-06	
7573	231994_at	2.1108	1.18E-08	CHDH
7574	230776_at	2.113403704	8.91E-06	RNF157-AS1
7575	228973_at	2.117518519	1.80E-09	DLG2
7576	239984_at	2.119696296	1.38E-06	
7577	1561899_at	2.120433333	6.89E-07	CLECL1
7578	237257_at	2.120903704	3.39E-12	RAB4B
7579	213839_at	2.121592593	1.29E-10	CLMN
7580	234480_at	2.121703704	1.96E-06	DKFZP761C1711
7581	204973_at	2.122174074	2.22E-09	GJB1
7582	236150_at	2.123959259	2.47E-13	HYKK
7583	227163_at	2.125655556	1.78E-09	GSTO2
7584	1566551_at	2.128692593	1.40E-07	
7585	214896_at	2.129007407	7.54E-12	LINC01314
7586	209243_s_at	2.132292593	1.08E-08	PEG3
7587	1554722_at	2.13242963	9.80E-08	TRPM3
7588	227865_at	2.133022222	2.57E-11	IDNK
7589	206601_s_at	2.133651852	2.43E-09	HOXD3 /// HOXD4 /// LOC401021
7590	226548_at	2.133892593	1.90E-07	SBK1
7591	230863_at	2.134281481	5.88E-06	LRP2
7592	216074_x_at	2.13687037	7.15E-06	WWC1
7593	237942_at	2.137481481	9.44E-08	SNRK-AS1
7594	243922_at	2.142948148	2.91E-11	
7595	205617_at	2.143492593	1.94E-13	PRRG2
7596	206469_x_at	2.144881481	7.50E-12	AKR7A3
7597	215027_at	2.146196296	2.78E-14	RAPGEF3
7598	213954_at	2.147059259	1.75E-10	FAM169A
7599	205750_at	2.14862963	2.62E-15	BPHL
7600	244124_at	2.151492593	8.11E-10	
7601	212821_at	2.152566667	1.60E-07	PLEKHG3
7602	1559590_at	2.154503704	8.81E-07	CHDH

7603	222078_at	2.154781481	5.44E-09	PKLR
7604	204654_s_at	2.155674074	6.58E-10	TFAP2A
7605	231916_at	2.156696296	6.96E-09	NOS1
7606	1562669_at	2.156951852	8.94E-08	
7607	244855_at	2.157188889	1.00E-05	
7608	233336_at	2.158281481	7.81E-06	
7609	230782_at	2.160803704	5.22E-11	SORD
7610	207327_at	2.161637037	1.45E-06	EYA4
7611	205164_at	2.164148148	1.65E-07	GCAT
7612	238518_x_at	2.165985185	3.65E-15	GLYCTK
7613	233547_x_at	2.166081481	3.95E-06	PDE1A
7614	212510_at	2.167262963	6.69E-17	GPD1L
7615	1558170_at	2.167585185	7.26E-06	
7616	233801_s_at	2.1697	1.60E-06	SEMA6D
7617	211465_x_at	2.171422222	3.40E-15	FUT6
7618	231325_at	2.171874074	2.28E-06	UNC5D
7619	1556346_at	2.172574074	4.17E-08	COTL1
7620	242093_at	2.173596296	8.03E-07	SYTL5
7621	203434_s_at	2.174433333	1.41E-07	MME
7622	211456_x_at	2.174596296	3.95E-09	MT1HL1
7623	214342_at	2.176185185	7.54E-12	ATXN7L1
7624	223805_at	2.176777778	5.84E-08	OSBPL6
7625	205712_at	2.176933333	2.56E-08	PTPRD
7626	236843_at	2.178307407	1.79E-10	NOX4
7627	229088_at	2.179125926	3.55E-10	ENPP1
7628	243756_at	2.179396296	2.57E-07	
7629	228621_at	2.180374074	6.25E-09	HFE2
7630	243027_at	2.181114815	4.51E-07	IGSF5
7631	1553364_at	2.1813	8.68E-07	PNPLA1
7632	224339_s_at	2.181685185	2.54E-06	ANGPTL1
7633	205253_at	2.182740741	1.31E-07	PBX1
7634	221572_s_at	2.182848148	5.59E-11	MIR6824 /// SLC26A6
7635	233130_at	2.183437037	2.22E-11	THRB-IT1 /// THRB-IT1
7636	212750_at	2.187125926	5.37E-12	PPP1R16B
7637	230951_at	2.188388889	2.21E-08	EPB41L5
7638	224482_s_at	2.1894	8.88E-09	RAB11FIP4
7639	210565_at	2.191159259	2.34E-07	GCGR
7640	228400_at	2.191833333	7.47E-06	SHROOM3
7641	231015_at	2.193022222	1.45E-09	KLF15
7642	223928_s_at	2.194911111	2.28E-06	GUCA1C
7643	211458_s_at	2.196766667	1.87E-16	GABARAPL1 /// GABARAPL3
7644	231729_s_at	2.196985185	1.78E-07	CAPS
7645	243369_at	2.198303704	4.42E-09	

7646	1559355_at	2.201959259	3.79E-07	NXPH2
7647	35148_at	2.202711111	7.70E-14	TJP3
7648	204412_s_at	2.203481481	6.20E-08	NEFH
7649	229024_at	2.203844444	2.38E-14	RNF150
7650	226591_at	2.204248148	9.36E-12	PWAR6
7651	243039_at	2.204348148	1.29E-15	
7652	239350_at	2.204481481	1.31E-09	MARVELD3
7653	236775_s_at	2.206551852	1.11E-07	
7654	222746_s_at	2.207255556	6.69E-09	BSPRY
7655	1557122_s_at	2.207896296	8.55E-09	GABRB2
7656	202597_at	2.207925926	7.62E-09	IRF6
7657	211422_at	2.208166667	1.79E-08	TRPM3
7658	208567_s_at	2.208477778	7.88E-08	KCNJ12 /// KCNJ18 /// LOC100996843
7659	216011_at	2.209588889	3.98E-08	SLC39A9
7660	205691_at	2.210048148	5.05E-06	SYNGR3
7661	229599_at	2.210188889	6.52E-09	SMIM22
7662	223596_at	2.211014815	8.31E-14	SLC12A6
7663	201562_s_at	2.211381481	1.45E-15	SORD
7664	239572_at	2.211944444	2.97E-08	GJA3
7665	211549_s_at	2.212240741	4.44E-08	HPGD
7666	216456_at	2.212262963	2.86E-08	
7667	233022_at	2.213	5.87E-11	TRPM3
7668	221716_s_at	2.218018519	3.42E-10	ACSBG2
7669	206527_at	2.218507407	4.56E-18	ABAT
7670	214433_s_at	2.219811111	2.04E-12	SELENBP1
7671	208121_s_at	2.219907407	9.69E-10	PTPRO
7672	202888_s_at	2.221266667	1.40E-07	ANPEP
7673	1556469_s_at	2.222096296	1.24E-06	AX747261
7674	236031_x_at	2.222848148	2.15E-10	FREM1
7675	1569290_s_at	2.223081481	9.51E-06	GRIA3
7676	208471_at	2.223833333	4.33E-06	HPR
7677	215686_x_at	2.223996296	2.80E-07	TFAP2B
7678	211470_s_at	2.22452963	3.85E-07	SULT1C2
7679	226573_at	2.224674074	1.26E-09	DIRAS1
7680	218796_at	2.2262	1.64E-10	FERMT1
7681	204388_s_at	2.229662963	4.62E-08	MAOA
7682	238834_at	2.2318	6.12E-11	MYLK3
7683	219915_s_at	2.232222222	2.09E-12	SLC16A10
7684	216893_s_at	2.232333333	4.12E-08	COL4A3
7685	208434_at	2.232362963	1.02E-06	MECOM
7686	1563906_at	2.234011111	4.64E-09	SOBP
7687	238245_at	2.23432963	4.91E-08	ENPP7
7688	209242_at	2.235033333	1.96E-12	PEG3

7689	210374_x_at	2.237337037	4.56E-08	PTGER3
7690	216600_x_at	2.240088889	9.30E-16	ALDOB
7691	244723_at	2.242788889	2.77E-09	ACSM2A
7692	41577_at	2.24362963	1.04E-11	PPP1R16B
7693	242789_at	2.244781481	2.68E-06	PDE1A
7694	210399_x_at	2.246237037	2.03E-11	FUT6
7695	242543_at	2.248307407	7.61E-10	LOC101928082 /// SH2D6
7696	236840_at	2.248322222	7.76E-06	C12orf56
7697	228320_x_at	2.250296296	1.23E-10	CCDC64
7698	218261_at	2.251359259	5.45E-13	AP1M2
7699	211116_at	2.251514815	1.44E-06	SLC9A2
7700	244665_at	2.251659259	1.28E-07	
7701	210119_at	2.252048148	5.90E-08	KCNJ15
7702	202712_s_at	2.253714815	2.44E-09	CKMT1A /// CKMT1B
7703	237870_at	2.253837037	1.36E-10	NQO2
7704	236639_at	2.254462963	5.79E-06	LOC101926906
7705	235050_at	2.254862963	3.82E-08	SLC2A12
7706	230519_at	2.256688889	3.35E-08	FAM124A
7707	243060_at	2.256796296	1.99E-06	
7708	210523_at	2.25912963	1.26E-06	BMPR1B
7709	239983_at	2.259255556	1.92E-09	SLC30A8
7710	223858_at	2.259566667	6.95E-11	ESRRB
7711	232282_at	2.260133333	1.25E-11	WNK3
7712	219551_at	2.262181481	2.18E-15	EAF2
7713	213380_x_at	2.262211111	1.29E-08	LOC101930052 /// LOC102724562 /// MST1 /// MST1L /// MST1P2
7714	218974_at	2.262277778	1.08E-13	SOBP
7715	217562_at	2.262885185	3.88E-06	BRINP3
7716	239533_at	2.266051852	3.82E-11	GPR155
7717	237881_at	2.268425926	2.27E-06	
7718	65517_at	2.2689	4.47E-14	AP1M2
7719	227886_at	2.271537037	3.92E-09	IFITM10
7720	219873_at	2.272651852	4.53E-13	COLEC11
7721	1560025_at	2.273722222	1.36E-10	AK094644
7722	242308_at	2.274622222	6.80E-09	MCOLN3
7723	233047_at	2.274711111	8.62E-09	FRMD7
7724	214891_at	2.276381481	6.61E-10	FBXO21
7725	233979_s_at	2.278448148	3.84E-09	ESPN
7726	1555500_s_at	2.278940741	2.59E-15	SLC2A4RG
7727	1562016_at	2.28132963	1.58E-08	
7728	217165_x_at	2.281751852	4.97E-09	MT1F
7729	239656_at	2.284792593	2.57E-08	LHFPL3-AS2
7730	1557776_at	2.285285185	4.48E-11	LOC101928303

7731	210833_at	2.286940741	9.28E-08	PTGER3
7732	230595_at	2.286974074	4.96E-07	PGM5-AS1
7733	1552548_at	2.28717037	2.37E-09	BSND
7734	213110_s_at	2.289048148	1.46E-07	COL4A5
7735	206385_s_at	2.293122222	2.79E-06	ANK3
7736	243952_at	2.295	1.78E-07	TPTEP1
7737	230802_at	2.298722222	5.11E-12	ARHGAP24
7738	236893_at	2.299055556	1.27E-08	HOXB-AS3
7739	205259_at	2.299188889	5.59E-09	NR3C2
7740	205471_s_at	2.301325926	6.81E-14	DACH1
7741	244151_at	2.303037037	4.17E-11	SMLR1
7742	1555310_a_at	2.303196296	4.99E-16	PAK6
7743	239921_at	2.305818519	1.04E-06	COL28A1
7744	226594_at	2.307259259	2.56E-13	ENTPD5
7745	220161_s_at	2.30777037	1.12E-09	EPB41L4B
7746	1554837_a_at	2.308477778	1.37E-11	CYP4A11 /// CYP4A22
7747	227226_at	2.310125926	7.95E-08	MRAP2
7748	203287_at	2.311085185	5.59E-13	LAD1
7749	206381_at	2.315162963	1.73E-09	SCN2A
7750	1562235_s_at	2.31592963	4.82E-09	
7751	204379_s_at	2.316522222	8.91E-09	FGFR3
7752	221122_at	2.317477778	1.41E-12	HRASLS2
7753	207095_at	2.319248148	4.81E-06	SLC10A2
7754	241694_at	2.319366667	1.38E-09	PKHD1
7755	209335_at	2.320722222	3.08E-07	DCN
7756	210064_s_at	2.32292963	1.09E-09	UPK1B
7757	207542_s_at	2.323766667	1.28E-07	AQP1
7758	1569555_at	2.325618519	1.28E-08	GDA
7759	1553722_s_at	2.326037037	2.76E-10	RNF152
7760	243776_at	2.327644444	5.20E-08	RP11-63A11.1
7761	228918_at	2.32857037	2.63E-12	SLC43A2
7762	240331_at	2.331344444	7.99E-08	
7763	221701_s_at	2.331637037	1.57E-13	STRA6
7764	206043_s_at	2.336803704	2.17E-12	ATP2C2
7765	238255_at	2.338196296	1.25E-12	
7766	1563792_at	2.33947037	1.57E-09	AMN
7767	205710_at	2.340974074	2.04E-06	LRP2
7768	206149_at	2.34102963	5.59E-07	CHP2
7769	240821_at	2.343292593	1.58E-09	
7770	1554343_a_at	2.343881481	1.58E-09	STAP1
7771	221796_at	2.344437037	7.62E-08	NTRK2
7772	216010_x_at	2.344618519	4.18E-10	FUT3
7773	202525_at	2.345759259	2.54E-07	PRSS8

7774	229337_at	2.346011111	7.56E-11	USP2
7775	219995_s_at	2.346762963	4.81E-06	ZNF750
7776	238451_at	2.346911111	1.68E-09	MPP7
7777	1559265_at	2.34812963	5.11E-07	SKIDA1
7778	226610_at	2.348718519	2.80E-06	CENPV
7779	243386_at	2.348925926	8.84E-14	CASZ1
7780	205589_at	2.349551852	9.95E-08	MYL3
7781	236101_at	2.351122222	6.79E-09	
7782	218687_s_at	2.351725926	3.70E-08	MUC13
7783	228017_s_at	2.3521	3.64E-06	NKAIN4
7784	229613_at	2.354718519	7.12E-09	RP11-401P9.4
7785	229296_at	2.355555556	2.50E-08	LOC100506119
7786	240480_at	2.356266667	8.22E-06	TINAG
7787	231232_at	2.3572	2.02E-08	LOC100506125
7788	227019_at	2.3577	5.04E-12	C1orf226
7789	206045_s_at	2.357866667	4.25E-06	NOL4
7790	214490_at	2.358266667	2.87E-07	ARSF
7791	209992_at	2.361062963	6.44E-17	PFKFB2
7792	206617_s_at	2.361877778	3.19E-09	RENBP
7793	238778_at	2.363525926	1.10E-10	MPP7
7794	233882_s_at	2.365688889	4.15E-10	SEMA6D
7795	232882_at	2.365862963	1.50E-08	
7796	209047_at	2.366411111	2.95E-09	AQP1
7797	228943_at	2.366481481	2.84E-11	MAP6
7798	219368_at	2.367003704	3.17E-06	NAP1L2
7799	223549_s_at	2.367159259	1.97E-08	ESPN
7800	228865_at	2.367514815	8.23E-08	C1orf116
7801	226733_at	2.368718519	1.40E-15	PFKFB2
7802	204588_s_at	2.370037037	8.90E-11	SLC7A7
7803	203071_at	2.371366667	3.39E-11	MIR6872 /// SEMA3B
7804	239965_at	2.372085185	6.94E-07	
7805	228613_at	2.372551852	3.92E-11	RAB11FIP3
7806	228377_at	2.372611111	7.03E-10	KLHL14
7807	213412_at	2.372981481	3.70E-09	TJP3
7808	221275_s_at	2.375455556	6.47E-09	
7809	220133_at	2.376725926	2.86E-08	ODAM
7810	225165_at	2.377133333	4.75E-09	PPP1R1B
7811	222271_at	2.377840741	1.07E-12	
7812	1555175_a_at	2.377985185	1.64E-07	PBLD
7813	235161_at	2.379711111	7.58E-09	RASSF8-AS1
7814	206775_at	2.379922222	1.97E-09	CUBN
7815	231981_at	2.380151852	4.04E-13	PRLR
7816	222853_at	2.385762963	2.84E-06	FLRT3

7817	238253_at	2.386837037	1.84E-10	WDR72
7818	205325_at	2.388648148	1.05E-09	PHYHIP
7819	230914_at	2.389692593	7.37E-07	HNF4A
7820	211663_x_at	2.390540741	1.26E-07	PTGDS
7821	209857_s_at	2.392548148	1.28E-09	SPHK2
7822	236038_at	2.392788889	7.23E-15	RNF150
7823	244344_at	2.393318519	7.91E-08	WNK4
7824	210832_x_at	2.396744444	1.69E-08	PTGER3
7825	204457_s_at	2.397818519	3.05E-09	GAS1
7826	205328_at	2.399196296	1.20E-06	CLDN10
7827	230949_at	2.399755556	1.83E-06	SLC23A3
7828	203797_at	2.401022222	2.31E-09	VSNL1
7829	1554027_a_at	2.403577778	8.36E-13	SLC4A4
7830	1556641_at	2.406522222	1.76E-08	SLC7A14
7831	1559351_at	2.407362963	6.13E-06	
7832	219789_at	2.408192593	2.18E-09	NPR3
7833	230716_at	2.409022222	1.21E-06	SMLR1
7834	1562440_at	2.409103704	5.36E-06	MAP3K13
7835	221232_s_at	2.409766667	2.02E-06	ANKRD2
7836	219722_s_at	2.411433333	8.77E-13	GDPD3
7837	1554300_a_at	2.414525926	2.59E-08	SVOPL
7838	237484_at	2.418137037	4.74E-11	SMCO3
7839	206797_at	2.418911111	6.20E-06	NAT2
7840	231880_at	2.421514815	1.44E-06	STRIP2
7841	230044_at	2.422981481	6.53E-11	PCYT2
7842	1570506_at	2.423792593	1.07E-08	
7843	241369_at	2.42492963	1.63E-07	LOC101929335
7844	240223_at	2.425437037	7.80E-08	SLC2A9
7845	206539_s_at	2.425848148	1.88E-09	CYP4F12
7846	216641_s_at	2.42942963	2.04E-12	LAD1
7847	239849_at	2.431244444	8.29E-07	
7848	216012_at	2.433666667	4.61E-11	
7849	240175_at	2.433881481	1.16E-13	
7850	219281_at	2.433981481	1.91E-14	MSRA
7851	202831_at	2.434322222	5.82E-10	GPX2
7852	218487_at	2.437125926	1.14E-14	ALAD
7853	204437_s_at	2.437366667	2.44E-09	FOLR1
7854	215692_s_at	2.441166667	6.35E-12	MPPED2
7855	231426_at	2.442788889	3.54E-06	
7856	204712_at	2.444896296	1.36E-07	WIF1
7857	206461_x_at	2.445396296	4.43E-11	MT1H
7858	206681_x_at	2.445766667	5.64E-08	GP2
7859	210121_at	2.447777778	6.39E-06	B3GALT2

7860	236058_at	2.448055556	1.73E-07	KDF1
7861	208307_at	2.449551852	1.29E-06	RBMY1A1 /// RBMY1B /// RBMY1D /// RBMY1E /// RBMY1F /// RBMY1J
7862	229596_at	2.449562963	3.51E-08	AMDHD1
7863	214681_at	2.451633333	1.66E-14	GK
7864	226274_at	2.452677778	1.43E-12	CLCN5
7865	229309_at	2.453292593	2.53E-12	ADRB1
7866	240200_at	2.453411111	5.68E-10	SULT1C2
7867	1556762_a_at	2.454048148	3.23E-07	
7868	241547_at	2.455611111	7.72E-06	A1CF
7869	1563881_at	2.455922222	2.30E-09	MAGI1
7870	238755_at	2.456140741	7.36E-09	RASSF10
7871	242901_at	2.456185185	3.29E-09	
7872	214811_at	2.4562	7.13E-08	RIMBP2
7873	1553729_s_at	2.458962963	3.09E-09	LRRC43
7874	204424_s_at	2.460322222	4.88E-09	LMO3
7875	205208_at	2.460774074	8.34E-09	ALDH1L1
7876	236934_at	2.462714815	1.06E-10	
7877	1554242_a_at	2.462788889	1.73E-06	COCH
7878	230222_at	2.464022222	3.02E-10	RP11-456H18.2
7879	210262_at	2.468637037	3.28E-08	CRISP2
7880	230917_at	2.468737037	2.62E-15	PLCG2
7881	220892_s_at	2.470325926	8.65E-09	PSAT1
7882	233442_at	2.470514815	8.01E-08	
7883	215653_at	2.47342963	2.07E-06	
7884	206610_s_at	2.475088889	4.55E-09	F11
7885	219121_s_at	2.477314815	3.96E-07	ESRP1
7886	215126_at	2.479385185	4.35E-09	LINC01314
7887	220432_s_at	2.482807407	8.49E-08	CYP39A1
7888	1552826_at	2.484451852	1.86E-10	SLC26A7
7889	216733_s_at	2.48467037	8.20E-09	GATM
7890	233450_at	2.485196296	1.24E-08	
7891	205833_s_at	2.486692593	8.06E-08	PART1
7892	215264_at	2.48737037	2.13E-11	EMX1
7893	235867_at	2.487451852	1.45E-15	GSTM3
7894	230785_at	2.488096296	2.32E-09	SALL3
7895	221054_s_at	2.4886	7.78E-07	TCL6
7896	243951_at	2.488737037	8.85E-13	ABCB1
7897	231693_at	2.488855556	4.93E-10	FABP1
7898	204717_s_at	2.493596296	5.21E-11	SLC29A2
7899	220507_s_at	2.496051852	4.01E-06	UPB1
7900	215259_s_at	2.496707407	5.30E-09	CADM4
7901	214274_s_at	2.496859259	2.06E-17	ACAA1

7902	230573_at	2.498640741	2.53E-14	SGK2
7903	212915_at	2.499044444	1.75E-08	PDZRN3
7904	209993_at	2.499425926	1.07E-09	ABCB1
7905	1555471_a_at	2.502574074	6.22E-08	FMN2
7906	242271_at	2.503562963	4.55E-08	SLC26A9
7907	224901_at	2.505085185	3.42E-12	SCD5
7908	236302_at	2.505248148	3.24E-11	PPM1E
7909	230946_at	2.505392593	1.16E-10	FMN2
7910	215300_s_at	2.505866667	5.02E-09	FMO5
7911	244565_at	2.506374074	3.52E-12	HMX2
7912	1556761_at	2.506833333	2.19E-06	
7913	225757_s_at	2.507648148	1.65E-11	CLMN
7914	201893_x_at	2.511396296	1.95E-06	DCN
7915	1555252_a_at	2.512474074	7.27E-09	TRPM3
7916	236261_at	2.515322222	2.52E-13	OSBPL6
7917	205844_at	2.516796296	5.71E-08	VNN1
7918	224209_s_at	2.521540741	4.09E-06	GDA
7919	223784_at	2.522181481	4.91E-06	TMEM27
7920	223822_at	2.522296296	7.05E-08	SUSD4
7921	231941_s_at	2.522414815	1.10E-06	MUC20
7922	237727_at	2.525859259	2.76E-12	
7923	208367_x_at	2.525937037	2.55E-06	CYP3A4
7924	1559410_at	2.527059259	6.82E-10	
7925	227560_at	2.528855556	7.78E-17	SFXN2
7926	205082_s_at	2.529048148	3.95E-08	AOX1
7927	228850_s_at	2.530933333	2.06E-12	SLIT2
7928	1564841_at	2.530955556	7.78E-06	
7929	237226_at	2.533788889	8.28E-10	RP11-503C24.6
7930	243610_at	2.536285185	3.37E-06	C9orf135
7931	218976_at	2.536374074	2.49E-13	DNAJC12
7932	1555787_at	2.536774074	1.40E-12	C11orf63
7933	205489_at	2.538096296	1.67E-10	CRYM
7934	202108_at	2.540059259	7.01E-14	PEPD
7935	239860_at	2.5414	2.05E-06	LOC100130232
7936	228739_at	2.541725926	9.06E-08	CYS1
7937	243755_at	2.54192963	2.06E-14	PRLR
7938	220224_at	2.542388889	6.31E-07	HAO1
7939	221221_s_at	2.544562963	1.27E-13	KLHL3
7940	228086_at	2.547540741	3.85E-09	STK33
7941	240927_at	2.551096296	7.05E-08	
7942	236597_at	2.552207407	8.81E-08	UGT3A1
7943	238877_at	2.553540741	1.88E-07	EYA4
7944	216638_s_at	2.553755556	3.74E-11	PRLR

7945	1552578_a_at	2.55377037	3.18E-08	MYO3B
7946	242344_at	2.556625926	1.01E-08	GABRB2
7947	241915_at	2.558092593	1.32E-08	ACSM2B
7948	210861_s_at	2.558592593	4.07E-08	WISP3
7949	224361_s_at	2.559392593	5.33E-10	IL17RB
7950	243614_s_at	2.560303704	2.54E-09	PRODH2
7951	1554938_a_at	2.562492593	3.37E-12	ACOT11
7952	1553912_at	2.563025926	1.13E-07	LINC00955
7953	242414_at	2.56372963	7.39E-12	LOC101929880 /// QPRT
7954	236236_at	2.563996296	9.30E-11	WNK3
7955	211552_s_at	2.566525926	2.60E-14	ALDH4A1
7956	207412_x_at	2.566574074	1.23E-08	CELP
7957	1558402_at	2.567574074	8.39E-08	CTD-3080P12.3
7958	239464_at	2.568403704	5.41E-10	KCNJ15
7959	240422_at	2.569925926	2.95E-09	
7960	205506_at	2.574640741	6.88E-07	VIL1
7961	231781_s_at	2.575607407	2.13E-11	LRRC2
7962	223721_s_at	2.578688889	3.78E-12	DNAJC12
7963	206325_at	2.579322222	1.02E-07	SERPINA6
7964	206208_at	2.580251852	1.63E-08	CA4
7965	202718_at	2.583540741	5.74E-08	IGFBP2
7966	205155_s_at	2.584222222	3.81E-11	SPTBN2
7967	60474_at	2.584259259	3.47E-08	FERMT1
7968	1559277_at	2.584566667	2.63E-08	FLJ35700
7969	223074_s_at	2.587659259	9.08E-10	AIF1L
7970	223782_s_at	2.587837037	2.80E-07	TINAG
7971	225911_at	2.59032963	1.38E-09	NPNT
7972	228335_at	2.590366667	6.27E-07	CLDN11
7973	208473_s_at	2.592340741	2.07E-08	GP2
7974	218332_at	2.593511111	7.58E-08	BEX1
7975	237031_at	2.595881481	8.65E-08	RP11-21L23.2
7976	209789_at	2.596237037	4.94E-08	CORO2B
7977	214180_at	2.598803704	1.47E-16	MAN1C1
7978	238133_at	2.601514815	2.84E-09	
7979	226403_at	2.60607037	4.20E-11	TMC4
7980	219268_at	2.608122222	9.38E-17	ETNK2
7981	216953_s_at	2.610248148	3.70E-06	WT1
7982	239707_at	2.610948148	4.16E-07	SLC5A10
7983	244264_at	2.611877778	5.61E-11	KLRG2
7984	1566970_at	2.612251852	1.60E-07	
7985	238106_at	2.61257037	1.90E-12	
7986	239381_at	2.613114815	1.82E-11	KLK7
7987	237058_x_at	2.614792593	6.42E-07	SLC6A13

7988	219414_at	2.615925926	1.10E-07	CLSTN2
7989	1554804_a_at	2.616207407	9.61E-12	CLDN19
7990	238160_at	2.617911111	7.93E-11	ACOT12
7991	226030_at	2.620581481	3.14E-16	ACADSB
7992	1556271_at	2.62252963	1.15E-10	LOC101928896
7993	219388_at	2.623659259	1.61E-12	GRHL2
7994	214424_s_at	2.627277778	4.12E-11	ALDOB
7995	227764_at	2.628133333	2.20E-08	LYPD6
7996	207858_s_at	2.62867037	1.59E-06	PKLR
7997	210020_x_at	2.6298	4.83E-11	CALML3
7998	232748_at	2.630674074	2.48E-08	PAPPA
7999	233030_at	2.631337037	1.15E-10	PNPLA3
8000	1570189_at	2.632055556	1.52E-08	LINC00671
8001	213332_at	2.632492593	2.07E-11	PAPPA2
8002	225817_at	2.633996296	5.91E-10	CGNL1 /// LOC101930344 /// LOC101930349
8003	232771_at	2.634533333	7.49E-08	NRK
8004	215559_at	2.63492963	2.03E-07	ABCC6 /// LOC101930322
8005	236389_x_at	2.635203704	5.49E-10	
8006	219945_at	2.635307407	2.01E-13	DDX25
8007	237341_at	2.637662963	2.88E-13	
8008	210194_at	2.639588889	1.68E-09	PLA2R1
8009	226622_at	2.642940741	3.73E-06	MUC20
8010	227209_at	2.643862963	4.50E-11	CNTN1
8011	1560119_at	2.64657037	4.12E-06	LINC00937
8012	219543_at	2.649685185	1.19E-09	PBLD
8013	214641_at	2.6536	2.97E-08	COL4A3
8014	214204_at	2.6568	1.34E-17	PACRG
8015	206230_at	2.658866667	6.85E-13	LHX1
8016	230971_x_at	2.662011111	2.73E-07	GLTPD2
8017	1554199_at	2.663040741	7.15E-09	PTPRO
8018	204733_at	2.663559259	1.22E-10	KLK6
8019	213629_x_at	2.668533333	8.92E-11	MT1F
8020	204447_at	2.668692593	3.56E-14	LZTS3
8021	223232_s_at	2.668718519	3.86E-11	CGN
8022	240541_at	2.669833333	7.64E-13	
8023	219370_at	2.675862963	2.53E-10	RPRM
8024	217013_at	2.676081481	2.59E-10	AZGP1P1
8025	231336_at	2.677877778	7.65E-10	CPNE4
8026	201563_at	2.679740741	2.11E-16	SORD
8027	231334_at	2.679888889	7.87E-14	LOC101929480
8028	226961_at	2.684544444	3.82E-13	PRR15
8029	214043_at	2.68512963	7.16E-11	PTPRD
8030	217568_at	2.685659259	5.82E-09	EDDM3A

8031	240060_at	2.687274074	1.87E-11	
8032	241509_at	2.687833333	1.41E-06	
8033	237491_at	2.688366667	4.06E-08	
8034	237269_at	2.69042963	5.28E-10	
8035	205102_at	2.693625926	1.10E-12	TMPRSS2
8036	202847_at	2.694425926	8.53E-15	PCK2
8037	214023_x_at	2.6952	2.10E-09	TUBB2B
8038	215314_at	2.695637037	6.58E-12	
8039	1554246_at	2.696296296	1.45E-06	C1orf210
8040	1552535_at	2.698988889	5.32E-11	CLDN19
8041	239726_at	2.699862963	5.17E-12	ANK3
8042	204199_at	2.7008	1.03E-12	RALGPS1
8043	209897_s_at	2.701074074	1.41E-12	SLIT2
8044	1555490_s_at	2.701888889	1.46E-11	PDZD3
8045	232067_at	2.702007407	4.91E-06	FAXC
8046	210051_at	2.702996296	8.88E-13	RAPGEF3
8047	205177_at	2.703444444	4.01E-09	TNNI1
8048	211748_x_at	2.703714815	3.22E-08	PTGDS
8049	212187_x_at	2.708711111	6.97E-09	PTGDS
8050	237108_x_at	2.710174074	1.40E-13	LINC00982
8051	205316_at	2.710540741	2.78E-17	SLC15A2
8052	209368_at	2.713040741	5.14E-08	EPHX2
8053	223748_at	2.713240741	5.30E-14	SLC4A11
8054	242947_at	2.713296296	1.62E-11	RP4-680D5.8
8055	204719_at	2.713385185	6.98E-11	ABCA8
8056	224505_s_at	2.714040741	2.51E-10	PLCD4
8057	211890_x_at	2.719292593	8.12E-12	CAPN3
8058	236682_at	2.721766667	1.75E-10	
8059	228929_at	2.721777778	1.15E-12	DNASE1
8060	205978_at	2.721811111	3.14E-10	KL
8061	244410_at	2.722344444	2.64E-10	PKHD1
8062	226492_at	2.725055556	3.43E-13	SEMA6D
8063	209613_s_at	2.726307407	3.55E-07	ADH1B
8064	236044_at	2.728385185	7.42E-13	PPAPDC1A
8065	1556159_at	2.730611111	6.07E-14	
8066	244112_x_at	2.730922222	1.28E-11	
8067	207148_x_at	2.732522222	2.66E-07	MYOZ2
8068	1559280_a_at	2.73272963	1.92E-06	APELA
8069	207729_at	2.733648148	6.34E-10	CDH9
8070	223623_at	2.737181481	4.64E-07	C2orf40
8071	1563386_at	2.737266667	1.13E-14	
8072	223075_s_at	2.737948148	5.11E-10	AIF1L
8073	1553319_at	2.738062963	3.19E-09	OXGR1

8074	205044_at	2.739225926	6.06E-06	GABRP
8075	240114_s_at	2.739233333	1.32E-13	TMEM174
8076	206876_at	2.739774074	2.48E-09	SIM1
8077	205776_at	2.740185185	1.53E-12	FMO5
8078	223895_s_at	2.74152963	7.48E-10	EPN3
8079	1555203_s_at	2.742144444	2.57E-06	SLC44A4
8080	243163_at	2.743144444	1.81E-12	HECW1
8081	228560_at	2.74322963	4.11E-07	CHDH
8082	221589_s_at	2.743714815	3.94E-18	ALDH6A1
8083	233958_at	2.746788889	1.31E-14	
8084	231230_at	2.747744444	3.17E-09	KCNK10
8085	219987_at	2.749718519	1.06E-11	ERVMER34-1
8086	231372_at	2.75162963	1.02E-09	SLC25A48
8087	238575_at	2.753451852	5.91E-09	OSBPL6
8088	229034_at	2.755555556	3.98E-13	SOBP
8089	244742_at	2.760922222	2.59E-07	LOC101929036 /// PAH
8090	244289_at	2.765611111	6.61E-13	ZNF300P1
8091	240059_at	2.766922222	1.10E-07	
8092	228912_at	2.768785185	1.18E-07	VIL1
8093	242536_at	2.770492593	2.28E-08	
8094	207007_at	2.771955556	2.92E-13	NR1I3
8095	229954_at	2.773422222	1.16E-10	CHDH
8096	240661_at	2.774481481	1.33E-10	LOC100506459
8097	210831_s_at	2.775181481	2.33E-07	PTGER3
8098	213782_s_at	2.778518519	2.09E-07	MYOZ2
8099	223582_at	2.779166667	1.12E-11	GPR98
8100	219127_at	2.77937037	5.05E-11	PRR15L
8101	210472_at	2.780077778	2.16E-08	MT1G
8102	225809_at	2.7801	1.39E-09	PARM1
8103	229313_at	2.781051852	2.06E-09	ANO5
8104	205083_at	2.7815	6.77E-08	AOX1
8105	210227_at	2.784240741	2.41E-07	DLGAP2
8106	235247_at	2.786418519	1.99E-11	
8107	204745_x_at	2.78717037	6.39E-12	MT1G
8108	217973_at	2.788011111	3.17E-16	DCXR
8109	233498_at	2.78952963	2.04E-13	ERBB4
8110	237334_at	2.791425926	1.51E-12	SFXN2
8111	231223_at	2.792951852	1.76E-07	CSMD1
8112	242579_at	2.793951852	4.50E-16	BMPR1B
8113	210619_s_at	2.794144444	2.48E-10	HYAL1
8114	205350_at	2.795166667	2.25E-11	CRABP1
8115	210375_at	2.795792593	4.00E-08	PTGER3
8116	228502_at	2.796048148	4.42E-09	RPS6KA6

8117	229730_at	2.796233333	4.71E-07	SMTNL2
8118	206517_at	2.796611111	9.70E-09	CDH16
8119	205343_at	2.796948148	5.61E-08	SULT1C2
8120	204260_at	2.797044444	1.36E-10	CHGB
8121	204653_at	2.797925926	2.49E-09	TFAP2A
8122	228360_at	2.798185185	9.23E-13	LYPD6B
8123	220624_s_at	2.79937037	1.97E-07	ELF5
8124	205769_at	2.799762963	4.95E-10	SLC27A2
8125	205768_s_at	2.801951852	3.44E-11	SLC27A2
8126	237063_at	2.803896296	1.90E-12	RP11-44F14.8
8127	231018_at	2.804822222	4.34E-10	PALM3
8128	229039_at	2.812688889	8.15E-12	SYN2
8129	214069_at	2.813914815	3.90E-07	ACSM2A /// ACSM2B
8130	238544_at	2.814448148	1.28E-07	
8131	206269_at	2.815314815	3.23E-09	GCM1
8132	239673_at	2.81657037	1.14E-11	
8133	221795_at	2.816762963	3.61E-10	NTRK2
8134	230472_at	2.81897037	8.72E-12	IRX1
8135	231711_at	2.818977778	2.98E-09	ACPP
8136	227695_at	2.822033333	1.27E-09	GLYATL1 /// LOC100287413
8137	206209_s_at	2.822948148	1.60E-08	CA4
8138	241483_at	2.825111111	6.71E-11	CLNK
8139	228717_at	2.827607407	8.92E-15	LOC102723864
8140	219747_at	2.830444444	5.14E-08	NDNF
8141	1564236_at	2.831555556	5.55E-08	
8142	223806_s_at	2.832248148	1.40E-10	NAPSA
8143	243708_at	2.834659259	2.34E-12	TMEM132E
8144	219140_s_at	2.835318519	4.71E-07	RBP4
8145	226018_at	2.835896296	9.13E-12	MTURN
8146	236234_at	2.837311111	1.66E-07	PDE1A
8147	229485_x_at	2.842703704	4.65E-11	SHISA3
8148	205229_s_at	2.843562963	2.43E-09	COCH
8149	236741_at	2.844914815	1.63E-09	WDR72
8150	206067_s_at	2.846759259	7.38E-06	WT1
8151	229796_at	2.850159259	3.24E-12	SIX4
8152	213992_at	2.850977778	8.18E-14	COL4A6
8153	243432_at	2.85257037	2.83E-10	CHL1-AS2 /// CHL1-AS2
8154	205682_x_at	2.857622222	6.30E-11	APOM
8155	202554_s_at	2.858151852	1.91E-12	GSTM3
8156	226281_at	2.85867037	4.19E-09	DNER
8157	244111_at	2.862088889	6.23E-07	KRT222
8158	223893_at	2.863992593	3.13E-09	ENAM
8159	244370_at	2.86457037	1.61E-06	KIAA2022

8160	218952_at	2.8677	5.43E-10	PCSK1N
8161	205597_at	2.868714815	1.01E-07	SLC44A4
8162	1554842_at	2.872681481	4.38E-12	SLC12A1
8163	210476_s_at	2.881574074	5.31E-12	PRLR
8164	219773_at	2.884455556	1.34E-13	NOX4
8165	219142_at	2.884566667	3.88E-16	RASL11B
8166	219655_at	2.886081481	9.90E-14	SUGCT
8167	244119_at	2.886288889	4.51E-08	LINC00551
8168	1557176_a_at	2.892477778	3.79E-11	C14orf37
8169	205780_at	2.893511111	4.51E-13	BIK
8170	231930_at	2.898766667	3.75E-09	ELMOD1 /// LOC643923
8171	231154_x_at	2.901437037	2.26E-06	TINAG
8172	219850_s_at	2.902974074	9.37E-10	EHF
8173	205778_at	2.903337037	1.05E-10	KLK7
8174	214999_s_at	2.903962963	4.25E-12	RAB11FIP3
8175	203435_s_at	2.903977778	9.37E-09	MME
8176	228035_at	2.906688889	1.78E-07	STK33
8177	220727_at	2.908718519	2.86E-16	KCNK10
8178	243743_at	2.910759259	2.15E-12	
8179	205666_at	2.914548148	4.55E-08	FMO1
8180	241949_at	2.915111111	3.75E-09	ACOT6
8181	230554_at	2.915951852	2.48E-06	ACSM2A
8182	219738_s_at	2.919655556	4.13E-09	PCDH9
8183	229476_s_at	2.920514815	2.14E-08	THRSP
8184	229797_at	2.92077037	4.95E-10	MCOLN3
8185	231773_at	2.92102963	3.44E-08	ANGPTL1
8186	231358_at	2.921214815	1.81E-12	MRO
8187	204534_at	2.925511111	3.77E-08	SEBOX /// VTN
8188	1562587_at	2.925892593	9.55E-11	CLNK
8189	222372_at	2.926459259	2.20E-09	MAGI1
8190	223821_s_at	2.931196296	1.11E-08	SUSD4
8191	206410_at	2.938962963	5.78E-11	NR0B2
8192	224412_s_at	2.939359259	8.37E-10	TRPM6
8193	206522_at	2.942803704	4.95E-07	MGAM
8194	1552757_s_at	2.943255556	4.55E-08	C9orf66
8195	218918_at	2.944333333	8.06E-16	MAN1C1
8196	231540_at	2.944403704	4.46E-12	LOC100130691
8197	228915_at	2.945862963	8.57E-12	DACH1
8198	241583_x_at	2.94677037	1.34E-07	
8199	223062_s_at	2.947162963	1.09E-10	PSAT1
8200	211885_x_at	2.947733333	6.19E-10	FUT6
8201	233040_at	2.948944444	3.42E-08	PLEKHA5
8202	214324_at	2.952033333	2.90E-09	GP2

8203	237049_at	2.953985185	4.87E-10	
8204	234224_at	2.954022222	1.47E-07	
8205	209220_at	2.955833333	2.11E-16	GPC3
8206	236407_at	2.957314815	2.16E-11	KCNE1
8207	205413_at	2.959337037	1.44E-12	MPPED2
8208	213651_at	2.961318519	4.67E-14	INPP5J
8209	237030_at	2.962403704	8.63E-12	ACPP
8210	207076_s_at	2.962981481	5.12E-14	ASS1
8211	224485_s_at	2.963714815	4.04E-10	SLC30A2
8212	221023_s_at	2.971733333	3.42E-11	KCNH6
8213	216699_s_at	2.973225926	3.60E-13	KLK1
8214	237736_at	2.97817037	3.26E-15	BSND
8215	206964_at	2.978681481	3.19E-08	NAT8B
8216	205934_at	2.978814815	1.31E-15	PLCL1
8217	240807_at	2.980948148	1.25E-11	LINC01187
8218	214602_at	2.981140741	5.75E-09	COL4A4
8219	207299_s_at	2.981944444	1.05E-10	GRM1
8220	231213_at	2.982125926	1.32E-09	PDE1A
8221	241914_s_at	2.982222222	1.92E-07	ACSM2A /// ACSM2B
8222	209604_s_at	2.98272963	4.03E-13	GATA3
8223	215807_s_at	2.983359259	7.37E-12	PLXNB1
8224	209981_at	2.983822222	1.19E-11	CSDC2
8225	230130_at	2.985348148	2.21E-11	SLIT2
8226	204931_at	2.985681481	8.45E-11	TCF21
8227	232361_s_at	2.990633333	1.94E-08	EHF
8228	210524_x_at	2.990655556	6.86E-12	
8229	1556300_s_at	2.993137037	9.36E-07	SIM1
8230	232245_at	2.995318519	1.32E-08	SLC25A34
8231	236220_at	2.997488889	1.11E-11	SLC16A10
8232	209602_s_at	3.008955556	1.25E-10	GATA3
8233	227202_at	3.009785185	2.69E-08	CNTN1
8234	210289_at	3.011022222	6.83E-06	NAT8 /// NAT8B
8235	206930_at	3.0114	3.96E-08	GLYAT
8236	210049_at	3.011903704	3.25E-09	SERPINC1
8237	230912_at	3.0153	1.19E-11	ASPDH
8238	231260_at	3.017822222	1.08E-10	ZNF582-AS1
8239	230493_at	3.018574074	4.07E-10	SHISA2
8240	230964_at	3.019451852	2.52E-10	FREM2
8241	222257_s_at	3.019592593	3.01E-06	ACE2
8242	205757_at	3.020466667	6.40E-15	ENTPD5
8243	207016_s_at	3.0208	6.98E-11	ALDH1A2 /// LOC101928635
8244	211917_s_at	3.021414815	1.79E-09	PRLR
8245	239765_at	3.022311111	1.25E-12	CPEB3

8246	216452_at	3.026396296	1.88E-10	TRPM3
8247	209591_s_at	3.029525926	1.99E-11	BMP7
8248	210944_s_at	3.035281481	3.05E-15	CAPN3
8249	209603_at	3.043796296	3.64E-09	GATA3
8250	239093_at	3.045022222	2.23E-14	HOGA1
8251	1552281_at	3.04612963	5.72E-11	SLC39A5
8252	1556156_at	3.047025926	2.07E-12	ESRRB
8253	236959_s_at	3.048755556	1.13E-10	
8254	213933_at	3.049155556	9.32E-11	PTGER3
8255	210179_at	3.051503704	3.42E-08	KCNJ13
8256	210326_at	3.052674074	5.41E-09	AGXT
8257	240192_at	3.052840741	1.30E-09	GATA3-AS1
8258	222930_s_at	3.053018519	4.60E-12	AGMAT
8259	233180_at	3.059396296	2.37E-12	
8260	219949_at	3.060981481	3.82E-11	LRRC2
8261	205364_at	3.063714815	2.00E-11	ACOX2
8262	206346_at	3.064566667	4.67E-13	PRLR
8263	236972_at	3.070888889	3.43E-09	TRIM63
8264	204836_at	3.072507407	1.53E-13	GLDC
8265	228504_at	3.074392593	3.98E-13	SCN7A
8266	219476_at	3.075214815	2.30E-09	C1orf116
8267	206558_at	3.076974074	3.63E-11	SIM2
8268	213362_at	3.078907407	4.74E-12	PTPRD
8269	221662_s_at	3.081781481	2.83E-16	SLC22A7
8270	243276_at	3.085688889	6.52E-12	ALS2CL
8271	202992_at	3.088603704	6.72E-10	C7
8272	231150_at	3.089774074	7.53E-14	RP11-61L19.2
8273	237839_at	3.094233333	3.88E-10	
8274	223732_at	3.095725926	1.81E-12	SLC23A1
8275	235515_at	3.096011111	1.12E-13	SYNE4
8276	228018_at	3.098544444	7.11E-07	NKAIN4
8277	231195_at	3.099455556	3.14E-13	KLRG2
8278	1562309_s_at	3.106081481	6.25E-10	PHF21B
8279	242269_at	3.107759259	6.79E-15	LINC00982
8280	243799_x_at	3.112022222	6.93E-10	ANGPTL3
8281	205472_s_at	3.112266667	3.52E-11	DACH1
8282	209696_at	3.112814815	1.89E-14	FBP1
8283	224189_x_at	3.114540741	2.91E-11	EHF
8284	230238_at	3.11522963	6.10E-11	SOWAHA
8285	1560784_x_at	3.115825926	1.45E-13	
8286	210408_s_at	3.116044444	7.00E-09	CPNE6
8287	202921_s_at	3.124374074	8.36E-13	ANK2
8288	237450_at	3.125488889	1.68E-12	LOC389332

8289	210085_s_at	3.126811111	1.64E-12	ANXA9
8290	226846_at	3.128155556	4.56E-14	PHYHD1
8291	231275_at	3.128766667	2.25E-09	LINC00982
8292	202286_s_at	3.130448148	2.27E-08	TACSTD2
8293	244330_at	3.132525926	1.35E-10	
8294	235129_at	3.13292963	1.16E-09	PPP1R1A
8295	243168_at	3.139733333	1.48E-07	
8296	1558323_at	3.141088889	1.31E-09	TMEM72
8297	244479_at	3.141281481	7.34E-11	
8298	205477_s_at	3.141611111	1.81E-10	AMBP
8299	219255_x_at	3.143274074	5.10E-08	IL17RB
8300	1559400_s_at	3.144281481	2.99E-12	PAPPA
8301	230822_at	3.146203704	2.25E-12	TMEM61
8302	1552549_a_at	3.147355556	5.01E-18	BSND
8303	213285_at	3.148666667	2.20E-15	TMEM30B
8304	210065_s_at	3.148825926	1.52E-08	UPK1B
8305	232422_at	3.149396296	2.60E-13	GGACT
8306	220303_at	3.151818519	3.47E-09	PDZD3
8307	209560_s_at	3.152703704	2.34E-08	DLK1
8308	222712_s_at	3.15567037	6.82E-09	MUC13
8309	227174_at	3.158603704	3.86E-10	WDR72
8310	231416_at	3.161477778	1.25E-09	DHDH
8311	205154_at	3.163540741	2.49E-13	LRRN2
8312	219461_at	3.16437037	4.97E-11	PAK6
8313	239964_at	3.166251852	3.25E-11	TCL6
8314	232202_at	3.166948148	6.36E-10	FAM83B
8315	240450_at	3.168511111	2.59E-11	
8316	206955_at	3.173248148	4.83E-11	AQP7 /// LOC100509620 /// LOC101930168
8317	203913_s_at	3.175737037	3.29E-10	HPGD
8318	243955_at	3.177314815	1.27E-13	
8319	1561195_at	3.178185185	3.74E-10	
8320	230084_at	3.186762963	1.53E-13	SLC30A2
8321	206119_at	3.188581481	1.63E-07	BHMT
8322	231052_at	3.197411111	2.95E-12	GLOD5
8323	205557_at	3.198418519	1.78E-09	BPI /// LOC149684
8324	219643_at	3.199418519	5.25E-11	LRP1B
8325	213438_at	3.204	8.63E-11	NFASC
8326	206692_at	3.204337037	2.93E-12	KCNJ10
8327	209757_s_at	3.207066667	4.49E-14	MYCN
8328	224156_x_at	3.20937037	4.83E-08	IL17RB
8329	214088_s_at	3.212218519	1.87E-14	FUT3
8330	235904_at	3.21467037	5.16E-08	UGT3A1
8331	206717_at	3.214933333	3.09E-06	MYH8

8332	234150_at	3.2179	8.32E-09	
8333	235048_at	3.218718519	1.34E-12	FAM169A
8334	214910_s_at	3.221540741	1.57E-10	APOM
8335	202740_at	3.223455556	3.10E-17	ABHD14A-ACY1 /// ACY1
8336	229975_at	3.224566667	2.53E-11	BMPR1B
8337	215129_at	3.226325926	4.43E-14	PIK3C2G
8338	232494_at	3.2299	1.62E-10	CYP8B1
8339	220135_s_at	3.238740741	2.40E-10	SLC7A9
8340	239291_at	3.238796296	7.72E-09	TRPM3
8341	1560782_at	3.240607407	2.24E-14	
8342	225846_at	3.246033333	4.42E-10	ESRP1
8343	235746_s_at	3.246774074	3.68E-11	PLA2R1
8344	227794_at	3.254955556	2.40E-10	GLYATL1 /// LOC100287413
8345	209590_at	3.256525926	3.51E-13	BMP7
8346	1559131_a_at	3.256740741	2.80E-12	LOC101928047
8347	206872_at	3.260407407	5.71E-09	SLC17A1
8348	236774_at	3.270544444	5.92E-10	
8349	228194_s_at	3.276474074	7.70E-10	SORCS1
8350	216604_s_at	3.28347037	1.43E-11	SLC7A8
8351	205777_at	3.286507407	9.85E-14	DUSP9
8352	230642_at	3.287114815	8.27E-12	RP11-297L17.2
8353	240323_at	3.28742963	8.59E-15	RP11-728F11.4
8354	206065_s_at	3.287451852	1.45E-08	DPYS
8355	1555112_a_at	3.291696296	8.63E-14	CCDC181
8356	219737_s_at	3.292255556	4.95E-12	PCDH9
8357	233504_at	3.292607407	4.87E-11	C9orf84
8358	242483_at	3.296755556	2.87E-14	
8359	204476_s_at	3.296974074	1.08E-13	PC
8360	243669_s_at	3.297092593	1.06E-07	PRAP1
8361	220376_at	3.299651852	1.94E-08	LRRC19
8362	244353_s_at	3.302988889	1.29E-15	SLC2A12
8363	230319_at	3.305433333	1.37E-08	
8364	239183_at	3.308025926	4.62E-10	ANGPTL1
8365	222073_at	3.310644444	2.84E-10	COL4A3
8366	219277_s_at	3.310944444	1.37E-10	OGDHL
8367	220393_at	3.314837037	2.77E-07	LGSN
8368	234310_s_at	3.315407407	1.27E-11	SUSD2
8369	210397_at	3.320425926	5.48E-11	DEFB1
8370	236523_at	3.321437037	2.61E-09	LOC285556
8371	215389_s_at	3.326518519	5.74E-13	TNNT2
8372	231208_at	3.337325926	7.37E-14	SLC13A3
8373	229659_s_at	3.338081481	1.56E-09	
8374	237639_at	3.34012963	3.98E-13	TMEM207

8375	215729_s_at	3.3421	1.22E-10	VGLL1
8376	219735_s_at	3.343407407	1.54E-19	TFCP2L1
8377	232606_at	3.345822222	1.29E-08	ANK2
8378	220100_at	3.346144444	2.18E-08	SLC22A11
8379	220675_s_at	3.347285185	3.97E-13	PNPLA3
8380	217617_at	3.355966667	2.33E-11	
8381	223702_x_at	3.35777037	1.58E-11	FTCD
8382	214842_s_at	3.359122222	2.44E-10	ALB
8383	230585_at	3.360062963	9.82E-11	KCNJ15
8384	230686_s_at	3.360362963	2.10E-14	SLC13A3
8385	220484_at	3.360674074	7.76E-10	MCOLN3
8386	205478_at	3.360688889	3.45E-08	PPP1R1A
8387	209343_at	3.368648148	6.19E-16	EFHD1
8388	211882_x_at	3.370348148	4.18E-10	FUT6
8389	217014_s_at	3.370614815	1.56E-14	AZGP1 /// AZGP1P1
8390	208240_s_at	3.376514815	2.78E-14	FGF1
8391	244530_at	3.376788889	1.81E-13	
8392	229254_at	3.378751852	1.91E-09	MFSD4
8393	203914_x_at	3.378811111	1.23E-11	HPGD
8394	1555553_a_at	3.382911111	4.33E-12	SLC22A7
8395	217248_s_at	3.382977778	3.07E-13	SLC7A8
8396	220059_at	3.385577778	5.15E-14	STAP1
8397	242967_at	3.386	2.24E-07	
8398	231051_at	3.387788889	4.94E-16	LINC00948
8399	221648_s_at	3.390148148	2.85E-10	AGMAT
8400	221008_s_at	3.390477778	1.61E-10	ETNPPL
8401	216039_at	3.39237037	6.18E-14	GABRA2
8402	242340_at	3.397040741	1.56E-10	RP1-78O14.1
8403	243672_at	3.408459259	1.53E-10	SALL3
8404	222325_at	3.414388889	6.02E-11	
8405	1553989_a_at	3.422385185	2.20E-16	ATP6V1C2
8406	1555612_s_at	3.423037037	5.41E-10	G6PC
8407	217628_at	3.424614815	1.14E-11	CLIC5
8408	232424_at	3.424851852	4.23E-12	PRDM16
8409	229158_at	3.425548148	1.31E-10	WNK4
8410	229740_at	3.429159259	1.15E-19	SMIM5
8411	220148_at	3.431196296	2.63E-10	ALDH8A1
8412	203256_at	3.433474074	1.45E-09	CDH3
8413	209966_x_at	3.435744444	5.43E-14	ESRRG
8414	207213_s_at	3.436366667	9.38E-10	USP2
8415	221590_s_at	3.4386	1.20E-18	ALDH6A1
8416	231517_at	3.440759259	4.81E-11	ZYG11A
8417	214774_x_at	3.441607407	9.75E-09	TOX3

8418	226597_at	3.448074074	5.67E-15	REEP6
8419	214475_x_at	3.457103704	1.89E-13	CAPN3
8420	216603_at	3.458681481	1.94E-11	SLC7A8
8421	211548_s_at	3.461766667	3.87E-11	HPGD
8422	206672_at	3.462896296	2.15E-18	AQP2
8423	203722_at	3.467862963	5.59E-16	ALDH4A1
8424	202920_at	3.470481481	2.09E-15	ANK2
8425	204997_at	3.471988889	1.31E-09	GPD1
8426	216782_at	3.479877778	1.52E-09	
8427	223979_x_at	3.4904	1.60E-11	FTCD
8428	206721_at	3.495351852	1.41E-13	CCDC181
8429	228233_at	3.496214815	5.45E-13	FREM1
8430	206963_s_at	3.497003704	4.40E-07	NAT8 /// NAT8B
8431	223699_at	3.506685185	1.04E-11	CNDP1
8432	213845_at	3.507848148	6.82E-12	GRIK2
8433	216623_x_at	3.509777778	4.33E-08	TOX3
8434	206529_x_at	3.511551852	3.95E-15	SLC26A4
8435	224299_x_at	3.518185185	6.10E-11	FTCD
8436	214347_s_at	3.523044444	3.27E-07	DDC
8437	224942_at	3.526525926	2.05E-18	PAPPA
8438	241476_at	3.526911111	1.00E-09	
8439	242998_at	3.527125926	8.30E-14	RDH12
8440	1556554_at	3.531355556	1.59E-12	TRIM50
8441	216092_s_at	3.531403704	5.43E-18	SLC7A8
8442	221605_s_at	3.540651852	1.55E-14	PIPOX
8443	207014_at	3.545685185	1.19E-12	GABRA2
8444	201981_at	3.546044444	2.57E-13	PAPPA
8445	230323_s_at	3.554711111	2.78E-09	TMEM45B
8446	215108_x_at	3.556425926	1.42E-09	TOX3
8447	219389_at	3.557822222	6.44E-12	SUSD4
8448	210398_x_at	3.5638	7.80E-10	FUT6
8449	1561951_at	3.570788889	1.53E-07	SLC5A12
8450	220502_s_at	3.579037037	1.14E-08	SLC13A1
8451	1553746_a_at	3.580711111	6.16E-09	OTOGL
8452	204591_at	3.585340741	1.10E-10	CHL1
8453	230889_at	3.595066667	4.17E-15	LOC645321
8454	210327_s_at	3.59647037	5.06E-12	AGXT
8455	207567_at	3.598151852	1.44E-13	SLC13A2
8456	239420_at	3.601037037	7.90E-18	LINC01187
8457	220332_at	3.601785185	4.17E-10	CLDN16
8458	228546_at	3.604151852	2.42E-11	DPP6
8459	224941_at	3.604818519	3.99E-16	PAPPA
8460	211712_s_at	3.6085	5.06E-12	ANXA9

8461	228236_at	3.611151852	1.07E-14	SLC52A3
8462	207650_x_at	3.61647037	1.04E-12	PTGER1
8463	210451_at	3.619862963	4.18E-09	PKLR
8464	216219_at	3.621088889	2.62E-15	AQP6
8465	1552755_at	3.625225926	5.13E-08	C9orf66
8466	205544_s_at	3.63472963	2.82E-09	CR2
8467	227450_at	3.636133333	1.03E-17	ERP27
8468	223618_at	3.639633333	4.35E-11	FMN2
8469	231632_at	3.64	1.77E-16	ERICH4
8470	213706_at	3.642559259	6.16E-11	GPD1
8471	214389_at	3.64487037	1.63E-09	SLC5A12
8472	239791_at	3.652955556	1.54E-14	HOXB-AS3
8473	223939_at	3.656392593	2.99E-13	SUCNR1
8474	228128_x_at	3.66902963	1.06E-15	PAPPA
8475	241726_at	3.680388889	6.06E-11	
8476	208034_s_at	3.681711111	3.22E-14	PROZ
8477	205676_at	3.682962963	1.64E-14	CYP27B1
8478	219840_s_at	3.68432963	3.45E-15	TCL6
8479	238919_at	3.689285185	2.29E-11	PCDH9
8480	243901_at	3.690488889	2.81E-12	ALDOB
8481	227971_at	3.703625926	5.35E-15	NRK
8482	205311_at	3.706996296	2.06E-07	DDC
8483	204290_s_at	3.708588889	3.10E-17	ALDH6A1
8484	211384_s_at	3.713833333	2.71E-14	CASR
8485	227480_at	3.714174074	8.30E-14	SUSD2
8486	229229_at	3.715688889	2.53E-08	AGXT2
8487	230406_at	3.715985185	4.39E-15	
8488	218960_at	3.722418519	4.45E-11	TMPRSS4
8489	220318_at	3.725151852	5.96E-15	EPN3
8490	229177_at	3.7319	2.57E-12	C16orf89
8491	215563_s_at	3.743251852	1.62E-10	MST1L
8492	206262_at	3.747533333	5.51E-11	ADH1C
8493	206552_s_at	3.750237037	1.72E-09	TAC1
8494	211231_x_at	3.759766667	7.47E-13	CYP4A11
8495	209459_s_at	3.761285185	2.07E-17	ABAT
8496	231311_at	3.764651852	4.99E-13	
8497	204268_at	3.769966667	6.67E-16	S100A2
8498	205984_at	3.775907407	6.81E-13	CRHBP
8499	204607_at	3.779537037	1.71E-09	HMGCS2
8500	238689_at	3.780218519	2.45E-12	GPR110
8501	230081_at	3.784322222	3.18E-12	PLCXD3
8502	1558324_a_at	3.804055556	2.21E-11	TMEM72
8503	202035_s_at	3.805607407	1.70E-12	SFRP1

8504	221661_at	3.807485185	2.60E-12	SLC22A7
8505	227629_at	3.809803704	9.46E-19	PRLR
8506	205216_s_at	3.814333333	7.76E-14	APOH
8507	231424_at	3.817185185	8.69E-10	SLC5A12
8508	216696_s_at	3.81727037	4.43E-12	PRODH2
8509	221588_x_at	3.829437037	7.85E-19	ALDH6A1
8510	220554_at	3.830796296	3.75E-18	SLC22A7
8511	219803_at	3.832311111	1.97E-09	ANGPTL3
8512	231667_at	3.842203704	2.05E-09	SLC39A5
8513	236892_s_at	3.850711111	5.40E-17	HOXB-AS3
8514	229241_at	3.862959259	5.05E-15	LDHD
8515	237254_at	3.8691	1.46E-15	SLC5A11
8516	209173_at	3.877966667	3.23E-10	AGR2
8517	1552797_s_at	3.882744444	4.92E-15	PROM2
8518	206286_s_at	3.88407037	6.75E-09	TDGF1 /// TDGF1P3
8519	206878_at	3.887892593	2.47E-15	DAO
8520	231982_at	3.89917037	1.16E-07	SMIM24
8521	204777_s_at	3.899781481	2.21E-12	MAL
8522	227506_at	3.903774074	6.53E-12	SLC16A9
8523	1569926_s_at	3.906177778	7.40E-14	SLC34A3
8524	233155_at	3.908614815	1.31E-14	UPP2
8525	203824_at	3.912025926	5.16E-08	TSPAN8
8526	224300_x_at	3.914211111	4.78E-11	FTCD
8527	208383_s_at	3.915496296	2.42E-10	PCK1
8528	207544_s_at	3.915585185	9.05E-12	ADH6
8529	231683_at	3.918862963	2.48E-14	GLYAT
8530	226147_s_at	3.924155556	1.31E-09	PIGR
8531	205820_s_at	3.927637037	1.18E-12	APOC3
8532	236088_at	3.935977778	1.20E-11	NTNG1
8533	215274_at	3.936592593	1.16E-16	SLC12A3
8534	224940_s_at	3.936974074	1.52E-17	PAPPA
8535	230883_at	3.938185185	3.42E-11	NXPH2
8536	224480_s_at	3.944481481	1.69E-17	AGPAT9
8537	242372_s_at	3.950803704	4.78E-12	MFSD4
8538	1554931_at	3.952096296	3.74E-12	CYP4A11 /// CYP4A22
8539	233712_at	3.954703704	7.34E-17	LOC101060181 /// LOC101928574 /// ZNF44
8540	202752_x_at	3.956688889	6.43E-15	SLC7A8
8541	238625_at	3.966496296	7.85E-10	C1orf168
8542	229580_at	3.97307037	3.94E-10	CLSTN2
8543	1563793_at	3.985966667	1.79E-12	LOC100130278
8544	243200_at	3.988237037	3.68E-13	RP11-369C8.1
8545	206755_at	3.992796296	7.83E-16	CYP2B6
8546	230830_at	4.012248148	2.10E-10	SLC51B

8547	1553394_a_at	4.012355556	5.97E-14	TFAP2B
8548	231496_at	4.020414815	1.40E-10	FCAMR
8549	220503_at	4.022925926	7.35E-08	SLC13A1
8550	220604_x_at	4.023159259	6.30E-11	FTCD
8551	205464_at	4.033877778	2.57E-09	SCNN1B
8552	207444_at	4.034496296	1.70E-11	SLC22A13
8553	210577_at	4.043414815	3.26E-15	CASR
8554	240378_at	4.051796296	4.37E-16	LOC101929040
8555	219768_at	4.052122222	1.33E-13	VTCN1
8556	208006_at	4.0524	1.16E-16	FOXI1
8557	1562046_at	4.055203704	1.50E-11	LOC101927244
8558	204289_at	4.062881481	7.76E-14	ALDH6A1
8559	1552766_at	4.06927037	4.07E-11	HS6ST2
8560	243418_at	4.069903704	5.99E-15	
8561	217319_x_at	4.078544444	2.61E-14	CYP4A11 /// CYP4A22
8562	1560383_at	4.079322222	2.35E-12	BC042091 /// RP11-752D24.2
8563	240320_at	4.112159259	5.93E-15	RNF212B
8564	220625_s_at	4.113688889	1.40E-12	ELF5
8565	231480_at	4.117366667	7.18E-14	SLC6A19
8566	209309_at	4.119022222	3.05E-13	AZGP1
8567	204965_at	4.120088889	2.44E-06	GC
8568	217530_at	4.126907407	3.32E-17	SLC34A1
8569	214261_s_at	4.130611111	6.79E-15	ADH6
8570	227194_at	4.130737037	1.95E-14	FAM3B
8571	238177_at	4.135488889	7.09E-15	SLC6A19
8572	220310_at	4.137455556	1.11E-15	TUBAL3
8573	231256_at	4.14082963	6.66E-14	LOC727944
8574	242375_x_at	4.166792593	1.13E-12	LOC101929036 /// PAH
8575	220724_at	4.167166667	2.51E-14	CWH43
8576	231156_at	4.172681481	1.40E-14	HAO2
8577	244337_at	4.178785185	7.40E-14	
8578	207981_s_at	4.180774074	6.11E-13	ESRRG
8579	244384_at	4.193362963	5.27E-16	
8580	229302_at	4.200381481	1.51E-18	TMEM178A
8581	218844_at	4.217466667	9.30E-19	ACSF2
8582	223986_x_at	4.220018519	1.56E-17	DMRT2
8583	220510_at	4.223811111	5.75E-18	RHBG
8584	228462_at	4.240696296	3.24E-10	IRX2
8585	213456_at	4.24572963	1.43E-08	SOSTDC1
8586	205675_at	4.245907407	4.44E-15	MTTP
8587	239270_at	4.262181481	4.83E-12	PLCXD3
8588	228740_at	4.262255556	9.16E-17	RP11-999E24.3
8589	206259_at	4.268592593	7.16E-14	PROC

8590	219866_at	4.273559259	2.02E-13	CLIC5
8591	207378_at	4.279474074	7.81E-10	TREH
8592	237350_at	4.27957037	5.96E-15	TTC36
8593	229057_at	4.282074074	7.10E-16	SCN2A
8594	220139_at	4.285522222	4.40E-13	DNMT3L
8595	1553970_s_at	4.287074074	6.18E-12	CEL
8596	231677_at	4.288662963	7.60E-15	
8597	209460_at	4.308107407	8.37E-18	ABAT
8598	223550_s_at	4.350659259	3.72E-14	CA10
8599	205910_s_at	4.361011111	2.17E-12	CEL
8600	206254_at	4.364618519	2.71E-14	EGF
8601	1557474_at	4.365151852	2.23E-15	LOC284578
8602	207407_x_at	4.365518519	2.67E-12	CYP4A11
8603	215059_at	4.366722222	9.23E-11	
8604	235915_at	4.368081481	2.29E-14	
8605	1553155_x_at	4.371414815	1.96E-18	ATP6V0D2
8606	224179_s_at	4.373225926	3.37E-14	MIOX
8607	231217_at	4.374651852	1.36E-14	SLC13A3
8608	220801_s_at	4.380133333	1.03E-12	HAO2
8609	204393_s_at	4.393603704	1.04E-12	ACPP
8610	237250_at	4.395922222	6.63E-14	RP11-320H14.1
8611	31835_at	4.402518519	2.74E-17	HRG
8612	222083_at	4.408162963	8.47E-10	GLYAT
8613	216223_at	4.420222222	4.08E-11	CPN2
8614	205473_at	4.422777778	1.96E-16	ATP6V1B1
8615	212768_s_at	4.438474074	3.75E-09	OLFM4
8616	205502_at	4.441151852	6.56E-16	CYP17A1
8617	1553153_at	4.44312963	1.44E-19	ATP6V0D2
8618	211357_s_at	4.464996296	9.58E-13	ALDOB
8619	240285_at	4.46822963	2.82E-13	AQP2
8620	244216_at	4.496714815	5.27E-16	
8621	242275_at	4.503648148	8.83E-18	
8622	208177_at	4.511737037	1.04E-17	SLC34A1
8623	214788_x_at	4.514940741	1.73E-13	DDN
8624	235988_at	4.521862963	5.99E-15	GPR110
8625	220889_s_at	4.528044444	2.73E-15	CA10
8626	217583_at	4.538166667	2.50E-14	LOC101929036 /// PAH
8627	225645_at	4.544781481	5.52E-12	EHF
8628	1552532_a_at	4.547559259	5.85E-16	ATP6V1C2
8629	231070_at	4.548151852	1.29E-14	IYD
8630	236360_at	4.549548148	8.33E-17	LINC00982
8631	240033_at	4.560966667	1.66E-16	PLG
8632	233604_at	4.564859259	3.97E-13	FLJ22763

8633	236225_at	4.566085185	1.77E-16	GGT6
8634	229341_at	4.566848148	5.27E-16	TFCP2L1
8635	229831_at	4.569092593	5.13E-12	CNTN3
8636	231483_at	4.569818519	3.12E-10	LOC100505985
8637	1554749_s_at	4.577037037	3.25E-19	CLCNKB
8638	219732_at	4.580611111	1.22E-19	LPPR1
8639	211276_at	4.59347037	4.20E-17	TCEAL2
8640	1552721_a_at	4.595781481	1.42E-12	FGF1
8641	206514_s_at	4.619211111	3.74E-16	CYP4F2 /// CYP4F3
8642	206515_at	4.629948148	1.14E-12	CYP4F3
8643	1553151_at	4.648696296	1.38E-14	ATP6V0D2
8644	236489_at	4.666322222	1.16E-12	GPR110
8645	207771_at	4.668974074	1.90E-14	SLC5A2
8646	1564964_at	4.670733333	5.04E-13	
8647	207249_s_at	4.689037037	2.00E-17	SLC28A2
8648	203453_at	4.71767037	2.98E-13	SCNN1A
8649	218186_at	4.71767037	3.03E-14	RAB25
8650	207047_s_at	4.718403704	3.25E-19	CLCNKA /// CLCNKB
8651	205911_at	4.738633333	8.25E-16	PTH1R
8652	242601_at	4.75737037	4.91E-17	HEPACAM2
8653	220723_s_at	4.758262963	4.83E-17	CWH43
8654	230565_at	4.760451852	2.05E-18	ATP6V1G3
8655	239132_at	4.771903704	2.90E-16	NOS1
8656	237799_at	4.798255556	1.52E-17	SLC22A12
8657	205592_at	4.798996296	1.16E-16	SLC4A1
8658	229782_at	4.806703704	1.21E-14	RMST
8659	231623_at	4.80672963	2.88E-13	TMEM174
8660	231021_at	4.808644444	5.43E-16	SLC6A19
8661	207174_at	4.810974074	3.40E-13	GPC5
8662	234314_at	4.830551852	2.79E-17	RALGAPA2
8663	209904_at	4.837618519	1.20E-16	TNNC1
8664	236652_at	4.84112963	5.85E-16	LOC149703
8665	241560_at	4.859348148	1.57E-11	
8666	236374_at	4.862940741	1.27E-12	CTXN3
8667	230687_at	4.880462963	2.90E-16	SLC13A3
8668	204130_at	4.888481481	5.18E-14	HSD11B2
8669	239178_at	4.902574074	4.62E-15	FGF9
8670	209443_at	4.903781481	2.02E-15	SERPINA5
8671	207673_at	4.90392963	1.93E-14	NPHS1
8672	230030_at	4.911203704	2.17E-16	HS6ST2
8673	214451_at	4.93112963	2.51E-16	TFAP2B
8674	213317_at	4.937848148	2.49E-13	CLIC5
8675	1552767_a_at	4.950307407	6.38E-17	HS6ST2

8676	205117_at	4.952474074	2.06E-12	FGF1
8677	219554_at	4.966781481	6.36E-18	RHCG
8678	237582_at	4.973481481	4.57E-16	
8679	228581_at	4.98087037	1.03E-15	KCNJ10
8680	237587_at	4.983974074	1.22E-19	
8681	202037_s_at	5.000581481	3.88E-16	SFRP1
8682	236646_at	5.033662963	2.46E-18	TMEM52B
8683	210452_x_at	5.049244444	4.92E-15	CYP4F2
8684	240910_at	5.051111111	5.77E-16	
8685	206226_at	5.055796296	6.62E-17	HRG
8686	205694_at	5.059014815	1.47E-15	TYRP1
8687	227642_at	5.059366667	2.15E-18	TFCP2L1
8688	229529_at	5.075622222	3.17E-17	TCF21
8689	204705_x_at	5.080681481	8.46E-13	ALDOB
8690	228375_at	5.105259259	1.87E-14	IGSF11
8691	206089_at	5.108459259	4.73E-15	NELL1
8692	205751_at	5.114385185	3.27E-13	SH3GL2
8693	226553_at	5.115155556	5.65E-19	TMPRSS2
8694	223704_s_at	5.116474074	1.03E-17	DMRT2
8695	236630_at	5.117644444	9.04E-19	AQP2
8696	230931_at	5.123840741	3.75E-18	PLG
8697	205719_s_at	5.126574074	8.89E-12	LOC101929036 /// PAH
8698	231376_at	5.131792593	8.21E-17	UPP2
8699	1565228_s_at	5.15807037	7.60E-15	ALB
8700	214053_at	5.161648148	1.42E-16	ERBB4
8701	239805_at	5.164492593	6.59E-17	SLC13A2
8702	206840_at	5.167107407	1.16E-16	AFM
8703	202036_s_at	5.190777778	9.96E-15	SFRP1
8704	238248_at	5.227785185	1.96E-16	
8705	239593_at	5.233503704	1.81E-18	TMEM213
8706	205336_at	5.245014815	1.95E-17	PVALB
8707	231068_at	5.245285185	2.23E-15	SLC47A2
8708	227241_at	5.246281481	2.50E-14	MUC15
8709	229916_at	5.2589	8.66E-20	ENPP6
8710	210343_s_at	5.262940741	1.82E-13	SLC22A6
8711	210199_at	5.269722222	6.62E-17	CRYAA
8712	231398_at	5.278118519	1.47E-16	SLC22A7
8713	211689_s_at	5.292651852	2.79E-17	TMPRSS2
8714	238287_at	5.303962963	3.86E-12	SLC7A13
8715	206754_s_at	5.311651852	1.33E-15	CYP2B6 /// CYP2B7P
8716	217238_s_at	5.347381481	3.14E-13	ALDOB
8717	205983_at	5.387548148	4.68E-16	DPEP1
8718	231634_at	5.409440741	2.19E-15	SLC12A3

8719	241436_at	5.420866667	2.62E-12	SCNN1G
8720	206404_at	5.431903704	1.03E-15	FGF9
8721	223869_at	5.440711111	1.89E-13	SOST
8722	239006_at	5.453725926	3.32E-17	SLC26A7
8723	205549_at	5.467	5.97E-16	PCP4
8724	244890_at	5.52002963	1.03E-17	
8725	227238_at	5.575474074	2.24E-15	MUC15
8726	220281_at	5.583703704	5.75E-14	SLC12A1
8727	1554748_at	5.603359259	1.46E-16	CLCNKB
8728	220197_at	5.61197037	7.58E-19	ATP6V0A4
8729	216910_at	5.634837037	8.28E-19	XPNPEP2
8730	1554668_a_at	5.656825926	2.19E-14	FAM151A
8731	231058_at	5.677155556	1.19E-14	FXYD4
8732	1559992_a_at	5.677814815	1.68E-16	LINC00645
8733	210165_at	5.678959259	3.24E-19	DNASE1
8734	206952_at	5.704762963	6.47E-12	G6PC
8735	206457_s_at	5.721792593	1.22E-19	DIO1
8736	231391_at	5.745214815	5.02E-12	CTXN3
8737	240183_at	5.74667037	1.70E-17	TMEM213
8738	213967_at	5.747796296	9.79E-19	RALYL
8739	243562_at	5.76837037	1.48E-15	KNG1
8740	217512_at	5.769618519	2.47E-17	KNG1
8741	216599_x_at	5.827185185	1.11E-13	SLC22A6
8742	205243_at	5.984544444	4.24E-17	SLC13A3
8743	209977_at	5.992833333	2.79E-17	PLG
8744	233183_at	6.046125926	3.25E-19	SLC4A9
8745	206716_at	6.076503704	3.65E-14	UMOD
8746	211298_s_at	6.123325926	4.20E-17	ALB
8747	214598_at	6.176788889	1.60E-16	CLDN8
8748	206484_s_at	6.229903704	3.55E-18	XPNPEP2
8749	208354_s_at	6.231981481	1.22E-19	SLC12A3
8750	231352_at	6.320422222	2.00E-17	SLC22A8
8751	205985_x_at	6.478907407	1.54E-19	CLCNKB
8752	209978_s_at	6.528596296	5.75E-18	LPA /// PLG
8753	204704_s_at	6.547537037	1.49E-15	ALDOB
8754	210403_s_at	6.584722222	1.76E-15	KCNJ1
8755	210402_at	6.607488889	4.20E-17	KCNJ1
8756	206024_at	6.622703704	4.31E-14	HPD
8757	220424_at	6.658748148	8.59E-15	NPHS2
8758	205626_s_at	6.748314815	1.51E-16	CALB1
8759	205625_s_at	6.897766667	1.12E-16	CALB1
8760	205244_s_at	6.913914815	1.30E-16	SLC13A3
8761	205892_s_at	7.076714815	2.14E-15	FABP1

8762	206054_at	7.367192593	4.42E-17	KNG1
8763	221298_s_at	7.425681481	3.10E-17	SLC22A8
